# Cryptic Diversity in Indo-Pacific Coral-Reef Fishes Revealed by DNA-Barcoding Provides New Support to the Centre-of-Overlap Hypothesis

**DOI:** 10.1371/journal.pone.0028987

**Published:** 2012-03-15

**Authors:** Nicolas Hubert, Christopher P. Meyer, Henrich J. Bruggemann, Fabien Guérin, Roberto J. L. Komeno, Benoit Espiau, Romain Causse, Jeffrey T. Williams, Serge Planes

**Affiliations:** 1 Laboratoire ECOMAR, Faculté des Sciences et Technologies, Université de La Réunion, Saint-Denis, Réunion, France; 2 CNRS-EPHE, CRIOBE – CBETM, Université de Perpignan Via Domitia, Perpignan, France; 3 Department of Invertebrate Zoology, National Museum of Natural History, Smithsonian Institution, Washington, D.C., United States of America; 4 UMR PVBMT, Faculté des Sciences et Technologies, Université de La Réunion, Saint-Denis, Réunion, France; 5 Institut Halieutique et des Sciences Marines, Université de Toliara, Toliara, Madagascar; 6 Département Milieux et Peuplements Aquatiques, Muséum National d'Histoire Naturelle, Paris, France; 7 Department of Vertebrate Zoology, National Museum of Natural History, Smithsonian Institution, Washington, D.C., United States of America; Ecole Normale Supérieure de Lyon, France

## Abstract

Diversity in coral reef fishes is not evenly distributed and tends to accumulate in the Indo-Malay-Philippines Archipelago (IMPA). The comprehension of the mechanisms that initiated this pattern is in its infancy despite its importance for the conservation of coral reefs. Considering the IMPA either as an area of overlap or a cradle of marine biodiversity, the hypotheses proposed to account for this pattern rely on extant knowledge about taxonomy and species range distribution. The recent large-scale use of standard molecular data (DNA barcoding), however, has revealed the importance of taking into account cryptic diversity when assessing tropical biodiversity. We DNA barcoded 2276 specimens belonging to 668 coral reef fish species through a collaborative effort conducted concomitantly in both Indian and Pacific oceans to appraise the importance of cryptic diversity in species with an Indo-Pacific distribution range. Of the 141 species sampled on each side of the IMPA, 62 presented no spatial structure whereas 67 exhibited divergent lineages on each side of the IMPA with K2P distances ranging between 1% and 12%, and 12 presented several lineages with K2P distances ranging between 3% and 22%. Thus, from this initial pool of 141 nominal species with Indo-Pacific distribution, 79 dissolved into 165 biological units among which 162 were found in a single ocean. This result is consistent with the view that the IMPA accumulates diversity as a consequence of its geological history, its location on the junction between the two main tropical oceans and the presence of a land bridge during glacial times in the IMPA that fostered allopatric divergence and secondary contacts between the Indian and Pacific oceans.

## Introduction

Biodiversity tends to concentrate across the Earth in restricted areas that have been identified as biodiversity hotspots [1], [2]. These areas of exceptional richness are distinguished by numerous endemic species and a high vulnerability to habitat loss. Globally, the modern decline of biodiversity calls for a better understanding of the processes regulating diversity and the consequences of species loss for ecosystem functioning [3]–[8]. In marine systems, coral reefs are among the most diverse habitats on the globe and their biodiversity culminates in the Indo-Malay-Philippines Archipelago (IMPA), declining East and West into the Indian and Pacific oceans [2], [9]–[12]. Although fifty-eight percent of the world's reefs are reported to be threatened by human activities that affect ecosystem services [2], , our understanding of regional-scale patterns of biodiversity on coral reefs is still limited [11], [13]–[17]. This knowledge is, however, of critical importance for guiding conservation policies [12], [18]–[21].

Several hypotheses invoking speciation and dispersal have been proposed to explain large-scale patterns of biodiversity in coral reefs [11]–[15], [22]–[24]. The two major hypotheses, namely the Centre-of-Origin and Centre-of-Overlap, are detailed in Table 1. Globally, the Centre-of-Origin hypothesis proposes the IMPA as a cradle of tropical reef biodiversity where lineages originate and species subsequently migrate into peripheral areas, where they ultimately remain as relicts. The Centre-of-Overlap hypothesis, by contrast, suggests that the higher diversity in the IMPA results from the overlap of faunas from several biogeographic provinces and predicts that only the tails of the species ranges reach the IMPA. If so, most species would have their midpoint range outside the IMPA.

**Table 1:** Summary of the predictions from the Centre-of-Overlap and Centre-of-Origin hypotheses.

Hypothesis	Assumptions	Diversification processes driving species richness in the IMPA	Predictions	References
Centre-of-Origin	the IMPA is a centre of origin where species originate and subsequently disperse in peripheral areas	Species originate in the IMPA through sympatric speciation and/or allopatric speciation among islands of the IMPA	(1) Midpoint ranges of species distribution ranges fall in the IMPA	[11]–[13], [22]
			(2) Large scale genetic structure is expected to be shallow as a consequence of high connectivity and larval dispersal around the IMPA	
			(3) Cryptic species may be expected to occur in sympatry as a consequence of high connectivity	
Centre-of-Overlap	the IMPA is a meeting point of several biogeographic provinces and faunas as a consequence of its location between the Indian and Pacific ocean	Species originate through geographic isolation and allopatric speciation on each side of the IMPA	(1) Midpoint ranges of species distribution ranges fall on each side the IMPA	[14]–[17], [25], [28], [30]–[32],
			(2) Large scale genetic structure is expected to be important as a consequence of geographic isolation	
			(3) Cryptic species may be expected to exhibit allopatric and non-overlapping distribution ranges	

It is a widely held view that the distribution of species richness and endemism follows a unimodal distribution with a peak coinciding with the IMPA and that most widespread species have their midpoint range in this area as predicted by the Centre-of-Origin hypothesis [2], [11], [24]. Recent molecular studies, however, emphasized that widespread coral reef fishes are often complexes of several cryptic species that have more restricted and non-overlapping ranges with midpoints outside the IMPA as predicted by the Centre-of-Overlap hypothesis (Table 1); [25]–[33]. This report highlights the importance of gathering accurate data on taxonomy and distribution when considering testing large-scale biodiversity hypotheses as ignoring cryptic diversity often distorts our perception of biodiversity, sometimes spectacularly in the Tropics [34]–[36]. When considering the task awaiting taxonomists in the Indo-Pacific (*i.e.* 5000 coral reef fish species according to fishbase in 2011), it becomes apparent that high-throughput sequencing of a standard gene region, i.e. DNA barcoding, can move things forward. Inasmuch as fish species exhibit inter-species divergence levels at the cytochrome c oxidase 1 (COI) gene as high as 25 times those found for intra-species divergence levels [37]–[38], cryptic diversity might be consistently detected [39].

**Figure 1:**
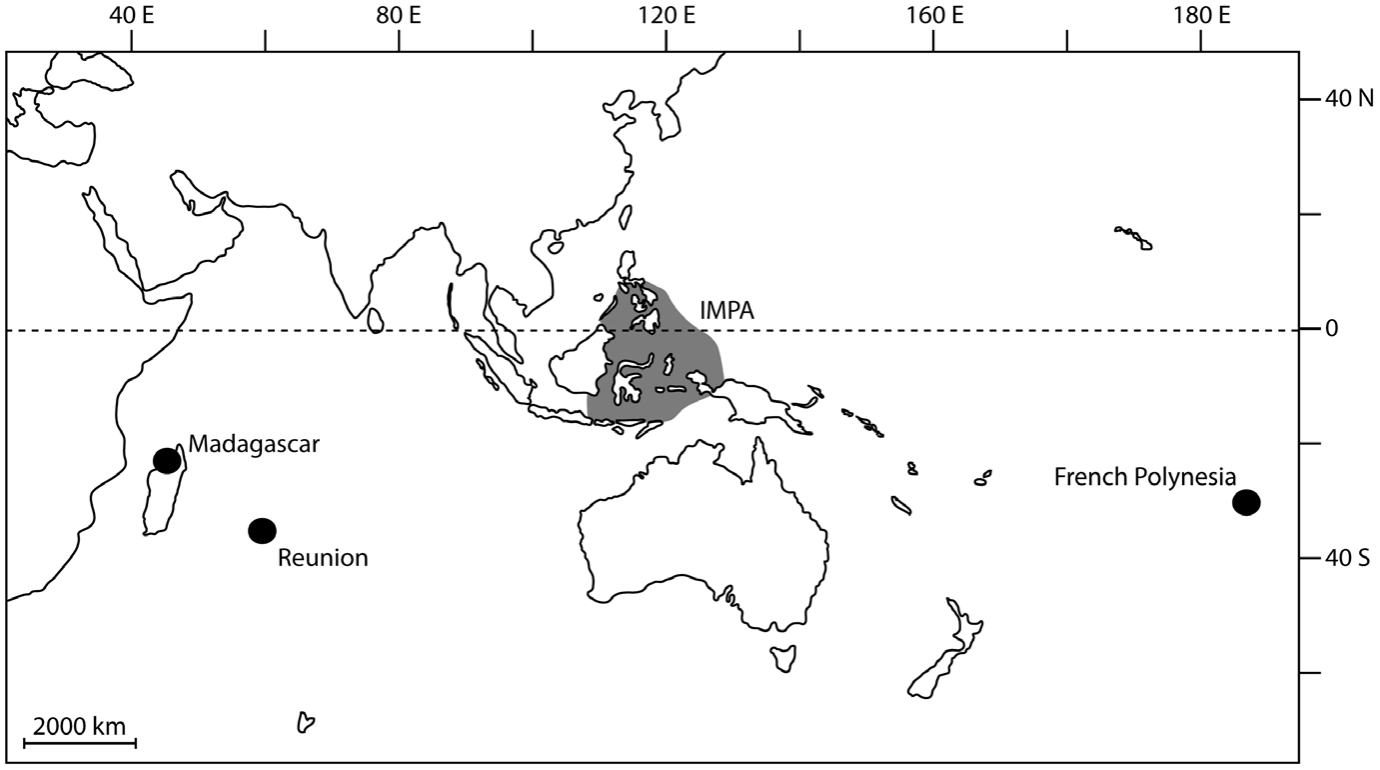
Map of the sampling localities and position of the Indo-Malay-Philippines Archipelago (IMPA) peak of diversity (derived from [**17**]).

To ascertain whether cryptic diversity is a general trend in widely distributed coral-reef fishes in the Indo-Pacific and appraise some of predictions made by the Centre-of-Origin and Centre-of-Overlap hypotheses (Table 1), we DNA-barcoded 2276 specimens from 668 species, all being collected in the framework of the large-scale barcode initiatives currently held in the Pacific (Moorea Biocode project, French Polynesia; Fig. 1) and Indian ocean (BIOTAS project, Réunion island and Madagascar; Fig. 1). Globally, 141 of the 668 species were sampled in both Indian and Pacific oceans and 79 of these widespread species encompassed several evolutionary lineages found in allopatry and supporting unexpectedly high levels of divergence. This finding highlights the overwhelming occurrence of cryptic diversity in taxa exhibiting Indo-Pacific range distribution since 60% of them dissolved in lineages restricted to a single ocean. In this study, the use of DNA barcodes to assess large-scale cryptic discontinuities in species-rich faunas proved to be effective and shed a new light on the origin of biodiversity in Indo-Pacific coral-reef fishes.

## Results

**Figure 2:**
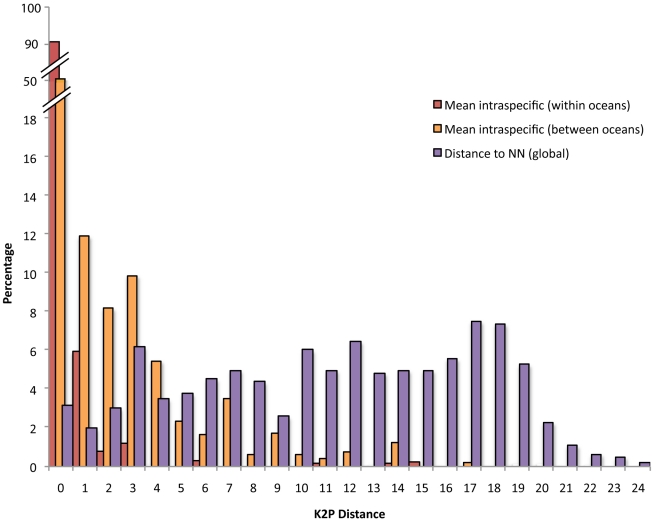
Summary of the distribution of K2P distances to the nearest neighbour and mean intraspecific distance (within and between oceans). NN, nearest neighbour.

A full-length PCR product was recovered for 2276 individuals belonging to 668 species (3.41 individuals per species on average), 265 genera and 79 families (Table S1). No insertions/deletions or codon stops were found as expected in functional sequences and sequences were easily aligned. The entire K2P-NJ tree derived from this study is available as Supplementary Information (Fig. S1). A steady increase of genetic variation through increasing taxonomic levels was observed (Table 2 and S2). Overall, genetic divergence among congeneric species was 15-fold higher on average than among individuals of the same species. Intraspecific divergence showed considerable heterogeneity, however, ranging from 0 to 22.16% with a mean of 1.06%, a value three-fold higher than previously reported for fishes [37]–[39]. COI barcodes failed to capture species boundaries in only three species pairs, namely *Abudefduf sexfasciatus* (4 individuals) and *A. vaigiensis* (2 individuals), *Chaetodon guttatissimus* (6 individuals) and *C. pelewensis* (2 individuals), *Chaetodon kleinii* (6 individuals) and *C. trichrous* (3 individuals). We inspected the distribution of the intraspecific variability between the Indian and Pacific oceans and found that most of the heterogeneity in the distribution of the intraspecific distances was due to the effect of geographic isolation between oceans (Fig. 2). Furthermore, 50% of the mean intraspecific K2P distances between oceans were even higher than the distances observed between the most closely related species in the entire data set (Fig. 2, Table 2 and S3).

**Table 2:** Summary of K2P distances for increasing taxonomic levels. Data are from 2276 sequences from 668 species and 265 genera (*SI* Appendix S2). Comparisons within species based on the 514 species with *N*>1.

Comparisons within	Taxa	Number of individuals (*N*)	Comparisons	Min	Mean	Max	SE
Species	514	2122	4784	0	1.06	22.16	0.03
Genus, among species	217	2228	28849	0.15	15.34	31.57	0.03
Family, among genera	70	2267	112978	1.87	20.55	33.10	0.01
Order, among families	14	2270	1341805	5.42	23.20	34.54	<0.01
Class, among orders	2	2276	1080131	14.62	24.110	39.15	<0.01

Globally, 141 out of the 668 species sampled were found in both Indian and Pacific oceans and the distribution of their COI barcodes fell into four patterns. Fig. S2 illustrates them while Table S3 enumerates the species belonging to each pattern.

### Pattern 1: A barcoded species with Indo-Pacific range remains a widely distributed species (shallow spatial structure)

**Figure 3:**
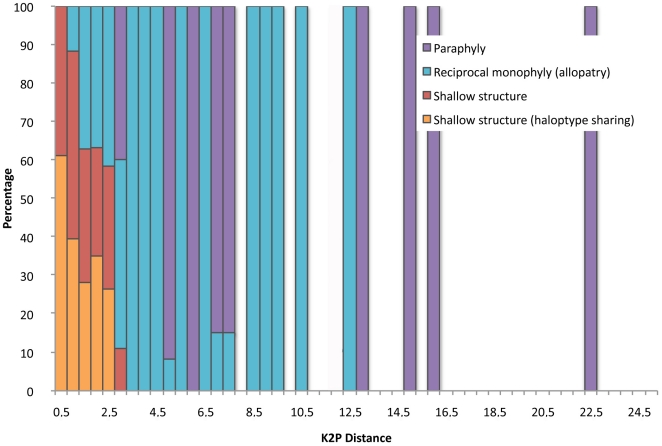
Distribution of the relative frequencies of COI barcodes patterns according to the maximum intra-specific K2P distance.

Globally, 62 of the 141 widespread species exhibited shallow spatial structure with mixed COI lineages between Indian and Pacific oceans (*i.e.* 43%) and 19 of the 62 species exhibited haplotype sharing and direct evidence of large-scale dispersal and gene flow. Most of the species falling into this pattern exhibited maximum K2P distances ranging between 0.1 and 1% with a few remarkable exceptions in *Bodianus axillaris* (8 individuals), *Epibulus insidiator* (7 individuals), *Gymnothorax javanicus* (7 individuals) and *G. buroensis* (11 individuals), *Ostracion cubicus* (7 individuals), *Plagiotremus tapeinosoma* (5 individuals) and *Sargocentron spiniferum* (6 individuals) and *S. punctatissimum* (10 individuals) where maximum K2P distances reached up to 2.8% (Fig. 3, Fig. S2, Table S3). Only species with shallow structure and no haplotype sharing, however, reached maximum K2P of 3% (Fig. 3).

### Pattern 2: A barcoded species with Indo-Pacific range includes two lineages each restricted to a single ocean (geographic monophyly)

This pattern was found to be the most frequent as 67 of the 141 widespread species achieved reciprocal geographic monophyly and exhibited distinct lineages in the Indian and Pacific oceans with maximum K2P distances ranging from 1.0% in *Dascyllus aruanus* (11 individuals) up to 12.1% in *Caracanthus unipinna* (3 individuals) (Fig. 3, Fig. S2, Table S3). Within each geographic lineage from the Indian and Pacific oceans, the maximum K2P distance attained 0.3%, close to levels, previously reported for fishes [37]–[39]. The commonness of this pattern supports a marked influence of geographic isolation in species with Indo-Pacific distribution ranges.

### Pattern 3: A barcoded species with Indo-Pacific range includes several lineages each restricted to a single ocean (geographic paraphyly)

At least six species exhibited complex patterns of cryptic diversity encompassing more than two lineages distributed in the Indian or Pacific oceans, sometimes both, but separated by surprisingly high K2P distances.

- ***Dinematichthys iluocoeteoides.*** This ovoviviparous species was initially described as widely distributed in the Indo-Pacific, but is currently thought to occur in: Red Sea, Indo-West Pacific: East Africa, Seychelles, Comores and Mascarenes east to Samoa and Tonga, north to Kagoshima Islands and Ogasawara islands and Taiwan, south to Queensland (Australia) and New Caledonia [40]. Among the eight individuals analyzed, three lineages were detected in the Southwestern Indian Ocean diverging by up to 15% and nested with another lineage from the Pacific (Fig. S1 and S2, Table S3). Divergence levels are consistent with the existence of at least four biological units among which three occur in sympatry within the Indian Ocean.

- ***Halichoeres hortulanus.*** DNA barcodes support the presence of two lineages in the Indian Ocean, one in Madagascar and one in the Mascarenes (Réunion), diverging by 5.9% (8 individuals; Fig. S1 and S2, Table S3). Likewise, a third distinct lineage was detected in the Pacific that turned to be more closely related to the Madasgacar group than the Mascarene group with a maximum K2P distance of 5.2%. Some geographic variation has been cited between Indian and Pacific Ocean populations that correlate with the present pattern [41].

- ***Kaupichthys diodontus.*** This species is considered to occur widely in the Indo-Pacific and some authors proposed to place it in synonymy with the Atlantic *K. hyoproroides* becoming a circum-tropical species [41]. The seven COI barcodes for *K. diodontus* support the presence of four lineages diverging by at least 12% and up to 22% (Fig. S1 and S2, Table S3). Among them, one has been sampled only in the Pacific, two others in the Indian Ocean and the fourth is potentially widely distributed in the Indian and Pacific oceans.

- ***Pseudogramma polyacantha.*** This species is widely reported in both Indian and Pacific Oceans [41]. The seven COI barcodes supported the presence of a distinct lineage at Réunion separated by 6.6%, while the Madagascar lineage seems widely distributed in the Indo-Pacific (Fig. S1, Table S3).

- ***Sebastapistes tinkhami.*** The four COI barcodes support the presence of three lineages diverging by up to 13.2% (Fig. S1, Table S3). Two very divergent lineages are found in French Polynesia, one of them being more closely related to a third lineage restricted to the Indian Ocean and found only at Réunion.

- ***Synodus dermatogenys.*** This species is generally abundant and also widely reported in the Indo-Pacific. The four COI barcodes support the presence of a distinct lineage in French Polynesia diverging by 3.3% from a second lineage with Indo-Pacific range (Fig. S1, Table S3).

### Pattern 4: A barcoded species with Indo-Pacific range includes several lineages nested with different species (taxonomic paraphyly or polyphyly)

In six species, COI barcodes supported the presence of several lineages, often restricted to a single ocean, but encompassing the COI cluster of a closely related, yet distinct, species from the same genus. The present pattern is very similar to pattern 3 but further involves interspecific relationships with sister-species often found in allopatry (Fig. S2).

- ***Labroides dimidiatus.*** The nine COI barcodes support the presence of two lineages diverging by 15%, each restricted to a single ocean but the Pacific lineage appears to be more closely related to *L. bicolor* (Fig. S1 and S2, Table S3). This result is consistent with previous observations [29] that geographic color variations correlate with genetic divergence. Present results, however, further involve *L. bicolor* in this complex of species.

- ***Myripristis berndt.*** The 15 COI barcodes detect one lineage in Madagascar and one lineage in the Pacific that diverge by 7.5% and sort in a larger clade including *M. adusta*, *M. violacea* and *M. amaena* from the Pacific Ocean and *M. hexagona* from the Indian ocean. The Madagascar cluster sort in a basal position while the Pacific cluster was found to be sister-clade to *M. amaena* and *M. hexagona* (Fig. S1 and S2, Table S3).

- ***Plectroglyphidodon lacrymatus.*** The nine COI barcodes support the presence of a lineage restricted to Madagascar and a second restricted to French Polynesia, both diverging by 5.9% (Fig. S1 and S2, Table S3). The Pacific lineage, however, is more closely related to *Stegastes pelicieri* from the Indian Ocean from which it diverges by 5%. Worth mentioning, previous phylogenetic studies emphasized that *Stegastes* is not monophyletic as several species including *S. fasciolatus* and *S. pelicieri* are more closely related to species from the genus *Plectroglyphidodon* than other congenerics [42].

**-** ***Pterois antennata.*** The six COI barcodes segregated into three lineages diverging by up to 7.1% (Fig. S1 and S2, Table S3). One lineage is found only in French Polynesia as a sister-clade of a second lineage only found in the Indian Ocean, both being the sister-clade of *Pterois radiata* in the Pacific (Fig. S2). Then, a third lineage was found in both Indian and Pacific Oceans.

- ***Sargocentron diadema.*** The six COI barcodes segregate into two distinct lineages, each restricted to a single locality and diverging by 4.7% (Fig. S1 and S2). The Barcode cluster found in French Polynesia, however, is more closely related to *Sargocentron microstoma* in the Pacific than its conspecifics from the Indian Ocean.

- ***Zebrasoma scopas.*** The nine COI barcodes segregate into two lineages, each restricted to a single ocean and diverging by 4.9% (Fig. S1, Table S3). The Pacific lineage, however, is more closely related to *Zebrasoma rostratum* from French Polynesia than to conspecifics from the Indian Ocean (Fig. S1).

## Discussion

COI DNA barcoding has shown that among the 141 species thought to have Indo-Pacific range distribution, only 62 failed to provide evidence of cryptic diversity throughout their range, whereas 79 dissolved into 165 biological units that were all readily identified by their barcodes and among which 162 were found to be restricted to a single ocean. Only three species pairs among the 668 species examined here (*i.e.* 0.008%) exhibit complex boundaries that COI barcodes failed to capture. Globally, COI barcoding is not only effective for the identification of species, but, more importantly, it proved to be highly effective for the discovery of provisional cryptic diversity awaiting an integrative screening as previously highlighted [34]–[36]. Actually, several cryptic lineages detected here correlate with geographic variation in color patterns as in *Labroides bicolor* [29] or *Halichoeres hortulanus* [41] and further support the evidence for extensive cryptic diversity in species currently thought to have a widespread Indo-Pacific range distribution.

Globally, the high number of copies, higher rate of genetic drift and mutation, and ease of sequencing make COI barcoding an attractive approach for molecular identification [43]. Yet, its maternal inheritance limits its power when applying to young species sharing polymorphism as a consequence of recent ancestry or species that can hybridize, since species delineation and identification may not be possible if prior information is not available [44]. Cases of shared polymorphism due to recent ancestry were found in the species pairs with mixed COI barcode clusters as *Abudefduf sexfasciatus* and *A. vaigiensis*, *Chaetodon guttatissimus* and *C. pelewensis*, *Chaetodon kleinii* and *C. trichrous*. However, all of these species pairs exhibit marked differences in color or morphology [41]. We cannot rule out that most species with Indo-Pacific range distribution for which we failed to detect distinct Indian and Pacific lineages, may suffer from the lack of resolution inherent in ancestral polymorphism. Indeed, direct evidence of gene flow such as the sharing of similar COI barcodes were found in only 19 of the 62 species with shallow spatial structure (Fig. 3, Fig. S2, Table S3). This report further advocates that the very high levels of cryptic diversity detected here may actually be underestimated as the more recently isolated lineages are likely to be undetected through the use of COI barcoding. These limits are unlikely to lead to an overestimation of cryptic diversity, as among the 165 provisional cryptic biological units detected here, 162 exhibit restricted geographic ranges consistent with an evolution of COI barcode diversity in these lineages through geographic isolation between the Indian and Pacific oceans.

Recent large-scale reviews about distribution of species richness in Indo-Pacific coral-reef fishes argue for a unimodal distribution with a peak coinciding with the IMPA [11]. This pattern has been recently interpreted as a consequence of intense diversification in the IMPA producing species that further disperse and colonize peripheral islands from the Indian and Pacific Oceans [22]. According to this model, the IMPA is a centre-of-origin that supplies new species in peripheral areas through long distance dispersal more frequently than speciation in the periphery of the IMPA. As a corollary, the extent of a species range is a function of its age and the gradient of diversity around the IMPA is a consequence of the continuous production of species in the centre that expanded their range through the Indian and Pacific oceans according to their age (*i.e.* wider range in older species). As a consequence, the centre-of-origin hypothesis predicts that species midpoint ranges occur in the IMPA, concomitantly to the peak of diversity as a consequence of higher diversification rates here than elsewhere through geological times. Keystone of the centre-of-origin hypothesis, the location of the midpoint range for the species with Indo-Pacific distribution is expected to fit the location of the centre of origin, thus the IMPA [11].

The detection of extensive cryptic diversity through large-scale COI barcoding questions the origin of the IMPA peak of diversity through an increased rate of diversification in the area. The patterns of cryptic diversity did not corroborate the prediction that range midpoints of species with Indo-Pacific distribution meet in the IMPA. Globally, 60% of the species with Indo-Pacific range distribution dissolved in biological units restricted to a single ocean, hence, having midpoint ranges on either side of the IMPA. By contrast, this result is consistent with large faunal discontinuities between the Indian and Pacific Oceans ichthyofaunas as a consequence of geographic isolation on each side of the IMPA as discussed by [13]. The Centre of Overlap hypothesis is in agreement with the predictions associated with the sea level fluctuations that accompanied the Milankovitch cycles. According to this scenario, sea level drops during glacial times led to the emergence of the Sunda shelf in the IMPA causing local extinctions due to lowered salinities and lower temperatures in the Indo-Malay region and erecting physical barriers to dispersal between the Indian and Pacific oceans, thereby fostering allopatric speciation [12], [13], [42], [45]. Despite some local extinctions in the Indo-Malay region, an increase in the diversification rates in the Celebes-to-Banda Sea and Philippine regions may be expected as a consequence of sea level fluctuations during glacial periods [12]. The Philippines to Banda Sea portion of the IMPA is a mosaic of deep seas (*i.e.* South China, Sulu, Celebes, Molucca and Banda seas) delimited by orogenic arches and providing opportunities for the maintenance of highly fragmented coral-reef communities in the area during periods of low sea levels [46]–[47]. In this context, higher diversification rates in the IMPA may be the consequence of multiple episodes of geographic isolation and barriers to gene flow. According to this hypothesis, the IMPA may be expected to exhibit more young endemic species than elsewhere as a consequence of intensive diversification and cladogenesis. A recent review of endemic species age in the area, however, argues that endemic species from the IMPA are not younger than elsewhere [17].

The patterns of cryptic diversity detected here are also in contradiction with the role of long distance dispersal as the main driver of species range expansion throughout the Indian and Pacific Oceans as predicted by the centre-of-origin hypothesis. By contrast, the detection of divergent lineages geographically restricted within the range of species with Indo-Pacific distribution argues that dispersal takes place at much smaller scales than those involved in the centre-of-origin hypothesis. This view is more consistent with an accumulation of diversity in the IMPA as a consequence of its geographic location at the junction of the two main tropical marine domains on earth. In addition, the present study suggests that the species richness in the IMPA may be still underestimated providing that the tail of the range distribution of the cryptic lineages detected here reach the IMPA. According to a scenario of allopatric speciation between the Indian and Pacific Oceans as a consequence of sea level drops, geographic breaks between sister-species may be expected to occur in the IMPA [13]. A growing number of examples derived from recent molecular studies corroborate this prediction [25], [28], [30]–[32], [48].

Globally, this study is the first large scale molecular study of cryptic diversity in coral reef fishes and it provides new evidence supporting a strong influence of geographic isolation on speciation, a result consistent with the IMPA (particularly the Philippines where distributional ranges overlap extensively) playing the role of an area of overlap rather than a cradle of marine biodiversity. The present study emphasises the importance of accounting for cryptic diversity when considering patterns of biodiversity in tropical ecosystems, a notion repeatedly emphasized and emanating from all large-scale taxonomic surveys of complex tropical animal communities through DNA barcoding conducted during the last decade [34]–[36].

## Materials and Methods

Several large-scale DNA-barcode initiatives have been initiated since 2006 on coral reefs. In the Pacific, the Moorea Biocode project is attempting to create the first comprehensive inventory of all non-microbial life in a complex tropical ecosystem (http://mooreabiocode.org/). Similarly, the BIOTAS project is attempting to inventory biodiversity in terrestrial and marine biotas from the Southwest Indian Ocean through large-scale barcoding and phylogenetic approaches (http://umr-pvbmt.cirad.fr/content/view/full/2068). Several campaigns have been conducted for marine taxa between March 2006 and May 2008 in Réunion, Madagascar and French Polynesia and several thousand of coral-reef fish specimens have been collected. The present survey originated in the context of a collaborative effort among these programs with the goal of conducting the first global assessment of cryptic diversity in Indo-Pacific coral-reef fishes.

All specimens were sampled between March 2006 and May 2008 in sites randomly picked across inner reefs and outer slopes in Madagascar, Réunion and French Polynesia at depths ranging from 0 to about 40 meters (Fig. 1, Fig. S1; Collection permits 630/AM/07 and 91/AM/08, “Affaires Maritimes”, Réunion; Collection permit 248/08-MAEP/SG/DGAEP/DPRH/SPP, “Direction de la pêche et des ressources halieutiques”, Madagascar; Permanent agreement, “Délégation à la Recherche”, French Polynesia). Specimens were caught using powdered derris root (containing about 7–8% rotenone) applied in quadrats each measuring about 20×20 m. DNA-source for this study included ethanol preserved tissues (muscles, fins). For each specimen, detailed geographic information and reference specimens were deposited as vouchers in publicly available collections (‘Muséum National d'Histoire Naturelle’ in Paris; National Museum of Natural History, Smithsonian Institution, Washington (DC); University of Réunion; University of California, Berkeley). Identifications were done by several of the authors based on morphological criteria (color, meristic counts) currently recognized in recent monographs and available data about species range distribution [41], [49]–[51] and FAO Identification Field Guides.

Genomic DNA was extracted using the QIAGEN DNeasy 96 Blood and Tissue Kit according to manufacturer specifications and further used with no dilution for amplification and sequencing. A 650-bp segment from the 5′ region of the cytochrome oxidase I gene (COI) was amplified using the primers FishBCL-5′TCAACYAATCAYAAAGATATYGGCAC3′ and FishBCH-5′ACTTCYGGGTGRCCRAARAATCA3′ [52]. PCR amplifications were performed in 10 µl including 6.3 µl of molecular grade water, 1.0 µl of 10× PCR buffer, 0.5 µl of MgCl_2_ (50 mM), 0.3 µl of each primer (10 mM), 0.5 µl of dNTPs (10 mM), 0.1 µl of Bioline Taq polymerase, and 1 µl of template DNA. The PCR conditions consisted of 95°C (5 min), 35 cycles of 95°C (30 sec), 52°C (30 sec), 72°C (45 sec) followed by a final extension at 72°C (5 min). PCR products were purified using the ExoSAP-IT protocol (Amersham Biosciences, Piscataway NY) and used as template for sequencing reaction using BigDye Terminator (version 3.1) Cycle Sequencing Kit (Applied Biosystems, Inc., Foster City, Calif.) Sequencing products were cleaned before running on the capillary sequencer using a Sephadex centrifugation protocol (Millipore MAHVN4550 plates).

All the sequences have been deposited in GenBank and accession numbers for the barcodes, specimen and collection data, sequences, trace files and primers details are available within the MBFC, FPFL, IPCOM, MBFA and SBF project files under the general container ‘Moorea Biocode – Fish’ in BOLD (http://www.barcodinglife.org; Fig. S1). Sequence divergence was calculated using the Kimura 2-parameter (K2P) model and the mid-point rooted Neighbour-joining (NJ) tree of K2P distances was created to provide a graphic representation of the species divergence as implemented in the ‘Sequence Analysis’ module of BOLD (Fig. S1).

## Supporting Information

Table S1 **Details of species and specimens. Barcode of Life Database (BOLD) specimen numbers given, along with GenBank accession numbers, geographic locality and voucher details.** Taxonomy and nomenclature following BOLD and fishbase.
(DOCX)

Table S2 **Summary of K2P distances to the nearest-neighbour for the 668 species.**
(DOCX)

Table S3 **List of species with Indo-Pacific range distribution belonging to each of the four pattern of COI barcodes distribution.**
(DOCX)

Figure S1 **Neighbour-joining tree of 2276 COI barcodes belonging to the 668 coral reef fish species examined here.**
(PDF)

Figure S2 **Description of the four pattern of COI barcodes distribution in species with Indo-Pacific range distribution.**
(TIF)
