# Supplementary material for: Cryptic Diversity in Indo-Pacific Coral-Reef Fishes Revealed by DNA-Barcoding Provides New Support to the Centre-of-Overlap Hypothesis
Source: PLoS One. 2012 Mar 15;7(3):e28987. doi: 10.1371/journal.pone.0028987 (PMC3305298; doi:10.1371/journal.pone.0028987)
Supplement: Table S1 — Details of species and specimens. Barcode of Life Database (BOLD) specimen numbers given, along with GenBank accession numbers, geographic locality and voucher details. Taxonomy and nomenclature following BOLD and fishbase. (DOCX) [file pone.0028987.s001.docx]

| **Family** | **Species** | **Geographic locality** | **Voucher** | **BOLD** | **GenBank** |
| --- | --- | --- | --- | --- | --- |
| Acanthuridae | *Acanthurus achilles* | French Polynesia:Society Islands:Moorea | MNHN:Ich:BIOCODES811 | MBIO1277.4 | HM034179 |
| Acanthuridae | *Acanthurus achilles* | French Polynesia:Society Islands:Moorea | MNHN:Ich:BIOCODES812 | MBIO1278.4 | HM034178 |
| Acanthuridae | *Acanthurus achilles* | French Polynesia:Marquesas Islands:Mohotane | CRIOBE:Ich:MBIO18242 | MBIO18242 | HM034177 |
| Acanthuridae | *Acanthurus blochii* | French Polynesia:Society Islands:Moorea | MNHN:Ich:BIOCODES749 | MBIO1159.4 | HM034180 |
| Acanthuridae | *Acanthurus dussumieri* | Madagascar:West:Nosy Be | ECOMAR:Ich:NBE0655 | NBE0655 | JQ349649 |
| Acanthuridae | *Acanthurus guttatus* | French Polynesia:Marquesas Islands:Mohotane | CRIOBE:Ich:MBIO18213 | MBIO18213 | HM034184 |
| Acanthuridae | *Acanthurus guttatus* | French Polynesia:Society Islands:Moorea | MNHN:Ich:BIOCODES505 | MBIO741.4 | HM034181 |
| Acanthuridae | *Acanthurus guttatus* | French Polynesia:Society Islands:Tetiaroa | MNHN:Ich:2009-1628 | MOCNESS M9.1-M0-2 | HM034137 |
| Acanthuridae | *Acanthurus guttatus* | Reunion:South:St Philippe | ECOMAR:Ich:REU1751 | REU1751 | JQ349650 |
| Acanthuridae | *Acanthurus lineatus* | French Polynesia:Society Islands:Moorea | MNHN:Ich:BIOCODES620 | MBIO918.4 | HM034183 |
| Acanthuridae | *Acanthurus lineatus* | French Polynesia:Society Islands:Moorea | SI:Ich:MBIO919 | MBIO919.4 | HM034182 |
| Acanthuridae | *Acanthurus lineatus* | Madagascar:West:Nosy Be | ECOMAR:Ich:NBE0009 | NBE0009 | JQ349651 |
| Acanthuridae | *Acanthurus lineatus* | Madagascar:West:Nosy Be | ECOMAR:Ich:NBE0668 | NBE0668 | JQ349652 |
| Acanthuridae | *Acanthurus nigricans* | French Polynesia:Society Islands:Moorea | MNHN:Ich:BIOCODES0087 | MBIO136.4 | HM034186 |
| Acanthuridae | *Acanthurus nigricans* | French Polynesia:Society Islands:Moorea | MNHN:Ich:BIOCODES0088 | MBIO137.4 | HM034185 |
| Acanthuridae | *Acanthurus nigricauda* | French Polynesia:Society Islands:Moorea | MNHN:Ich:BIOCODES745 | MBIO1154.4 | HM034187 |
| Acanthuridae | *Acanthurus nigricauda* | French Polynesia:Society Islands:Moorea | MNHN:Ich:BIOCODES0304 | MBIO441.4 | HM034189 |
| Acanthuridae | *Acanthurus nigricauda* | French Polynesia:Society Islands:Moorea | MNHN:Ich:BIOCODES621 | MBIO917.4 | HM034188 |
| Acanthuridae | *Acanthurus nigricauda* | Reunion:West:La Saline | ECOMAR:Ich:REU0297 | REU0297 | JQ349654 |
| Acanthuridae | *Acanthurus nigricauda* | Reunion:West:St Gilles | ECOMAR:Ich:REU155-1 | REU155_1 | JQ349655 |
| Acanthuridae | *Acanthurus nigricauda* | Reunion:West:La Saline | ECOMAR:Ich:REU272-1 | REU272_1 | JQ349653 |
| Acanthuridae | *Acanthurus nigrofuscus* | French Polynesia:Society Islands:Moorea | MNHN:Ich:BIOCODES0079 | MBIO126.4 | HM034190 |
| Acanthuridae | *Acanthurus nigrofuscus* | French Polynesia:Society Islands:Moorea | MNHN:Ich:BIOCODES0017 | MBIO41.4 | HM034192 |
| Acanthuridae | *Acanthurus nigrofuscus* | French Polynesia:Society Islands:Moorea | MNHN:Ich:BIOCODES0018 | MBIO42.4 | HM034191 |
| Acanthuridae | *Acanthurus nigrofuscus* | French Polynesia:Society Islands:Tetiaroa | MNHN:Ich:2009-1629 | MOCNESS M9.1-M0-1 | HM034138 |
| Acanthuridae | *Acanthurus nigrofuscus* | French Polynesia:Society Islands:Tetiaroa | MNHN:Ich:2009-1634 | MOCNESS M9.1-M0-11 | HM034143 |
| Acanthuridae | *Acanthurus nigrofuscus* | French Polynesia:Society Islands:Tetiaroa | MNHN:Ich:2009-1635 | MOCNESS M9.1-M0-12 | HM034144 |
| Acanthuridae | *Acanthurus nigrofuscus* | French Polynesia:Society Islands:Tetiaroa | MNHN:Ich:2009-1636 | MOCNESS M9.1-M0-13 | HM034145 |
| Acanthuridae | *Acanthurus nigrofuscus* | French Polynesia:Society Islands:Tetiaroa | MNHN:Ich:2009-1630 | MOCNESS M9.1-M0-3 | HM034139 |
| Acanthuridae | *Acanthurus nigrofuscus* | French Polynesia:Society Islands:Tetiaroa | MNHN:Ich:2009-1631 | MOCNESS M9.1-M0-4 | HM034140 |
| Acanthuridae | *Acanthurus nigrofuscus* | French Polynesia:Society Islands:Tetiaroa | MNHN:Ich:2009-1632 | MOCNESS M9.1-M0-6 | HM034141 |
| Acanthuridae | *Acanthurus nigrofuscus* | French Polynesia:Society Islands:Tetiaroa | MNHN:Ich:2009-1633 | MOCNESS M9.1-M0-7 | HM034142 |
| Acanthuridae | *Acanthurus nigrofuscus* | Madagascar:West:Nosy Be | ECOMAR:Ich:NBE1252 | NBE1252 | JQ349657 |
| Acanthuridae | *Acanthurus nigrofuscus* | Reunion:North:La possession | ECOMAR:Ich:REU0145 | REU0145 | JQ349658 |
| Acanthuridae | *Acanthurus nigrofuscus* | Reunion:West:St Leu | ECOMAR:Ich:REU1707 | REU1707 | JQ349659 |
| Acanthuridae | *Acanthurus nigrofuscus* | Reunion:West:St Leu | ECOMAR:Ich:REU2646 | REU2646 | JQ349656 |
| Acanthuridae | *Acanthurus nubilus* | French Polynesia:Society Islands:Moorea | MNHN:Ich:BIOCODES643 | MBIO953.4 | HM034193 |
| Acanthuridae | *Acanthurus olivaceus* | French Polynesia:Society Islands:Moorea | SI:Ich:MBIO1233 | MBIO1233.4 | HM034197 |
| Acanthuridae | *Acanthurus olivaceus* | French Polynesia:Marquesas Islands:Mohotane | CRIOBE:Ich:MBIO18257 | MBIO18257 | HM034196 |
| Acanthuridae | *Acanthurus olivaceus* | French Polynesia:Marquesas Islands:Mohotane | CRIOBE:Ich:MBIO18261 | MBIO18261 | HM034195 |
| Acanthuridae | *Acanthurus olivaceus* | French Polynesia:Marquesas Islands:Mohotane | CRIOBE:Ich:MBIO18328 | MBIO18328 | HM034194 |
| Acanthuridae | *Acanthurus olivaceus* | French Polynesia:Society Islands:Moorea | MNHN:Ich:BIOCODES619 | MBIO920.4 | HM034198 |
| Acanthuridae | *Acanthurus polyzona* | Reunion:South:St Philippe | ECOMAR:Ich:REU1690 | REU1690 | JQ349660 |
| Acanthuridae | *Acanthurus polyzona* | Reunion:South:St Philippe | ECOMAR:Ich:REU1748 | REU1748 | JQ349664 |
| Acanthuridae | *Acanthurus polyzona* | Reunion:South:St Philippe | ECOMAR:Ich:REU1810 | REU1810 | JQ349661 |
| Acanthuridae | *Acanthurus polyzona* | Reunion:South:St Philippe | ECOMAR:Ich:REU1811 | REU1811 | JQ349663 |
| Acanthuridae | *Acanthurus polyzona* | Reunion:South:St Philippe | ECOMAR:Ich:REU1812 | REU1812 | JQ349662 |
| Acanthuridae | *Acanthurus pyroferus* | French Polynesia:Society Islands:Moorea | SI:Ich:MBIO1316 | MBIO1316.4 | HM034200 |
| Acanthuridae | *Acanthurus pyroferus* | French Polynesia:Marquesas Islands:Mohotane | CRIOBE:Ich:MBIO18263 | MBIO18263 | HM034199 |
| Acanthuridae | *Acanthurus pyroferus* | French Polynesia:Society Islands:Moorea | MNHN:Ich:BIOCODES616 | MBIO909.4 | HM034202 |
| Acanthuridae | *Acanthurus pyroferus* | French Polynesia:Society Islands:Moorea | MNHN:Ich:BIOCODES615 | MBIO910.4 | HM034201 |
| Acanthuridae | *Acanthurus tennentii* | Madagascar:West:Nosy Be | ECOMAR:Ich:NBE1096 | NBE1096 | JQ349665 |
| Acanthuridae | *Acanthurus tennentii* | Reunion:West:St Gilles | ECOMAR:Ich:REU184-1 | REU184_1 | JQ349666 |
| Acanthuridae | *Acanthurus thompsoni* | French Polynesia:Society Islands:Moorea | SI:Ich:MBIO1620 | MBIO1620.4 | HM034203 |
| Acanthuridae | *Acanthurus thompsoni* | French Polynesia:Society Islands:Moorea | MNHN:Ich:BIOCODES0011 | MBIO33.4 | HM034205 |
| Acanthuridae | *Acanthurus thompsoni* | French Polynesia:Society Islands:Moorea | MNHN:Ich:BIOCODES666 | MBIO987.4 | HM034204 |
| Acanthuridae | *Acanthurus triostegus* | French Polynesia:Society Islands:Moorea | MNHN:Ich:BIOCODES632 | MBIO934.4 | HM034207 |
| Acanthuridae | *Acanthurus triostegus* | French Polynesia:Society Islands:Moorea | SI:Ich:MBIO935 | MBIO935.4 | HM034206 |
| Acanthuridae | *Acanthurus triostegus* | French Polynesia:Society Islands:Tetiaroa | MNHN:Ich:2009-1637 | MOCNESS M10.1-M0-37 | HM034146 |
| Acanthuridae | *Acanthurus triostegus* | French Polynesia:Society Islands:Tetiaroa | MNHN:Ich:2009-1638 | MOCNESS M10.1-M0-38 | HM034147 |
| Acanthuridae | *Acanthurus triostegus* | French Polynesia:Society Islands:Tetiaroa | MNHN:Ich:2009-1639 | MOCNESS M10.1-M0-39 | HM034148 |
| Acanthuridae | *Acanthurus triostegus* | French Polynesia:Society Islands:Tetiaroa | MNHN:Ich:2009-1643 | MOCNESS M9.1-M0-10 | HM034152 |
| Acanthuridae | *Acanthurus triostegus* | French Polynesia:Society Islands:Tetiaroa | MNHN:Ich:2009-1644 | MOCNESS M9.1-M0-14 | HM034153 |
| Acanthuridae | *Acanthurus triostegus* | French Polynesia:Society Islands:Tetiaroa | MNHN:Ich:2009-1640 | MOCNESS M9.1-M0-5 | HM034149 |
| Acanthuridae | *Acanthurus triostegus* | French Polynesia:Society Islands:Tetiaroa | MNHN:Ich:2009-1641 | MOCNESS M9.1-M0-8 | HM034150 |
| Acanthuridae | *Acanthurus triostegus* | French Polynesia:Society Islands:Tetiaroa | MNHN:Ich:2009-1642 | MOCNESS M9.1-M0-9 | HM034151 |
| Acanthuridae | *Acanthurus triostegus* | Madagascar:West:Nosy Be | ECOMAR:Ich:NBE0014 | NBE0014 | JQ349668 |
| Acanthuridae | *Acanthurus triostegus* | Madagascar:West:Nosy Be | ECOMAR:Ich:NBE0658 | NBE0658 | JQ349669 |
| Acanthuridae | *Acanthurus triostegus* | Reunion:West:St Gilles | ECOMAR:Ich:REU008-1 | REU008_1 | JQ349667 |
| Acanthuridae | *Acanthurus triostegus* | Reunion:West:St Gilles | ECOMAR:Ich:REU008-2 | REU008_2 | JQ349670 |
| Acanthuridae | *Acanthurus xanthopterus* | French Polynesia:Society Islands:Moorea | MNHN:Ich:BIOCODES750 | MBIO1153.4 | HM034208 |
| Acanthuridae | *Acanthurus xanthopterus* | Madagascar:West:Nosy Be | ECOMAR:Ich:NBE1064 | NBE1064 | JQ349671 |
| Acanthuridae | *Ctenochaetus binotatus* | Reunion:West:St Leu | ECOMAR:Ich:REU1705 | REU1705 | JQ349920 |
| Acanthuridae | *Ctenochaetus binotatus* | Reunion:West:St Leu | ECOMAR:Ich:REU1706 | REU1706 | JQ349919 |
| Acanthuridae | *Ctenochaetus flavicauda* | French Polynesia:Society Islands:Moorea | SI:Ich:MBIO1406 | MBIO1406.4 | HM034210 |
| Acanthuridae | *Ctenochaetus flavicauda* | French Polynesia:Marquesas Islands:Mohotane | CRIOBE:Ich:MBIO18256 | MBIO18256 | HM034209 |
| Acanthuridae | *Ctenochaetus flavicauda* | French Polynesia:Society Islands:Moorea | MNHN:Ich:BIOCODES665 | MBIO982.4 | HM034212 |
| Acanthuridae | *Ctenochaetus flavicauda* | French Polynesia:Society Islands:Moorea | MNHN:Ich:BIOCODES660 | MBIO983.4 | HM034211 |
| Acanthuridae | *Ctenochaetus flavicauda* | Madagascar:West:Nosy Be | ECOMAR:Ich:NBE0392 | NBE0392 | JQ349922 |
| Acanthuridae | *Ctenochaetus flavicauda* | Reunion:West:St Gilles | ECOMAR:Ich:REU0763 | REU0763 | JQ349921 |
| Acanthuridae | *Ctenochaetus striatus* | French Polynesia:Society Islands:Moorea | MNHN:Ich:BIOCODES0090 | MBIO140.4 | HM034214 |
| Acanthuridae | *Ctenochaetus striatus* | French Polynesia:Society Islands:Moorea | MNHN:Ich:BIOCODES0091 | MBIO141.4 | HM034213 |
| Acanthuridae | *Ctenochaetus striatus* | Madagascar:West:Nosy Be | ECOMAR:Ich:NBE0011 | NBE0011 | JQ349926 |
| Acanthuridae | *Ctenochaetus striatus* | Reunion:West:St Gilles | ECOMAR:Ich:REU064-1 | REU064_1 | JQ349923 |
| Acanthuridae | *Ctenochaetus striatus* | Reunion:West:St Gilles | ECOMAR:Ich:REU064-2 | REU064_2 | JQ349924 |
| Acanthuridae | *Ctenochaetus striatus* | Reunion:West:St Leu | ECOMAR:Ich:REU1615 | REU1615 | JQ349927 |
| Acanthuridae | *Ctenochaetus striatus* | Reunion:West:St Leu | ECOMAR:Ich:REU1703 | REU1703 | JQ349925 |
| Acanthuridae | *Ctenochaetus striatus* | Reunion:West:St Leu | ECOMAR:Ich:REU2648 | REU2648 | JQ349928 |
| Acanthuridae | *Naso annulatus* | French Polynesia:Society Islands:Moorea | MNHN:Ich:BIOCODES1007 | MBIO1798.4 | HM034241 |
| Acanthuridae | *Naso annulatus* | French Polynesia:Society Islands:Tetiaroa | MNHN:Ich:2009-1645 | MOCNESS M10.1-M0-41 | HM034154 |
| Acanthuridae | *Naso annulatus* | French Polynesia:Society Islands:Tetiaroa | MNHN:Ich:2009-1646 | MOCNESS M10.1-M0-46 | HM034155 |
| Acanthuridae | *Naso annulatus* | Reunion:West:La Saline | ECOMAR:Ich:REU273-1 | REU273_1 | JQ350124 |
| Acanthuridae | *Naso annulatus* | Reunion:West:St Gilles | ECOMAR:Ich:REU331-1 | REU331_1 | JQ350125 |
| Acanthuridae | *Naso hexacanthus* | Reunion:West:St Leu | ECOMAR:Ich:REU0935 | REU0935 | JQ350126 |
| Acanthuridae | *Naso lituratus* | French Polynesia:Society Islands:Moorea | MNHN:Ich:BIOCODES677 | MBIO1002.4 | HM034242 |
| Acanthuridae | *Naso lituratus* | French Polynesia:Society Islands:Moorea | MNHN:Ich:BIOCODES0120 | MBIO177.4 | HM034246 |
| Acanthuridae | *Naso lituratus* | French Polynesia:Society Islands:Moorea | MNHN:Ich:BIOCODES0121 | MBIO178.4 | HM034245 |
| Acanthuridae | *Naso lituratus* | French Polynesia:Society Islands:Moorea | MNHN:Ich:BIOCODES0009 | MBIO29.4 | HM034248 |
| Acanthuridae | *Naso lituratus* | French Polynesia:Society Islands:Moorea | MNHN:Ich:BIOCODES0008 | MBIO30.4 | HM034247 |
| Acanthuridae | *Naso lituratus* | French Polynesia:Society Islands:Moorea | MNHN:Ich:BIOCODES583 | MBIO861.4 | HM034244 |
| Acanthuridae | *Naso lituratus* | French Polynesia:Society Islands:Moorea | MNHN:Ich:BIOCODES584 | MBIO862.4 | HM034243 |
| Acanthuridae | *Naso lituratus* | Reunion:West:St Gilles | ECOMAR:Ich:REU330-1 | REU330_1 | JQ350127 |
| Acanthuridae | *Naso unicornis* | French Polynesia:Society Islands:Moorea | MNHN:Ich:BIOCODES0141 | MBIO206.4 | HM034249 |
| Acanthuridae | *Naso unicornis* | French Polynesia:Society Islands:Tetiaroa | MNHN:Ich:2009-1648 | MOCNESS M10.1-M0-42 | HM034157 |
| Acanthuridae | *Naso unicornis* | French Polynesia:Society Islands:Tetiaroa | MNHN:Ich:2009-1649 | MOCNESS M10.1-M0-44 | HM034158 |
| Acanthuridae | *Naso unicornis* | French Polynesia:Society Islands:Tetiaroa | MNHN:Ich:2009-1650 | MOCNESS M10.1-M0-45 | HM034159 |
| Acanthuridae | *Naso unicornis* | French Polynesia:Society Islands:Tetiaroa | MNHN:Ich:2009-1647 | MOCNESS M9.1-M0-15 | HM034156 |
| Acanthuridae | *Naso unicornis* | Madagascar:West:Nosy Be | ECOMAR:Ich:NBE0638 | NBE0638 | JQ350129 |
| Acanthuridae | *Naso unicornis* | Reunion:West:St Gilles | ECOMAR:Ich:REU063-1 | REU063_1 | JQ350128 |
| Acanthuridae | *Naso unicornis* | Reunion:West:St Gilles | ECOMAR:Ich:REU063-2 | REU063_2 | JQ350132 |
| Acanthuridae | *Naso unicornis* | Reunion:West:St Gilles | ECOMAR:Ich:REU154-1 | REU154_1 | JQ350130 |
| Acanthuridae | *Naso unicornis* | Reunion:West:St Gilles | ECOMAR:Ich:REU1663 | REU1663 | JQ350131 |
| Acanthuridae | *Naso vlamingii* | French Polynesia:Society Islands:Moorea | MNHN:Ich:BIOCODES780 | MBIO1214.4 | HM034251 |
| Acanthuridae | *Naso vlamingii* | French Polynesia:Society Islands:Moorea | SI:Ich:MBIO1215 | MBIO1215.4 | HM034250 |
| Acanthuridae | *Paracanthurus hepatus* | Madagascar:West:Nosy Be | ECOMAR:Ich:NBE1104 | NBE1104 | JQ350163 |
| Acanthuridae | *Paracanthurus hepatus* | Madagascar:West:Nosy Be | ECOMAR:Ich:NBE1105 | NBE1105 | JQ350162 |
| Acanthuridae | *Paracanthurus hepatus* | Madagascar:West:Nosy Be | ECOMAR:Ich:NBE1106 | NBE1106 | JQ350160 |
| Acanthuridae | *Paracanthurus hepatus* | Reunion:West:St Gilles | ECOMAR:Ich:REU0747 | REU0747 | JQ350161 |
| Acanthuridae | *Zebrasoma desjardinii* | Reunion:West:La Saline | ECOMAR:Ich:REU0295 | REU0295 | JQ350426 |
| Acanthuridae | *Zebrasoma desjardinii* | Reunion:West:La Saline | ECOMAR:Ich:REU0296 | REU0296 | JQ350425 |
| Acanthuridae | *Zebrasoma desjardinii* | Reunion:West:St Gilles | ECOMAR:Ich:REU068-1 | REU068_1 | JQ350424 |
| Acanthuridae | *Zebrasoma desjardinii* | Reunion:West:St Gilles | ECOMAR:Ich:REU0705 | REU0705 | JQ350423 |
| Acanthuridae | *Zebrasoma desjardinii* | Reunion:West:St Gilles | ECOMAR:Ich:REU101-1 | REU101_1 | JQ350422 |
| Acanthuridae | *Zebrasoma rostratum* | French Polynesia:Marquesas Islands:Mohotane | CRIOBE:Ich:MBIO18243 | MBIO18243 | HM034282 |
| Acanthuridae | *Zebrasoma scopas* | French Polynesia:Society Islands:Moorea | MNHN:Ich:BIOCODES0106 | MBIO160.4 | HM034286 |
| Acanthuridae | *Zebrasoma scopas* | French Polynesia:Society Islands:Moorea | MNHN:Ich:BIOCODES0107 | MBIO161.4 | HM034285 |
| Acanthuridae | *Zebrasoma scopas* | French Polynesia:Society Islands:Moorea | CRIOBE:Ich:MBIO18386 | MBIO18386 | HM034283 |
| Acanthuridae | *Zebrasoma scopas* | French Polynesia:Society Islands:Moorea | SI:Ich:MBIO442 | MBIO442.4 | HM034284 |
| Acanthuridae | *Zebrasoma scopas* | Madagascar:West:Nosy Be | ECOMAR:Ich:NBE0012 | NBE0012 | JQ350428 |
| Acanthuridae | *Zebrasoma scopas* | Madagascar:West:Nosy Be | ECOMAR:Ich:NBE0013 | NBE0013 | JQ350431 |
| Acanthuridae | *Zebrasoma scopas* | Reunion:West:St Gilles | ECOMAR:Ich:REU009-1 | REU009_1 | JQ350427 |
| Acanthuridae | *Zebrasoma scopas* | Reunion:West:St Gilles | ECOMAR:Ich:REU066-1 | REU066_1 | JQ350429 |
| Acanthuridae | *Zebrasoma scopas* | Reunion:West:St Gilles | ECOMAR:Ich:REU1661 | REU1661 | JQ350430 |
| Acanthuridae | *Zebrasoma veliferum* | French Polynesia:Society Islands:Moorea | MNHN:Ich:BIOCODES697 | MBIO1033.4 | HM034288 |
| Acanthuridae | *Zebrasoma veliferum* | French Polynesia:Society Islands:Moorea | SI:Ich:MBIO1034 | MBIO1034.4 | HM034287 |
| Albulidae | *Albula glossodonta* | French Polynesia:Society Islands:Moorea | SI:Ich:MBIO1821 | MBIO1821.4 | JQ431400 |
| Anguillidae | *Anguilla marmorata* | French Polynesia:Society Islands:Moorea | MNHN:Ich:BIOCODES828 | MBIO1300.4 | JQ431413 |
| Anguillidae | *Anguilla marmorata* | French Polynesia:Society Islands:Moorea | SI:Ich:MBIO1301 | MBIO1301.4 | JQ431414 |
| Anguillidae | *Anguilla megastoma* | French Polynesia:Society Islands:Moorea | MNHN:Ich:BIOCODES1019 | MBIO1818.4 | JQ431416 |
| Anguillidae | *Anguilla megastoma* | French Polynesia:Society Islands:Moorea | SI:Ich:MBIO1819 | MBIO1819.4 | JQ431415 |
| Antennariidae | *Antennarius coccineus* | French Polynesia:Society Islands:Moorea | MNHN:Ich:BIOCODES722 | MBIO1107.4 | JQ431417 |
| Antennariidae | *Antennarius coccineus* | French Polynesia:Society Islands:Moorea | MNHN:Ich:BIOCODES0246 | MBIO361.4 | JQ431418 |
| Antennariidae | *Antennarius coccineus* | French Polynesia:Society Islands:Moorea | MNHN:Ich:BIOCODES0247 | MBIO363.4 | JQ431419 |
| Antennariidae | *Antennarius coccineus* | French Polynesia:Society Islands:Moorea | MNHN:Ich:BIOCODES0248 | MBIO364.4 | JQ431420 |
| Apogonidae | *Apogon angustatus* | French Polynesia:Society Islands:Moorea | SI:Ich:MBIO1374 | MBIO1374.4 | JQ431428 |
| Apogonidae | *Apogon angustatus* | French Polynesia:Society Islands:Moorea | MNHN:Ich:BIOCODES664 | MBIO979.4 | JQ431427 |
| Apogonidae | *Apogon angustatus* | French Polynesia:Society Islands:Moorea | MNHN:Ich:BIOCODES661 | MBIO980.4 | JQ431426 |
| Apogonidae | *Apogon angustatus* | Madagascar:West:Nosy Be | ECOMAR:Ich:NBE0393 | NBE0393 | JQ349707 |
| Apogonidae | *Apogon angustatus* | Madagascar:West:Nosy Be | ECOMAR:Ich:NBE0394 | NBE0394 | JQ349703 |
| Apogonidae | *Apogon angustatus* | Madagascar:West:Nosy Be | ECOMAR:Ich:NBE0395 | NBE0395 | JQ349704 |
| Apogonidae | *Apogon angustatus* | Madagascar:West:Nosy Be | ECOMAR:Ich:NBE0396 | NBE0396 | JQ349705 |
| Apogonidae | *Apogon angustatus* | Madagascar:West:Nosy Be | ECOMAR:Ich:NBE1132 | NBE1132 | JQ349706 |
| Apogonidae | *Apogon apogonides* | French Polynesia:Society Islands:Moorea | MNHN:Ich:BIOCODES724 | MBIO1120.4 | JQ431430 |
| Apogonidae | *Apogon apogonides* | French Polynesia:Society Islands:Moorea | MNHN:Ich:BIOCODES725 | MBIO1121.4 | JQ431429 |
| Apogonidae | *Apogon apogonides* | Madagascar:West:Nosy Be | ECOMAR:Ich:NBE1215 | NBE1215 | JQ349712 |
| Apogonidae | *Apogon apogonides* | Madagascar:West:Nosy Be | ECOMAR:Ich:NBE1216 | NBE1216 | JQ349708 |
| Apogonidae | *Apogon apogonides* | Madagascar:West:Nosy Be | ECOMAR:Ich:NBE1217 | NBE1217 | JQ349711 |
| Apogonidae | *Apogon apogonides* | Madagascar:West:Nosy Be | ECOMAR:Ich:NBE1218 | NBE1218 | JQ349710 |
| Apogonidae | *Apogon apogonides* | Madagascar:West:Nosy Be | ECOMAR:Ich:NBE1219 | NBE1219 | JQ349709 |
| Apogonidae | *Apogon aureus* | Madagascar:West:Nosy Be | ECOMAR:Ich:NBE1220 | NBE1220 | JQ349713 |
| Apogonidae | *Apogon aureus* | Madagascar:West:Nosy Be | ECOMAR:Ich:NBE1221 | NBE1221 | JQ349716 |
| Apogonidae | *Apogon aureus* | Madagascar:West:Nosy Be | ECOMAR:Ich:NBE1222 | NBE1222 | JQ349715 |
| Apogonidae | *Apogon aureus* | Madagascar:West:Nosy Be | ECOMAR:Ich:NBE1223 | NBE1223 | JQ349714 |
| Apogonidae | *Apogon caudicinctus* | Madagascar:West:Nosy Be | ECOMAR:Ich:NBE0106 | NBE0106 | JQ349717 |
| Apogonidae | *Apogon coccineus* | Madagascar:West:Nosy Be | ECOMAR:Ich:NBE0522 | NBE0522 | JQ349722 |
| Apogonidae | *Apogon coccineus* | Madagascar:West:Nosy Be | ECOMAR:Ich:NBE0523 | NBE0523 | JQ349718 |
| Apogonidae | *Apogon coccineus* | Madagascar:West:Nosy Be | ECOMAR:Ich:NBE0524 | NBE0524 | JQ349720 |
| Apogonidae | *Apogon coccineus* | Madagascar:West:Nosy Be | ECOMAR:Ich:NBE0525 | NBE0525 | JQ349719 |
| Apogonidae | *Apogon coccineus* | Madagascar:West:Nosy Be | ECOMAR:Ich:NBE0526 | NBE0526 | JQ349721 |
| Apogonidae | *Apogon cookii* | Madagascar:West:Nosy Be | ECOMAR:Ich:NBE0266 | NBE0266 | JQ349725 |
| Apogonidae | *Apogon cookii* | Madagascar:West:Nosy Be | ECOMAR:Ich:NBE0267 | NBE0267 | JQ349723 |
| Apogonidae | *Apogon cookii* | Madagascar:West:Nosy Be | ECOMAR:Ich:NBE1029 | NBE1029 | JQ349724 |
| Apogonidae | *Apogon deetsie* | French Polynesia:Society Islands:Moorea | MNHN:Ich:BIOCODES0861 | MBIO1411.4 | JQ431431 |
| Apogonidae | *Apogon doryssa* | French Polynesia:Society Islands:Moorea | MNHN:Ich:BIOCODES0243 | MBIO357.4 | JQ431432 |
| Apogonidae | *Apogon doryssa* | French Polynesia:Society Islands:Moorea | MNHN:Ich:BIOCODES0244 | MBIO358.4 | JQ431433 |
| Apogonidae | *Apogon exostigma* | French Polynesia:Society Islands:Moorea | MNHN:Ich:BIOCODES0281 | MBIO496.4 | JQ431435 |
| Apogonidae | *Apogon exostigma* | French Polynesia:Society Islands:Moorea | MNHN:Ich:BIOCODES0345 | MBIO497.4 | JQ431434 |
| Apogonidae | *Apogon exostigma* | Madagascar:West:Nosy Be | ECOMAR:Ich:NBE0101 | NBE0101 | JQ349730 |
| Apogonidae | *Apogon exostigma* | Madagascar:West:Nosy Be | ECOMAR:Ich:NBE0102 | NBE0102 | JQ349729 |
| Apogonidae | *Apogon exostigma* | Madagascar:West:Nosy Be | ECOMAR:Ich:NBE0103 | NBE0103 | JQ349728 |
| Apogonidae | *Apogon exostigma* | Madagascar:West:Nosy Be | ECOMAR:Ich:NBE0533 | NBE0533 | JQ349727 |
| Apogonidae | *Apogon exostigma* | Madagascar:West:Nosy Be | ECOMAR:Ich:NBE0534 | NBE0534 | JQ349726 |
| Apogonidae | *Apogon fraenatus* | French Polynesia:Society Islands:Moorea | MNHN:Ich:BIOCODES0450 | MBIO656.4 | JQ431437 |
| Apogonidae | *Apogon fraenatus* | French Polynesia:Society Islands:Moorea | SI:Ich:MBIO657 | MBIO657.4 | JQ431436 |
| Apogonidae | *Apogon fraenatus* | Madagascar:West:Nosy Be | ECOMAR:Ich:NBE0474 | NBE0474 | JQ349731 |
| Apogonidae | *Apogon fraenatus* | Madagascar:West:Nosy Be | ECOMAR:Ich:NBE0475 | NBE0475 | JQ349732 |
| Apogonidae | *Apogon fraenatus* | Madagascar:West:Nosy Be | ECOMAR:Ich:NBE0596 | NBE0596 | JQ349733 |
| Apogonidae | *Apogon fraenatus* | Madagascar:West:Nosy Be | ECOMAR:Ich:NBE1224 | NBE1224 | JQ349734 |
| Apogonidae | *Apogon fragilis* | Madagascar:West:Nosy Be | ECOMAR:Ich:NBE0527 | NBE0527 | JQ349737 |
| Apogonidae | *Apogon fragilis* | Madagascar:West:Nosy Be | ECOMAR:Ich:NBE0528 | NBE0528 | JQ349736 |
| Apogonidae | *Apogon fragilis* | Madagascar:West:Nosy Be | ECOMAR:Ich:NBE0529 | NBE0529 | JQ349739 |
| Apogonidae | *Apogon fragilis* | Madagascar:West:Nosy Be | ECOMAR:Ich:NBE0530 | NBE0530 | JQ349740 |
| Apogonidae | *Apogon fragilis* | Madagascar:West:Nosy Be | ECOMAR:Ich:NBE0531 | NBE0531 | JQ349735 |
| Apogonidae | *Apogon fragilis* | Madagascar:West:Nosy Be | ECOMAR:Ich:NBE1033 | NBE1033 | JQ349738 |
| Apogonidae | *Apogon fuscus* | Reunion:West:St Gilles | ECOMAR:Ich:REU113-1 | REU113_1 | JQ349741 |
| Apogonidae | *Apogon fuscus* | Reunion:West:St Gilles | ECOMAR:Ich:REU1637 | REU1637 | JQ349747 |
| Apogonidae | *Apogon fuscus* | Reunion:West:St Gilles | ECOMAR:Ich:REU1638 | REU1638 | JQ349746 |
| Apogonidae | *Apogon fuscus* | Reunion:West:St Gilles | ECOMAR:Ich:REU1639 | REU1639 | JQ349745 |
| Apogonidae | *Apogon fuscus* | Reunion:West:St Gilles | ECOMAR:Ich:REU1640 | REU1640 | JQ349744 |
| Apogonidae | *Apogon fuscus* | Reunion:West:St Gilles | ECOMAR:Ich:REU1641 | REU1641 | JQ349743 |
| Apogonidae | *Apogon fuscus* | Reunion:West:St Gilles | ECOMAR:Ich:REU1646 | REU1646 | JQ349742 |
| Apogonidae | *Apogon guamensis* | Madagascar:West:Nosy Be | ECOMAR:Ich:NBE0284 | NBE0284 | JQ349751 |
| Apogonidae | *Apogon guamensis* | Madagascar:West:Nosy Be | ECOMAR:Ich:NBE0285 | NBE0285 | JQ349752 |
| Apogonidae | *Apogon guamensis* | Madagascar:West:Nosy Be | ECOMAR:Ich:NBE0286 | NBE0286 | JQ349750 |
| Apogonidae | *Apogon guamensis* | Madagascar:West:Nosy Be | ECOMAR:Ich:NBE0287 | NBE0287 | JQ349749 |
| Apogonidae | *Apogon guamensis* | Madagascar:West:Nosy Be | ECOMAR:Ich:NBE1028 | NBE1028 | JQ349748 |
| Apogonidae | *Apogon indicus* | French Polynesia:Society Islands:Moorea | MNHN:Ich:BIOCODES0161 | MBIO228.4 | JQ431439 |
| Apogonidae | *Apogon kallopterus* | French Polynesia:Society Islands:Moorea | MNHN:Ich:BIOCODES0227 | MBIO332.4 | JQ431444 |
| Apogonidae | *Apogon kallopterus* | French Polynesia:Society Islands:Moorea | MNHN:Ich:BIOCODES0228 | MBIO333.4 | JQ431441 |
| Apogonidae | *Apogon kallopterus* | French Polynesia:Society Islands:Moorea | MNHN:Ich:BIOCODES525 | MBIO776.4 | JQ431442 |
| Apogonidae | *Apogon kallopterus* | French Polynesia:Society Islands:Moorea | MNHN:Ich:BIOCODES526 | MBIO777.4 | JQ431443 |
| Apogonidae | *Apogon kallopterus* | French Polynesia:Society Islands:Moorea | MNHN:Ich:BIOCODES0610 | MBIO899.4 | JQ431440 |
| Apogonidae | *Apogon kallopterus* | Madagascar:West:Nosy Be | ECOMAR:Ich:NBE0403 | NBE0403 | JQ349754 |
| Apogonidae | *Apogon kallopterus* | Madagascar:West:Nosy Be | ECOMAR:Ich:NBE0404 | NBE0404 | JQ349764 |
| Apogonidae | *Apogon kallopterus* | Madagascar:West:Nosy Be | ECOMAR:Ich:NBE0405 | NBE0405 | JQ349756 |
| Apogonidae | *Apogon kallopterus* | Madagascar:West:Nosy Be | ECOMAR:Ich:NBE0406 | NBE0406 | JQ349757 |
| Apogonidae | *Apogon kallopterus* | Madagascar:West:Nosy Be | ECOMAR:Ich:NBE1127 | NBE1127 | JQ349760 |
| Apogonidae | *Apogon kallopterus* | Madagascar:West:Nosy Be | ECOMAR:Ich:NBE1128 | NBE1128 | JQ349759 |
| Apogonidae | *Apogon kallopterus* | Madagascar:West:Nosy Be | ECOMAR:Ich:NBE1226 | NBE1226 | JQ349755 |
| Apogonidae | *Apogon kallopterus* | Madagascar:West:Nosy Be | ECOMAR:Ich:NBE1227 | NBE1227 | JQ349758 |
| Apogonidae | *Apogon kallopterus* | Reunion:West:St Gilles | ECOMAR:Ich:REU1636 | REU1636 | JQ349763 |
| Apogonidae | *Apogon kallopterus* | Reunion:West:La Saline | ECOMAR:Ich:REU2541 | REU2541 | JQ349761 |
| Apogonidae | *Apogon kallopterus* | Reunion:West:St Leu | ECOMAR:Ich:REU2703 | REU2703 | JQ349762 |
| Apogonidae | *Apogon nigrofasciatus* | French Polynesia:Society Islands:Moorea | MNHN:Ich:BIOCODES317 | MBIO458.4 | JQ431447 |
| Apogonidae | *Apogon nigrofasciatus* | French Polynesia:Society Islands:Moorea | MNHN:Ich:BIOCODES316 | MBIO459.4 | JQ431448 |
| Apogonidae | *Apogon nigrofasciatus* | French Polynesia:Society Islands:Moorea | MNHN:Ich:BIOCODES541 | MBIO798.4 | JQ431446 |
| Apogonidae | *Apogon nigrofasciatus* | French Polynesia:Society Islands:Moorea | MNHN:Ich:BIOCODES542 | MBIO799.4 | JQ431445 |
| Apogonidae | *Apogon savayensis* | French Polynesia:Society Islands:Moorea | MNHN:Ich:BIOCODES0946 | MBIO1608.4 | JQ431452 |
| Apogonidae | *Apogon savayensis* | French Polynesia:Society Islands:Moorea | MNHN:Ich:BIOCODES0948 | MBIO1609.4 | JQ431451 |
| Apogonidae | *Apogon savayensis* | French Polynesia:Society Islands:Moorea | MNHN:Ich:BIOCODES0353 | MBIO505.4 | JQ431449 |
| Apogonidae | *Apogon savayensis* | French Polynesia:Society Islands:Moorea | MNHN:Ich:BIOCODES0354 | MBIO506.4 | JQ431450 |
| Apogonidae | *Apogon semiornatus* | Madagascar:West:Nosy Be | ECOMAR:Ich:NBE0399 | NBE0399 | JQ349769 |
| Apogonidae | *Apogon semiornatus* | Madagascar:West:Nosy Be | ECOMAR:Ich:NBE0400 | NBE0400 | JQ349765 |
| Apogonidae | *Apogon semiornatus* | Madagascar:West:Nosy Be | ECOMAR:Ich:NBE0401 | NBE0401 | JQ349767 |
| Apogonidae | *Apogon semiornatus* | Madagascar:West:Nosy Be | ECOMAR:Ich:NBE0402 | NBE0402 | JQ349766 |
| Apogonidae | *Apogon semiornatus* | Madagascar:West:Nosy Be | ECOMAR:Ich:NBE1203 | NBE1203 | JQ349768 |
| Apogonidae | *Apogon taeniophorus* | French Polynesia:Society Islands:Moorea | MNHN:Ich:BIOCODES769 | MBIO1197.4 | JQ431453 |
| Apogonidae | *Apogon taeniophorus* | French Polynesia:Society Islands:Moorea | MNHN:Ich:BIOCODES0996 | MBIO1716.4 | JQ431454 |
| Apogonidae | *Apogon taeniopterus* | French Polynesia:Society Islands:Moorea | MNHN:Ich:BIOCODES0945 | MBIO1604.4 | JQ431455 |
| Apogonidae | *Apogon taeniopterus* | French Polynesia:Society Islands:Moorea | MNHN:Ich:BIOCODES0943 | MBIO1605.4 | JQ431456 |
| Apogonidae | *Apogonichthys ocellatus* | French Polynesia:Society Islands:Moorea | MNHN:Ich:BIOCODES499 | MBIO739.4 | JQ431438 |
| Apogonidae | *Apogonichthys ocellatus* | Reunion:West:La Saline | ECOMAR:Ich:REU2600 | REU2600 | JQ349753 |
| Apogonidae | *Archamia flavofasciata* | Madagascar:West:Nosy Be | ECOMAR:Ich:NBE1031 | NBE1031 | JQ349776 |
| Apogonidae | *Archamia flavofasciata* | Madagascar:West:Nosy Be | ECOMAR:Ich:NBE1032 | NBE1032 | JQ349774 |
| Apogonidae | *Archamia flavofasciata* | Madagascar:West:Nosy Be | ECOMAR:Ich:NBE1034 | NBE1034 | JQ349775 |
| Apogonidae | *Archamia flavofasciata* | Madagascar:West:Nosy Be | ECOMAR:Ich:NBE1035 | NBE1035 | JQ349777 |
| Apogonidae | *Cercamia cladara* | French Polynesia:Society Islands:Moorea | MNHN:Ich:BIOCODES0448 | MBIO646.4 | JQ431579 |
| Apogonidae | *Cheilodipterus artus* | French Polynesia:Society Islands:Moorea | MNHN:Ich:BIOCODES741 | MBIO1131.4 | JQ431610 |
| Apogonidae | *Cheilodipterus artus* | Madagascar:West:Nosy Be | ECOMAR:Ich:NBE0469 | NBE0469 | JQ349883 |
| Apogonidae | *Cheilodipterus artus* | Madagascar:West:Nosy Be | ECOMAR:Ich:NBE0470 | NBE0470 | JQ349884 |
| Apogonidae | *Cheilodipterus artus* | Madagascar:West:Nosy Be | ECOMAR:Ich:NBE0471 | NBE0471 | JQ349882 |
| Apogonidae | *Cheilodipterus artus* | Madagascar:West:Nosy Be | ECOMAR:Ich:NBE0472 | NBE0472 | JQ349886 |
| Apogonidae | *Cheilodipterus artus* | Madagascar:West:Nosy Be | ECOMAR:Ich:NBE0473 | NBE0473 | JQ349885 |
| Apogonidae | *Cheilodipterus macrodon* | French Polynesia:Society Islands:Moorea | MNHN:Ich:BIOCODES613 | MBIO906.4 | JQ431611 |
| Apogonidae | *Cheilodipterus macrodon* | French Polynesia:Society Islands:Moorea | MNHN:Ich:BIOCODES612 | MBIO907.4 | JQ431612 |
| Apogonidae | *Cheilodipterus quinquelineatus* | French Polynesia:Society Islands:Moorea | MNHN:Ich:BIOCODES1031 | MBIO1838.4 | JQ431614 |
| Apogonidae | *Cheilodipterus quinquelineatus* | French Polynesia:Society Islands:Moorea | MNHN:Ich:BIOCODES1032 | MBIO1839.4 | JQ431613 |
| Apogonidae | *Cheilodipterus quinquelineatus* | Madagascar:West:Nosy Be | ECOMAR:Ich:NBE0127 | NBE0127 | JQ349890 |
| Apogonidae | *Cheilodipterus quinquelineatus* | Madagascar:West:Nosy Be | ECOMAR:Ich:NBE0129 | NBE0129 | JQ349889 |
| Apogonidae | *Cheilodipterus quinquelineatus* | Madagascar:West:Nosy Be | ECOMAR:Ich:NBE0130 | NBE0130 | JQ349888 |
| Apogonidae | *Cheilodipterus quinquelineatus* | Madagascar:West:Nosy Be | ECOMAR:Ich:NBE0131 | NBE0131 | JQ349887 |
| Apogonidae | *Foa brachygramma* | Madagascar:West:Nosy Be | ECOMAR:Ich:NBE0270 | NBE0270 | JQ349980 |
| Apogonidae | *Fowleria aurita* | Madagascar:West:Nosy Be | ECOMAR:Ich:NBE0271 | NBE0271 | JQ349981 |
| Apogonidae | *Fowleria isostigma* | French Polynesia:Society Islands:Moorea | MNHN:Ich:BIOCODES0937 | MBIO1588.4 | JQ431746 |
| Apogonidae | *Fowleria marmorata* | French Polynesia:Society Islands:Moorea | MNHN:Ich:BIOCODES0457 | MBIO668.4 | JQ431747 |
| Apogonidae | *Fowleria marmorata* | Madagascar:West:Nosy Be | ECOMAR:Ich:NBE0149 | NBE0149 | JQ349982 |
| Apogonidae | *Fowleria marmorata* | Madagascar:West:Nosy Be | ECOMAR:Ich:NBE0150 | NBE0150 | JQ349986 |
| Apogonidae | *Fowleria marmorata* | Madagascar:West:Nosy Be | ECOMAR:Ich:NBE0151 | NBE0151 | JQ349983 |
| Apogonidae | *Fowleria marmorata* | Madagascar:West:Nosy Be | ECOMAR:Ich:NBE0152 | NBE0152 | JQ349985 |
| Apogonidae | *Fowleria marmorata* | Madagascar:West:Nosy Be | ECOMAR:Ich:NBE0153 | NBE0153 | JQ349984 |
| Apogonidae | *Fowleria vaiulae* | French Polynesia:Society Islands:Moorea | MNHN:Ich:BIOCODES0242 | MBIO350.4 | JQ431749 |
| Apogonidae | *Fowleria vaiulae* | French Polynesia:Society Islands:Moorea | MNHN:Ich:BIOCODES0239 | MBIO351.4 | JQ431748 |
| Apogonidae | *Fowleria variegata* | Madagascar:West:Nosy Be | ECOMAR:Ich:NBE0268 | NBE0268 | JQ349988 |
| Apogonidae | *Fowleria variegata* | Madagascar:West:Nosy Be | ECOMAR:Ich:NBE0269 | NBE0269 | JQ349987 |
| Apogonidae | *Fowleria variegata* | Madagascar:West:Nosy Be | ECOMAR:Ich:NBE0272 | NBE0272 | JQ349989 |
| Apogonidae | *Fowleria variegata* | Madagascar:West:Nosy Be | ECOMAR:Ich:NBE0273 | NBE0273 | JQ349990 |
| Apogonidae | *Fowleria variegata* | Reunion:West:St Gilles | ECOMAR:Ich:REU1633 | REU1633 | JQ349993 |
| Apogonidae | *Fowleria variegata* | Reunion:West:St Gilles | ECOMAR:Ich:REU1634 | REU1634 | JQ349992 |
| Apogonidae | *Fowleria variegata* | Reunion:West:St Gilles | ECOMAR:Ich:REU1635 | REU1635 | JQ349991 |
| Apogonidae | *Gymnapogon africanus* | Madagascar:West:Nosy Be | ECOMAR:Ich:NBE1176 | NBE1176 | JQ350015 |
| Apogonidae | *Gymnapogon* sp. | Reunion:West:St Leu | ECOMAR:Ich:REU1022 | REU1022 | JQ350016 |
| Apogonidae | *Gymnapogon urospilotus* | French Polynesia:Society Islands:Moorea | MNHN:Ich:BIOCODES0422 | MBIO601.4 | JQ431782 |
| Apogonidae | *Gymnapogon urospilotus* | French Polynesia:Society Islands:Moorea | MNHN:Ich:BIOCODES0420 | MBIO602.4 | JQ431783 |
| Apogonidae | *Pseudamiops gracilicauda* | French Polynesia:Society Islands:Moorea | SI:Ich:MBIO1601 | MBIO1601.4 | JQ432049 |
| Apogonidae | *Pseudamiops gracilicauda* | French Polynesia:Society Islands:Moorea | SI:Ich:MBIO645 | MBIO645.4 | JQ432048 |
| Apogonidae | *Rhabdamia cypselura* | Madagascar:West:Nosy Be | ECOMAR:Ich:NBE1204 | NBE1204 | JQ350300 |
| Apogonidae | *Rhabdamia cypselura* | Madagascar:West:Nosy Be | ECOMAR:Ich:NBE1205 | NBE1205 | JQ350301 |
| Apogonidae | *Rhabdamia cypselura* | Madagascar:West:Nosy Be | ECOMAR:Ich:NBE1206 | NBE1206 | JQ350302 |
| Apogonidae | *Rhabdamia cypselura* | Madagascar:West:Nosy Be | ECOMAR:Ich:NBE1207 | NBE1207 | JQ350303 |
| Aulostomidae | *Aulostomus chinensis* | French Polynesia:Society Islands:Moorea | MNHN:Ich:BIOCODES502 | MBIO748.4 | JQ431472 |
| Aulostomidae | *Aulostomus chinensis* | Reunion:West:St Leu | ECOMAR:Ich:REU1675 | REU1675 | JQ349792 |
| Aulostomidae | *Aulostomus chinensis* | Reunion:West:St Leu | ECOMAR:Ich:REU2643 | REU2643 | JQ349793 |
| Aulostomidae | *Aulostomus chinensis* | Reunion:West:St Leu | ECOMAR:Ich:REU2644 | REU2644 | JQ349794 |
| Balistidae | *Balistapus undulatus* | French Polynesia:Society Islands:Moorea | MNHN:Ich:BIOCODES0015 | MBIO39.4 | JQ431474 |
| Balistidae | *Balistapus undulatus* | French Polynesia:Society Islands:Moorea | MNHN:Ich:BIOCODES0016 | MBIO40.4 | JQ431475 |
| Balistidae | *Balistapus undulatus* | Madagascar:West:Nosy Be | ECOMAR:Ich:NBE0360 | NBE0360 | JQ349796 |
| Balistidae | *Balistapus undulatus* | Madagascar:West:Nosy Be | ECOMAR:Ich:NBE1257 | NBE1257 | JQ349795 |
| Balistidae | *Balistoides conspicillum* | Reunion:West:St Leu | ECOMAR:Ich:REU0924 | REU0924 | JQ349797 |
| Balistidae | *Balistoides viridescens* | French Polynesia:Society Islands:Moorea | MNHN:Ich:BIOCODES673 | MBIO1000.4 | JQ431478 |
| Balistidae | *Balistoides viridescens* | French Polynesia:Society Islands:Moorea | MNHN:Ich:BIOCODES744 | MBIO1156.4 | JQ431477 |
| Balistidae | *Balistoides viridescens* | French Polynesia:Society Islands:Moorea | MNHN:Ich:BIOCODES808 | MBIO1268.4 | JQ431476 |
| Balistidae | *Melichthys niger* | French Polynesia:Society Islands:Moorea | MNHN:Ich:BIOCODES0147 | MBIO214.4 | JQ431903 |
| Balistidae | *Melichthys vidua* | French Polynesia:Society Islands:Moorea | MNHN:Ich:BIOCODES0184 | MBIO260.4 | JQ431904 |
| Balistidae | *Melichthys vidua* | French Polynesia:Society Islands:Moorea | MNHN:Ich:BIOCODES0198 | MBIO284.4 | JQ431906 |
| Balistidae | *Melichthys vidua* | French Polynesia:Society Islands:Moorea | SI:Ich:MBIO285 | MBIO285.4 | JQ431905 |
| Balistidae | *Odonus niger* | French Polynesia:Society Islands:Moorea | MNHN:Ich:BIOCODES0464 | MBIO669.4 | JQ431936 |
| Balistidae | *Odonus niger* | French Polynesia:Society Islands:Moorea | MNHN:Ich:BIOCODES0463 | MBIO670.4 | JQ431937 |
| Balistidae | *Odonus niger* | French Polynesia:Society Islands:Moorea | MNHN:Ich:BIOCODES626 | MBIO925.4 | JQ431938 |
| Balistidae | *Rhinecanthus aculeatus* | French Polynesia:Society Islands:Moorea | MNHN:Ich:BIOCODES554 | MBIO817.4 | JQ432084 |
| Balistidae | *Rhinecanthus aculeatus* | French Polynesia:Society Islands:Moorea | MNHN:Ich:BIOCODES552 | MBIO818.4 | JQ432085 |
| Balistidae | *Rhinecanthus aculeatus* | Madagascar:West:Nosy Be | ECOMAR:Ich:NBE0645 | NBE0645 | JQ350305 |
| Balistidae | *Rhinecanthus aculeatus* | Reunion:West:St Gilles | ECOMAR:Ich:REU070-1 | REU070_1 | JQ350304 |
| Balistidae | *Rhinecanthus aculeatus* | Reunion:West:St Gilles | ECOMAR:Ich:REU070-2 | REU070_2 | JQ350306 |
| Balistidae | *Rhinecanthus lunula* | French Polynesia:Society Islands:Moorea | MNHN:Ich:BIOCODES790 | MBIO1228.4 | JQ432087 |
| Balistidae | *Rhinecanthus lunula* | French Polynesia:Society Islands:Moorea | SI:Ich:MBIO1229 | MBIO1229.4 | JQ432086 |
| Balistidae | *Rhinecanthus rectangulus* | French Polynesia:Society Islands:Moorea | MNHN:Ich:BIOCODES0189 | MBIO271.4 | JQ432088 |
| Balistidae | *Rhinecanthus rectangulus* | French Polynesia:Society Islands:Moorea | MNHN:Ich:BIOCODES0190 | MBIO272.4 | JQ432089 |
| Balistidae | *Rhinecanthus rectangulus* | Reunion:West:St Gilles | ECOMAR:Ich:REU0778 | REU0778 | JQ350307 |
| Balistidae | *Sufflamen bursa* | French Polynesia:Society Islands:Moorea | MNHN:Ich:BIOCODES0449 | MBIO651.4 | JQ432174 |
| Balistidae | *Sufflamen bursa* | French Polynesia:Society Islands:Moorea | SI:Ich:MBIO652 | MBIO652.4 | JQ432175 |
| Balistidae | *Sufflamen bursa* | Reunion:West:St Gilles | ECOMAR:Ich:REU0700 | REU0700 | JQ350376 |
| Balistidae | *Sufflamen bursa* | Reunion:West:St Gilles | ECOMAR:Ich:REU0701 | REU0701 | JQ350377 |
| Balistidae | *Sufflamen chrysopterum* | Madagascar:West:Nosy Be | ECOMAR:Ich:NBE0357 | NBE0357 | JQ350379 |
| Balistidae | *Sufflamen chrysopterum* | Madagascar:West:Nosy Be | ECOMAR:Ich:NBE0358 | NBE0358 | JQ350378 |
| Balistidae | *Sufflamen chrysopterum* | Madagascar:West:Nosy Be | ECOMAR:Ich:NBE1148 | NBE1148 | JQ350382 |
| Balistidae | *Sufflamen chrysopterum* | Reunion:West:St Gilles | ECOMAR:Ich:REU0728 | REU0728 | JQ350381 |
| Balistidae | *Sufflamen chrysopterum* | Reunion:West:St Leu | ECOMAR:Ich:REU0910 | REU0910 | JQ350380 |
| Balistidae | *Sufflamen fraenatum* | French Polynesia:Society Islands:Moorea | MNHN:Ich:BIOCODES735 | MBIO1137.4 | JQ432176 |
| Balistidae | *Sufflamen fraenatum* | French Polynesia:Society Islands:Moorea | SI:Ich:MBIO1138 | MBIO1138.4 | JQ432177 |
| Balistidae | *Xanthichthys auromarginatus* | French Polynesia:Society Islands:Moorea | MNHN:Ich:BIOCODES0957 | MBIO1617.4 | JQ432220 |
| Belonidae | *Tylosurus crocodilus* | French Polynesia:Society Islands:Moorea | MNHN:Ich:BIOCODES0139 | MBIO204.4 | JQ432203 |
| Belonidae | *Tylosurus crocodilus* | French Polynesia:Society Islands:Moorea | SI:Ich:MBIO954 | MBIO954.4 | JQ432202 |
| Blenniidae | *Antennablennius bifilum* | Reunion:West:St Gilles | ECOMAR:Ich:REU1681 | REU1681 | JQ349702 |
| Blenniidae | *Aspidontus taeniatus* | Madagascar:West:Nosy Be | ECOMAR:Ich:NBE1193 | NBE1193 | JQ349783 |
| Blenniidae | *Aspidontus taeniatus* | Madagascar:West:Nosy Be | ECOMAR:Ich:NBE1194 | NBE1194 | JQ349782 |
| Blenniidae | *Aspidontus taeniatus* | Reunion:West:St Gilles | ECOMAR:Ich:REU0504 | REU0504 | JQ349784 |
| Blenniidae | *Blenniella gibbifrons* | French Polynesia:Society Islands:Moorea | MNHN:Ich:BIOCODES0979 | MBIO1667.4 | JQ431485 |
| Blenniidae | *Blenniella gibbifrons* | French Polynesia:Society Islands:Moorea | MNHN:Ich:BIOCODES0980 | MBIO1668.4 | JQ431486 |
| Blenniidae | *Blenniella gibbifrons* | Reunion:South:St Philippe | ECOMAR:Ich:REU1851 | REU1851 | JQ349798 |
| Blenniidae | *Blenniella paula* | French Polynesia:Society Islands:Moorea | MNHN:Ich:BIOCODES331 | MBIO476.4 | JQ431487 |
| Blenniidae | *Blenniella periophthalmus* | Reunion:West:La Saline | ECOMAR:Ich:REU2569 | REU2569 | JQ349799 |
| Blenniidae | *Cirripectes castaneus* | Reunion:North:La possession | ECOMAR:Ich:REU0146 | REU0146 | JQ349906 |
| Blenniidae | *Cirripectes castaneus* | Reunion:South:St Philippe | ECOMAR:Ich:REU1763 | REU1763 | JQ349903 |
| Blenniidae | *Cirripectes castaneus* | Reunion:South:St Philippe | ECOMAR:Ich:REU1764 | REU1764 | JQ349902 |
| Blenniidae | *Cirripectes castaneus* | Reunion:South:St Philippe | ECOMAR:Ich:REU1843 | REU1843 | JQ349901 |
| Blenniidae | *Cirripectes castaneus* | Reunion:South:St Philippe | ECOMAR:Ich:REU1844 | REU1844 | JQ349900 |
| Blenniidae | *Cirripectes castaneus* | Reunion:West:La Saline | ECOMAR:Ich:REU2554 | REU2554 | JQ349904 |
| Blenniidae | *Cirripectes castaneus* | Reunion:West:La Saline | ECOMAR:Ich:REU2555 | REU2555 | JQ349905 |
| Blenniidae | *Cirripectes fuscoguttatus* | French Polynesia:Society Islands:Moorea | MNHN:Ich:BIOCODES0994 | MBIO1714.4 | JQ431646 |
| Blenniidae | *Cirripectes fuscoguttatus* | French Polynesia:Society Islands:Moorea | SI:Ich:MBIO1715 | MBIO1715.4 | JQ431645 |
| Blenniidae | *Cirripectes quagga* | French Polynesia:Society Islands:Moorea | MNHN:Ich:BIOCODES0201 | MBIO291.4 | JQ431648 |
| Blenniidae | *Cirripectes quagga* | French Polynesia:Society Islands:Moorea | MNHN:Ich:BIOCODES0202 | MBIO292.4 | JQ431647 |
| Blenniidae | *Cirripectes stigmaticus* | Reunion:West:St Leu | ECOMAR:Ich:REU1013 | REU1013 | JQ349910 |
| Blenniidae | *Cirripectes stigmaticus* | Reunion:West:St Gilles | ECOMAR:Ich:REU189-1 | REU189_1 | JQ349908 |
| Blenniidae | *Cirripectes stigmaticus* | Reunion:West:St Gilles | ECOMAR:Ich:REU189-2 | REU189_2 | JQ349907 |
| Blenniidae | *Cirripectes stigmaticus* | Reunion:West:La Saline | ECOMAR:Ich:REU2559 | REU2559 | JQ349909 |
| Blenniidae | *Cirripectes variolosus* | French Polynesia:Society Islands:Moorea | MNHN:Ich:BIOCODES0212 | MBIO307.4 | JQ431650 |
| Blenniidae | *Cirripectes variolosus* | French Polynesia:Society Islands:Moorea | MNHN:Ich:BIOCODES0213 | MBIO308.4 | JQ431649 |
| Blenniidae | *Ecsenius midas* | Madagascar:West:Nosy Be | ECOMAR:Ich:NBE1141 | NBE1141 | JQ349943 |
| Blenniidae | *Ecsenius midas* | Madagascar:West:Nosy Be | ECOMAR:Ich:NBE1198 | NBE1198 | JQ349942 |
| Blenniidae | *Ecsenius midas* | Madagascar:West:Nosy Be | ECOMAR:Ich:NBE1199 | NBE1199 | JQ349944 |
| Blenniidae | *Ecsenius midas* | Madagascar:West:Nosy Be | ECOMAR:Ich:NBE1202 | NBE1202 | JQ349945 |
| Blenniidae | *Ecsenius nalolo* | Madagascar:West:Nosy Be | ECOMAR:Ich:NBE0423 | NBE0423 | JQ349946 |
| Blenniidae | *Ecsenius nalolo* | Madagascar:West:Nosy Be | ECOMAR:Ich:NBE1154 | NBE1154 | JQ349948 |
| Blenniidae | *Ecsenius nalolo* | Madagascar:West:Nosy Be | ECOMAR:Ich:NBE1155 | NBE1155 | JQ349947 |
| Blenniidae | *Enchelyurus ater* | French Polynesia:Society Islands:Moorea | MNHN:Ich:BIOCODES0255 | MBIO375.4 | JQ431707 |
| Blenniidae | *Enchelyurus ater* | French Polynesia:Society Islands:Moorea | MNHN:Ich:BIOCODES0256 | MBIO376.4 | JQ431706 |
| Blenniidae | *Enchelyurus kraussii* | Reunion:West:St Gilles | ECOMAR:Ich:REU1678 | REU1678 | JQ349950 |
| Blenniidae | *Enchelyurus kraussii* | Reunion:West:St Gilles | ECOMAR:Ich:REU1679 | REU1679 | JQ349951 |
| Blenniidae | *Entomacrodus cymatobiotus* | French Polynesia:Society Islands:Moorea | MNHN:Ich:BIOCODES0986 | MBIO1685.4 | JQ431714 |
| Blenniidae | *Entomacrodus cymatobiotus* | French Polynesia:Society Islands:Moorea | MNHN:Ich:BIOCODES0987 | MBIO1686.4 | JQ431713 |
| Blenniidae | *Entomacrodus epalzeocheilos* | Reunion:South:St Philippe | ECOMAR:Ich:REU1766 | REU1766 | JQ349956 |
| Blenniidae | *Entomacrodus striatus* | Reunion:South:St Philippe | ECOMAR:Ich:REU1767 | REU1767 | JQ349960 |
| Blenniidae | *Entomacrodus striatus* | Reunion:South:St Philippe | ECOMAR:Ich:REU1768 | REU1768 | JQ349957 |
| Blenniidae | *Entomacrodus striatus* | Reunion:South:St Philippe | ECOMAR:Ich:REU1846 | REU1846 | JQ349958 |
| Blenniidae | *Entomacrodus striatus* | Reunion:South:St Philippe | ECOMAR:Ich:REU1847 | REU1847 | JQ349959 |
| Blenniidae | *Exallias brevis* | French Polynesia:Society Islands:Moorea | SI:Ich:MBIO1263 | MBIO1263.4 | JQ431739 |
| Blenniidae | *Exallias brevis* | French Polynesia:Society Islands:Moorea | MNHN:Ich:BIOCODES0218 | MBIO315.4 | JQ431737 |
| Blenniidae | *Exallias brevis* | French Polynesia:Society Islands:Moorea | MNHN:Ich:BIOCODES0219 | MBIO316.4 | JQ431738 |
| Blenniidae | *Exallias brevis* | Reunion:West:St Gilles | ECOMAR:Ich:REU190-1 | REU190_1 | JQ349976 |
| Blenniidae | *Exallias brevis* | Reunion:West:St Gilles | ECOMAR:Ich:REU190-2 | REU190_2 | JQ349974 |
| Blenniidae | *Exallias brevis* | Reunion:West:La Saline | ECOMAR:Ich:REU2551 | REU2551 | JQ349977 |
| Blenniidae | *Exallias brevis* | Reunion:West:La Saline | ECOMAR:Ich:REU2552 | REU2552 | JQ349975 |
| Blenniidae | *Glyptoparus delicatulus* | French Polynesia:Society Islands:Moorea | MNHN:Ich:BIOCODES0265 | MBIO384.4 | JQ431754 |
| Blenniidae | *Istiblennius bellus* | Reunion:West:St Gilles | ECOMAR:Ich:REU1680 | REU1680 | JQ350059 |
| Blenniidae | *Istiblennius bellus* | Reunion:South:St Philippe | ECOMAR:Ich:REU1692 | REU1692 | JQ350062 |
| Blenniidae | *Istiblennius bellus* | Reunion:South:St Philippe | ECOMAR:Ich:REU1765 | REU1765 | JQ350061 |
| Blenniidae | *Istiblennius bellus* | Reunion:South:St Philippe | ECOMAR:Ich:REU1845 | REU1845 | JQ350060 |
| Blenniidae | *Istiblennius dussumieri* | Madagascar:West:Nosy Be | ECOMAR:Ich:NBE1316 | NBE1316 | JQ350063 |
| Blenniidae | *Istiblennius dussumieri* | Madagascar:West:Nosy Be | ECOMAR:Ich:NBE1318 | NBE1318 | JQ350064 |
| Blenniidae | *Istiblennius edentulus* | Madagascar:West:Nosy Be | ECOMAR:Ich:NBE0227 | NBE0227 | JQ350065 |
| Blenniidae | *Istiblennius edentulus* | Madagascar:West:Nosy Be | ECOMAR:Ich:NBE0230 | NBE0230 | JQ350066 |
| Blenniidae | *Istiblennius spilotus* | Reunion:South:St Philippe | ECOMAR:Ich:REU1770 | REU1770 | JQ350067 |
| Blenniidae | *Meiacanthus mossambicus* | Madagascar:West:Nosy Be | ECOMAR:Ich:NBE0059 | NBE0059 | JQ350102 |
| Blenniidae | *Meiacanthus mossambicus* | Madagascar:West:Nosy Be | ECOMAR:Ich:NBE0126 | NBE0126 | JQ350101 |
| Blenniidae | *Meiacanthus mossambicus* | Madagascar:West:Nosy Be | ECOMAR:Ich:NBE0210 | NBE0210 | JQ350104 |
| Blenniidae | *Meiacanthus mossambicus* | Madagascar:West:Nosy Be | ECOMAR:Ich:NBE0583 | NBE0583 | JQ350103 |
| Blenniidae | *Nannosalarias nativitatis* | French Polynesia:Society Islands:Moorea | MNHN:Ich:BIOCODES0167 | MBIO238.4 | JQ431930 |
| Blenniidae | *Nannosalarias nativitatis* | French Polynesia:Society Islands:Moorea | SI:Ich:MBIO239 | MBIO239.4 | JQ431929 |
| Blenniidae | *Petroscirtes mitratus* | Madagascar:West:Nosy Be | ECOMAR:Ich:NBE0293 | NBE0293 | JQ350211 |
| Blenniidae | *Petroscirtes xestus* | French Polynesia:Society Islands:Moorea | MNHN:Ich:BIOCODES723 | MBIO1111.4 | JQ431997 |
| Blenniidae | *Petroscirtes xestus* | French Polynesia:Society Islands:Moorea | MNHN:Ich:BIOCODES0912 | MBIO1519.4 | JQ431996 |
| Blenniidae | *Petroscirtes xestus* | French Polynesia:Society Islands:Moorea | MNHN:Ich:BIOCODES0910 | MBIO1520.4 | JQ431995 |
| Blenniidae | *Plagiotremus rhinorhynchos* | Madagascar:West:Nosy Be | ECOMAR:Ich:NBE1168 | NBE1168 | JQ350213 |
| Blenniidae | *Plagiotremus rhinorhynchos* | Madagascar:West:Nosy Be | ECOMAR:Ich:NBE1169 | NBE1169 | JQ350212 |
| Blenniidae | *Plagiotremus rhinorhynchos* | Madagascar:West:Nosy Be | ECOMAR:Ich:NBE1170 | NBE1170 | JQ350214 |
| Blenniidae | *Plagiotremus tapeinosoma* | French Polynesia:Society Islands:Moorea | SI:Ich:MBIO1561 | MBIO1561.4 | JQ431998 |
| Blenniidae | *Plagiotremus tapeinosoma* | French Polynesia:Society Islands:Moorea | MNHN:Ich:BIOCODES652 | MBIO955.4 | JQ431999 |
| Blenniidae | *Plagiotremus tapeinosoma* | French Polynesia:Society Islands:Moorea | SI:Ich:MBIO956 | MBIO956.4 | JQ432000 |
| Blenniidae | *Plagiotremus tapeinosoma* | Madagascar:West:Nosy Be | ECOMAR:Ich:NBE0385 | NBE0385 | JQ350215 |
| Blenniidae | *Plagiotremus tapeinosoma* | Madagascar:West:Nosy Be | ECOMAR:Ich:NBE1134 | NBE1134 | JQ350216 |
| Blenniidae | *Stanulus seychellensis* | French Polynesia:Society Islands:Moorea | MNHN:Ich:BIOCODES0985 | MBIO1683.4 | JQ432161 |
| Blenniidae | *Stanulus seychellensis* | French Polynesia:Society Islands:Moorea | SI:Ich:MBIO1684 | MBIO1684.4 | JQ432160 |
| Bothidae | *Bothus mancus* | French Polynesia:Society Islands:Moorea | MNHN:Ich:BIOCODES788 | MBIO1230.4 | JQ431490 |
| Bothidae | *Bothus mancus* | French Polynesia:Society Islands:Moorea | SI:Ich:MBIO1387 | MBIO1387.4 | JQ431491 |
| Bothidae | *Bothus mancus* | French Polynesia:Society Islands:Moorea | MNHN:Ich:BIOCODES489 | MBIO725.4 | JQ431489 |
| Bothidae | *Bothus pantherinus* | French Polynesia:Society Islands:Moorea | MNHN:Ich:BIOCODES748 | MBIO1152.4 | JQ431492 |
| Bothidae | *Bothus pantherinus* | French Polynesia:Society Islands:Moorea | SI:Ich:MBIO834 | MBIO834.4 | JQ431493 |
| Bothidae | *Bothus pantherinus* | French Polynesia:Society Islands:Moorea | SI:Ich:MBIO912 | MBIO912.4 | JQ431494 |
| Bothidae | *Bothus pantherinus* | Madagascar:West:Nosy Be | ECOMAR:Ich:NBE0617 | NBE0617 | JQ349801 |
| Bothidae | *Bothus* sp. | French Polynesia:Society Islands:Moorea | MNHN:Ich:BIOCODES737 | MBIO1119.4 | JQ431495 |
| Bythitidae | *Brosmophyciops pautzkei* | French Polynesia:Society Islands:Moorea | MNHN:Ich:BIOCODES671 | MBIO993.4 | JQ431497 |
| Bythitidae | *Dinematichthys iluocoeteoides* | French Polynesia:Society Islands:Moorea | MNHN:Ich:BIOCODES0132 | MBIO192.4 | JQ431686 |
| Bythitidae | *Dinematichthys iluocoeteoides* | French Polynesia:Society Islands:Moorea | MNHN:Ich:BIOCODES0133 | MBIO193.4 | JQ431685 |
| Bythitidae | *Dinematichthys iluocoeteoides* | Madagascar:West:Nosy Be | ECOMAR:Ich:NBE0135 | NBE0135 | JQ349938 |
| Bythitidae | *Dinematichthys iluocoeteoides* | Madagascar:West:Nosy Be | ECOMAR:Ich:NBE0436 | NBE0436 | JQ349940 |
| Bythitidae | *Dinematichthys iluocoeteoides* | Madagascar:West:Nosy Be | ECOMAR:Ich:NBE0494 | NBE0494 | JQ349936 |
| Bythitidae | *Dinematichthys iluocoeteoides* | Reunion:West:St Leu | ECOMAR:Ich:REU1018 | REU1018 | JQ349939 |
| Bythitidae | *Dinematichthys iluocoeteoides* | Reunion:South:St Philippe | ECOMAR:Ich:REU1762 | REU1762 | JQ349937 |
| Bythitidae | *Dinematichthys iluocoeteoides* | Reunion:South:St Philippe | ECOMAR:Ich:REU1862 | REU1862 | JQ349941 |
| Caesionidae | *Caesio caerulaurea* | Madagascar:West:Nosy Be | ECOMAR:Ich:NBE0040 | NBE0040 | JQ349802 |
| Caesionidae | *Caesio caerulaurea* | Madagascar:West:Nosy Be | ECOMAR:Ich:NBE1190 | NBE1190 | JQ349803 |
| Caesionidae | *Caesio lunaris* | Madagascar:West:Nosy Be | ECOMAR:Ich:NBE0653 | NBE0653 | JQ349804 |
| Caesionidae | *Caesio xanthonota* | Madagascar:West:Nosy Be | ECOMAR:Ich:NBE1131 | NBE1131 | JQ349810 |
| Caesionidae | *Caesio xanthonota* | Madagascar:West:Nosy Be | ECOMAR:Ich:NBE1189 | NBE1189 | JQ349806 |
| Caesionidae | *Caesio xanthonota* | Madagascar:West:Nosy Be | ECOMAR:Ich:NBE1211 | NBE1211 | JQ349807 |
| Caesionidae | *Caesio xanthonota* | Madagascar:West:Nosy Be | ECOMAR:Ich:NBE1212 | NBE1212 | JQ349805 |
| Caesionidae | *Caesio xanthonota* | Madagascar:West:Nosy Be | ECOMAR:Ich:NBE1213 | NBE1213 | JQ349808 |
| Caesionidae | *Caesio xanthonota* | Madagascar:West:Nosy Be | ECOMAR:Ich:NBE1214 | NBE1214 | JQ349809 |
| Caesionidae | *Pterocaesio chrysozona* | Madagascar:West:Nosy Be | ECOMAR:Ich:NBE1177 | NBE1177 | JQ350291 |
| Caesionidae | *Pterocaesio chrysozona* | Madagascar:West:Nosy Be | ECOMAR:Ich:NBE1178 | NBE1178 | JQ350290 |
| Caesionidae | *Pterocaesio chrysozona* | Madagascar:West:Nosy Be | ECOMAR:Ich:NBE1180 | NBE1180 | JQ350289 |
| Caesionidae | *Pterocaesio tile* | French Polynesia:Society Islands:Moorea | SI:Ich:MBIO1254 | MBIO1254.4 | JQ432073 |
| Caesionidae | *Pterocaesio tile* | French Polynesia:Society Islands:Moorea | MNHN:Ich:BIOCODES645 | MBIO951.4 | JQ432072 |
| Caesionidae | *Pterocaesio tile* | Reunion:West:St Leu | ECOMAR:Ich:REU0917 | REU0917 | JQ350292 |
| Callionymidae | *Callionymus delicatulus* | Madagascar:West:Nosy Be | ECOMAR:Ich:NBE0570 | NBE0570 | JQ349812 |
| Callionymidae | *Callionymus delicatulus* | Madagascar:West:Nosy Be | ECOMAR:Ich:NBE0571 | NBE0571 | JQ349813 |
| Callionymidae | *Callionymus delicatulus* | Madagascar:West:Nosy Be | ECOMAR:Ich:NBE0572 | NBE0572 | JQ349811 |
| Callionymidae | *Callionymus simplicicornis* | French Polynesia:Society Islands:Moorea | MNHN:Ich:BIOCODES731 | MBIO1114.4 | JQ431510 |
| Callionymidae | *Callionymus simplicicornis* | French Polynesia:Society Islands:Moorea | MNHN:Ich:BIOCODES0913 | MBIO1509.5 | JQ431511 |
| Callionymidae | *Synchiropus ocellatus* | French Polynesia:Society Islands:Moorea | MNHN:Ich:BIOCODES0915 | MBIO1528.4 | JQ432180 |
| Caproidae | *Antigonia steindachneri* | French Polynesia:Society Islands:Moorea | MNHN:Ich:BIOCODES1053 | MBIO1865.4 | JQ431421 |
| Caracanthidae | *Caracanthus maculatus* | French Polynesia:Society Islands:Moorea | MNHN:Ich:BIOCODES0046 | MBIO82.4 | JQ431535 |
| Caracanthidae | *Caracanthus maculatus* | French Polynesia:Society Islands:Moorea | SI:Ich:MBIO83 | MBIO83.4 | JQ431534 |
| Caracanthidae | *Caracanthus madagascariensis* | Reunion:South:St Philippe | ECOMAR:Ich:REU1761 | REU1761 | JQ349839 |
| Caracanthidae | *Caracanthus unipinna* | French Polynesia:Society Islands:Moorea | MNHN:Ich:BIOCODES0850 | MBIO1357.4 | JQ431536 |
| Caracanthidae | *Caracanthus unipinna* | French Polynesia:Society Islands:Moorea | SI:Ich:MBIO1358 | MBIO1358.4 | JQ431537 |
| Caracanthidae | *Caracanthus unipinna* | Reunion:West:La Saline | ECOMAR:Ich:REU2590 | REU2590 | JQ349840 |
| Carangidae | *Carangoides ferdau* | French Polynesia:Society Islands:Moorea | MNHN:Ich:BIOCODES1030 | MBIO1835.4 | JQ431538 |
| Carangidae | *Carangoides orthogrammus* | French Polynesia:Society Islands:Moorea | MNHN:Ich:BIOCODES672 | MBIO1001.4 | JQ431539 |
| Carangidae | *Caranx ignobilis* | French Polynesia:Society Islands:Moorea | MNHN:Ich:BIOCODES1044 | MBIO1858.4 | JQ431540 |
| Carangidae | *Caranx lugubris* | French Polynesia:Society Islands:Moorea | MNHN:Ich:BIOCODES1054 | MBIO1866.4 | JQ431541 |
| Carangidae | *Caranx lugubris* | French Polynesia:Society Islands:Moorea | SI:Ich:MBIO1867 | MBIO1867.4 | JQ431542 |
| Carangidae | *Caranx melampygus* | French Polynesia:Society Islands:Moorea | MNHN:Ich:BIOCODES1047 | MBIO1862.4 | JQ431544 |
| Carangidae | *Caranx melampygus* | French Polynesia:Society Islands:Moorea | MNHN:Ich:BIOCODES0379 | MBIO538.4 | JQ431543 |
| Carangidae | *Caranx papuensis* | French Polynesia:Society Islands:Moorea | MNHN:Ich:BIOCODES1048 | MBIO1863.4 | JQ431545 |
| Carangidae | *Caranx papuensis* | Madagascar:West:Nosy Be | ECOMAR:Ich:NBE0008 | NBE0008 | JQ349841 |
| Carangidae | *Caranx sexfasciatus* | French Polynesia:Society Islands:Moorea | MNHN:Ich:BIOCODES764 | MBIO1191.4 | JQ431546 |
| Carangidae | *Caranx sexfasciatus* | French Polynesia:Society Islands:Moorea | MNHN:Ich:BIOCODES1046 | MBIO1860.4 | JQ431550 |
| Carangidae | *Caranx sexfasciatus* | French Polynesia:Society Islands:Moorea | SI:Ich:MBIO1861 | MBIO1861.4 | JQ431547 |
| Carangidae | *Caranx sexfasciatus* | French Polynesia:Society Islands:Moorea | MNHN:Ich:BIOCODES564 | MBIO835.4 | JQ431549 |
| Carangidae | *Caranx sexfasciatus* | French Polynesia:Society Islands:Moorea | MNHN:Ich:BIOCODES567 | MBIO836.4 | JQ431548 |
| Carangidae | *Decapterus macarellus* | French Polynesia:Society Islands:Moorea | MNHN:Ich:BIOCODES0859 | MBIO1408.4 | JQ431682 |
| Carangidae | *Decapterus macarellus* | French Polynesia:Society Islands:Moorea | MNHN:Ich:BIOCODES0860 | MBIO1409.4 | JQ431681 |
| Carangidae | *Elagatis bipinnulata* | French Polynesia:Society Islands:Moorea | MNHN:Ich:BIOCODES825 | MBIO1297.4 | JQ431698 |
| Carangidae | *Scomberoides lysan* | French Polynesia:Society Islands:Moorea | MNHN:Ich:BIOCODES1045 | MBIO1859.4 | JQ432119 |
| Carangidae | *Seriola rivoliana* | French Polynesia:Society Islands:Moorea | MNHN:Ich:BIOCODES0993 | MBIO1706.4 | JQ432150 |
| Carangidae | *Seriola rivoliana* | French Polynesia:Society Islands:Moorea | SI:Ich:MBIO1707 | MBIO1707.4 | JQ432149 |
| Carangidae | *Trachinotus baillonii* | French Polynesia:Society Islands:Moorea | MNHN:Ich:BIOCODES813 | MBIO1276.4 | JQ432196 |
| Carangidae | *Trachinotus baillonii* | French Polynesia:Society Islands:Moorea | MNHN:Ich:MBIO1437 | MBIO1437.4 | JQ432197 |
| Carangidae | *Trachurus delagoa* | Madagascar:West:Nosy Be | ECOMAR:Ich:NBE1089 | NBE1089 | JQ350400 |
| Carapidae | *Carapus mourlani* | French Polynesia:Society Islands:Moorea | MNHN:Ich:BIOCODES0900 | MBIO1492.4 | JQ431552 |
| Carapidae | *Carapus mourlani* | French Polynesia:Society Islands:Moorea | SI:Ich:MBIO1493 | MBIO1493.4 | JQ431551 |
| Carapidae | *Carapus* sp. | Madagascar:West:Nosy Be | ECOMAR:Ich:NBE1262 | NBE1262 | JQ349842 |
| Carcharhinidae | *Carcharhinus melanopterus* | French Polynesia:Society Islands:Moorea | UPVD:Ich:MBIO1873 | MBIO1873.4 | JQ431553 |
| Centropomidae | *Lates calcarifer* | French Polynesia:Society Islands:Moorea | MNHN:Ich:BIOCODES721 | MBIO1144.4 | JQ431879 |
| Chaetodontidae | *Chaetodon auriga* | French Polynesia:Society Islands:Moorea | MNHN:Ich:BIOCODES504 | MBIO742.4 | JQ431581 |
| Chaetodontidae | *Chaetodon auriga* | Madagascar:West:Nosy Be | ECOMAR:Ich:NBE0211 | NBE0211 | JF434764 |
| Chaetodontidae | *Chaetodon auriga* | Reunion:West:Hermitage | ECOMAR:Ich:REU0112 | REU0112 | JF434763 |
| Chaetodontidae | *Chaetodon auriga* | Reunion:West:St Leu | ECOMAR:Ich:REU0903 | REU0903 | JF434765 |
| Chaetodontidae | *Chaetodon auriga* | Reunion:West:Hermitage | ECOMAR:Ich:REU151-1 | REU151-1 | JF434762 |
| Chaetodontidae | *Chaetodon auriga* | Reunion:West:Hermitage | ECOMAR:Ich:REU301-1 | REU301-1 | JF434761 |
| Chaetodontidae | *Chaetodon bennetti* | French Polynesia:Society Islands:Moorea | MNHN:Ich:2008-972 | MBIO1310 | JF434768 |
| Chaetodontidae | *Chaetodon bennetti* | Madagascar:West:Nosy Be | ECOMAR:Ich:NBE0453 | NBE0453 | JF434767 |
| Chaetodontidae | *Chaetodon bennetti* | Madagascar:West:Nosy Be | ECOMAR:Ich:NBE0454 | NBE0454 | JF434766 |
| Chaetodontidae | *Chaetodon blackburnii* | Reunion:West:Hermitage | ECOMAR:Ich:REU0754 | REU0754 | JF434770 |
| Chaetodontidae | *Chaetodon blackburnii* | Reunion:West:Hermitage | ECOMAR:Ich:REU0755 | REU0755 | JF434769 |
| Chaetodontidae | *Chaetodon citrinellus* | French Polynesia:Society Islands:Moorea | MNHN:Ich:2008-502 | MBIO0513 | JF434772 |
| Chaetodontidae | *Chaetodon citrinellus* | French Polynesia:Society Islands:Moorea | MNHN:Ich:2008-722 | MBIO0856 | JF434771 |
| Chaetodontidae | *Chaetodon citrinellus* | French Polynesia:Society Islands:Moorea | MNHN:Ich:BIOCODES0359 | MBIO514.4 | JQ431583 |
| Chaetodontidae | *Chaetodon citrinellus* | French Polynesia:Society Islands:Moorea | MNHN:Ich:BIOCODES579 | MBIO855.4 | JQ431582 |
| Chaetodontidae | *Chaetodon ephippium* | French Polynesia:Society Islands:Moorea | MNHN:Ich:BIOCODES806 | MBIO1260.4 | JQ431584 |
| Chaetodontidae | *Chaetodon ephippium* | French Polynesia:Society Islands:Moorea | MNHN:Ich:MBIO1261 | MBIO1261 | JF434773 |
| Chaetodontidae | *Chaetodon falcula* | Madagascar:West:Nosy Be | ECOMAR:Ich:NBE0042 | NBE0042 | JF434776 |
| Chaetodontidae | *Chaetodon falcula* | Madagascar:West:Nosy Be | ECOMAR:Ich:NBE0455 | NBE0455 | JF434775 |
| Chaetodontidae | *Chaetodon falcula* | Madagascar:West:Nosy Be | ECOMAR:Ich:NBE2035 | NBE2035 | JF434774 |
| Chaetodontidae | *Chaetodon guttatissimus* | Madagascar:West:Nosy Be | ECOMAR:Ich:NBE0354 | NBE0354 | JF434782 |
| Chaetodontidae | *Chaetodon guttatissimus* | Madagascar:West:Nosy Be | ECOMAR:Ich:NBE0355 | NBE0355 | JF434781 |
| Chaetodontidae | *Chaetodon guttatissimus* | Reunion:West:Hermitage | ECOMAR:Ich:REU0502 | REU0502 | JF434780 |
| Chaetodontidae | *Chaetodon guttatissimus* | Reunion:West:Hermitage | ECOMAR:Ich:REU0759 | REU0759 | JF434779 |
| Chaetodontidae | *Chaetodon guttatissimus* | Reunion:West:Hermitage | ECOMAR:Ich:REU102-1 | REU102-1 | JF434778 |
| Chaetodontidae | *Chaetodon guttatissimus* | Reunion:West:Hermitage | ECOMAR:Ich:REU213-1 | REU213-1 | JF434777 |
| Chaetodontidae | *Chaetodon kleinii* | Madagascar:West:Nosy Be | ECOMAR:Ich:NBE0359 | NBE0359 | JF434785 |
| Chaetodontidae | *Chaetodon kleinii* | Reunion:West:Hermitage | ECOMAR:Ich:REU0758 | REU0758 | JF434784 |
| Chaetodontidae | *Chaetodon kleinii* | Reunion:West:St Leu | ECOMAR:Ich:REU0940 | REU0940 | JF434786 |
| Chaetodontidae | *Chaetodon kleinii* | Reunion:West:Hermitage | ECOMAR:Ich:REU104-1 | REU104-1 | JF434783 |
| Chaetodontidae | *Chaetodon kleinii* | Reunion:West:St Leu | ECOMAR:Ich:REU1694 | REU1694 | JQ349876 |
| Chaetodontidae | *Chaetodon kleinii* | Reunion:West:St Gilles | ECOMAR:Ich:REU334-1 | REU334_1 | JQ349877 |
| Chaetodontidae | *Chaetodon lunula* | French Polynesia:Society Islands:Moorea | MNHN:Ich:BIOCODES0361 | MBIO517.4 | JQ431585 |
| Chaetodontidae | *Chaetodon lunula* | French Polynesia:Society Islands:Moorea | SI:Ich:MBIO518 | MBIO518.4 | JQ431586 |
| Chaetodontidae | *Chaetodon lunula* | Madagascar:West:Nosy Be | ECOMAR:Ich:NBE0347 | NBE0347 | JF434792 |
| Chaetodontidae | *Chaetodon lunula* | Madagascar:West:Nosy Be | ECOMAR:Ich:NBE0654 | NBE0654 | JF434791 |
| Chaetodontidae | *Chaetodon lunula* | Reunion:West:Hermitage | ECOMAR:Ich:REU0501 | REU0501 | JF434790 |
| Chaetodontidae | *Chaetodon lunula* | Reunion:West:Hermitage | ECOMAR:Ich:REU0721 | REU0721 | JF434789 |
| Chaetodontidae | *Chaetodon lunula* | Reunion:West:St Leu | ECOMAR:Ich:REU1611 | REU1611 | JF434788 |
| Chaetodontidae | *Chaetodon lunula* | Reunion:West:Hermitage | ECOMAR:Ich:REU209-1 | REU209-1 | JF434787 |
| Chaetodontidae | *Chaetodon lunulatus* | French Polynesia:Society Islands:Moorea | MNHN:Ich:2008-506 | MBIO0519 | JF434794 |
| Chaetodontidae | *Chaetodon lunulatus* | French Polynesia:Society Islands:Moorea | MNHN:Ich:MBIO520 | MBIO0520 | JF434793 |
| Chaetodontidae | *Chaetodon lunulatus* | French Polynesia:Society Islands:Moorea | MNHN:Ich:BIOCODES0357 | MBIO519.4 | JQ431587 |
| Chaetodontidae | *Chaetodon lunulatus* | French Polynesia:Society Islands:Moorea | SI:Ich:MBIO520 | MBIO520.4 | JQ431588 |
| Chaetodontidae | *Chaetodon melannotus* | Madagascar:West:Nosy Be | ECOMAR:Ich:NBE0212 | NBE0212 | JF434800 |
| Chaetodontidae | *Chaetodon melannotus* | Madagascar:West:Nosy Be | ECOMAR:Ich:NBE0352 | NBE0352 | JF434799 |
| Chaetodontidae | *Chaetodon melannotus* | Reunion:West:Hermitage | ECOMAR:Ich:REU0113 | REU0113 | JF434798 |
| Chaetodontidae | *Chaetodon melannotus* | Reunion:West:Hermitage | ECOMAR:Ich:REU0114 | REU0114 | JF434797 |
| Chaetodontidae | *Chaetodon melannotus* | Reunion:West:Hermitage | ECOMAR:Ich:REU0294 | REU0294 | JF434796 |
| Chaetodontidae | *Chaetodon melannotus* | Reunion:West:Hermitage | ECOMAR:Ich:REU211-1 | REU211-1 | JF434795 |
| Chaetodontidae | *Chaetodon mertensii* | French Polynesia:Society Islands:Moorea | MNHN:Ich:2008-598 | MBIO0663 | JF434802 |
| Chaetodontidae | *Chaetodon mertensii* | French Polynesia:Society Islands:Moorea | MNHN:Ich:MBIO664 | MBIO0664 | JF434801 |
| Chaetodontidae | *Chaetodon meyeri* | Madagascar:West:Nosy Be | ECOMAR:Ich:NBE0350 | NBE0350 | JF434804 |
| Chaetodontidae | *Chaetodon meyeri* | Reunion:West:Hermitage | ECOMAR:Ich:REU0790 | REU0790 | JF434806 |
| Chaetodontidae | *Chaetodon meyeri* | Reunion:West:St Leu | ECOMAR:Ich:REU0904 | REU0904 | JF434805 |
| Chaetodontidae | *Chaetodon meyeri* | Reunion:West:St Leu | ECOMAR:Ich:REU1614 | REU1614 | JF434803 |
| Chaetodontidae | *Chaetodon ornatissimus* | French Polynesia:Society Islands:Moorea | MNHN:Ich:2008-328 | MBIO0257 | JF434807 |
| Chaetodontidae | *Chaetodon pelewensis* | French Polynesia:Society Islands:Moorea | MNHN:Ich:2008-198 | MBIO0084 | JF434809 |
| Chaetodontidae | *Chaetodon pelewensis* | French Polynesia:Society Islands:Moorea | MNHN:Ich:2008-199 | MBIO0085 | JF434808 |
| Chaetodontidae | *Chaetodon quadrimaculatus* | French Polynesia:Society Islands:Moorea | MNHN:Ich:2008-778 | MBIO0940 | JF434811 |
| Chaetodontidae | *Chaetodon quadrimaculatus* | French Polynesia:Society Islands:Moorea | MNHN:Ich:2008-779 | MBIO0941 | JF434810 |
| Chaetodontidae | *Chaetodon reticulatus* | French Polynesia:Society Islands:Moorea | MNHN:Ich:2008-174 | MBIO0049 | JF434813 |
| Chaetodontidae | *Chaetodon reticulatus* | French Polynesia:Society Islands:Moorea | MNHN:Ich:2008-716 | MBIO0847 | JF434812 |
| Chaetodontidae | *Chaetodon reticulatus* | French Polynesia:Society Islands:Moorea | MNHN:Ich:BIOCODES0024 | MBIO50.4 | JQ431589 |
| Chaetodontidae | *Chaetodon trichrous* | French Polynesia:Society Islands:Moorea | MNHN:Ich:2008-611 | MBIO0683 | JF434815 |
| Chaetodontidae | *Chaetodon trichrous* | French Polynesia:Society Islands:Moorea | MNHN:Ich:2008-748 | MBIO0898 | JF434814 |
| Chaetodontidae | *Chaetodon trichrous* | French Polynesia:Society Islands:Moorea | MNHN:Ich:BIOCODES0469 | MBIO684.4 | JQ431590 |
| Chaetodontidae | *Chaetodon trifascialis* | French Polynesia:Society Islands:Moorea | SI:Ich:MBIO1251 | MBIO1251.4 | JQ431592 |
| Chaetodontidae | *Chaetodon trifascialis* | French Polynesia:Society Islands:Moorea | MNHN:Ich:BIOCODES644 | MBIO944.4 | JQ431591 |
| Chaetodontidae | *Chaetodon trifascialis* | Madagascar:West:Nosy Be | ECOMAR:Ich:NBE0643 | NBE0643 | JF434822 |
| Chaetodontidae | *Chaetodon trifascialis* | Madagascar:West:Nosy Be | ECOMAR:Ich:NBE0644 | NBE0644 | JF434821 |
| Chaetodontidae | *Chaetodon trifascialis* | Madagascar:West:Nosy Be | ECOMAR:Ich:NBE1292 | NBE1292 | JF434820 |
| Chaetodontidae | *Chaetodon trifascialis* | Reunion:West:Hermitage | ECOMAR:Ich:REU0745 | REU0745 | JF434819 |
| Chaetodontidae | *Chaetodon trifascialis* | Reunion:West:Hermitage | ECOMAR:Ich:REU0746 | REU0746 | JF434818 |
| Chaetodontidae | *Chaetodon trifascialis* | Reunion:West:Hermitage | ECOMAR:Ich:REU103-1 | REU103-1 | JF434817 |
| Chaetodontidae | *Chaetodon trifascialis* | Reunion:West:Hermitage | ECOMAR:Ich:REU186-1 | REU186-1 | JF434816 |
| Chaetodontidae | *Chaetodon trifasciatus* | Madagascar:West:Nosy Be | ECOMAR:Ich:NBE0045 | NBE0045 | JF434830 |
| Chaetodontidae | *Chaetodon trifasciatus* | Madagascar:West:Nosy Be | ECOMAR:Ich:NBE0046 | NBE0046 | JF434829 |
| Chaetodontidae | *Chaetodon trifasciatus* | Madagascar:West:Nosy Be | ECOMAR:Ich:NBE0573 | NBE0573 | JF434827 |
| Chaetodontidae | *Chaetodon trifasciatus* | Madagascar:West:Nosy Be | ECOMAR:Ich:NBE1010 | NBE1010 | JF434826 |
| Chaetodontidae | *Chaetodon trifasciatus* | Reunion:West:Hermitage | ECOMAR:Ich:REU0718 | REU0718 | JF434825 |
| Chaetodontidae | *Chaetodon trifasciatus* | Reunion:West:Hermitage | ECOMAR:Ich:REU0719 | REU0719 | JF434824 |
| Chaetodontidae | *Chaetodon trifasciatus* | Reunion:West:Hermitage | ECOMAR:Ich:REU0720 | REU0720 | JF434823 |
| Chaetodontidae | *Chaetodon trifasciatus* | Reunion:West:St Leu | ECOMAR:Ich:REU0942 | REU0942 | JF434828 |
| Chaetodontidae | *Chaetodon ulietensis* | French Polynesia:Society Islands:Moorea | MNHN:Ich:2008-958 | MBIO1286 | JF434832 |
| Chaetodontidae | *Chaetodon ulietensis* | French Polynesia:Society Islands:Moorea | MNHN:Ich:MBIO1295 | MBIO1295 | JF434831 |
| Chaetodontidae | *Chaetodon unimaculatus* | French Polynesia:Society Islands:Moorea | MNHN:Ich:2008-508 | MBIO0522 | JF434836 |
| Chaetodontidae | *Chaetodon unimaculatus* | French Polynesia:Society Islands:Moorea | MNHN:Ich:BIOCODES0362 | MBIO523.4 | JQ431593 |
| Chaetodontidae | *Chaetodon unimaculatus* | French Polynesia:Society Islands:Moorea | MNHN:Ich:BIOCODES538 | MBIO794.4 | JQ431594 |
| Chaetodontidae | *Chaetodon unimaculatus* | Reunion:West:Hermitage | ECOMAR:Ich:REU0760 | REU0760 | JF434835 |
| Chaetodontidae | *Chaetodon unimaculatus* | Reunion:West:Hermitage | ECOMAR:Ich:REU185-1 | REU185-1 | JF434834 |
| Chaetodontidae | *Chaetodon unimaculatus* | Reunion:West:St Leu | ECOMAR:Ich:REU2631 | REU2631 | JF434833 |
| Chaetodontidae | *Chaetodon vagabundus* | French Polynesia:Society Islands:Moorea | MNHN:Ich:BIOCODES0363 | MBIO521.4 | JQ431595 |
| Chaetodontidae | *Chaetodon vagabundus* | French Polynesia:Society Islands:Moorea | SI:Ich:MBIO801 | MBIO801.4 | JQ431597 |
| Chaetodontidae | *Chaetodon vagabundus* | French Polynesia:Society Islands:Moorea | MNHN:Ich:BIOCODES609 | MBIO897.5 | JQ431596 |
| Chaetodontidae | *Chaetodon vagabundus* | Madagascar:West:Nosy Be | ECOMAR:Ich:NBE0348 | NBE0348 | JF434842 |
| Chaetodontidae | *Chaetodon vagabundus* | Madagascar:West:Nosy Be | ECOMAR:Ich:NBE0349 | NBE0349 | JF434841 |
| Chaetodontidae | *Chaetodon vagabundus* | Reunion:West:Hermitage | ECOMAR:Ich:REU0772 | REU0772 | JF434840 |
| Chaetodontidae | *Chaetodon vagabundus* | Reunion:West:Hermitage | ECOMAR:Ich:REU153-1 | REU153-1 | JF434839 |
| Chaetodontidae | *Chaetodon vagabundus* | Reunion:West:Hermitage | ECOMAR:Ich:REU212-1 | REU212-1 | JF434838 |
| Chaetodontidae | *Chaetodon vagabundus* | Reunion:West:Hermitage | ECOMAR:Ich:REU277-1 | REU277-1 | JF434837 |
| Chaetodontidae | *Chaetodon xanthurus* | Reunion:West:Hermitage | ECOMAR:Ich:REU0748 | REU0748 | JF434845 |
| Chaetodontidae | *Chaetodon xanthurus* | Reunion:West:Hermitage | ECOMAR:Ich:REU0749 | REU0749 | JF434844 |
| Chaetodontidae | *Chaetodon xanthurus* | Reunion:West:St Leu | ECOMAR:Ich:REU1613 | REU1613 | JF434843 |
| Chaetodontidae | *Chaetodon zanzibarensis* | Madagascar:West:Nosy Be | ECOMAR:Ich:BMADA0046 | BMADA0046 | JF434846 |
| Chaetodontidae | *Forcipiger flavissimus* | French Polynesia:Society Islands:Moorea | MNHN:Ich:BIOCODES0183 | MBIO258.4 | JQ431742 |
| Chaetodontidae | *Forcipiger flavissimus* | French Polynesia:Society Islands:Moorea | SI:Ich:MBIO653 | MBIO653.4 | JQ431743 |
| Chaetodontidae | *Forcipiger flavissimus* | French Polynesia:Society Islands:Moorea | MNHN:Ich:BIOCODES587 | MBIO867.4 | JQ431744 |
| Chaetodontidae | *Forcipiger flavissimus* | Reunion:West:Hermitage | ECOMAR:Ich:REU0737 | REU0737 | JF434973 |
| Chaetodontidae | *Forcipiger flavissimus* | Reunion:West:Hermitage | ECOMAR:Ich:REU0738 | REU0738 | JF434972 |
| Chaetodontidae | *Forcipiger flavissimus* | Reunion:West:Hermitage | ECOMAR:Ich:REU0756 | REU0756 | JF434971 |
| Chaetodontidae | *Forcipiger flavissimus* | Reunion:West:St Leu | ECOMAR:Ich:REU1695 | REU1695 | JF434970 |
| Chaetodontidae | *Forcipiger longirostris* | French Polynesia:Society Islands:Moorea | MNHN:Ich:2008-768 | MBIO0927 | JF434975 |
| Chaetodontidae | *Forcipiger longirostris* | French Polynesia:Society Islands:Moorea | MNHN:Ich:MBIO928 | MBIO0928 | JF434974 |
| Chaetodontidae | *Forcipiger longirostris* | French Polynesia:Society Islands:Moorea | MNHN:Ich:BIOCODES0888 | MBIO1475.4 | JQ431745 |
| Chaetodontidae | *Hemitaurichthys polylepis* | French Polynesia:Society Islands:Moorea | MNHN:Ich:2008-775 | MBIO0936 | JF435020 |
| Chaetodontidae | *Hemitaurichthys polylepis* | French Polynesia:Society Islands:Moorea | MNHN:Ich:2008-776 | MBIO0937 | JF435019 |
| Chaetodontidae | *Hemitaurichthys thompsoni* | French Polynesia:Society Islands:Moorea | MNHN:Ich:2008-988 | MBIO1378 | JF435022 |
| Chaetodontidae | *Hemitaurichthys thompsoni* | French Polynesia:Society Islands:Moorea | MNHN:Ich:MBIO1379 | MBIO1379 | JF435021 |
| Chaetodontidae | *Hemitaurichthys zoster* | Reunion:West:Hermitage | ECOMAR:Ich:REU0791 | REU0791 | JF435024 |
| Chaetodontidae | *Hemitaurichthys zoster* | Reunion:West:Hermitage | ECOMAR:Ich:REU0792 | REU0792 | JF435023 |
| Chaetodontidae | *Heniochus acuminatus* | French Polynesia:Society Islands:Moorea | MNHN:Ich:BIOCODES746 | MBIO1148.4 | JQ431852 |
| Chaetodontidae | *Heniochus acuminatus* | French Polynesia:Society Islands:Moorea | SI:Ich:MBIO1149 | MBIO1149.4 | JQ431850 |
| Chaetodontidae | *Heniochus acuminatus* | French Polynesia:Society Islands:Moorea | MNHN:Ich:BIOCODES0426 | MBIO607.4 | JQ431851 |
| Chaetodontidae | *Heniochus acuminatus* | Reunion:West:Hermitage | ECOMAR:Ich:REU0730 | REU0730 | JF435025 |
| Chaetodontidae | *Heniochus chrysostomus* | French Polynesia:Society Islands:Moorea | MNHN:Ich:2008-511 | MBIO0526 | JF435027 |
| Chaetodontidae | *Heniochus chrysostomus* | French Polynesia:Society Islands:Moorea | MNHN:Ich:2008-512 | MBIO0527 | JF435026 |
| Chaetodontidae | *Heniochus chrysostomus* | French Polynesia:Society Islands:Moorea | MNHN:Ich:BIOCODES484 | MBIO723.4 | JQ431854 |
| Chaetodontidae | *Heniochus chrysostomus* | French Polynesia:Society Islands:Moorea | MNHN:Ich:BIOCODES488 | MBIO724.4 | JQ431853 |
| Chaetodontidae | *Heniochus diphreutes* | Madagascar:West:Nosy Be | ECOMAR:Ich:NBE0041 | NBE0041 | JF435031 |
| Chaetodontidae | *Heniochus diphreutes* | Madagascar:West:Nosy Be | ECOMAR:Ich:NBE0180 | NBE0180 | JF435030 |
| Chaetodontidae | *Heniochus diphreutes* | Madagascar:West:Nosy Be | ECOMAR:Ich:NBE0616 | NBE0616 | JF435029 |
| Chaetodontidae | *Heniochus diphreutes* | Reunion:West:Hermitage | ECOMAR:Ich:REU0729 | REU0729 | JF435028 |
| Chaetodontidae | *Heniochus monoceros* | French Polynesia:Society Islands:Moorea | MNHN:Ich:BIOCODES747 | MBIO1150.4 | JQ431855 |
| Chaetodontidae | *Heniochus monoceros* | French Polynesia:Society Islands:Moorea | MNHN:Ich:BIOCODES800 | MBIO1248.4 | JQ431856 |
| Chaetodontidae | *Heniochus monoceros* | Reunion:West:St Leu | ECOMAR:Ich:REU0905 | REU0905 | JF435035 |
| Chaetodontidae | *Heniochus monoceros* | Reunion:West:St Leu | ECOMAR:Ich:REU0906 | REU0906 | JF435034 |
| Chaetodontidae | *Heniochus monoceros* | Reunion:West:Hermitage | ECOMAR:Ich:REU152-1 | REU152-1 | JF435033 |
| Chaetodontidae | *Heniochus monoceros* | Reunion:West:Hermitage | ECOMAR:Ich:REU276-1 | REU276-1 | JF435032 |
| Chanidae | *Chanos chanos* | French Polynesia:Society Islands:Moorea | MNHN:Ich:BIOCODES1021 | MBIO1822.4 | JQ431601 |
| Chanidae | *Chanos chanos* | French Polynesia:Society Islands:Moorea | MNHN:Ich:BIOCODES1022 | MBIO1823.4 | JQ431600 |
| Chlopsidae | *Kaupichthys diodontus* | French Polynesia:Society Islands:Moorea | MNHN:Ich:BIOCODES0080 | MBIO127.4 | JQ431869 |
| Chlopsidae | *Kaupichthys diodontus* | French Polynesia:Society Islands:Moorea | MNHN:Ich:BIOCODES0927 | MBIO1567.4 | JQ431867 |
| Chlopsidae | *Kaupichthys diodontus* | French Polynesia:Society Islands:Moorea | SI:Ich:MBIO1643 | MBIO1643.4 | JQ431868 |
| Chlopsidae | *Kaupichthys diodontus* | Madagascar:West:Nosy Be | ECOMAR:Ich:NBE0591 | NBE0591 | JQ350070 |
| Chlopsidae | *Kaupichthys diodontus* | Madagascar:West:Nosy Be | ECOMAR:Ich:NBE0592 | NBE0592 | JQ350068 |
| Chlopsidae | *Kaupichthys diodontus* | Madagascar:West:Nosy Be | ECOMAR:Ich:NBE0593 | NBE0593 | JQ350069 |
| Chlopsidae | *Kaupichthys diodontus* | Reunion:South:St Philippe | ECOMAR:Ich:REU1787 | REU1787 | JQ350071 |
| Cichlidae | *Oreochromis mossambicus* | French Polynesia:Society Islands:Moorea | MNHN:Ich:BIOCODES1023 | MBIO1826.4 | JQ431947 |
| Cichlidae | *Oreochromis mossambicus* | French Polynesia:Society Islands:Moorea | MNHN:Ich:BIOCODES1024 | MBIO1827.4 | JQ431946 |
| Cirrhitidae | *Amblycirrhitus bimacula* | French Polynesia:Society Islands:Moorea | MNHN:Ich:BIOCODES0858 | MBIO1407.4 | JQ431404 |
| Cirrhitidae | *Amblycirrhitus bimacula* | Reunion:West:St Leu | ECOMAR:Ich:REU1006 | REU1006 | JQ349679 |
| Cirrhitidae | *Amblycirrhitus bimacula* | Reunion:West:St Leu | ECOMAR:Ich:REU1007 | REU1007 | JQ349680 |
| Cirrhitidae | *Cirrhitichthys oxycephalus* | Madagascar:West:Nosy Be | ECOMAR:Ich:NBE0426 | NBE0426 | JQ349894 |
| Cirrhitidae | *Cirrhitichthys oxycephalus* | Madagascar:West:Nosy Be | ECOMAR:Ich:NBE0427 | NBE0427 | JQ349895 |
| Cirrhitidae | *Cirrhitichthys oxycephalus* | Madagascar:West:Nosy Be | ECOMAR:Ich:NBE1208 | NBE1208 | JQ349897 |
| Cirrhitidae | *Cirrhitichthys oxycephalus* | Madagascar:West:Nosy Be | ECOMAR:Ich:NBE1209 | NBE1209 | JQ349896 |
| Cirrhitidae | *Cirrhitops fasciatus* | Reunion:West:St Gilles | ECOMAR:Ich:REU0752 | REU0752 | JQ349898 |
| Cirrhitidae | *Cirrhitops fasciatus* | Reunion:West:St Leu | ECOMAR:Ich:REU1008 | REU1008 | JQ349899 |
| Cirrhitidae | *Cirrhitus pinnulatus* | French Polynesia:Society Islands:Moorea | MNHN:Ich:BIOCODES0981 | MBIO1663.4 | JQ431642 |
| Cirrhitidae | *Cirrhitus pinnulatus* | French Polynesia:Society Islands:Moorea | MNHN:Ich:BIOCODES0982 | MBIO1664.4 | JQ431643 |
| Cirrhitidae | *Cirrhitus pinnulatus* | French Polynesia:Society Islands:Moorea | MNHN:Ich:BIOCODES0216 | MBIO311.4 | JQ431641 |
| Cirrhitidae | *Cirrhitus pinnulatus* | French Polynesia:Society Islands:Moorea | MNHN:Ich:BIOCODES0215 | MBIO312.4 | JQ431644 |
| Cirrhitidae | *Neocirrhites armatus* | French Polynesia:Society Islands:Moorea | MNHN:Ich:BIOCODES0007 | MBIO34.4 | JQ431933 |
| Cirrhitidae | *Paracirrhites arcatus* | French Polynesia:Society Islands:Moorea | MNHN:Ich:BIOCODES0001 | MBIO21.4 | JQ431958 |
| Cirrhitidae | *Paracirrhites arcatus* | French Polynesia:Society Islands:Moorea | MNHN:Ich:BIOCODES0002 | MBIO22.4 | JQ431955 |
| Cirrhitidae | *Paracirrhites arcatus* | French Polynesia:Society Islands:Moorea | MNHN:Ich:BIOCODES531 | MBIO782.4 | JQ431956 |
| Cirrhitidae | *Paracirrhites arcatus* | French Polynesia:Society Islands:Moorea | SI:Ich:MBIO783 | MBIO783.4 | JQ431957 |
| Cirrhitidae | *Paracirrhites arcatus* | Madagascar:West:Nosy Be | ECOMAR:Ich:NBE1136 | NBE1136 | JQ350165 |
| Cirrhitidae | *Paracirrhites arcatus* | Madagascar:West:Nosy Be | ECOMAR:Ich:NBE1230 | NBE1230 | JQ350167 |
| Cirrhitidae | *Paracirrhites arcatus* | Reunion:West:St Gilles | ECOMAR:Ich:REU0771 | REU0771 | JQ350168 |
| Cirrhitidae | *Paracirrhites arcatus* | Reunion:West:St Leu | ECOMAR:Ich:REU0914 | REU0914 | JQ350166 |
| Cirrhitidae | *Paracirrhites arcatus* | Reunion:West:St Leu | ECOMAR:Ich:REU1001 | REU1001 | JQ350164 |
| Cirrhitidae | *Paracirrhites forsteri* | French Polynesia:Society Islands:Moorea | MNHN:Ich:BIOCODES0032 | MBIO61.4 | JQ431959 |
| Cirrhitidae | *Paracirrhites forsteri* | French Polynesia:Society Islands:Moorea | MNHN:Ich:BIOCODES0058 | MBIO99.4 | JQ431960 |
| Cirrhitidae | *Paracirrhites forsteri* | Madagascar:West:Nosy Be | ECOMAR:Ich:NBE1126 | NBE1126 | JQ350169 |
| Cirrhitidae | *Paracirrhites forsteri* | Madagascar:West:Nosy Be | ECOMAR:Ich:NBE1137 | NBE1137 | JQ350171 |
| Cirrhitidae | *Paracirrhites forsteri* | Reunion:West:St Gilles | ECOMAR:Ich:REU0773 | REU0773 | JQ350170 |
| Cirrhitidae | *Paracirrhites hemistictus* | French Polynesia:Society Islands:Moorea | SI:Ich:MBIO1284 | MBIO1284.4 | JQ431964 |
| Cirrhitidae | *Paracirrhites hemistictus* | French Polynesia:Society Islands:Moorea | SI:Ich:MBIO1402 | MBIO1402.4 | JQ431961 |
| Cirrhitidae | *Paracirrhites hemistictus* | French Polynesia:Society Islands:Moorea | MNHN:Ich:BIOCODES0973 | MBIO1657.4 | JQ431965 |
| Cirrhitidae | *Paracirrhites hemistictus* | French Polynesia:Society Islands:Moorea | MNHN:Ich:MBIO1658 | MBIO1658.4 | JQ431963 |
| Cirrhitidae | *Paracirrhites hemistictus* | French Polynesia:Society Islands:Moorea | MNHN:Ich:BIOCODES0185 | MBIO261.4 | JQ431962 |
| Clupeidae | *Herklotsichthys spilurus* | Reunion:South:St Philippe | ECOMAR:Ich:REU1835 | REU1835 | JQ350053 |
| Congridae | *Ariosoma scheelei* | French Polynesia:Society Islands:Moorea | MNHN:Ich:BIOCODES0901 | MBIO1490.4 | JQ431461 |
| Congridae | *Ariosoma scheelei* | French Polynesia:Society Islands:Moorea | SI:Ich:MBIO1491 | MBIO1491.4 | JQ431460 |
| Congridae | *Conger cinereus* | French Polynesia:Society Islands:Moorea | MNHN:Ich:BIOCODES760 | MBIO1174.4 | JQ431651 |
| Congridae | *Conger cinereus* | French Polynesia:Society Islands:Moorea | SI:Ich:MBIO1175 | MBIO1175.4 | JQ431653 |
| Congridae | *Conger cinereus* | French Polynesia:Society Islands:Moorea | MNHN:Ich:BIOCODES269 | MBIO428.4 | JQ431652 |
| Congridae | *Conger cinereus* | Reunion:South:St Philippe | ECOMAR:Ich:REU1855 | REU1855 | JQ349914 |
| Congridae | *Conger cinereus* | Reunion:South:St Philippe | ECOMAR:Ich:REU1856 | REU1856 | JQ349912 |
| Congridae | *Conger cinereus* | Reunion:West:La Saline | ECOMAR:Ich:REU2549 | REU2549 | JQ349913 |
| Congridae | *Gorgasia galzini* | French Polynesia:Society Islands:Moorea | MNHN:Ich:BIOCODES0997 | MBIO1740.4 | JQ431775 |
| Congridae | *Heteroconger hassi* | Madagascar:West:Nosy Be | ECOMAR:Ich:NBE0122 | NBE0122 | JQ350055 |
| Congridae | *Heteroconger hassi* | Madagascar:West:Nosy Be | ECOMAR:Ich:NBE0123 | NBE0123 | JQ350054 |
| Congridae | *Heteroconger hassi* | Madagascar:West:Nosy Be | ECOMAR:Ich:NBE0124 | NBE0124 | JQ350056 |
| Congridae | *Heteroconger hassi* | Madagascar:West:Nosy Be | ECOMAR:Ich:NBE1005 | NBE1005 | JQ350057 |
| Congridae | *Heteroconger lentiginosus* | French Polynesia:Society Islands:Moorea | MNHN:Ich:MBIO1766 | MBIO1766.4 | JQ431857 |
| Creediidae | *Chalixodytes chameleontoculis* | French Polynesia:Society Islands:Moorea | MNHN:Ich:BIOCODES0854 | MBIO1388.4 | JQ431598 |
| Creediidae | *Chalixodytes chameleontoculis* | French Polynesia:Society Islands:Moorea | SI:Ich:MBIO1389 | MBIO1389.4 | JQ431599 |
| Creediidae | *Limnichthys nitidus* | French Polynesia:Society Islands:Moorea | MNHN:Ich:BIOCODES0101 | MBIO152.4 | JQ431887 |
| Creediidae | *Limnichthys nitidus* | French Polynesia:Society Islands:Moorea | SI:Ich:MBIO153 | MBIO153.4 | JQ431886 |
| Dactylopteridae | *Dactyloptena orientalis* | French Polynesia:Society Islands:Moorea | MNHN:Ich:BIOCODES736 | MBIO1135.4 | JQ431673 |
| Dactylopteridae | *Dactyloptena orientalis* | French Polynesia:Society Islands:Moorea | SI:Ich:MBIO1136 | MBIO1136.4 | JQ431672 |
| Dasyatidae | *Himantura fai* | French Polynesia:Society Islands:Moorea | MNHN:Ich:BIOCODES0890 | MBIO1476.4 | JQ431865 |
| Dasyatidae | *Himantura fai* | French Polynesia:Society Islands:Bora-Bora | UPVD:Ich:MBIO1874 | MBIO1874.4 | JQ431860 |
| Dasyatidae | *Himantura fai* | French Polynesia:Tuamotu Islands:Rangiroa | UPVD:Ich:MBIO1875 | MBIO1875.4 | JQ431861 |
| Dasyatidae | *Himantura fai* | French Polynesia:Tuamotu Islands:Rangiroa | UPVD:Ich:MBIO1876 | MBIO1876.4 | JQ431863 |
| Dasyatidae | *Himantura fai* | French Polynesia:Society Islands:Moorea | UPVD:Ich:MBIO1877 | MBIO1877.4 | JQ431866 |
| Dasyatidae | *Himantura fai* | French Polynesia:Tuamotu Islands:Mataiva | UPVD:Ich:MBIO1878 | MBIO1878.4 | JQ431862 |
| Dasyatidae | *Himantura fai* | French Polynesia:Society Islands:Tahiti | UPVD:Ich:MBIO1879 | MBIO1879.4 | JQ431864 |
| Diodontidae | *Diodon hystrix* | French Polynesia:Society Islands:Moorea | MNHN:Ich:BIOCODES0220 | MBIO319.4 | JQ431688 |
| Diodontidae | *Diodon hystrix* | French Polynesia:Society Islands:Moorea | MNHN:Ich:BIOCODES0221 | MBIO320.4 | JQ431687 |
| Eleotridae | *Calumia godeffroyi* | French Polynesia:Society Islands:Moorea | SI:Ich:MBIO1372 | MBIO1372.4 | JQ431519 |
| Eleotridae | *Calumia godeffroyi* | French Polynesia:Society Islands:Moorea | MNHN:Ich:BIOCODES0045 | MBIO81.4 | JQ431518 |
| Eleotridae | *Eleotris fusca* | French Polynesia:Society Islands:Moorea | MNHN:Ich:BIOCODES827 | MBIO1302.4 | JQ431699 |
| Eleotridae | *Eleotris fusca* | French Polynesia:Society Islands:Moorea | MNHN:Ich:BIOCODES829 | MBIO1303.4 | JQ431700 |
| Exocoetidae | *Cheilopogon pitcairnensis* | French Polynesia:Society Islands:Moorea | MNHN:Ich:BIOCODES0143 | MBIO208.4 | JQ431615 |
| Exocoetidae | *Cheilopogon pitcairnensis* | French Polynesia:Society Islands:Moorea | MNHN:Ich:BIOCODES0144 | MBIO209.4 | JQ431616 |
| Fistulariidae | *Fistularia commersonii* | French Polynesia:Society Islands:Moorea | MNHN:Ich:BIOCODES0378 | MBIO541.4 | JQ431741 |
| Fistulariidae | *Fistularia commersonii* | French Polynesia:Society Islands:Moorea | SI:Ich:MBIO743 | MBIO743.4 | JQ431740 |
| Fistulariidae | *Fistularia commersonii* | Reunion:West:St Gilles | ECOMAR:Ich:REU1672 | REU1672 | JQ349978 |
| Fistulariidae | *Fistularia commersonii* | Reunion:West:La Saline | ECOMAR:Ich:REU2548 | REU2548 | JQ349979 |
| Gerreidae | *Gerres oyena* | Madagascar:West:Nosy Be | ECOMAR:Ich:NBE1066 | NBE1066 | JQ349998 |
| Gerreidae | *Gerres oyena* | Madagascar:West:Nosy Be | ECOMAR:Ich:NBE1067 | NBE1067 | JQ349997 |
| Gerreidae | *Gerres oyena* | Madagascar:West:Nosy Be | ECOMAR:Ich:NBE1088 | NBE1088 | JQ349999 |
| Gobiidae | *Acentrogobius nebulosus* | Madagascar:West:Nosy Be | ECOMAR:Ich:NBE0498 | NBE0498 | JQ349675 |
| Gobiidae | *Acentrogobius nebulosus* | Madagascar:West:Nosy Be | ECOMAR:Ich:NBE0499 | NBE0499 | JQ349676 |
| Gobiidae | *Acentrogobius nebulosus* | Madagascar:West:Nosy Be | ECOMAR:Ich:NBE0500 | NBE0500 | JQ349674 |
| Gobiidae | *Acentrogobius nebulosus* | Madagascar:West:Nosy Be | ECOMAR:Ich:NBE0501 | NBE0501 | JQ349673 |
| Gobiidae | *Acentrogobius nebulosus* | Madagascar:West:Nosy Be | ECOMAR:Ich:NBE0502 | NBE0502 | JQ349672 |
| Gobiidae | *Amblyeleotris diagonalis* | Madagascar:West:Nosy Be | ECOMAR:Ich:NBE0460 | NBE0460 | JQ349681 |
| Gobiidae | *Amblyeleotris katherine* | French Polynesia:Society Islands:Moorea | MNHN:Ich:BIOCODES706 | MBIO1052.4 | JQ431408 |
| Gobiidae | *Amblyeleotris katherine* | French Polynesia:Society Islands:Moorea | SI:Ich:MBIO1053 | MBIO1053.4 | JQ431405 |
| Gobiidae | *Amblyeleotris katherine* | French Polynesia:Society Islands:Moorea | MNHN:Ich:BIOCODES838 | MBIO1317.4 | JQ431407 |
| Gobiidae | *Amblyeleotris katherine* | French Polynesia:Society Islands:Moorea | SI:Ich:MBIO1377 | MBIO1377.4 | JQ431406 |
| Gobiidae | *Amblygobius hectori* | Madagascar:West:Nosy Be | ECOMAR:Ich:NBE0117 | NBE0117 | JQ349683 |
| Gobiidae | *Amblygobius hectori* | Madagascar:West:Nosy Be | ECOMAR:Ich:NBE0118 | NBE0118 | JQ349687 |
| Gobiidae | *Amblygobius hectori* | Madagascar:West:Nosy Be | ECOMAR:Ich:NBE0119 | NBE0119 | JQ349686 |
| Gobiidae | *Amblygobius hectori* | Madagascar:West:Nosy Be | ECOMAR:Ich:NBE0507 | NBE0507 | JQ349685 |
| Gobiidae | *Amblygobius hectori* | Madagascar:West:Nosy Be | ECOMAR:Ich:NBE0547 | NBE0547 | JQ349684 |
| Gobiidae | *Amblygobius phalaena* | French Polynesia:Society Islands:Moorea | MNHN:Ich:BIOCODES768 | MBIO1188.4 | JQ431409 |
| Gobiidae | *Amblygobius sphynx* | Madagascar:West:Nosy Be | ECOMAR:Ich:NBE0456 | NBE0456 | JQ349693 |
| Gobiidae | *Amblygobius sphynx* | Madagascar:West:Nosy Be | ECOMAR:Ich:NBE0457 | NBE0457 | JQ349694 |
| Gobiidae | *Amblygobius sphynx* | Madagascar:West:Nosy Be | ECOMAR:Ich:NBE0461 | NBE0461 | JQ349689 |
| Gobiidae | *Amblygobius sphynx* | Madagascar:West:Nosy Be | ECOMAR:Ich:NBE0465 | NBE0465 | JQ349688 |
| Gobiidae | *Amblygobius sphynx* | Madagascar:West:Nosy Be | ECOMAR:Ich:NBE0466 | NBE0466 | JQ349695 |
| Gobiidae | *Amblygobius sphynx* | Madagascar:West:Nosy Be | ECOMAR:Ich:NBE0481 | NBE0481 | JQ349692 |
| Gobiidae | *Amblygobius sphynx* | Madagascar:West:Nosy Be | ECOMAR:Ich:NBE0482 | NBE0482 | JQ349691 |
| Gobiidae | *Amblygobius sphynx* | Madagascar:West:Nosy Be | ECOMAR:Ich:NBE0483 | NBE0483 | JQ349690 |
| Gobiidae | *Asterropteryx ensifera* | French Polynesia:Society Islands:Moorea | MNHN:Ich:BIOCODES0439 | MBIO639.4 | JQ431470 |
| Gobiidae | *Asterropteryx ensifera* | Madagascar:West:Nosy Be | ECOMAR:Ich:NBE0512 | NBE0512 | JQ349786 |
| Gobiidae | *Asterropteryx ensifera* | Madagascar:West:Nosy Be | ECOMAR:Ich:NBE0513 | NBE0513 | JQ349787 |
| Gobiidae | *Asterropteryx ensifera* | Madagascar:West:Nosy Be | ECOMAR:Ich:NBE0514 | NBE0514 | JQ349785 |
| Gobiidae | *Asterropteryx semipunctata* | French Polynesia:Society Islands:Moorea | SI:Ich:MBIO1754 | MBIO1754.4 | JQ431471 |
| Gobiidae | *Asterropteryx semipunctata* | Reunion:West:St Gilles | ECOMAR:Ich:REU1664 | REU1664 | JQ349790 |
| Gobiidae | *Asterropteryx semipunctata* | Reunion:West:St Gilles | ECOMAR:Ich:REU1665 | REU1665 | JQ349791 |
| Gobiidae | *Asterropteryx semipunctata* | Reunion:West:St Leu | ECOMAR:Ich:REU2614 | REU2614 | JQ349788 |
| Gobiidae | *Asterropteryx semipunctata* | Reunion:West:St Leu | ECOMAR:Ich:REU2615 | REU2615 | JQ349789 |
| Gobiidae | *Awaous ocellaris* | French Polynesia:Society Islands:Moorea | MNHN:Ich:BIOCODES1016 | MBIO1813.4 | JQ431473 |
| Gobiidae | *Bathygobius coalitus* | French Polynesia:Society Islands:Moorea | MNHN:Ich:BIOCODES561 | MBIO827.4 | JQ431479 |
| Gobiidae | *Bathygobius cocosensis* | French Polynesia:Society Islands:Moorea | MNHN:Ich:BIOCODES556 | MBIO822.4 | JQ431481 |
| Gobiidae | *Bathygobius cocosensis* | French Polynesia:Society Islands:Moorea | SI:Ich:MBIO823 | MBIO823.4 | JQ431480 |
| Gobiidae | *Bathygobius cotticeps* | French Polynesia:Society Islands:Moorea | SI:Ich:MBIO1692 | MBIO1692.4 | JQ431482 |
| Gobiidae | *Bathygobius cotticeps* | French Polynesia:Society Islands:Moorea | MNHN:Ich:MBIO1736 | MBIO1736.4 | JQ431483 |
| Gobiidae | *Cabillus tongarevae* | French Polynesia:Society Islands:Moorea | MNHN:Ich:BIOCODES274 | MBIO409.4 | JQ431503 |
| Gobiidae | *Cabillus tongarevae* | French Polynesia:Society Islands:Moorea | MNHN:Ich:BIOCODES273 | MBIO410.4 | JQ431504 |
| Gobiidae | *Callogobius sclateri* | French Polynesia:Society Islands:Moorea | MNHN:Ich:BIOCODES0252 | MBIO367.4 | JQ431514 |
| Gobiidae | *Callogobius sclateri* | French Polynesia:Society Islands:Moorea | MNHN:Ich:BIOCODES0251 | MBIO368.4 | JQ431513 |
| Gobiidae | *Callogobius sclateri* | French Polynesia:Society Islands:Moorea | MNHN:Ich:BIOCODES0445 | MBIO637.4 | JQ431512 |
| Gobiidae | *Coryphopterus duospilus* | French Polynesia:Society Islands:Moorea | MNHN:Ich:BIOCODES846 | MBIO1337.4 | JQ431661 |
| Gobiidae | *Coryphopterus duospilus* | French Polynesia:Society Islands:Moorea | MNHN:Ich:BIOCODES845 | MBIO1338.4 | JQ431660 |
| Gobiidae | *Coryphopterus humeralis* | French Polynesia:Society Islands:Moorea | MNHN:Ich:BIOCODES0267 | MBIO388.4 | JQ431663 |
| Gobiidae | *Coryphopterus humeralis* | French Polynesia:Society Islands:Moorea | SI:Ich:MBIO389 | MBIO389.4 | JQ431662 |
| Gobiidae | *Coryphopterus neophytus* | French Polynesia:Society Islands:Moorea | MNHN:Ich:BIOCODES1005 | MBIO1779.4 | JQ431664 |
| Gobiidae | *Coryphopterus neophytus* | French Polynesia:Society Islands:Moorea | SI:Ich:MBIO1780 | MBIO1780.4 | JQ431665 |
| Gobiidae | *Coryphopterus neophytus* | Reunion:West:St Gilles | ECOMAR:Ich:REU1624 | REU1624 | JQ349915 |
| Gobiidae | *Ctenogobiops feroculus* | French Polynesia:Society Islands:Moorea | MNHN:Ich:BIOCODES0263 | MBIO383.4 | JQ431671 |
| Gobiidae | *Eviota albolineata* | French Polynesia:Society Islands:Moorea | MNHN:Ich:BIOCODES0172 | MBIO254.4 | JQ431731 |
| Gobiidae | *Eviota albolineata* | French Polynesia:Society Islands:Moorea | MNHN:Ich:BIOCODES0279 | MBIO396.4 | JQ431729 |
| Gobiidae | *Eviota albolineata* | French Polynesia:Society Islands:Moorea | MNHN:Ich:BIOCODES0280 | MBIO397.4 | JQ431732 |
| Gobiidae | *Eviota albolineata* | French Polynesia:Society Islands:Moorea | MNHN:Ich:BIOCODES0434 | MBIO621.4 | JQ431728 |
| Gobiidae | *Eviota albolineata* | French Polynesia:Society Islands:Moorea | MNHN:Ich:BIOCODES0433 | MBIO622.4 | JQ431730 |
| Gobiidae | *Eviota disrupta* | French Polynesia:Society Islands:Moorea | MNHN:Ich:BIOCODES0174 | MBIO250.4 | JQ431734 |
| Gobiidae | *Eviota disrupta* | French Polynesia:Society Islands:Moorea | MNHN:Ich:BIOCODES0175 | MBIO251.4 | JQ431733 |
| Gobiidae | *Eviota distigma* | Madagascar:West:Nosy Be | ECOMAR:Ich:NBE0136 | NBE0136 | JQ349971 |
| Gobiidae | *Eviota indica* | Madagascar:West:Nosy Be | ECOMAR:Ich:NBE0569 | NBE0569 | JQ349972 |
| Gobiidae | *Eviota prasina* | Madagascar:West:Nosy Be | ECOMAR:Ich:NBE1158 | NBE1158 | JQ349973 |
| Gobiidae | *Eviota* sp. | French Polynesia:Society Islands:Moorea | MNHN:Ich:BIOCODES0269 | MBIO391.4 | JQ431736 |
| Gobiidae | *Eviota* sp*.* | French Polynesia:Society Islands:Moorea | MNHN:Ich:BIOCODES0271 | MBIO392.4 | JQ431735 |
| Gobiidae | *Fusigobius* sp. | Madagascar:West:Nosy Be | ECOMAR:Ich:NBE0486 | NBE0486 | JQ349994 |
| Gobiidae | *Fusigobius* sp*.* | Madagascar:West:Nosy Be | ECOMAR:Ich:NBE0487 | NBE0487 | JQ349996 |
| Gobiidae | *Fusigobius* sp*.* | Madagascar:West:Nosy Be | ECOMAR:Ich:NBE0488 | NBE0488 | JQ349995 |
| Gobiidae | *Gnatholepis anjerensis* | French Polynesia:Society Islands:Moorea | SI:Ich:MBIO1514 | MBIO1514.4 | JQ431760 |
| Gobiidae | *Gnatholepis anjerensis* | French Polynesia:Society Islands:Moorea | SI:Ich:MBIO1524 | MBIO1524.4 | JQ431759 |
| Gobiidae | *Gnatholepis anjerensis* | French Polynesia:Society Islands:Moorea | MNHN:Ich:BIOCODES0914 | MBIO1525.4 | JQ431757 |
| Gobiidae | *Gnatholepis anjerensis* | French Polynesia:Society Islands:Moorea | MNHN:Ich:BIOCODES281 | MBIO404.4 | JQ431758 |
| Gobiidae | *Gnatholepis cauerensis* | French Polynesia:Society Islands:Moorea | MNHN:Ich:BIOCODES0421 | MBIO597.4 | JQ431762 |
| Gobiidae | *Gnatholepis cauerensis* | French Polynesia:Society Islands:Moorea | MNHN:Ich:BIOCODES0417 | MBIO598.4 | JQ431761 |
| Gobiidae | *Gnatholepis scapulostigma* | Reunion:West:St Gilles | ECOMAR:Ich:REU1623 | REU1623 | JQ350003 |
| Gobiidae | *Gobiodon citrinus* | Madagascar:West:Nosy Be | ECOMAR:Ich:NBE1000 | NBE1000 | JQ350005 |
| Gobiidae | *Gobiodon citrinus* | Madagascar:West:Nosy Be | ECOMAR:Ich:NBE1001 | NBE1001 | JQ350004 |
| Gobiidae | *Gobiodon exigua* | French Polynesia:Society Islands:Moorea | SI:Ich:MBIO1342 | MBIO1342.4 | JQ431763 |
| Gobiidae | *Gobiodon exigua* | French Polynesia:Society Islands:Moorea | MNHN:Ich:BIOCODES0102 | MBIO154.4 | JQ431764 |
| Gobiidae | *Gobiodon prolixus* | Madagascar:West:Nosy Be | ECOMAR:Ich:NBE0550 | NBE0550 | JQ350006 |
| Gobiidae | *Gobiodon quinquestrigatus* | French Polynesia:Society Islands:Moorea | MNHN:Ich:BIOCODES847 | MBIO1340.4 | JQ431767 |
| Gobiidae | *Gobiodon quinquestrigatus* | French Polynesia:Society Islands:Moorea | SI:Ich:MBIO1341 | MBIO1341.4 | JQ431768 |
| Gobiidae | *Gobiodon quinquestrigatus* | French Polynesia:Society Islands:Moorea | MNHN:Ich:BIOCODES0117 | MBIO173.4 | JQ431765 |
| Gobiidae | *Gobiodon quinquestrigatus* | French Polynesia:Society Islands:Moorea | MNHN:Ich:BIOCODES0118 | MBIO174.4 | JQ431766 |
| Gobiidae | *Gobiodon rivulatus* | Reunion:West:La Saline | ECOMAR:Ich:REU2584 | REU2584 | JQ350010 |
| Gobiidae | *Gobiodon rivulatus* | Reunion:West:La Saline | ECOMAR:Ich:REU2585 | REU2585 | JQ350007 |
| Gobiidae | *Gobiodon rivulatus* | Reunion:West:La Saline | ECOMAR:Ich:REU2586 | REU2586 | JQ350009 |
| Gobiidae | *Gobiodon rivulatus* | Reunion:West:La Saline | ECOMAR:Ich:REU2587 | REU2587 | JQ350008 |
| Gobiidae | *Gobiodon unicolor* | French Polynesia:Society Islands:Moorea | SI:Ich:MBIO1365 | MBIO1365.4 | JQ431769 |
| Gobiidae | *Gobiodon unicolor* | Reunion:West:La Saline | ECOMAR:Ich:REU2591 | REU2591 | JQ350011 |
| Gobiidae | *Gobiodon unicolor* | Reunion:West:La Saline | ECOMAR:Ich:REU2592 | REU2592 | JQ350012 |
| Gobiidae | *Paragobiodon echinocephalus* | Madagascar:West:Nosy Be | ECOMAR:Ich:NBE0546 | NBE0546 | JQ350172 |
| Gobiidae | *Paragobiodon lacunicolus* | French Polynesia:Society Islands:Moorea | MNHN:Ich:BIOCODES0262 | MBIO379.4 | JQ431966 |
| Gobiidae | *Paragobiodon lacunicolus* | French Polynesia:Society Islands:Moorea | MNHN:Ich:BIOCODES0257 | MBIO380.4 | JQ431967 |
| Gobiidae | *Paragobiodon modestus* | French Polynesia:Society Islands:Moorea | MNHN:Ich:BIOCODES0123 | MBIO181.4 | JQ431968 |
| Gobiidae | *Paragobiodon modestus* | French Polynesia:Society Islands:Moorea | MNHN:Ich:BIOCODES0124 | MBIO182.4 | JQ431971 |
| Gobiidae | *Paragobiodon modestus* | French Polynesia:Society Islands:Moorea | MNHN:Ich:BIOCODES0240 | MBIO353.4 | JQ431970 |
| Gobiidae | *Paragobiodon modestus* | French Polynesia:Society Islands:Moorea | MNHN:Ich:BIOCODES0241 | MBIO354.4 | JQ431969 |
| Gobiidae | *Pleurosicya labiata* | French Polynesia:Society Islands:Moorea | MNHN:Ich:BIOCODES276 | MBIO405.4 | JQ432019 |
| Gobiidae | *Pleurosicya labiata* | French Polynesia:Society Islands:Moorea | MNHN:Ich:BIOCODES277 | MBIO406.4 | JQ432018 |
| Gobiidae | *Pleurosicya mossambica* | French Polynesia:Society Islands:Moorea | SI:Ich:MBIO1777 | MBIO1777.4 | JQ432021 |
| Gobiidae | *Pleurosicya mossambica* | French Polynesia:Society Islands:Moorea | MNHN:Ich:BIOCODES0440 | MBIO636.4 | JQ432020 |
| Gobiidae | *Priolepis ailina* | French Polynesia:Society Islands:Moorea | MNHN:Ich:BIOCODES0851 | MBIO1366.4 | JQ432032 |
| Gobiidae | *Priolepis cinctus* | Madagascar:West:Nosy Be | ECOMAR:Ich:NBE1151 | NBE1151 | JQ350252 |
| Gobiidae | *Priolepis cinctus* | Madagascar:West:Nosy Be | ECOMAR:Ich:NBE1152 | NBE1152 | JQ350249 |
| Gobiidae | *Priolepis cinctus* | Madagascar:West:Nosy Be | ECOMAR:Ich:NBE1153 | NBE1153 | JQ350250 |
| Gobiidae | *Priolepis cinctus* | Reunion:South:St Philippe | ECOMAR:Ich:REU1860 | REU1860 | JQ350251 |
| Gobiidae | *Priolepis compita* | French Polynesia:Society Islands:Moorea | MNHN:Ich:BIOCODES0110 | MBIO165.4 | JQ432033 |
| Gobiidae | *Priolepis farcimen* | French Polynesia:Society Islands:Moorea | MNHN:Ich:BIOCODES0181 | MBIO255.4 | JQ432035 |
| Gobiidae | *Priolepis farcimen* | French Polynesia:Society Islands:Moorea | SI:Ich:MBIO256 | MBIO256.4 | JQ432034 |
| Gobiidae | *Priolepis inhaca* | French Polynesia:Society Islands:Moorea | SI:Ich:MBIO1244 | MBIO1244.4 | JQ432037 |
| Gobiidae | *Priolepis inhaca* | French Polynesia:Society Islands:Moorea | MNHN:Ich:BIOCODES0438 | MBIO633.4 | JQ432036 |
| Gobiidae | *Priolepis inhaca* | Madagascar:West:Nosy Be | ECOMAR:Ich:NBE0574 | NBE0574 | JQ350253 |
| Gobiidae | *Priolepis semidoliata* | French Polynesia:Society Islands:Moorea | MNHN:Ich:BIOCODES0282 | MBIO400.4 | JQ432038 |
| Gobiidae | *Priolepis semidoliata* | French Polynesia:Society Islands:Moorea | MNHN:Ich:BIOCODES0283 | MBIO401.4 | JQ432039 |
| Gobiidae | *Priolepis squamogena* | French Polynesia:Society Islands:Moorea | MNHN:Ich:BIOCODES0418 | MBIO593.4 | JQ432040 |
| Gobiidae | *Priolepis squamogena* | French Polynesia:Society Islands:Moorea | MNHN:Ich:BIOCODES0415 | MBIO594.4 | JQ432041 |
| Gobiidae | *Priolepis triops* | French Polynesia:Society Islands:Moorea | MNHN:Ich:BIOCODES0916 | MBIO1531.4 | JQ432042 |
| Gobiidae | *Sicyopterus lagocephalus* | French Polynesia:Society Islands:Moorea | MNHN:Ich:BIOCODES828 | MBIO1306.4 | JQ432152 |
| Gobiidae | *Sicyopterus lagocephalus* | French Polynesia:Society Islands:Moorea | MNHN:Ich:BIOCODES830 | MBIO1307.4 | JQ432151 |
| Gobiidae | *Sicyopterus pugnans* | French Polynesia:Society Islands:Moorea | MNHN:Ich:BIOCODES831 | MBIO1299.4 | JQ432153 |
| Gobiidae | *Sicyopterus pugnans* | French Polynesia:Society Islands:Moorea | MNHN:Ich:BIOCODES1010 | MBIO1806.4 | JQ432155 |
| Gobiidae | *Sicyopterus pugnans* | French Polynesia:Society Islands:Moorea | MNHN:Ich:BIOCODES1013 | MBIO1807.4 | JQ432154 |
| Gobiidae | *Stenogobius genivittatus* | French Polynesia:Society Islands:Moorea | MNHN:Ich:BIOCODES1014 | MBIO1810.4 | JQ432169 |
| Gobiidae | *Stenogobius genivittatus* | French Polynesia:Society Islands:Moorea | MNHN:Ich:BIOCODES1015 | MBIO1811.4 | JQ432168 |
| Gobiidae | *Stiphodon elegans* | French Polynesia:Society Islands:Moorea | MNHN:Ich:BIOCODES1009 | MBIO1804.4 | JQ432173 |
| Gobiidae | *Stiphodon elegans* | French Polynesia:Society Islands:Moorea | SI:Ich:MBIO1805 | MBIO1805.4 | JQ432172 |
| Gobiidae | *Trimma macrophthalma* | Madagascar:West:Nosy Be | ECOMAR:Ich:NBE1159 | NBE1159 | JQ350401 |
| Gobiidae | *Trimma macrophthalma* | Madagascar:West:Nosy Be | ECOMAR:Ich:NBE1160 | NBE1160 | JQ350402 |
| Gobiidae | *Trimma mendelssohni* | Madagascar:West:Nosy Be | ECOMAR:Ich:NBE0489 | NBE0489 | JQ350405 |
| Gobiidae | *Trimma mendelssohni* | Madagascar:West:Nosy Be | ECOMAR:Ich:NBE0490 | NBE0490 | JQ350404 |
| Gobiidae | *Trimma mendelssohni* | Madagascar:West:Nosy Be | ECOMAR:Ich:NBE0491 | NBE0491 | JQ350407 |
| Gobiidae | *Trimma mendelssohni* | Madagascar:West:Nosy Be | ECOMAR:Ich:NBE0551 | NBE0551 | JQ350406 |
| Gobiidae | *Trimma mendelssohni* | Madagascar:West:Nosy Be | ECOMAR:Ich:NBE0552 | NBE0552 | JQ350403 |
| Gobiidae | *Trimma milta* | French Polynesia:Society Islands:Moorea | MNHN:Ich:BIOCODES0431 | MBIO612.4 | JQ432199 |
| Gobiidae | *Trimma milta* | French Polynesia:Society Islands:Moorea | MNHN:Ich:BIOCODES0436 | MBIO617.4 | JQ432200 |
| Gobiidae | *Trimma milta* | French Polynesia:Society Islands:Moorea | MNHN:Ich:BIOCODES0441 | MBIO626.4 | JQ432201 |
| Gobiidae | *Trimma milta* | French Polynesia:Society Islands:Moorea | MNHN:Ich:BIOCODES0442 | MBIO627.4 | JQ432198 |
| Gobiidae | *Valenciennea helsdingenii* | Madagascar:West:Nosy Be | ECOMAR:Ich:NBE1293 | NBE1293 | JQ350413 |
| Gobiidae | *Valenciennea parva* | Madagascar:West:Nosy Be | ECOMAR:Ich:NBE0612 | NBE0612 | JQ350414 |
| Gobiidae | *Valenciennea strigata* | French Polynesia:Society Islands:Moorea | MNHN:Ich:BIOCODES0906 | MBIO1507.4 | JQ432215 |
| Gobiidae | *Valenciennea strigata* | French Polynesia:Society Islands:Moorea | SI:Ich:MBIO1508 | MBIO1508.4 | JQ432214 |
| Gobiidae | *Valenciennea strigata* | Madagascar:West:Nosy Be | ECOMAR:Ich:NBE0368 | NBE0368 | JQ350415 |
| Gobiidae | *Valenciennea strigata* | Reunion:West:St Leu | ECOMAR:Ich:REU1024 | REU1024 | JQ350416 |
| Haemulidae | *Diagramma pictum* | Madagascar:West:Nosy Be | ECOMAR:Ich:NBE0002 | NBE0002 | JQ349935 |
| Haemulidae | *Diagramma pictum* | Madagascar:West:Nosy Be | ECOMAR:Ich:NBE0003 | NBE0003 | JQ349934 |
| Haemulidae | *Diagramma pictum* | Madagascar:West:Nosy Be | ECOMAR:Ich:NBE0205 | NBE0205 | JQ349933 |
| Haemulidae | *Plectorhinchus flavomaculatus* | Madagascar:West:Nosy Be | ECOMAR:Ich:NBE0340 | NBE0340 | JQ350217 |
| Haemulidae | *Plectorhinchus gaterinus* | Madagascar:West:Nosy Be | ECOMAR:Ich:NBE0004 | NBE0004 | JQ350219 |
| Haemulidae | *Plectorhinchus gaterinus* | Madagascar:West:Nosy Be | ECOMAR:Ich:NBE0005 | NBE0005 | JQ350218 |
| Haemulidae | *Plectorhinchus gaterinus* | Madagascar:West:Nosy Be | ECOMAR:Ich:NBE0200 | NBE0200 | JQ350220 |
| Haemulidae | *Plectorhinchus gaterinus* | Madagascar:West:Nosy Be | ECOMAR:Ich:NBE0632 | NBE0632 | JQ350221 |
| Haemulidae | *Plectorhinchus orientalis* | Madagascar:West:Nosy Be | ECOMAR:Ich:NBE1300 | NBE1300 | JQ350222 |
| Holocentridae | *Myripristis adusta* | French Polynesia:Society Islands:Moorea | MNHN:Ich:BIOCODES762 | MBIO1181.4 | HM034216 |
| Holocentridae | *Myripristis adusta* | French Polynesia:Society Islands:Moorea | SI:Ich:MBIO1182 | MBIO1182.4 | HM034215 |
| Holocentridae | *Myripristis amaena* | French Polynesia:Society Islands:Moorea | MNHN:Ich:BIOCODES0940 | MBIO1598.4 | HM034220 |
| Holocentridae | *Myripristis amaena* | French Polynesia:Society Islands:Moorea | MNHN:Ich:BIOCODES0967 | MBIO1634.4 | HM034218 |
| Holocentridae | *Myripristis amaena* | French Polynesia:Society Islands:Moorea | SI:Ich:MBIO1635 | MBIO1635.4 | HM034217 |
| Holocentridae | *Myripristis amaena* | French Polynesia:Society Islands:Moorea | MNHN:Ich:BIOCODES1039 | MBIO1840.4 | HM034219 |
| Holocentridae | *Myripristis amaena* | French Polynesia:Society Islands:Tetiaroa | MNHN:Ich:2009-1651 | MOCNESS M9.1-M0-33 | HM034160 |
| Holocentridae | *Myripristis berndti* | French Polynesia:Society Islands:Moorea | MNHN:Ich:BIOCODES776 | MBIO1210.4 | HM034227 |
| Holocentridae | *Myripristis berndti* | French Polynesia:Society Islands:Moorea | MNHN:Ich:BIOCODES712 | MBIO1211.4 | HM034226 |
| Holocentridae | *Myripristis berndti* | French Polynesia:Society Islands:Moorea | MNHN:Ich:BIOCODES0968 | MBIO1630.4 | HM034222 |
| Holocentridae | *Myripristis berndti* | French Polynesia:Society Islands:Moorea | SI:Ich:MBIO1631 | MBIO1631.4 | HM034221 |
| Holocentridae | *Myripristis berndti* | French Polynesia:Marquesas Islands:Mohotane | CRIOBE:Ich:MBIO18161 | MBIO18161 | HM034225 |
| Holocentridae | *Myripristis berndti* | French Polynesia:Marquesas Islands:Mohotane | CRIOBE:Ich:MBIO18218 | MBIO18218 | HM034224 |
| Holocentridae | *Myripristis berndti* | French Polynesia:Marquesas Islands:Mohotane | CRIOBE:Ich:MBIO18221 | MBIO18221 | HM034223 |
| Holocentridae | *Myripristis berndti* | French Polynesia:Society Islands:Moorea | MNHN:Ich:BIOCODES0374 | MBIO534.4 | HM034229 |
| Holocentridae | *Myripristis berndti* | French Polynesia:Society Islands:Moorea | MNHN:Ich:BIOCODES0371 | MBIO535.4 | HM034228 |
| Holocentridae | *Myripristis berndti* | French Polynesia:Society Islands:Tetiaroa | MNHN:Ich:2009-1652 | MOCNESS M9.1-M0-32 | HM034161 |
| Holocentridae | *Myripristis berndti* | French Polynesia:Society Islands:Tetiaroa | MNHN:Ich:2009-1653 | MOCNESS M9.1-M0-35 | HM034162 |
| Holocentridae | *Myripristis berndti* | Madagascar:West:Nosy Be | ECOMAR:Ich:NBE0070 | NBE0070 | JQ350114 |
| Holocentridae | *Myripristis berndti* | Madagascar:West:Nosy Be | ECOMAR:Ich:NBE0071 | NBE0071 | JQ350117 |
| Holocentridae | *Myripristis berndti* | Madagascar:West:Nosy Be | ECOMAR:Ich:NBE0072 | NBE0072 | JQ350116 |
| Holocentridae | *Myripristis berndti* | Madagascar:West:Nosy Be | ECOMAR:Ich:NBE0463 | NBE0463 | JQ350115 |
| Holocentridae | *Myripristis hexagona* | Reunion:West:St Gilles | ECOMAR:Ich:REU112-1 | REU112_1 | JQ350118 |
| Holocentridae | *Myripristis hexagona* | Reunion:West:St Gilles | ECOMAR:Ich:REU157-1 | REU157_1 | JQ350119 |
| Holocentridae | *Myripristis hexagona* | Reunion:West:St Leu | ECOMAR:Ich:REU2677 | REU2677 | JQ350121 |
| Holocentridae | *Myripristis hexagona* | Reunion:West:St Leu | ECOMAR:Ich:REU2678 | REU2678 | JQ350120 |
| Holocentridae | *Myripristis kuntee* | French Polynesia:Society Islands:Moorea | MNHN:Ich:BIOCODES0966 | MBIO1632.4 | HM034231 |
| Holocentridae | *Myripristis kuntee* | French Polynesia:Society Islands:Moorea | SI:Ich:MBIO1633 | MBIO1633.4 | HM034230 |
| Holocentridae | *Myripristis kuntee* | French Polynesia:Marquesas Islands:Mohotane | CRIOBE:Ich:MBIO18235 | MBIO18235 | HM034232 |
| Holocentridae | *Myripristis pralinia* | French Polynesia:Society Islands:Moorea | MNHN:Ich:BIOCODES841 | MBIO1321.4 | HM034236 |
| Holocentridae | *Myripristis pralinia* | French Polynesia:Society Islands:Moorea | MNHN:Ich:BIOCODES842 | MBIO1322.4 | HM034235 |
| Holocentridae | *Myripristis pralinia* | French Polynesia:Society Islands:Moorea | MNHN:Ich:BIOCODES0969 | MBIO1627.4 | HM034234 |
| Holocentridae | *Myripristis pralinia* | French Polynesia:Society Islands:Moorea | MNHN:Ich:BIOCODES0965 | MBIO1628.4 | HM034233 |
| Holocentridae | *Myripristis pralinia* | French Polynesia:Society Islands:Moorea | MNHN:Ich:BIOCODES0114 | MBIO170.4 | HM034238 |
| Holocentridae | *Myripristis pralinia* | French Polynesia:Society Islands:Moorea | MNHN:Ich:BIOCODES611 | MBIO903.4 | HM034237 |
| Holocentridae | *Myripristis pralinia* | Madagascar:West:Nosy Be | ECOMAR:Ich:NBE0068 | NBE0068 | JQ350123 |
| Holocentridae | *Myripristis pralinia* | Madagascar:West:Nosy Be | ECOMAR:Ich:NBE0069 | NBE0069 | JQ350122 |
| Holocentridae | *Myripristis violacea* | French Polynesia:Society Islands:Moorea | MNHN:Ich:BIOCODES1000 | MBIO1744.4 | HM034240 |
| Holocentridae | *Myripristis violacea* | French Polynesia:Society Islands:Moorea | MNHN:Ich:BIOCODES1001 | MBIO1745.4 | HM034239 |
| Holocentridae | *Myripristis violacea* | French Polynesia:Society Islands:Tetiaroa | MNHN:Ich:2009-1654 | MOCNESS M9.1-M0-34 | HM034163 |
| Holocentridae | *Neoniphon sammara* | French Polynesia:Society Islands:Moorea | MNHN:Ich:BIOCODES0929 | MBIO1574.4 | HM034253 |
| Holocentridae | *Neoniphon sammara* | French Polynesia:Society Islands:Moorea | SI:Ich:MBIO1575 | MBIO1575.4 | HM034252 |
| Holocentridae | *Neoniphon sammara* | French Polynesia:Society Islands:Moorea | MNHN:Ich:BIOCODES0308 | MBIO446.4 | HM034255 |
| Holocentridae | *Neoniphon sammara* | French Polynesia:Society Islands:Moorea | MNHN:Ich:BIOCODES0312 | MBIO447.4 | HM034254 |
| Holocentridae | *Neoniphon sammara* | Reunion:West:St Gilles | ECOMAR:Ich:REU072-1 | REU072_1 | JQ350142 |
| Holocentridae | *Neoniphon sammara* | Reunion:West:St Gilles | ECOMAR:Ich:REU072-2 | REU072_2 | JQ350141 |
| Holocentridae | *Neoniphon sammara* | Reunion:West:La Saline | ECOMAR:Ich:REU206-1 | REU206_1 | JQ350140 |
| Holocentridae | *Neoniphon sammara* | Reunion:West:La Saline | ECOMAR:Ich:REU206-2 | REU206_2 | JQ350138 |
| Holocentridae | *Neoniphon sammara* | Reunion:West:St Leu | ECOMAR:Ich:REU2664 | REU2664 | JQ350139 |
| Holocentridae | *Plectrypops lima* | French Polynesia:Society Islands:Moorea | MNHN:Ich:BIOCODES0115 | MBIO171.4 | HM034256 |
| Holocentridae | *Plectrypops lima* | Reunion:West:St Leu | ECOMAR:Ich:REU0946 | REU0946 | JQ350231 |
| Holocentridae | *Plectrypops lima* | Reunion:West:St Leu | ECOMAR:Ich:REU0947 | REU0947 | JQ350230 |
| Holocentridae | *Sargocentron caudimaculatum* | French Polynesia:Marquesas Islands:Mohotane | CRIOBE:Ich:MBIO18217 | MBIO18217 | HM034257 |
| Holocentridae | *Sargocentron caudimaculatum* | French Polynesia:Society Islands:Moorea | MNHN:Ich:BIOCODES0428 | MBIO609.4 | HM034272 |
| Holocentridae | *Sargocentron caudimaculatum* | French Polynesia:Society Islands:Tetiaroa | MNHN:Ich:2009-1655 | MOCNESS M9.1-M0-21 | HM034164 |
| Holocentridae | *Sargocentron caudimaculatum* | French Polynesia:Society Islands:Tetiaroa | MNHN:Ich:2009-1656 | MOCNESS M9.1-M0-23 | HM034165 |
| Holocentridae | *Sargocentron caudimaculatum* | Madagascar:West:Nosy Be | ECOMAR:Ich:NBE0353 | NBE0353 | JQ350308 |
| Holocentridae | *Sargocentron diadema* | French Polynesia:Society Islands:Moorea | MNHN:Ich:BIOCODES778 | MBIO1206.4 | HM034259 |
| Holocentridae | *Sargocentron diadema* | French Polynesia:Society Islands:Moorea | MNHN:Ich:BIOCODES711 | MBIO1207.4 | HM034258 |
| Holocentridae | *Sargocentron diadema* | Reunion:West:St Gilles | ECOMAR:Ich:REU158-1 | REU158_1 | JQ350311 |
| Holocentridae | *Sargocentron diadema* | Reunion:West:St Gilles | ECOMAR:Ich:REU158-2 | REU158_2 | JQ350310 |
| Holocentridae | *Sargocentron diadema* | Reunion:West:St Gilles | ECOMAR:Ich:REU1625 | REU1625 | JQ350309 |
| Holocentridae | *Sargocentron diadema* | Reunion:West:St Leu | ECOMAR:Ich:REU2668 | REU2668 | JQ350312 |
| Holocentridae | *Sargocentron melanospilos* | French Polynesia:Society Islands:Moorea | MNHN:Ich:BIOCODES779 | MBIO1205.4 | HM034260 |
| Holocentridae | *Sargocentron melanospilos* | French Polynesia:Society Islands:Moorea | MNHN:Ich:BIOCODES568 | MBIO839.4 | HM034261 |
| Holocentridae | *Sargocentron microstoma* | French Polynesia:Society Islands:Moorea | MNHN:Ich:BIOCODES0111 | MBIO166.4 | HM034265 |
| Holocentridae | *Sargocentron microstoma* | French Polynesia:Society Islands:Moorea | MNHN:Ich:BIOCODES0112 | MBIO167.4 | HM034264 |
| Holocentridae | *Sargocentron microstoma* | French Polynesia:Society Islands:Moorea | MNHN:Ich:BIOCODES508 | MBIO751.4 | HM034263 |
| Holocentridae | *Sargocentron microstoma* | French Polynesia:Society Islands:Moorea | MNHN:Ich:BIOCODES509 | MBIO752.4 | HM034262 |
| Holocentridae | *Sargocentron punctatissimum* | French Polynesia:Marquesas Islands:Mohotane | CRIOBE:Ich:MBIO18192 | MBIO18192 | HM034266 |
| Holocentridae | *Sargocentron punctatissimum* | French Polynesia:Society Islands:Moorea | MNHN:Ich:BIOCODES0155 | MBIO223.4 | HM034270 |
| Holocentridae | *Sargocentron punctatissimum* | French Polynesia:Society Islands:Moorea | MNHN:Ich:BIOCODES0156 | MBIO224.4 | HM034269 |
| Holocentridae | *Sargocentron punctatissimum* | French Polynesia:Society Islands:Moorea | MNHN:Ich:BIOCODES558 | MBIO824.4 | HM034268 |
| Holocentridae | *Sargocentron punctatissimum* | French Polynesia:Society Islands:Moorea | MNHN:Ich:BIOCODES557 | MBIO825.4 | HM034267 |
| Holocentridae | *Sargocentron punctatissimum* | Reunion:West:St Gilles | ECOMAR:Ich:REU159-1 | REU159_1 | JQ350314 |
| Holocentridae | *Sargocentron punctatissimum* | Reunion:West:St Gilles | ECOMAR:Ich:REU159-2 | REU159_2 | JQ350315 |
| Holocentridae | *Sargocentron punctatissimum* | Reunion:West:St Gilles | ECOMAR:Ich:REU1630 | REU1630 | JQ350313 |
| Holocentridae | *Sargocentron punctatissimum* | Reunion:West:St Gilles | ECOMAR:Ich:REU1631 | REU1631 | JQ350316 |
| Holocentridae | *Sargocentron punctatissimum* | Reunion:South:St Philippe | ECOMAR:Ich:REU1753 | REU1753 | JQ350317 |
| Holocentridae | *Sargocentron sp.* | French Polynesia:Society Islands:Moorea | SI:Ich:MBIO1709 | MBIO1709.4 | HM034271 |
| Holocentridae | *Sargocentron spiniferum* | French Polynesia:Society Islands:Moorea | MNHN:Ich:BIOCODES0305 | MBIO443.4 | HM034276 |
| Holocentridae | *Sargocentron spiniferum* | French Polynesia:Society Islands:Moorea | SI:Ich:MBIO444 | MBIO444.4 | HM034275 |
| Holocentridae | *Sargocentron spiniferum* | French Polynesia:Society Islands:Moorea | MNHN:Ich:BIOCODES0373 | MBIO530.4 | HM034274 |
| Holocentridae | *Sargocentron spiniferum* | French Polynesia:Society Islands:Moorea | MNHN:Ich:BIOCODES0369 | MBIO531.4 | HM034273 |
| Holocentridae | *Sargocentron spiniferum* | Madagascar:West:Nosy Be | ECOMAR:Ich:NBE0107 | NBE0107 | JQ350318 |
| Holocentridae | *Sargocentron spiniferum* | Reunion:West:St Leu | ECOMAR:Ich:REU2663 | REU2663 | JQ350319 |
| Holocentridae | *Sargocentron tiere* | French Polynesia:Society Islands:Moorea | MNHN:Ich:BIOCODES0138 | MBIO200.4 | HM034279 |
| Holocentridae | *Sargocentron tiere* | French Polynesia:Society Islands:Moorea | MNHN:Ich:BIOCODES0139 | MBIO201.4 | HM034278 |
| Holocentridae | *Sargocentron tiere* | French Polynesia:Society Islands:Moorea | MNHN:Ich:BIOCODES605 | MBIO902.4 | HM034277 |
| Holocentridae | *Sargocentron tiere* | French Polynesia:Society Islands:Tetiaroa | MNHN:Ich:2009-1657 | MOCNESS M9.1-M0-17 | HM034166 |
| Holocentridae | *Sargocentron tiere* | French Polynesia:Society Islands:Tetiaroa | MNHN:Ich:2009-1658 | MOCNESS M9.1-M0-18 | HM034167 |
| Holocentridae | *Sargocentron tiere* | French Polynesia:Society Islands:Tetiaroa | MNHN:Ich:2009-1659 | MOCNESS M9.1-M0-19 | HM034168 |
| Holocentridae | *Sargocentron tiere* | French Polynesia:Society Islands:Tetiaroa | MNHN:Ich:2009-1660 | MOCNESS M9.1-M0-22 | HM034169 |
| Holocentridae | *Sargocentron tiere* | French Polynesia:Society Islands:Tetiaroa | MNHN:Ich:2009-1661 | MOCNESS M9.1-M0-24 | HM034170 |
| Holocentridae | *Sargocentron tiere* | French Polynesia:Society Islands:Tetiaroa | MNHN:Ich:2009-1662 | MOCNESS M9.1-M0-25 | HM034171 |
| Holocentridae | *Sargocentron tiere* | French Polynesia:Society Islands:Tetiaroa | MNHN:Ich:2009-1663 | MOCNESS M9.1-M0-26 | HM034172 |
| Holocentridae | *Sargocentron tiere* | French Polynesia:Society Islands:Tetiaroa | MNHN:Ich:2009-1664 | MOCNESS M9.1-M0-27 | HM034173 |
| Holocentridae | *Sargocentron tiere* | French Polynesia:Society Islands:Tetiaroa | MNHN:Ich:2009-1665 | MOCNESS M9.1-M0-29 | HM034174 |
| Holocentridae | *Sargocentron tiere* | French Polynesia:Society Islands:Tetiaroa | MNHN:Ich:2009-1666 | MOCNESS M9.1-M0-30 | HM034175 |
| Holocentridae | *Sargocentron tiere* | Reunion:West:St Leu | ECOMAR:Ich:REU0965 | REU0965 | JQ350322 |
| Holocentridae | *Sargocentron tiere* | Reunion:West:St Leu | ECOMAR:Ich:REU0966 | REU0966 | JQ350321 |
| Holocentridae | *Sargocentron tiere* | Reunion:West:St Leu | ECOMAR:Ich:REU0967 | REU0967 | JQ350323 |
| Holocentridae | *Sargocentron tiere* | Reunion:West:St Leu | ECOMAR:Ich:REU0968 | REU0968 | JQ350320 |
| Holocentridae | *Sargocentron tiereoides* | French Polynesia:Society Islands:Moorea | SI:Ich:MBIO658 | MBIO658.4 | HM034281 |
| Holocentridae | *Sargocentron tiereoides* | French Polynesia:Society Islands:Moorea | MNHN:Ich:BIOCODES606 | MBIO901.4 | HM034280 |
| Holocentridae | *Sargocentron tiereoides* | French Polynesia:Society Islands:Tetiaroa | MNHN:Ich:2009-1667 | MOCNESS M9.1-M0-20 | HM034176 |
| Holocentridae | *Sargocentron violaceum* | Madagascar:West:Nosy Be | ECOMAR:Ich:NBE0083 | NBE0083 | JQ350324 |
| Holocentridae | *Sargocentron violaceum* | Madagascar:West:Nosy Be | ECOMAR:Ich:NBE1065 | NBE1065 | JQ350325 |
| Kuhliidae | *Kuhlia malo* | French Polynesia:Society Islands:Moorea | MNHN:Ich:BIOCODES1012 | MBIO1801.4 | JQ431871 |
| Kuhliidae | *Kuhlia malo* | French Polynesia:Society Islands:Moorea | MNHN:Ich:BIOCODES1008 | MBIO1802.4 | JQ431870 |
| Kuhliidae | *Kuhlia mugil* | Reunion:West:St Gilles | ECOMAR:Ich:REU1684 | REU1684 | JQ350072 |
| Kuhliidae | *Kuhlia mugil* | Reunion:South:St Philippe | ECOMAR:Ich:REU1836 | REU1836 | JQ350074 |
| Kuhliidae | *Kuhlia mugil* | Reunion:South:St Philippe | ECOMAR:Ich:REU1837 | REU1837 | JQ350073 |
| Kuhliidae | *Kuhlia rupestris* | Reunion:South:St Philippe | ECOMAR:Ich:REU1776 | REU1776 | JQ350075 |
| Kuhliidae | *Kuhlia rupestris* | Reunion:South:St Philippe | ECOMAR:Ich:REU1778 | REU1778 | JQ350078 |
| Kuhliidae | *Kuhlia rupestris* | Reunion:South:St Philippe | ECOMAR:Ich:REU1838 | REU1838 | JQ350077 |
| Kuhliidae | *Kuhlia rupestris* | Reunion:South:St Philippe | ECOMAR:Ich:REU1839 | REU1839 | JQ350076 |
| Kuhliidae | *Kuhlia sandvicensis* | French Polynesia:Society Islands:Moorea | MNHN:Ich:BIOCODES547 | MBIO806.4 | JQ431872 |
| Kuhliidae | *Kuhlia sandvicensis* | French Polynesia:Society Islands:Moorea | MNHN:Ich:BIOCODES544 | MBIO807.4 | JQ431873 |
| Kyphosidae | *Kyphosus cinerascens* | Madagascar:West:Nosy Be | ECOMAR:Ich:NBE1082 | NBE1082 | JQ350079 |
| Kyphosidae | *Kyphosus vaigiensis* | French Polynesia:Society Islands:Moorea | MNHN:Ich:BIOCODES0142 | MBIO207.4 | JQ431874 |
| Labridae | *Anampses caeruleopunctatus* | French Polynesia:Society Islands:Moorea | MNHN:Ich:BIOCODES1037 | MBIO1849.4 | JQ431410 |
| Labridae | *Anampses caeruleopunctatus* | Madagascar:West:Nosy Be | ECOMAR:Ich:NBE0639 | NBE0639 | JF434738 |
| Labridae | *Anampses caeruleopunctatus* | Reunion:West:St Gilles | ECOMAR:Ich:REU0733 | REU0733 | JQ349696 |
| Labridae | *Anampses caeruleopunctatus* | Reunion:West:St Leu | ECOMAR:Ich:REU0919 | REU0919 | JF434739 |
| Labridae | *Anampses lineatus* | Reunion:West:St Leu | ECOMAR:Ich:REU0922 | REU0922 | JF434740 |
| Labridae | *Anampses twistii* | French Polynesia:Society Islands:Moorea | MNHN:Ich:BIOCODES799 | MBIO1247.4 | JQ431412 |
| Labridae | *Anampses twistii* | French Polynesia:Society Islands:Moorea | SI:Ich:MBIO1552 | MBIO1552.4 | JQ431411 |
| Labridae | *Anampses meleagrides* | Madagascar:West:Nosy Be | ECOMAR:Ich:NBE0637 | NBE0637 | JF434745 |
| Labridae | *Anampses meleagrides* | Madagascar:West:Nosy Be | ECOMAR:Ich:NBE1125 | NBE1125 | JF434744 |
| Labridae | *Anampses meleagrides* | Madagascar:West:Nosy Be | ECOMAR:Ich:NBE1286 | NBE1286 | JF434743 |
| Labridae | *Anampses meleagrides* | Madagascar:West:Nosy Be | ECOMAR:Ich:NBE1287 | NBE1287 | JF434742 |
| Labridae | *Anampses meleagrides* | Madagascar:West:Nosy Be | ECOMAR:Ich:NBE1288 | NBE1288 | JF434741 |
| Labridae | *Bodianus anthioides* | Madagascar:West:Nosy Be | ECOMAR:Ich:NBE1113 | NBE1113 | JF434747 |
| Labridae | *Bodianus anthioides* | Madagascar:West:Nosy Be | ECOMAR:Ich:NBE1114 | NBE1114 | JF434746 |
| Labridae | *Bodianus anthioides* | Reunion:West:St Gilles | ECOMAR:Ich:REU0734 | REU0734 | JQ349800 |
| Labridae | *Bodianus axillaris* | French Polynesia:Society Islands:Moorea | MNHN:Ich:BIOCODES824 | MBIO1296.4 | JQ431488 |
| Labridae | *Bodianus axillaris* | Madagascar:West:Nosy Be | ECOMAR:Ich:NBE0074 | NBE0074 | JF434754 |
| Labridae | *Bodianus axillaris* | Madagascar:West:Nosy Be | ECOMAR:Ich:NBE0075 | NBE0075 | JF434753 |
| Labridae | *Bodianus axillaris* | Madagascar:West:Nosy Be | ECOMAR:Ich:NBE0356 | NBE0356 | JF434752 |
| Labridae | *Bodianus axillaris* | Reunion:West:Hermitage | ECOMAR:Ich:REU0735 | REU0735 | JF434751 |
| Labridae | *Bodianus axillaris* | Reunion:West:Hermitage | ECOMAR:Ich:REU0775 | REU0775 | JF434750 |
| Labridae | *Bodianus axillaris* | Reunion:West:St Leu | ECOMAR:Ich:REU0995 | REU0995 | JF434749 |
| Labridae | *Bodianus axillaris* | Reunion:West:St Leu | ECOMAR:Ich:REU1617 | REU1617 | JF434748 |
| Labridae | *Bodianus diana* | Madagascar:West:Nosy Be | ECOMAR:Ich:NBE0365 | NBE0365 | JF434759 |
| Labridae | *Bodianus diana* | Madagascar:West:Nosy Be | ECOMAR:Ich:NBE1115 | NBE1115 | JF434758 |
| Labridae | *Bodianus diana* | Madagascar:West:Nosy Be | ECOMAR:Ich:NBE1116 | NBE1116 | JF434757 |
| Labridae | *Bodianus diana* | Madagascar:West:Nosy Be | ECOMAR:Ich:NBE1117 | NBE1117 | JF434756 |
| Labridae | *Bodianus diana* | Madagascar:West:Nosy Be | ECOMAR:Ich:NBE1118 | NBE1118 | JF434755 |
| Labridae | *Bodianus perditio* | Madagascar:West:Nosy Be | ECOMAR:Ich:NBE1140 | NBE1140 | JF434760 |
| Labridae | *Cheilinus chlorourus* | French Polynesia:Society Islands:Moorea | MNHN:Ich:2008-908 | MBIO1189 | JF434849 |
| Labridae | *Cheilinus chlorourus* | French Polynesia:Society Islands:Moorea | MNHN:Ich:BIOCODES712 | MBIO1189.4 | JQ431603 |
| Labridae | *Cheilinus chlorourus* | French Polynesia:Society Islands:Moorea | MNHN:Ich:MBIO1190 | MBIO1190 | JF434848 |
| Labridae | *Cheilinus chlorourus* | French Polynesia:Society Islands:Moorea | SI:Ich:MBIO1190 | MBIO1190.4 | JQ431602 |
| Labridae | *Cheilinus chlorourus* | Reunion:West:Hermitage | ECOMAR:Ich:REU146-2 | REU146-2 | JF434847 |
| Labridae | *Cheilinus fasciatus* | Madagascar:West:Nosy Be | ECOMAR:Ich:NBE0203 | NBE0203 | JF434856 |
| Labridae | *Cheilinus fasciatus* | Madagascar:West:Nosy Be | ECOMAR:Ich:NBE0478 | NBE0478 | JF434855 |
| Labridae | *Cheilinus fasciatus* | Madagascar:West:Nosy Be | ECOMAR:Ich:NBE0479 | NBE0479 | JF434854 |
| Labridae | *Cheilinus fasciatus* | Madagascar:West:Nosy Be | ECOMAR:Ich:NBE0480 | NBE0480 | JF434853 |
| Labridae | *Cheilinus fasciatus* | Madagascar:West:Nosy Be | ECOMAR:Ich:NBE0625 | NBE0625 | JF434852 |
| Labridae | *Cheilinus fasciatus* | Madagascar:West:Nosy Be | ECOMAR:Ich:NBE0626 | NBE0626 | JF434851 |
| Labridae | *Cheilinus fasciatus* | Madagascar:West:Nosy Be | ECOMAR:Ich:NBE0627 | NBE0627 | JQ349878 |
| Labridae | *Cheilinus fasciatus* | Madagascar:West:Nosy Be | ECOMAR:Ich:NBE1017 | NBE1017 | JF434850 |
| Labridae | *Cheilinus oxycephalus* | French Polynesia:Society Islands:Moorea | SI:Ich:MBIO1585 | MBIO1585.4 | JQ431604 |
| Labridae | *Cheilinus oxycephalus* | Madagascar:West:Nosy Be | ECOMAR:Ich:NBE0049 | NBE0049 | JF434858 |
| Labridae | *Cheilinus oxycephalus* | Madagascar:West:Nosy Be | ECOMAR:Ich:NBE0050 | NBE0050 | JF434857 |
| Labridae | *Cheilinus oxycephalus* | Madagascar:West:Nosy Be | ECOMAR:Ich:NBE1014 | NBE1014 | JQ349879 |
| Labridae | *Cheilinus trilobatus* | French Polynesia:Society Islands:Moorea | MNHN:Ich:BIOCODES0272 | MBIO395.4 | JQ431606 |
| Labridae | *Cheilinus trilobatus* | French Polynesia:Society Islands:Moorea | MNHN:Ich:BIOCODES386 | MBIO550.4 | JQ431607 |
| Labridae | *Cheilinus trilobatus* | French Polynesia:Society Islands:Moorea | MNHN:Ich:BIOCODES382 | MBIO551.4 | JQ431605 |
| Labridae | *Cheilinus trilobatus* | Madagascar:West:Nosy Be | ECOMAR:Ich:NBE0652 | NBE0652 | JQ349881 |
| Labridae | *Cheilinus trilobatus* | Reunion:West:Hermitage | ECOMAR:Ich:REU0736 | REU0736 | JF434860 |
| Labridae | *Cheilinus trilobatus* | Reunion:West:St Gilles | ECOMAR:Ich:REU146-3 | REU146_3 | JQ349880 |
| Labridae | *Cheilinus trilobatus* | Reunion:West:Hermitage | ECOMAR:Ich:REU146-1 | REU146-1 | JF434859 |
| Labridae | *Cheilio inermis* | French Polynesia:Society Islands:Moorea | MNHN:Ich:BIOCODES0878 | MBIO1456.4 | JQ431608 |
| Labridae | *Cheilio inermis* | French Polynesia:Society Islands:Moorea | SI:Ich:MBIO1457 | MBIO1457.4 | JQ431609 |
| Labridae | *Cheilio inermis* | Madagascar:West:Nosy Be | ECOMAR:Ich:NBE0048 | NBE0048 | JF434863 |
| Labridae | *Cheilio inermis* | Madagascar:West:Nosy Be | ECOMAR:Ich:NBE1424 | NBE1424 | JF434862 |
| Labridae | *Cheilio inermis* | Madagascar:West:Nosy Be | ECOMAR:Ich:NBE1425 | NBE1425 | JF434861 |
| Labridae | *Cirrhilabrus exquisitus* | French Polynesia:Society Islands:Moorea | MNHN:Ich:2008-812 | MBIO0991 | JF434935 |
| Labridae | *Cirrhilabrus scottorum* | French Polynesia:Society Islands:Moorea | MNHN:Ich:2008-342 | MBIO0275 | JF434937 |
| Labridae | *Cirrhilabrus scottorum* | French Polynesia:Society Islands:Moorea | MNHN:Ich:MBIO276 | MBIO0276 | JF434936 |
| Labridae | *Cirrhilabrus scottorum* | French Polynesia:Society Islands:Moorea | MNHN:Ich:BIOCODES0072 | MBIO117.4 | JQ431638 |
| Labridae | *Cirrhilabrus scottorum* | French Polynesia:Society Islands:Moorea | MNHN:Ich:BIOCODES0073 | MBIO118.4 | JQ431640 |
| Labridae | *Cirrhilabrus scottorum* | French Polynesia:Society Islands:Moorea | MNHN:Ich:BIOCODES675 | MBIO990.4 | JQ431639 |
| Labridae | *Coris aygula* | French Polynesia:Society Islands:Moorea | MNHN:Ich:BIOCODES0399 | MBIO569.4 | JQ431654 |
| Labridae | *Coris aygula* | French Polynesia:Society Islands:Moorea | SI:Ich:MBIO570 | MBIO570.4 | JQ431655 |
| Labridae | *Coris aygula* | French Polynesia:Society Islands:Moorea | MNHN:Ich:BIOCODES0427 | MBIO608.4 | JQ431657 |
| Labridae | *Coris aygula* | French Polynesia:Society Islands:Moorea | MNHN:Ich:BIOCODES480 | MBIO713.4 | JQ431656 |
| Labridae | *Coris aygula* | French Polynesia:Society Islands:Moorea | SI:Ich:MBIO714 | MBIO714.4 | JQ431658 |
| Labridae | *Coris aygula* | Madagascar:West:Nosy Be | ECOMAR:Ich:NBE0362 | NBE0362 | JF434939 |
| Labridae | *Coris aygula* | Reunion:West:Hermitage | ECOMAR:Ich:REU0765 | REU0765 | JF434938 |
| Labridae | *Coris caudimacula* | Madagascar:West:Nosy Be | ECOMAR:Ich:NBE0366 | NBE0366 | JF434943 |
| Labridae | *Coris caudimacula* | Madagascar:West:Nosy Be | ECOMAR:Ich:NBE0367 | NBE0367 | JF434942 |
| Labridae | *Coris caudimacula* | Madagascar:West:Nosy Be | ECOMAR:Ich:NBE1285 | NBE1285 | JF434941 |
| Labridae | *Coris caudimacula* | Reunion:West:Hermitage | ECOMAR:Ich:REU0784 | REU0784 | JF434940 |
| Labridae | *Coris cuvieri* | Madagascar:West:Nosy Be | ECOMAR:Ich:NBE0379 | NBE0379 | JF434947 |
| Labridae | *Coris cuvieri* | Madagascar:West:Nosy Be | ECOMAR:Ich:NBE0380 | NBE0380 | JF434946 |
| Labridae | *Coris cuvieri* | Madagascar:West:Nosy Be | ECOMAR:Ich:NBE0381 | NBE0381 | JF434945 |
| Labridae | *Coris cuvieri* | Reunion:West:Hermitage | ECOMAR:Ich:REU0770 | REU0770 | JF434944 |
| Labridae | *Coris gaimard* | French Polynesia:Society Islands:Moorea | MNHN:Ich:2008-599 | MBIO0665 | JF434948 |
| Labridae | *Coris gaimard* | French Polynesia:Society Islands:Moorea | MNHN:Ich:BIOCODES0456 | MBIO665.4 | JQ431659 |
| Labridae | *Epibulus insidiator* | French Polynesia:Society Islands:Moorea | MNHN:Ich:BIOCODES387 | MBIO553.4 | JQ431716 |
| Labridae | *Epibulus insidiator* | French Polynesia:Society Islands:Moorea | MNHN:Ich:BIOCODES385 | MBIO554.4 | JQ431715 |
| Labridae | *Epibulus insidiator* | Madagascar:West:Nosy Be | ECOMAR:Ich:NBE0027 | NBE0027 | JF434969 |
| Labridae | *Epibulus insidiator* | Madagascar:West:Nosy Be | ECOMAR:Ich:NBE0028 | NBE0028 | JF434968 |
| Labridae | *Epibulus insidiator* | Madagascar:West:Nosy Be | ECOMAR:Ich:NBE0076 | NBE0076 | JF434967 |
| Labridae | *Epibulus insidiator* | Madagascar:West:Nosy Be | ECOMAR:Ich:NBE1018 | NBE1018 | JF434966 |
| Labridae | *Epibulus insidiator* | Reunion:West:St Leu | ECOMAR:Ich:REU2619 | REU2619 | JF434965 |
| Labridae | *Gomphosus caeruleus* | Madagascar:West:Nosy Be | ECOMAR:Ich:NBE0043 | NBE0043 | JF434979 |
| Labridae | *Gomphosus caeruleus* | Reunion:West:Hermitage | ECOMAR:Ich:REU0110 | REU0110 | JF434978 |
| Labridae | *Gomphosus caeruleus* | Reunion:West:Hermitage | ECOMAR:Ich:REU0750 | REU0750 | JF434977 |
| Labridae | *Gomphosus caeruleus* | Reunion:West:Hermitage | ECOMAR:Ich:REU0751 | REU0751 | JF434976 |
| Labridae | *Gomphosus varius* | French Polynesia:Society Islands:Moorea | MNHN:Ich:2008-674 | MBIO0784 | JF434981 |
| Labridae | *Gomphosus varius* | French Polynesia:Society Islands:Moorea | MNHN:Ich:MBIO785 | MBIO0785 | JF434980 |
| Labridae | *Gomphosus varius* | French Polynesia:Society Islands:Moorea | MNHN:Ich:BIOCODES801 | MBIO1253.4 | JQ431774 |
| Labridae | *Gomphosus varius* | French Polynesia:Society Islands:Moorea | MNHN:Ich:BIOCODES0407 | MBIO577.4 | JQ431773 |
| Labridae | *Gomphosus varius* | French Polynesia:Society Islands:Moorea | MNHN:Ich:BIOCODES0404 | MBIO578.4 | JQ431770 |
| Labridae | *Gomphosus varius* | French Polynesia:Society Islands:Moorea | MNHN:Ich:BIOCODES532 | MBIO784.4 | JQ431771 |
| Labridae | *Gomphosus varius* | French Polynesia:Society Islands:Moorea | SI:Ich:MBIO785 | MBIO785.4 | JQ431772 |
| Labridae | *Halichoeres cosmetus* | Madagascar:West:Nosy Be | ECOMAR:Ich:NBE0363 | NBE0363 | JF434984 |
| Labridae | *Halichoeres cosmetus* | Madagascar:West:Nosy Be | ECOMAR:Ich:NBE0364 | NBE0364 | JF434983 |
| Labridae | *Halichoeres cosmetus* | Reunion:West:Hermitage | ECOMAR:Ich:REU0787 | REU0787 | JF434986 |
| Labridae | *Halichoeres cosmetus* | Reunion:West:Hermitage | ECOMAR:Ich:REU0788 | REU0788 | JF434985 |
| Labridae | *Halichoeres cosmetus* | Reunion:West:St Leu | ECOMAR:Ich:REU1021 | REU1021 | JF434982 |
| Labridae | *Halichoeres hortulanus* | French Polynesia:Society Islands:Moorea | MNHN:Ich:BIOCODES0062 | MBIO104.4 | JQ431840 |
| Labridae | *Halichoeres hortulanus* | French Polynesia:Society Islands:Moorea | MNHN:Ich:BIOCODES0398 | MBIO571.4 | JQ431837 |
| Labridae | *Halichoeres hortulanus* | French Polynesia:Society Islands:Moorea | MNHN:Ich:BIOCODES513 | MBIO759.4 | JQ431839 |
| Labridae | *Halichoeres hortulanus* | French Polynesia:Society Islands:Moorea | MNHN:Ich:BIOCODES514 | MBIO760.4 | JQ431838 |
| Labridae | *Halichoeres hortulanus* | Madagascar:West:Nosy Be | ECOMAR:Ich:NBE0047 | NBE0047 | JF434990 |
| Labridae | *Halichoeres hortulanus* | Madagascar:West:Nosy Be | ECOMAR:Ich:NBE0361 | NBE0361 | JF434989 |
| Labridae | *Halichoeres hortulanus* | Madagascar:West:Nosy Be | ECOMAR:Ich:NBE0647 | NBE0647 | JF434988 |
| Labridae | *Halichoeres hortulanus* | Reunion:West:Hermitage | ECOMAR:Ich:REU0767 | REU0767 | JF434987 |
| Labridae | *Halichoeres margaritaceus* | French Polynesia:Society Islands:Moorea | MNHN:Ich:2008-556 | MBIO0588 | JF434992 |
| Labridae | *Halichoeres margaritaceus* | French Polynesia:Society Islands:Moorea | MNHN:Ich:2008-557 | MBIO0589 | JF434991 |
| Labridae | *Halichoeres margaritaceus* | French Polynesia:Society Islands:Moorea | MNHN:Ich:BIOCODES0412 | MBIO588.4 | JQ431842 |
| Labridae | *Halichoeres margaritaceus* | French Polynesia:Society Islands:Moorea | MNHN:Ich:BIOCODES0411 | MBIO589.4 | JQ431841 |
| Labridae | *Halichoeres marginatus* | French Polynesia:Society Islands:Moorea | MNHN:Ich:BIOCODES826 | MBIO1292.4 | JQ431843 |
| Labridae | *Halichoeres marginatus* | Madagascar:West:Nosy Be | ECOMAR:Ich:NBE0648 | NBE0648 | JF434994 |
| Labridae | *Halichoeres marginatus* | Reunion:West:Hermitage | ECOMAR:Ich:REU0793 | REU0793 | JF434995 |
| Labridae | *Halichoeres marginatus* | Reunion:West:St Leu | ECOMAR:Ich:REU1023 | REU1023 | JF434993 |
| Labridae | *Halichoeres marginatus* | Reunion:West:St Gilles | ECOMAR:Ich:REU107-1 | REU107_1 | JQ350052 |
| Labridae | *Halichoeres marginatus* | Reunion:South:St Philippe | ECOMAR:Ich:REU1693 | REU1693 | JQ350051 |
| Labridae | *Halichoeres nebulosus* | Madagascar:West:Nosy Be | ECOMAR:Ich:NBE0235 | NBE0235 | JF434998 |
| Labridae | *Halichoeres nebulosus* | Madagascar:West:Nosy Be | ECOMAR:Ich:NBE1323 | NBE1323 | JF434997 |
| Labridae | *Halichoeres nebulosus* | Madagascar:West:Nosy Be | ECOMAR:Ich:NBE1325 | NBE1325 | JF434996 |
| Labridae | *Halichoeres nigrescens* | Madagascar:West:Nosy Be | ECOMAR:Ich:NBE0236 | NBE0236 | JF435002 |
| Labridae | *Halichoeres nigrescens* | Madagascar:West:Nosy Be | ECOMAR:Ich:NBE0237 | NBE0237 | JF435001 |
| Labridae | *Halichoeres nigrescens* | Madagascar:West:Nosy Be | ECOMAR:Ich:NBE0238 | NBE0238 | JF435000 |
| Labridae | *Halichoeres nigrescens* | Madagascar:West:Nosy Be | ECOMAR:Ich:NBE1030 | NBE1030 | JF434999 |
| Labridae | *Halichoeres ornatissimus* | French Polynesia:Society Islands:Moorea | MNHN:Ich:BIOCODES0071 | MBIO115.4 | JQ431844 |
| Labridae | *Halichoeres ornatissimus* | French Polynesia:Society Islands:Moorea | MNHN:Ich:2008-959 | MBIO1288 | JF435004 |
| Labridae | *Halichoeres ornatissimus* | French Polynesia:Society Islands:Moorea | SI:Ich:MBIO1381 | MBIO1381.4 | JQ431845 |
| Labridae | *Halichoeres ornatissimus* | French Polynesia:Society Islands:Moorea | MNHN:Ich:MBIO1382 | MBIO1382 | JF435003 |
| Labridae | *Halichoeres scapularis* | Madagascar:West:Nosy Be | ECOMAR:Ich:NBE1266 | NBE1266 | JF435010 |
| Labridae | *Halichoeres scapularis* | Madagascar:West:Nosy Be | ECOMAR:Ich:NBE1267 | NBE1267 | JF435009 |
| Labridae | *Halichoeres scapularis* | Madagascar:West:Nosy Be | ECOMAR:Ich:NBE1268 | NBE1268 | JF435008 |
| Labridae | *Halichoeres scapularis* | Reunion:West:Hermitage | ECOMAR:Ich:REU013-1 | REU013-1 | JF435007 |
| Labridae | *Halichoeres scapularis* | Reunion:West:Hermitage | ECOMAR:Ich:REU074-1 | REU074-1 | JF435006 |
| Labridae | *Halichoeres scapularis* | Reunion:West:Hermitage | ECOMAR:Ich:REU074-2 | REU074-2 | JF435005 |
| Labridae | *Halichoeres trimaculatus* | French Polynesia:Society Islands:Moorea | MNHN:Ich:2008-537 | MBIO0561 | JF435012 |
| Labridae | *Halichoeres trimaculatus* | French Polynesia:Society Islands:Moorea | MNHN:Ich:2008-538 | MBIO0562 | JF435011 |
| Labridae | *Halichoeres trimaculatus* | French Polynesia:Society Islands:Moorea | MNHN:Ich:BIOCODES535 | MBIO789.4 | JQ431846 |
| Labridae | *Halichoeres trimaculatus* | French Polynesia:Society Islands:Moorea | MNHN:Ich:BIOCODES536 | MBIO790.4 | JQ431847 |
| Labridae | *Hemigymnus fasciatus* | French Polynesia:Society Islands:Moorea | SI:Ich:MBIO1252 | MBIO1252.4 | JQ431848 |
| Labridae | *Hemigymnus fasciatus* | French Polynesia:Society Islands:Moorea | MNHN:Ich:BIOCODES630 | MBIO933.4 | JQ431849 |
| Labridae | *Hemigymnus fasciatus* | Madagascar:West:Nosy Be | ECOMAR:Ich:NBE0204 | NBE0204 | JF435016 |
| Labridae | *Hemigymnus fasciatus* | Madagascar:West:Nosy Be | ECOMAR:Ich:NBE1299 | NBE1299 | JF435015 |
| Labridae | *Hemigymnus fasciatus* | Reunion:West:Hermitage | ECOMAR:Ich:REU0743 | REU0743 | JF435014 |
| Labridae | *Hemigymnus fasciatus* | Reunion:West:Hermitage | ECOMAR:Ich:REU0744 | REU0744 | JF435013 |
| Labridae | *Hemigymnus melapterus* | Madagascar:West:Nosy Be | ECOMAR:Ich:NBE0458 | NBE0458 | JF435018 |
| Labridae | *Hemigymnus melapterus* | Madagascar:West:Nosy Be | ECOMAR:Ich:NBE0459 | NBE0459 | JF435017 |
| Labridae | *Hologymnosus annulatus* | Madagascar:West:Nosy Be | ECOMAR:Ich:NBE0377 | NBE0377 | JF435038 |
| Labridae | *Hologymnosus annulatus* | Madagascar:West:Nosy Be | ECOMAR:Ich:NBE1309 | NBE1309 | JF435037 |
| Labridae | *Hologymnosus annulatus* | Madagascar:West:Nosy Be | ECOMAR:Ich:NBE1310 | NBE1310 | JF435036 |
| Labridae | *Labroides bicolor* | French Polynesia:Society Islands:Moorea | MNHN:Ich:2008-559 | MBIO0592 | JF435041 |
| Labridae | *Labroides bicolor* | French Polynesia:Society Islands:Moorea | MNHN:Ich:BIOCODES0414 | MBIO592.4 | JQ431875 |
| Labridae | *Labroides bicolor* | Madagascar:West:Nosy Be | ECOMAR:Ich:NBE1133 | NBE1133 | JF435039 |
| Labridae | *Labroides bicolor* | Reunion:West:Hermitage | ECOMAR:Ich:REU0786 | REU0786 | JF435040 |
| Labridae | *Labroides dimidiatus* | French Polynesia:Society Islands:Moorea | MNHN:Ich:BIOCODES0410 | MBIO581.4 | JQ431876 |
| Labridae | *Labroides dimidiatus* | French Polynesia:Society Islands:Moorea | MNHN:Ich:BIOCODES0403 | MBIO582.4 | JQ431878 |
| Labridae | *Labroides dimidiatus* | French Polynesia:Society Islands:Moorea | MNHN:Ich:BIOCODES651 | MBIO960.4 | JQ431877 |
| Labridae | *Labroides dimidiatus* | Madagascar:West:Nosy Be | ECOMAR:Ich:NBE0113 | NBE0113 | JQ350083 |
| Labridae | *Labroides dimidiatus* | Madagascar:West:Nosy Be | ECOMAR:Ich:NBE0114 | NBE0114 | JQ350082 |
| Labridae | *Labroides dimidiatus* | Madagascar:West:Nosy Be | ECOMAR:Ich:NBE0115 | NBE0115 | JQ350080 |
| Labridae | *Labroides dimidiatus* | Madagascar:West:Nosy Be | ECOMAR:Ich:NBE0226 | NBE0226 | JF435043 |
| Labridae | *Labroides dimidiatus* | Madagascar:West:Nosy Be | ECOMAR:Ich:NBE0584 | NBE0584 | JQ350081 |
| Labridae | *Labroides dimidiatus* | Reunion:West:St Leu | ECOMAR:Ich:REU1616 | REU1616 | JF435042 |
| Labridae | *Labropsis polynesica* | French Polynesia:Society Islands:Moorea | MNHN:Ich:2008-927 | MBIO1226 | JF435045 |
| Labridae | *Labropsis polynesica* | French Polynesia:Society Islands:Moorea | MNHN:Ich:MBIO1765 | MBIO1765 | JF435044 |
| Labridae | *Macropharyngodon bipartitus bipartitus* | Madagascar:West:Nosy Be | ECOMAR:Ich:NBE1122 | NBE1122 | JF435048 |
| Labridae | *Macropharyngodon bipartitus bipartitus* | Madagascar:West:Nosy Be | ECOMAR:Ich:NBE1123 | NBE1123 | JF435047 |
| Labridae | *Macropharyngodon bipartitus bipartitus* | Madagascar:West:Nosy Be | ECOMAR:Ich:NBE1124 | NBE1124 | JF435046 |
| Labridae | *Macropharyngodon bipartitus bipartitus* | Reunion:West:St Leu | ECOMAR:Ich:REU0918 | REU0918 | JF435049 |
| Labridae | *Macropharyngodon meleagris* | French Polynesia:Society Islands:Moorea | MNHN:Ich:2008-1157 | MBIO1847 | JF435051 |
| Labridae | *Macropharyngodon meleagris* | French Polynesia:Society Islands:Moorea | MNHN:Ich:MBIO1848 | MBIO1848 | JF435050 |
| Labridae | *Novaculichthys taeniourus* | French Polynesia:Society Islands:Moorea | MNHN:Ich:2008-956 | MBIO1282 | JF435061 |
| Labridae | *Novaculichthys taeniourus* | French Polynesia:Society Islands:Moorea | MNHN:Ich:MBIO1283 | MBIO1283 | JF435060 |
| Labridae | *Oxycheilinus arenatus* | French Polynesia:Society Islands:Moorea | MNHN:Ich:2008-1085 | MBIO1614 | JF435062 |
| Labridae | *Oxycheilinus bimaculatus* | French Polynesia:Society Islands:Moorea | MNHN:Ich:BIOCODES798 | MBIO1245.4 | JQ431954 |
| Labridae | *Oxycheilinus bimaculatus* | French Polynesia:Society Islands:Moorea | SI:Ich:MBIO1246 | MBIO1246.4 | JQ431953 |
| Labridae | *Oxycheilinus digramma* | Madagascar:West:Nosy Be | ECOMAR:Ich:NBE0026 | NBE0026 | JF435066 |
| Labridae | *Oxycheilinus digramma* | Madagascar:West:Nosy Be | ECOMAR:Ich:NBE0077 | NBE0077 | JF435065 |
| Labridae | *Oxycheilinus digramma* | Madagascar:West:Nosy Be | ECOMAR:Ich:NBE0202 | NBE0202 | JF435064 |
| Labridae | *Oxycheilinus digramma* | Madagascar:West:Nosy Be | ECOMAR:Ich:NBE0615 | NBE0615 | JF435063 |
| Labridae | *Oxycheilinus unifasciatus* | French Polynesia:Society Islands:Moorea | MNHN:Ich:2008-943 | MBIO1257 | JF435068 |
| Labridae | *Oxycheilinus unifasciatus* | French Polynesia:Society Islands:Moorea | MNHN:Ich:MBIO1345 | MBIO1345 | JF435067 |
| Labridae | *Polylepion russelli* | French Polynesia:Society Islands:Moorea | MNHN:Ich:2008-1173 | MBIO1871 | JF435093 |
| Labridae | *Polylepion russelli* | French Polynesia:Society Islands:Moorea | MNHN:Ich:BIOCODES1049 | MBIO1871.4 | JQ432026 |
| Labridae | *Pseudocheilinus evanidus* | French Polynesia:Society Islands:Moorea | MNHN:Ich:2008-536 | MBIO0560 | JF435139 |
| Labridae | *Pseudocheilinus hexataenia* | French Polynesia:Society Islands:Moorea | MNHN:Ich:BIOCODES0074 | MBIO120.4 | JQ432060 |
| Labridae | *Pseudocheilinus hexataenia* | French Polynesia:Society Islands:Moorea | MNHN:Ich:BIOCODES0075 | MBIO121.4 | JQ432059 |
| Labridae | *Pseudocheilinus hexataenia* | French Polynesia:Society Islands:Moorea | MNHN:Ich:BIOCODES527 | MBIO779.4 | JQ432058 |
| Labridae | *Pseudocheilinus hexataenia* | French Polynesia:Society Islands:Moorea | MNHN:Ich:BIOCODES528 | MBIO780.4 | JQ432057 |
| Labridae | *Pseudocheilinus hexataenia* | Madagascar:West:Nosy Be | ECOMAR:Ich:NBE0412 | NBE0412 | JF435141 |
| Labridae | *Pseudocheilinus hexataenia* | Madagascar:West:Nosy Be | ECOMAR:Ich:NBE0413 | NBE0413 | JQ350265 |
| Labridae | *Pseudocheilinus hexataenia* | Madagascar:West:Nosy Be | ECOMAR:Ich:NBE0414 | NBE0414 | JF435140 |
| Labridae | *Pseudocheilinus hexataenia* | Madagascar:West:Nosy Be | ECOMAR:Ich:NBE0415 | NBE0415 | JQ350266 |
| Labridae | *Pseudocheilinus ocellatus* | French Polynesia:Society Islands:Moorea | MNHN:Ich:2008-810 | MBIO0989 | JF435142 |
| Labridae | *Pseudocheilinus octotaenia* | French Polynesia:Society Islands:Moorea | SI:Ich:MBIO1412 | MBIO1412.4 | JQ432061 |
| Labridae | *Pseudocheilinus octotaenia* | French Polynesia:Society Islands:Moorea | MNHN:Ich:BIOCODES0042 | MBIO75.4 | JQ432062 |
| Labridae | *Pseudocheilinus octotaenia* | Reunion:West:Hermitage | ECOMAR:Ich:REU0785 | REU0785 | JF435144 |
| Labridae | *Pseudocheilinus octotaenia* | Reunion:West:St Leu | ECOMAR:Ich:REU0920 | REU0920 | JF435145 |
| Labridae | *Pseudocheilinus octotaenia* | Reunion:West:St Leu | ECOMAR:Ich:REU1015 | REU1015 | JF435143 |
| Labridae | *Pseudocheilinus tetrataenia* | French Polynesia:Society Islands:Moorea | MNHN:Ich:2008-215 | MBIO0106 | JF435147 |
| Labridae | *Pseudocheilinus tetrataenia* | French Polynesia:Society Islands:Moorea | MNHN:Ich:2008-216 | MBIO0107 | JF435146 |
| Labridae | *Pseudocoris aurantiofasciata* | French Polynesia:Society Islands:Moorea | MNHN:Ich:2008-1089 | MBIO1626 | JF435148 |
| Labridae | *Pseudodax moluccanus* | Madagascar:West:Nosy Be | ECOMAR:Ich:NBE0382 | NBE0382 | JF435149 |
| Labridae | *Pseudojuloides atavai* | French Polynesia:Society Islands:Moorea | MNHN:Ich:2008-960 | MBIO1289 | JF435151 |
| Labridae | *Pseudojuloides atavai* | French Polynesia:Society Islands:Moorea | MNHN:Ich:MBIO1549 | MBIO1549 | JF435150 |
| Labridae | *Stethojulis albovittata* | Madagascar:West:Nosy Be | ECOMAR:Ich:NBE0657 | NBE0657 | JQ350375 |
| Labridae | *Stethojulis albovittata* | Madagascar:West:Nosy Be | ECOMAR:Ich:NBE1265 | NBE1265 | JQ350372 |
| Labridae | *Stethojulis albovittata* | Reunion:West:St Leu | ECOMAR:Ich:REU1011 | REU1011 | JF435176 |
| Labridae | *Stethojulis albovittata* | Reunion:West:St Leu | ECOMAR:Ich:REU1012 | REU1012 | JF435175 |
| Labridae | *Stethojulis albovittata* | Reunion:West:St Gilles | ECOMAR:Ich:REU145-2 | REU145_2 | JQ350374 |
| Labridae | *Stethojulis albovittata* | Reunion:West:Hermitage | ECOMAR:Ich:REU145-1 | REU145-1 | JF435174 |
| Labridae | *Stethojulis albovittata* | Reunion:West:St Gilles | ECOMAR:Ich:REU192-1 | REU192_1 | JQ350373 |
| Labridae | *Stethojulis bandanensis* | French Polynesia:Society Islands:Moorea | MNHN:Ich:2008-679 | MBIO0792 | JF435177 |
| Labridae | *Stethojulis bandanensis* | French Polynesia:Society Islands:Moorea | MNHN:Ich:BIOCODES0402 | MBIO574.4 | JQ432170 |
| Labridae | *Stethojulis bandanensis* | French Polynesia:Society Islands:Moorea | SI:Ich:MBIO793 | MBIO793.4 | JQ432171 |
| Labridae | *Stethojulis strigiventer* | Madagascar:West:Nosy Be | ECOMAR:Ich:NBE1002 | NBE1002 | JF435179 |
| Labridae | *Stethojulis strigiventer* | Madagascar:West:Nosy Be | ECOMAR:Ich:NBE1426 | NBE1426 | JF435178 |
| Labridae | *Thalassoma amblycephalum* | French Polynesia:Society Islands:Moorea | MNHN:Ich:BIOCODES0152 | MBIO219.4 | JQ432191 |
| Labridae | *Thalassoma amblycephalum* | French Polynesia:Society Islands:Moorea | SI:Ich:MBIO220 | MBIO220.4 | JQ432190 |
| Labridae | *Thalassoma amblycephalum* | Madagascar:West:Nosy Be | ECOMAR:Ich:NBE0435 | NBE0435 | JF435183 |
| Labridae | *Thalassoma amblycephalum* | Madagascar:West:Nosy Be | ECOMAR:Ich:NBE0673 | NBE0673 | JF435182 |
| Labridae | *Thalassoma amblycephalum* | Madagascar:West:Nosy Be | ECOMAR:Ich:NBE1181 | NBE1181 | JF435181 |
| Labridae | *Thalassoma amblycephalum* | Madagascar:West:Nosy Be | ECOMAR:Ich:NBE1182 | NBE1182 | JF435180 |
| Labridae | *Thalassoma genivittatum* | Reunion:West:Hermitage | ECOMAR:Ich:REU075-1 | REU075-1 | JF435188 |
| Labridae | *Thalassoma genivittatum* | Reunion:West:Hermitage | ECOMAR:Ich:REU0761 | REU0761 | JF435187 |
| Labridae | *Thalassoma genivittatum* | Reunion:West:St Leu | ECOMAR:Ich:REU0921 | REU0921 | JF435189 |
| Labridae | *Thalassoma genivittatum* | Reunion:West:St Leu | ECOMAR:Ich:REU1618 | REU1618 | JF435186 |
| Labridae | *Thalassoma genivittatum* | Reunion:West:St Leu | ECOMAR:Ich:REU1619 | REU1619 | JF435185 |
| Labridae | *Thalassoma genivittatum* | Reunion:West:St Leu | ECOMAR:Ich:REU2627 | REU2627 | JF435184 |
| Labridae | *Thalassoma hardwicke* | French Polynesia:Society Islands:Moorea | MNHN:Ich:BIOCODES0409 | MBIO584.4 | JQ432195 |
| Labridae | *Thalassoma hardwicke* | French Polynesia:Society Islands:Moorea | MNHN:Ich:BIOCODES0405 | MBIO585.4 | JQ432194 |
| Labridae | *Thalassoma hardwicke* | French Polynesia:Society Islands:Moorea | MNHN:Ich:BIOCODES519 | MBIO766.4 | JQ432193 |
| Labridae | *Thalassoma hardwicke* | French Polynesia:Society Islands:Moorea | SI:Ich:MBIO767 | MBIO767.4 | JQ432192 |
| Labridae | *Thalassoma hardwicke* | Madagascar:West:Nosy Be | ECOMAR:Ich:NBE0646 | NBE0646 | JF435196 |
| Labridae | *Thalassoma hardwicke* | Madagascar:West:Nosy Be | ECOMAR:Ich:NBE1277 | NBE1277 | JF435195 |
| Labridae | *Thalassoma hardwicke* | Madagascar:West:Nosy Be | ECOMAR:Ich:NBE1278 | NBE1278 | JF435194 |
| Labridae | *Thalassoma hardwicke* | Madagascar:West:Nosy Be | ECOMAR:Ich:NBE1279 | NBE1279 | JF435193 |
| Labridae | *Thalassoma hardwicke* | Reunion:West:Hermitage | ECOMAR:Ich:REU0111 | REU0111 | JF435192 |
| Labridae | *Thalassoma hardwicke* | Reunion:West:St Leu | ECOMAR:Ich:REU2624 | REU2624 | JF435191 |
| Labridae | *Thalassoma hardwicke* | Reunion:West:St Leu | ECOMAR:Ich:REU2625 | REU2625 | JF435190 |
| Labridae | *Thalassoma hebraicum* | Madagascar:West:Nosy Be | ECOMAR:Ich:NBE0051 | NBE0051 | JF435199 |
| Labridae | *Thalassoma hebraicum* | Madagascar:West:Nosy Be | ECOMAR:Ich:NBE0641 | NBE0641 | JF435198 |
| Labridae | *Thalassoma hebraicum* | Madagascar:West:Nosy Be | ECOMAR:Ich:NBE0642 | NBE0642 | JF435197 |
| Labridae | *Thalassoma hebraicum* | Madagascar:West:Nosy Be | ECOMAR:Ich:NBE1280 | NBE1280 | JQ350393 |
| Labridae | *Thalassoma hebraicum* | Madagascar:West:Nosy Be | ECOMAR:Ich:NBE1281 | NBE1281 | JQ350394 |
| Labridae | *Thalassoma lunare* | Madagascar:West:Nosy Be | ECOMAR:Ich:NBE0033 | NBE0033 | JF435203 |
| Labridae | *Thalassoma lunare* | Madagascar:West:Nosy Be | ECOMAR:Ich:NBE0239 | NBE0239 | JF435202 |
| Labridae | *Thalassoma lunare* | Madagascar:West:Nosy Be | ECOMAR:Ich:NBE0582 | NBE0582 | JQ350396 |
| Labridae | *Thalassoma lunare* | Madagascar:West:Nosy Be | ECOMAR:Ich:NBE1042 | NBE1042 | JQ350395 |
| Labridae | *Thalassoma lunare* | Madagascar:West:Nosy Be | ECOMAR:Ich:NBE1043 | NBE1043 | JQ350398 |
| Labridae | *Thalassoma lunare* | Madagascar:West:Nosy Be | ECOMAR:Ich:NBE1233 | NBE1233 | JF435201 |
| Labridae | *Thalassoma lunare* | Madagascar:West:Nosy Be | ECOMAR:Ich:NBE1234 | NBE1234 | JF435200 |
| Labridae | *Thalassoma lunare* | Madagascar:West:Nosy Be | ECOMAR:Ich:NBE1235 | NBE1235 | JQ350397 |
| Labridae | *Thalassoma lutescens* | French Polynesia:Society Islands:Moorea | MNHN:Ich:2008-639 | MBIO0733 | JF435204 |
| Labridae | *Thalassoma purpureum* | Reunion:South:St Philippe | ECOMAR:Ich:REU1683 | REU1683 | JF435209 |
| Labridae | *Thalassoma purpureum* | Reunion:South:St Philippe | ECOMAR:Ich:REU1755 | REU1755 | JF435208 |
| Labridae | *Thalassoma purpureum* | Reunion:South:St Philippe | ECOMAR:Ich:REU1759 | REU1759 | JF435207 |
| Labridae | *Thalassoma purpureum* | Reunion:South:St Philippe | ECOMAR:Ich:REU1814 | REU1814 | JF435206 |
| Labridae | *Thalassoma purpureum* | Reunion:South:St Philippe | ECOMAR:Ich:REU1815 | REU1815 | JF435205 |
| Labridae | *Thalassoma quinquevittatum* | French Polynesia:Society Islands:Moorea | MNHN:Ich:2008-299 | MBIO0215 | JF435210 |
| Labridae | *Thalassoma trilobatum* | French Polynesia:Society Islands:Moorea | MNHN:Ich:2008-303 | MBIO0221 | JF435212 |
| Labridae | *Thalassoma trilobatum* | French Polynesia:Society Islands:Moorea | MNHN:Ich:2008-647 | MBIO0746 | JF435211 |
| Labridae | *Wetmorella albofasciata* | French Polynesia:Society Islands:Moorea | MNHN:Ich:BIOCODES843 | MBIO1327.4 | JQ432219 |
| Labridae | *Wetmorella nigropinnata* | French Polynesia:Society Islands:Moorea | MNHN:Ich:2008-218 | MBIO0110 | JF435215 |
| Labridae | *Wetmorella nigropinnata* | French Polynesia:Society Islands:Moorea | MNHN:Ich:2008-287 | MBIO0199 | JF435214 |
| Labridae | *Wetmorella nigropinnata* | Madagascar:West:Nosy Be | ECOMAR:Ich:NBE0116 | NBE0116 | JF435213 |
| Lethrinidae | *Gnathodentex aureolineatus* | French Polynesia:Society Islands:Moorea | MNHN:Ich:BIOCODES0337 | MBIO486.4 | JQ431756 |
| Lethrinidae | *Gnathodentex aureolineatus* | French Polynesia:Society Islands:Moorea | MNHN:Ich:BIOCODES0340 | MBIO487.4 | JQ431755 |
| Lethrinidae | *Gnathodentex aureolineatus* | Reunion:West:St Gilles | ECOMAR:Ich:REU0509 | REU0509 | JQ350001 |
| Lethrinidae | *Gnathodentex aureolineatus* | Reunion:West:St Gilles | ECOMAR:Ich:REU0510 | REU0510 | JQ350000 |
| Lethrinidae | *Gnathodentex aureolineatus* | Reunion:West:St Gilles | ECOMAR:Ich:REU071-1 | REU071_1 | JQ350002 |
| Lethrinidae | *Lethrinus harak* | Madagascar:West:Nosy Be | ECOMAR:Ich:NBE0007 | NBE0007 | JQ350085 |
| Lethrinidae | *Lethrinus harak* | Madagascar:West:Nosy Be | ECOMAR:Ich:NBE0233 | NBE0233 | JQ350086 |
| Lethrinidae | *Lethrinus harak* | Madagascar:West:Nosy Be | ECOMAR:Ich:NBE0661 | NBE0661 | JQ350084 |
| Lethrinidae | *Lethrinus lentjan* | Madagascar:West:Nosy Be | ECOMAR:Ich:NBE1312 | NBE1312 | JQ350087 |
| Lethrinidae | *Lethrinus mahsena* | Madagascar:West:Nosy Be | ECOMAR:Ich:NBE1109 | NBE1109 | JQ350089 |
| Lethrinidae | *Lethrinus mahsena* | Madagascar:West:Nosy Be | ECOMAR:Ich:NBE1110 | NBE1110 | JQ350088 |
| Lethrinidae | *Lethrinus olivaceus* | French Polynesia:Society Islands:Moorea | MNHN:Ich:BIOCODES809 | MBIO1269.4 | JQ431885 |
| Lethrinidae | *Monotaxis grandoculis* | French Polynesia:Society Islands:Moorea | MNHN:Ich:BIOCODES0140 | MBIO205.4 | JQ431908 |
| Lethrinidae | *Monotaxis grandoculis* | French Polynesia:Society Islands:Moorea | MNHN:Ich:BIOCODES0322 | MBIO468.4 | JQ431910 |
| Lethrinidae | *Monotaxis grandoculis* | French Polynesia:Society Islands:Moorea | MNHN:Ich:BIOCODES581 | MBIO858.4 | JQ431907 |
| Lethrinidae | *Monotaxis grandoculis* | French Polynesia:Society Islands:Moorea | MNHN:Ich:BIOCODES582 | MBIO859.4 | JQ431909 |
| Lethrinidae | *Monotaxis grandoculis* | Reunion:West:St Gilles | ECOMAR:Ich:REU0789 | REU0789 | JQ350109 |
| Lethrinidae | *Monotaxis* sp*.* | Madagascar:West:Nosy Be | ECOMAR:Ich:NBE0631 | NBE0631 | JQ350110 |
| Lutjanidae | *Aphareus furca* | French Polynesia:Society Islands:Moorea | MNHN:Ich:BIOCODES0866 | MBIO1439.4 | JQ431422 |
| Lutjanidae | *Aphareus furca* | French Polynesia:Society Islands:Moorea | MNHN:Ich:BIOCODES0881 | MBIO1466.4 | JQ431425 |
| Lutjanidae | *Aphareus furca* | French Polynesia:Society Islands:Moorea | MNHN:Ich:BIOCODES0992 | MBIO1708.4 | JQ431424 |
| Lutjanidae | *Aphareus furca* | French Polynesia:Society Islands:Moorea | MNHN:Ich:BIOCODES629 | MBIO930.4 | JQ431423 |
| Lutjanidae | *Aprion virescens* | French Polynesia:Society Islands:Moorea | MNHN:Ich:MBIO1267 | MBIO1267.4 | JQ431458 |
| Lutjanidae | *Etelis carbunculus* | French Polynesia:Society Islands:Moorea | MNHN:Ich:BIOCODES1055 | MBIO1864.4 | JQ431727 |
| Lutjanidae | *Lutjanus bohar* | French Polynesia:Society Islands:Moorea | SI:Ich:MBIO1311 | MBIO1311.4 | JQ431892 |
| Lutjanidae | *Lutjanus bohar* | French Polynesia:Society Islands:Moorea | MNHN:Ich:BIOCODES646 | MBIO952.4 | JQ431893 |
| Lutjanidae | *Lutjanus fulvus* | French Polynesia:Society Islands:Moorea | MNHN:Ich:BIOCODES742 | MBIO1145.4 | JQ431894 |
| Lutjanidae | *Lutjanus fulvus* | French Polynesia:Society Islands:Moorea | MNHN:Ich:BIOCODES740 | MBIO1146.4 | JQ431896 |
| Lutjanidae | *Lutjanus fulvus* | French Polynesia:Society Islands:Moorea | MNHN:Ich:BIOCODES0333 | MBIO478.4 | JQ431895 |
| Lutjanidae | *Lutjanus gibbus* | French Polynesia:Society Islands:Moorea | MNHN:Ich:BIOCODES767 | MBIO1185.4 | JQ431898 |
| Lutjanidae | *Lutjanus gibbus* | French Polynesia:Society Islands:Moorea | SI:Ich:MBIO1622 | MBIO1622.4 | JQ431897 |
| Lutjanidae | *Lutjanus gibbus* | Madagascar:West:Nosy Be | ECOMAR:Ich:NBE1112 | NBE1112 | JQ350092 |
| Lutjanidae | *Lutjanus gibbus* | Madagascar:West:Nosy Be | ECOMAR:Ich:NBE1255 | NBE1255 | JQ350090 |
| Lutjanidae | *Lutjanus gibbus* | Madagascar:West:Nosy Be | ECOMAR:Ich:NBE1258 | NBE1258 | JQ350091 |
| Lutjanidae | *Lutjanus kasmira* | French Polynesia:Society Islands:Moorea | MNHN:Ich:BIOCODES738 | MBIO1127.4 | JQ431899 |
| Lutjanidae | *Lutjanus kasmira* | French Polynesia:Society Islands:Moorea | SI:Ich:MBIO1151 | MBIO1151.4 | JQ431900 |
| Lutjanidae | *Lutjanus kasmira* | Reunion:West:St Gilles | ECOMAR:Ich:REU0722 | REU0722 | JQ350093 |
| Lutjanidae | *Lutjanus kasmira* | Reunion:West:St Gilles | ECOMAR:Ich:REU0723 | REU0723 | JQ350094 |
| Lutjanidae | *Lutjanus monostigma* | Madagascar:West:Nosy Be | ECOMAR:Ich:NBE0006 | NBE0006 | JQ350098 |
| Lutjanidae | *Lutjanus monostigma* | Madagascar:West:Nosy Be | ECOMAR:Ich:NBE0628 | NBE0628 | JQ350097 |
| Lutjanidae | *Lutjanus monostigma* | Madagascar:West:Nosy Be | ECOMAR:Ich:NBE0629 | NBE0629 | JQ350096 |
| Lutjanidae | *Lutjanus monostigma* | Madagascar:West:Nosy Be | ECOMAR:Ich:NBE0630 | NBE0630 | JQ350095 |
| Lutjanidae | *Lutjanus rivulatus* | Madagascar:West:Nosy Be | ECOMAR:Ich:NBE1314 | NBE1314 | JQ350099 |
| Lutjanidae | *Macolor niger* | Madagascar:West:Nosy Be | ECOMAR:Ich:NBE0672 | NBE0672 | JQ350100 |
| Lutjanidae | *Pristipomoides auricilla* | French Polynesia:Society Islands:Moorea | MNHN:Ich:BIOCODES0882 | MBIO1463.4 | JQ432045 |
| Lutjanidae | *Pristipomoides auricilla* | French Polynesia:Society Islands:Moorea | MNHN:Ich:BIOCODES0883 | MBIO1464.4 | JQ432044 |
| Lutjanidae | *Pristipomoides auricilla* | French Polynesia:Society Islands:Moorea | SI:Ich:MBIO1711 | MBIO1711.4 | JQ432043 |
| Lutjanidae | *Pristipomoides filamentosus* | French Polynesia:Society Islands:Moorea | MNHN:Ich:BIOCODES0885 | MBIO1467.4 | JQ432047 |
| Lutjanidae | *Pristipomoides filamentosus* | French Polynesia:Society Islands:Moorea | SI:Ich:MBIO1468 | MBIO1468.4 | JQ432046 |
| Malacanthidae | *Malacanthus brevirostris* | French Polynesia:Society Islands:Moorea | MNHN:Ich:BIOCODES793 | MBIO1237.4 | JQ431902 |
| Malacanthidae | *Malacanthus brevirostris* | French Polynesia:Society Islands:Moorea | SI:Ich:MBIO1837 | MBIO1837.4 | JQ431901 |
| Microdesmidae | *Gunnellichthys monostigma* | French Polynesia:Society Islands:Moorea | MNHN:Ich:BIOCODES0899 | MBIO1494.4 | JQ431780 |
| Microdesmidae | *Gunnellichthys monostigma* | French Polynesia:Society Islands:Moorea | MNHN:Ich:BIOCODES0898 | MBIO1495.4 | JQ431779 |
| Microdesmidae | *Gunnellichthys viridescens* | French Polynesia:Society Islands:Moorea | MNHN:Ich:BIOCODES796 | MBIO1241.4 | JQ431781 |
| Microdesmidae | *Nemateleotris magnifica* | French Polynesia:Society Islands:Moorea | MNHN:Ich:BIOCODES0465 | MBIO675.4 | JQ431931 |
| Microdesmidae | *Nemateleotris magnifica* | French Polynesia:Society Islands:Moorea | MNHN:Ich:BIOCODES0454 | MBIO676.4 | JQ431932 |
| Microdesmidae | *Nemateleotris magnifica* | Madagascar:West:Nosy Be | ECOMAR:Ich:NBE1135 | NBE1135 | JQ350135 |
| Microdesmidae | *Nemateleotris magnifica* | Reunion:West:St Leu | ECOMAR:Ich:REU1016 | REU1016 | JQ350134 |
| Microdesmidae | *Nemateleotris magnifica* | Reunion:West:St Leu | ECOMAR:Ich:REU1017 | REU1017 | JQ350136 |
| Microdesmidae | *Ptereleotris evides* | French Polynesia:Society Islands:Moorea | MNHN:Ich:BIOCODES1035 | MBIO1845.4 | JQ432069 |
| Microdesmidae | *Ptereleotris evides* | Madagascar:West:Nosy Be | ECOMAR:Ich:NBE1184 | NBE1184 | JQ350286 |
| Microdesmidae | *Ptereleotris evides* | Madagascar:West:Nosy Be | ECOMAR:Ich:NBE1289 | NBE1289 | JQ350287 |
| Microdesmidae | *Ptereleotris heteroptera* | Reunion:West:St Gilles | ECOMAR:Ich:REU0782 | REU0782 | JQ350288 |
| Microdesmidae | *Ptereleotris zebra* | French Polynesia:Society Islands:Moorea | MNHN:Ich:BIOCODES1034 | MBIO1843.4 | JQ432070 |
| Microdesmidae | *Ptereleotris zebra* | French Polynesia:Society Islands:Moorea | SI:Ich:MBIO1844 | MBIO1844.4 | JQ432071 |
| Monacanthidae | *Aluterus scriptus* | Madagascar:West:Nosy Be | ECOMAR:Ich:NBE1311 | NBE1311 | JQ349678 |
| Monacanthidae | *Amanses scopas* | French Polynesia:Society Islands:Moorea | MNHN:Ich:BIOCODES805 | MBIO1255.4 | JQ431402 |
| Monacanthidae | *Amanses scopas* | French Polynesia:Society Islands:Moorea | SI:Ich:MBIO1256 | MBIO1256.4 | JQ431403 |
| Monacanthidae | *Amanses scopas* | French Polynesia:Society Islands:Moorea | MNHN:Ich:BIOCODES667 | MBIO988.4 | JQ431401 |
| Monacanthidae | *Cantherhines dumerilii* | French Polynesia:Society Islands:Moorea | MNHN:Ich:BIOCODES803 | MBIO1258.4 | JQ431522 |
| Monacanthidae | *Cantherhines dumerilii* | French Polynesia:Society Islands:Moorea | MNHN:Ich:BIOCODES0348 | MBIO500.4 | JQ431520 |
| Monacanthidae | *Cantherhines dumerilii* | French Polynesia:Society Islands:Moorea | SI:Ich:MBIO692 | MBIO692.4 | JQ431521 |
| Monacanthidae | *Cantherhines dumerilii* | Reunion:West:St Gilles | ECOMAR:Ich:REU0726 | REU0726 | JQ349818 |
| Monacanthidae | *Cantherhines dumerilii* | Reunion:West:St Leu | ECOMAR:Ich:REU2704 | REU2704 | JQ349816 |
| Monacanthidae | *Cantherhines dumerilii* | Reunion:West:St Leu | ECOMAR:Ich:REU2705 | REU2705 | JQ349817 |
| Monacanthidae | *Cantherhines pardalis* | Madagascar:West:Nosy Be | ECOMAR:Ich:NBE0662 | NBE0662 | JQ349820 |
| Monacanthidae | *Cantherhines pardalis* | Madagascar:West:Nosy Be | ECOMAR:Ich:NBE0663 | NBE0663 | JQ349819 |
| Monacanthidae | *Cantherhines pardalis* | Reunion:West:St Gilles | ECOMAR:Ich:REU0727 | REU0727 | JQ349821 |
| Monacanthidae | *Cantherhines pardalis* | Reunion:West:St Gilles | ECOMAR:Ich:REU187-1 | REU187_1 | JQ349822 |
| Monacanthidae | *Cantherhines sandwichiensis* | French Polynesia:Society Islands:Moorea | SI:Ich:MBIO1551 | MBIO1551.4 | JQ431523 |
| Monacanthidae | *Cantherhines sandwichiensis* | French Polynesia:Society Islands:Moorea | MNHN:Ich:BIOCODES0031 | MBIO60.4 | JQ431524 |
| Monacanthidae | *Oxymonacanthus longirostris* | Madagascar:West:Nosy Be | ECOMAR:Ich:NBE1078 | NBE1078 | JQ350157 |
| Monacanthidae | *Oxymonacanthus longirostris* | Madagascar:West:Nosy Be | ECOMAR:Ich:NBE1079 | NBE1079 | JQ350158 |
| Monacanthidae | *Oxymonacanthus longirostris* | Madagascar:West:Nosy Be | ECOMAR:Ich:NBE1080 | NBE1080 | JQ350156 |
| Monacanthidae | *Oxymonacanthus longirostris* | Madagascar:West:Nosy Be | ECOMAR:Ich:NBE1081 | NBE1081 | JQ350154 |
| Monacanthidae | *Oxymonacanthus longirostris* | Reunion:West:St Gilles | ECOMAR:Ich:REU149-1 | REU149_1 | JQ350155 |
| Monacanthidae | *Pervagor aspricaudus* | French Polynesia:Society Islands:Moorea | MNHN:Ich:BIOCODES0043 | MBIO77.4 | JQ431994 |
| Monacanthidae | *Pervagor aspricaudus* | French Polynesia:Society Islands:Moorea | SI:Ich:MBIO78 | MBIO78.4 | JQ431991 |
| Monacanthidae | *Pervagor aspricaudus* | French Polynesia:Society Islands:Moorea | MNHN:Ich:BIOCODES658 | MBIO970.4 | JQ431993 |
| Monacanthidae | *Pervagor aspricaudus* | French Polynesia:Society Islands:Moorea | MNHN:Ich:BIOCODES657 | MBIO971.4 | JQ431992 |
| Monacanthidae | *Pervagor aspricaudus* | Reunion:West:St Leu | ECOMAR:Ich:REU0911 | REU0911 | JQ350205 |
| Monacanthidae | *Pervagor aspricaudus* | Reunion:West:St Leu | ECOMAR:Ich:REU0912 | REU0912 | JQ350209 |
| Monacanthidae | *Pervagor aspricaudus* | Reunion:West:St Leu | ECOMAR:Ich:REU0998 | REU0998 | JQ350206 |
| Monacanthidae | *Pervagor aspricaudus* | Reunion:West:St Leu | ECOMAR:Ich:REU0999 | REU0999 | JQ350207 |
| Monacanthidae | *Pervagor aspricaudus* | Reunion:West:St Leu | ECOMAR:Ich:REU1000 | REU1000 | JQ350208 |
| Monacanthidae | *Pervagor janthinosoma* | Reunion:West:St Gilles | ECOMAR:Ich:REU0506 | REU0506 | JQ350210 |
| Monodactylidae | *Monodactylus argenteus* | Madagascar:West:Nosy Be | ECOMAR:Ich:NBE1068 | NBE1068 | JQ350105 |
| Monodactylidae | *Monodactylus argenteus* | Madagascar:West:Nosy Be | ECOMAR:Ich:NBE1069 | NBE1069 | JQ350106 |
| Monodactylidae | *Monodactylus argenteus* | Madagascar:West:Nosy Be | ECOMAR:Ich:NBE1070 | NBE1070 | JQ350107 |
| Monodactylidae | *Monodactylus argenteus* | Madagascar:West:Nosy Be | ECOMAR:Ich:NBE1071 | NBE1071 | JQ350108 |
| Moringuidae | *Moringua* sp. 1 | French Polynesia:Society Islands:Moorea | MNHN:Ich:BIOCODES0104 | MBIO156.4 | JQ431915 |
| Moringuidae | *Moringua* sp. 1 | French Polynesia:Society Islands:Moorea | MNHN:Ich:BIOCODES0105 | MBIO157.4 | JQ431916 |
| Moringuidae | *Moringua* sp. 1 | French Polynesia:Society Islands:Moorea | SI:Ich:MBIO63 | MBIO63.4 | JQ431914 |
| Moringuidae | *Moringua* sp. 2 | French Polynesia:Society Islands:Moorea | MNHN:Ich:BIOCODES0033 | MBIO62.4 | JQ431917 |
| Moringuidae | *Moringua* sp. 3 | French Polynesia:Society Islands:Moorea | MNHN:Ich:BIOCODES571 | MBIO841.4 | JQ431918 |
| Moringuidae | *Moringua* sp. 3 | French Polynesia:Society Islands:Moorea | MNHN:Ich:BIOCODES570 | MBIO842.4 | JQ431919 |
| Moringuidae | *Moringua* sp. 3 | French Polynesia:Society Islands:Moorea | SI:Ich:MBIO846 | MBIO846.4 | JQ431920 |
| Mugilidae | *Crenimugil crenilabis* | French Polynesia:Society Islands:Moorea | MNHN:Ich:BIOCODES0865 | MBIO1438.4 | JQ431669 |
| Mugilidae | *Crenimugil crenilabis* | French Polynesia:Society Islands:Moorea | SI:Ich:MBIO1645 | MBIO1645.4 | JQ431670 |
| Mugilidae | *Ellochelon vaigiensis* | French Polynesia:Society Islands:Moorea | MNHN:Ich:BIOCODES550 | MBIO809.4 | JQ431702 |
| Mugilidae | *Ellochelon vaigiensis* | French Polynesia:Society Islands:Moorea | MNHN:Ich:BIOCODES545 | MBIO810.4 | JQ431701 |
| Mugilidae | *Moolgarda engeli* | French Polynesia:Society Islands:Moorea | MNHN:Ich:BIOCODES551 | MBIO813.4 | JQ431912 |
| Mugilidae | *Moolgarda engeli* | French Polynesia:Society Islands:Moorea | MNHN:Ich:BIOCODES548 | MBIO814.4 | JQ431911 |
| Mugilidae | *Moolgarda engeli* | French Polynesia:Society Islands:Moorea | MNHN:Ich:BIOCODES560 | MBIO828.4 | JQ431913 |
| Mullidae | *Mulloidichthys flavolineatus* | French Polynesia:Society Islands:Moorea | MNHN:Ich:BIOCODES0307 | MBIO445.4 | JQ431921 |
| Mullidae | *Mulloidichthys flavolineatus* | French Polynesia:Society Islands:Moorea | MNHN:Ich:MBIO472 | MBIO472.4 | JQ431924 |
| Mullidae | *Mulloidichthys flavolineatus* | French Polynesia:Society Islands:Moorea | MNHN:Ich:BIOCODES539 | MBIO795.4 | JQ431923 |
| Mullidae | *Mulloidichthys flavolineatus* | French Polynesia:Society Islands:Moorea | MNHN:Ich:BIOCODES540 | MBIO796.4 | JQ431922 |
| Mullidae | *Mulloidichthys flavolineatus* | Reunion:West:St Gilles | ECOMAR:Ich:REU012-1 | REU012_1 | JQ350112 |
| Mullidae | *Mulloidichthys flavolineatus* | Reunion:West:St Leu | ECOMAR:Ich:REU2639 | REU2639 | JQ350111 |
| Mullidae | *Mulloidichthys vanicolensis* | French Polynesia:Society Islands:Moorea | MNHN:Ich:BIOCODES0958 | MBIO1618.4 | JQ431925 |
| Mullidae | *Mulloidichthys vanicolensis* | French Polynesia:Society Islands:Moorea | SI:Ich:MBIO1619 | MBIO1619.4 | JQ431926 |
| Mullidae | *Mulloidichthys vanicolensis* | Reunion:West:St Gilles | ECOMAR:Ich:REU0507 | REU0507 | JQ350113 |
| Mullidae | *Parupeneus barberinus* | French Polynesia:Society Islands:Moorea | MNHN:Ich:BIOCODES714 | MBIO1194.4 | JQ431976 |
| Mullidae | *Parupeneus barberinus* | French Polynesia:Society Islands:Moorea | MNHN:Ich:BIOCODES765 | MBIO1195.4 | JQ431975 |
| Mullidae | *Parupeneus barberinus* | Madagascar:West:Nosy Be | ECOMAR:Ich:NBE1040 | NBE1040 | JQ350180 |
| Mullidae | *Parupeneus barberinus* | Madagascar:West:Nosy Be | ECOMAR:Ich:NBE1041 | NBE1041 | JQ350179 |
| Mullidae | *Parupeneus bifasciatus* | Reunion:West:St Gilles | ECOMAR:Ich:REU0757 | REU0757 | JQ350181 |
| Mullidae | *Parupeneus bifasciatus* | Reunion:West:St Leu | ECOMAR:Ich:REU1003 | REU1003 | JQ350182 |
| Mullidae | *Parupeneus ciliatus* | French Polynesia:Society Islands:Moorea | MNHN:Ich:BIOCODES766 | MBIO1186.4 | JQ431977 |
| Mullidae | *Parupeneus ciliatus* | French Polynesia:Society Islands:Moorea | SI:Ich:MBIO1187 | MBIO1187.4 | JQ431978 |
| Mullidae | *Parupeneus ciliatus* | Reunion:West:St Gilles | ECOMAR:Ich:REU105-1 | REU105_1 | JQ350183 |
| Mullidae | *Parupeneus cyclostomus* | French Polynesia:Society Islands:Moorea | SI:Ich:MBIO1404 | MBIO1404.4 | JQ431981 |
| Mullidae | *Parupeneus cyclostomus* | French Polynesia:Society Islands:Moorea | MNHN:Ich:MBIO465 | MBIO465.4 | JQ431979 |
| Mullidae | *Parupeneus cyclostomus* | French Polynesia:Society Islands:Moorea | SI:Ich:MBIO466 | MBIO466.4 | JQ431980 |
| Mullidae | *Parupeneus cyclostomus* | Madagascar:West:Nosy Be | ECOMAR:Ich:NBE0351 | NBE0351 | JQ350184 |
| Mullidae | *Parupeneus cyclostomus* | Reunion:West:St Gilles | ECOMAR:Ich:REU0774 | REU0774 | JQ350185 |
| Mullidae | *Parupeneus fraserorum* | Madagascar:West:Nosy Be | ECOMAR:Ich:NBE0607 | NBE0607 | JQ350186 |
| Mullidae | *Parupeneus heptacanthus* | French Polynesia:Society Islands:Moorea | MNHN:Ich:BIOCODES0880 | MBIO1461.4 | JQ431982 |
| Mullidae | *Parupeneus insularis* | French Polynesia:Society Islands:Moorea | MNHN:Ich:BIOCODES0977 | MBIO1647.4 | JQ431984 |
| Mullidae | *Parupeneus insularis* | French Polynesia:Society Islands:Moorea | SI:Ich:MBIO1648 | MBIO1648.4 | JQ431983 |
| Mullidae | *Parupeneus insularis* | French Polynesia:Society Islands:Moorea | MNHN:Ich:BIOCODES511 | MBIO750.4 | JQ431985 |
| Mullidae | *Parupeneus macronemus* | Madagascar:West:Nosy Be | ECOMAR:Ich:NBE0659 | NBE0659 | JQ350190 |
| Mullidae | *Parupeneus macronemus* | Reunion:West:St Leu | ECOMAR:Ich:REU0972 | REU0972 | JQ350191 |
| Mullidae | *Parupeneus macronemus* | Reunion:West:St Leu | ECOMAR:Ich:REU0973 | REU0973 | JQ350187 |
| Mullidae | *Parupeneus macronemus* | Reunion:West:St Leu | ECOMAR:Ich:REU2638 | REU2638 | JQ350189 |
| Mullidae | *Parupeneus macronemus* | Reunion:West:St Gilles | ECOMAR:Ich:REU305-1 | REU305_1 | JQ350188 |
| Mullidae | *Parupeneus multifasciatus* | French Polynesia:Society Islands:Moorea | MNHN:Ich:BIOCODES0063 | MBIO105.4 | JQ431986 |
| Mullidae | *Parupeneus multifasciatus* | French Polynesia:Society Islands:Moorea | MNHN:Ich:BIOCODES505 | MBIO744.4 | JQ431988 |
| Mullidae | *Parupeneus multifasciatus* | French Polynesia:Society Islands:Moorea | SI:Ich:MBIO745 | MBIO745.4 | JQ431987 |
| Mullidae | *Parupeneus pleurostigma* | French Polynesia:Society Islands:Moorea | MNHN:Ich:BIOCODES1033 | MBIO1841.4 | JQ431989 |
| Mullidae | *Parupeneus pleurostigma* | French Polynesia:Society Islands:Moorea | SI:Ich:MBIO1842 | MBIO1842.4 | JQ431990 |
| Mullidae | *Parupeneus pleurostigma* | Reunion:West:St Gilles | ECOMAR:Ich:REU0740 | REU0740 | JQ350193 |
| Mullidae | *Parupeneus pleurostigma* | Reunion:West:St Leu | ECOMAR:Ich:REU0916 | REU0916 | JQ350192 |
| Mullidae | *Upeneus tragula* | Madagascar:West:Nosy Be | ECOMAR:Ich:NBE0468 | NBE0468 | JQ350409 |
| Mullidae | *Upeneus tragula* | Madagascar:West:Nosy Be | ECOMAR:Ich:NBE1297 | NBE1297 | JQ350408 |
| Muraenidae | *Anarchias seychellensis* | Reunion:South:St Philippe | ECOMAR:Ich:REU1785 | REU1785 | JQ349701 |
| Muraenidae | *Anarchias seychellensis* | Reunion:South:St Philippe | ECOMAR:Ich:REU1858 | REU1858 | JQ349700 |
| Muraenidae | *Anarchias seychellensis* | Reunion:South:St Philippe | ECOMAR:Ich:REU1876 | REU1876 | JQ349697 |
| Muraenidae | *Anarchias seychellensis* | Reunion:South:St Philippe | ECOMAR:Ich:REU1877 | REU1877 | JQ349698 |
| Muraenidae | *Anarchias seychellensis* | Reunion:South:St Philippe | ECOMAR:Ich:REU1878 | REU1878 | JQ349699 |
| Muraenidae | *Echidna leucotaenia* | French Polynesia:Society Islands:Moorea | MNHN:Ich:BIOCODES0924 | MBIO1564.4 | JQ431695 |
| Muraenidae | *Echidna leucotaenia* | French Polynesia:Society Islands:Moorea | MNHN:Ich:BIOCODES0171 | MBIO246.4 | JQ431694 |
| Muraenidae | *Echidna leucotaenia* | French Polynesia:Society Islands:Moorea | SI:Ich:MBIO247 | MBIO247.4 | JQ431693 |
| Muraenidae | *Echidna nebulosa* | French Polynesia:Society Islands:Moorea | MNHN:Ich:BIOCODES758 | MBIO1169.4 | JQ431696 |
| Muraenidae | *Echidna polyzona* | French Polynesia:Society Islands:Moorea | MNHN:Ich:BIOCODES759 | MBIO1170.4 | JQ431697 |
| Muraenidae | *Enchelycore bayeri* | French Polynesia:Society Islands:Moorea | MNHN:Ich:BIOCODES0941 | MBIO1602.4 | JQ431703 |
| Muraenidae | *Enchelycore bayeri* | French Polynesia:Society Islands:Moorea | MNHN:Ich:BIOCODES297 | MBIO437.4 | JQ431705 |
| Muraenidae | *Enchelycore bayeri* | French Polynesia:Society Islands:Moorea | MNHN:Ich:BIOCODES298 | MBIO438.4 | JQ431704 |
| Muraenidae | *Enchelycore pardalis* | Reunion:South:St Philippe | ECOMAR:Ich:REU1873 | REU1873 | JQ349949 |
| Muraenidae | *Gymnothorax australicola* | French Polynesia:Society Islands:Moorea | MNHN:Ich:BIOCODES299 | MBIO440.4 | JQ431786 |
| Muraenidae | *Gymnothorax buroensis* | French Polynesia:Society Islands:Moorea | MNHN:Ich:BIOCODES0083 | MBIO131.4 | JQ431788 |
| Muraenidae | *Gymnothorax buroensis* | French Polynesia:Society Islands:Moorea | MNHN:Ich:BIOCODES0085 | MBIO132.4 | JQ431787 |
| Muraenidae | *Gymnothorax buroensis* | French Polynesia:Society Islands:Moorea | MNHN:Ich:BIOCODES289 | MBIO413.4 | JQ431789 |
| Muraenidae | *Gymnothorax buroensis* | French Polynesia:Society Islands:Moorea | MNHN:Ich:BIOCODES290 | MBIO414.4 | JQ431791 |
| Muraenidae | *Gymnothorax buroensis* | French Polynesia:Society Islands:Moorea | MNHN:Ich:BIOCODES597 | MBIO884.4 | JQ431790 |
| Muraenidae | *Gymnothorax buroensis* | Madagascar:West:Nosy Be | ECOMAR:Ich:NBE1261 | NBE1261 | JQ350017 |
| Muraenidae | *Gymnothorax buroensis* | Reunion:West:St Leu | ECOMAR:Ich:REU1935 | REU1935 | JQ350019 |
| Muraenidae | *Gymnothorax buroensis* | Reunion:West:St Leu | ECOMAR:Ich:REU1936 | REU1936 | JQ350018 |
| Muraenidae | *Gymnothorax buroensis* | Reunion:West:St Leu | ECOMAR:Ich:REU1938 | REU1938 | JQ350021 |
| Muraenidae | *Gymnothorax buroensis* | Reunion:West:St Leu | ECOMAR:Ich:REU1939 | REU1939 | JQ350020 |
| Muraenidae | *Gymnothorax buroensis* | Reunion:West:St Leu | ECOMAR:Ich:REU2642 | REU2642 | JQ350022 |
| Muraenidae | *Gymnothorax chilospilus* | French Polynesia:Society Islands:Moorea | MNHN:Ich:BIOCODES0127 | MBIO186.4 | JQ431794 |
| Muraenidae | *Gymnothorax chilospilus* | French Polynesia:Society Islands:Moorea | MNHN:Ich:BIOCODES287 | MBIO421.4 | JQ431795 |
| Muraenidae | *Gymnothorax chilospilus* | French Polynesia:Society Islands:Moorea | SI:Ich:MBIO422 | MBIO422.4 | JQ431792 |
| Muraenidae | *Gymnothorax chilospilus* | French Polynesia:Society Islands:Moorea | MNHN:Ich:BIOCODES300 | MBIO430.4 | JQ431793 |
| Muraenidae | *Gymnothorax chilospilus* | French Polynesia:Society Islands:Moorea | MNHN:Ich:BIOCODES595 | MBIO881.4 | JQ431797 |
| Muraenidae | *Gymnothorax chilospilus* | French Polynesia:Society Islands:Moorea | MNHN:Ich:BIOCODES596 | MBIO882.4 | JQ431796 |
| Muraenidae | *Gymnothorax chilospilus* | Reunion:South:St Philippe | ECOMAR:Ich:REU1784 | REU1784 | JQ350023 |
| Muraenidae | *Gymnothorax elegans* | French Polynesia:Society Islands:Moorea | MNHN:Ich:BIOCODES685 | MBIO1006.4 | JQ431798 |
| Muraenidae | *Gymnothorax eurostus* | French Polynesia:Society Islands:Moorea | MNHN:Ich:BIOCODES0134 | MBIO196.4 | JQ431800 |
| Muraenidae | *Gymnothorax eurostus* | French Polynesia:Society Islands:Moorea | SI:Ich:MBIO197 | MBIO197.4 | JQ431799 |
| Muraenidae | *Gymnothorax favagineus* | Madagascar:West:Nosy Be | ECOMAR:Ich:NBE0310 | NBE0310 | JQ350025 |
| Muraenidae | *Gymnothorax favagineus* | Madagascar:West:Nosy Be | ECOMAR:Ich:NBE0603 | NBE0603 | JQ350024 |
| Muraenidae | *Gymnothorax fimbriatus* | French Polynesia:Society Islands:Moorea | MNHN:Ich:BIOCODES0086 | MBIO135.4 | JQ431801 |
| Muraenidae | *Gymnothorax flavimarginatus* | Madagascar:West:Nosy Be | ECOMAR:Ich:NBE0449 | NBE0449 | JQ350026 |
| Muraenidae | *Gymnothorax flavimarginatus* | Reunion:South:St Philippe | ECOMAR:Ich:REU1879 | REU1879 | JQ350028 |
| Muraenidae | *Gymnothorax flavimarginatus* | Reunion:West:La Saline | ECOMAR:Ich:REU2583 | REU2583 | JQ350027 |
| Muraenidae | *Gymnothorax fuscomaculatus* | Reunion:West:St Leu | ECOMAR:Ich:REU1931 | REU1931 | JQ350029 |
| Muraenidae | *Gymnothorax fuscomaculatus* | Reunion:West:St Leu | ECOMAR:Ich:REU1932 | REU1932 | JQ350032 |
| Muraenidae | *Gymnothorax fuscomaculatus* | Reunion:West:St Leu | ECOMAR:Ich:REU1933 | REU1933 | JQ350031 |
| Muraenidae | *Gymnothorax fuscomaculatus* | Reunion:West:St Leu | ECOMAR:Ich:REU1934 | REU1934 | JQ350030 |
| Muraenidae | *Gymnothorax gracilicauda* | French Polynesia:Society Islands:Moorea | MNHN:Ich:BIOCODES686 | MBIO1007.4 | JQ431802 |
| Muraenidae | *Gymnothorax gracilicauda* | French Polynesia:Society Islands:Moorea | MNHN:Ich:BIOCODES0170 | MBIO244.4 | JQ431803 |
| Muraenidae | *Gymnothorax griseus* | Madagascar:West:Nosy Be | ECOMAR:Ich:NBE0311 | NBE0311 | JQ350034 |
| Muraenidae | *Gymnothorax griseus* | Madagascar:West:Nosy Be | ECOMAR:Ich:NBE1026 | NBE1026 | JQ350035 |
| Muraenidae | *Gymnothorax griseus* | Reunion:West:St Gilles | ECOMAR:Ich:REU1674 | REU1674 | JQ350033 |
| Muraenidae | *Gymnothorax javanicus* | French Polynesia:Society Islands:Moorea | MNHN:Ich:BIOCODES678 | MBIO1005.4 | JQ431806 |
| Muraenidae | *Gymnothorax javanicus* | French Polynesia:Society Islands:Moorea | MNHN:Ich:BIOCODES0919 | MBIO1544.4 | JQ431804 |
| Muraenidae | *Gymnothorax javanicus* | French Polynesia:Society Islands:Moorea | SI:Ich:MBIO1603 | MBIO1603.4 | JQ431805 |
| Muraenidae | *Gymnothorax javanicus* | Madagascar:West:Nosy Be | ECOMAR:Ich:NBE0304 | NBE0304 | JQ350036 |
| Muraenidae | *Gymnothorax javanicus* | Madagascar:West:Nosy Be | ECOMAR:Ich:NBE0305 | NBE0305 | JQ350039 |
| Muraenidae | *Gymnothorax javanicus* | Madagascar:West:Nosy Be | ECOMAR:Ich:NBE0306 | NBE0306 | JQ350037 |
| Muraenidae | *Gymnothorax javanicus* | Madagascar:West:Nosy Be | ECOMAR:Ich:NBE1027 | NBE1027 | JQ350038 |
| Muraenidae | *Gymnothorax margaritophorus* | French Polynesia:Society Islands:Moorea | MNHN:Ich:BIOCODES690 | MBIO1010.4 | JQ431812 |
| Muraenidae | *Gymnothorax margaritophorus* | French Polynesia:Society Islands:Moorea | SI:Ich:MBIO1011 | MBIO1011.4 | JQ431808 |
| Muraenidae | *Gymnothorax margaritophorus* | French Polynesia:Society Islands:Moorea | MNHN:Ich:BIOCODES284 | MBIO417.4 | JQ431810 |
| Muraenidae | *Gymnothorax margaritophorus* | French Polynesia:Society Islands:Moorea | MNHN:Ich:BIOCODES285 | MBIO418.4 | JQ431811 |
| Muraenidae | *Gymnothorax margaritophorus* | French Polynesia:Society Islands:Moorea | MNHN:Ich:BIOCODES292 | MBIO424.4 | JQ431814 |
| Muraenidae | *Gymnothorax margaritophorus* | French Polynesia:Society Islands:Moorea | MNHN:Ich:BIOCODES296 | MBIO425.4 | JQ431813 |
| Muraenidae | *Gymnothorax margaritophorus* | French Polynesia:Society Islands:Moorea | MNHN:Ich:BIOCODES301 | MBIO429.4 | JQ431809 |
| Muraenidae | *Gymnothorax margaritophorus* | French Polynesia:Society Islands:Moorea | MNHN:Ich:BIOCODES593 | MBIO878.4 | JQ431807 |
| Muraenidae | *Gymnothorax margaritophorus* | French Polynesia:Society Islands:Moorea | MNHN:Ich:BIOCODES594 | MBIO879.4 | JQ431815 |
| Muraenidae | *Gymnothorax margaritophorus* | Reunion:West:St Leu | ECOMAR:Ich:REU1940 | REU1940 | JQ350040 |
| Muraenidae | *Gymnothorax melatremus* | French Polynesia:Society Islands:Moorea | MNHN:Ich:BIOCODES688 | MBIO1015.4 | JQ431817 |
| Muraenidae | *Gymnothorax melatremus* | French Polynesia:Society Islands:Moorea | MNHN:Ich:BIOCODES0081 | MBIO128.4 | JQ431816 |
| Muraenidae | *Gymnothorax melatremus* | French Polynesia:Society Islands:Moorea | SI:Ich:MBIO693 | MBIO693.4 | JQ431818 |
| Muraenidae | *Gymnothorax pictus* | Madagascar:West:Nosy Be | ECOMAR:Ich:NBE0125 | NBE0125 | JQ350043 |
| Muraenidae | *Gymnothorax pictus* | Madagascar:West:Nosy Be | ECOMAR:Ich:NBE0604 | NBE0604 | JQ350042 |
| Muraenidae | *Gymnothorax pictus* | Madagascar:West:Nosy Be | ECOMAR:Ich:NBE0605 | NBE0605 | JQ350041 |
| Muraenidae | *Gymnothorax pictus* | Madagascar:West:Nosy Be | ECOMAR:Ich:NBE0606 | NBE0606 | JQ350044 |
| Muraenidae | *Gymnothorax reevesii* | French Polynesia:Society Islands:Moorea | MNHN:Ich:BIOCODES755 | MBIO1163.4 | JQ431819 |
| Muraenidae | *Gymnothorax reevesii* | French Polynesia:Society Islands:Moorea | MNHN:Ich:BIOCODES756 | MBIO1164.4 | JQ431820 |
| Muraenidae | *Gymnothorax richardsonii* | French Polynesia:Society Islands:Moorea | MNHN:Ich:BIOCODES569 | MBIO840.4 | JQ431821 |
| Muraenidae | *Gymnothorax rueppellii* | French Polynesia:Society Islands:Moorea | MNHN:Ich:BIOCODES0168 | MBIO240.4 | JQ431822 |
| Muraenidae | *Gymnothorax* sp. 1 | French Polynesia:Society Islands:Moorea | MNHN:Ich:BIOCODES0875 | MBIO1462.4 | JQ431823 |
| Muraenidae | *Gymnothorax* sp. 2 | French Polynesia:Society Islands:Moorea | MNHN:Ich:BIOCODES590 | MBIO870.4 | JQ431824 |
| Muraenidae | *Gymnothorax* sp. 3 | Madagascar:West:Nosy Be | ECOMAR:Ich:NBE0539 | NBE0539 | JQ350045 |
| Muraenidae | *Gymnothorax thyrsoideus* | French Polynesia:Society Islands:Moorea | MNHN:Ich:BIOCODES0082 | MBIO129.4 | JQ431827 |
| Muraenidae | *Gymnothorax thyrsoideus* | French Polynesia:Society Islands:Moorea | MNHN:Ich:BIOCODES0867 | MBIO1435.4 | JQ431825 |
| Muraenidae | *Gymnothorax thyrsoideus* | French Polynesia:Society Islands:Moorea | SI:Ich:MBIO1642 | MBIO1642.4 | JQ431826 |
| Muraenidae | *Gymnothorax undulatus* | French Polynesia:Society Islands:Moorea | MNHN:Ich:BIOCODES752 | MBIO1160.4 | JQ431829 |
| Muraenidae | *Gymnothorax undulatus* | French Polynesia:Society Islands:Moorea | MNHN:Ich:BIOCODES751 | MBIO1161.4 | JQ431828 |
| Muraenidae | *Gymnothorax undulatus* | Madagascar:West:Nosy Be | ECOMAR:Ich:NBE0302 | NBE0302 | JQ350047 |
| Muraenidae | *Gymnothorax undulatus* | Madagascar:West:Nosy Be | ECOMAR:Ich:NBE0307 | NBE0307 | JQ350046 |
| Muraenidae | *Gymnothorax undulatus* | Madagascar:West:Nosy Be | ECOMAR:Ich:NBE0308 | NBE0308 | JQ350049 |
| Muraenidae | *Gymnothorax undulatus* | Reunion:West:St Gilles | ECOMAR:Ich:REU1673 | REU1673 | JQ350048 |
| Muraenidae | *Gymnothorax zonipectis* | French Polynesia:Society Islands:Moorea | MNHN:Ich:BIOCODES689 | MBIO1012.4 | JQ431836 |
| Muraenidae | *Gymnothorax zonipectis* | French Polynesia:Society Islands:Moorea | MNHN:Ich:BIOCODES681 | MBIO1013.4 | JQ431833 |
| Muraenidae | *Gymnothorax zonipectis* | French Polynesia:Society Islands:Moorea | MNHN:Ich:BIOCODES0109 | MBIO164.4 | JQ431832 |
| Muraenidae | *Gymnothorax zonipectis* | French Polynesia:Society Islands:Moorea | MNHN:Ich:BIOCODES0473 | MBIO694.4 | JQ431835 |
| Muraenidae | *Gymnothorax zonipectis* | French Polynesia:Society Islands:Moorea | MNHN:Ich:BIOCODES0477 | MBIO695.4 | JQ431830 |
| Muraenidae | *Gymnothorax zonipectis* | French Polynesia:Society Islands:Moorea | MNHN:Ich:BIOCODES591 | MBIO875.4 | JQ431834 |
| Muraenidae | *Gymnothorax zonipectis* | French Polynesia:Society Islands:Moorea | MNHN:Ich:BIOCODES592 | MBIO876.4 | JQ431831 |
| Muraenidae | *Gymnothorax zonipectis* | Madagascar:West:Nosy Be | ECOMAR:Ich:NBE1260 | NBE1260 | JQ350050 |
| Muraenidae | *Uropterygius alboguttatus* | French Polynesia:Society Islands:Moorea | MNHN:Ich:BIOCODES684 | MBIO1008.4 | JQ432204 |
| Muraenidae | *Uropterygius fuscoguttatus* | French Polynesia:Society Islands:Moorea | MNHN:Ich:BIOCODES0093 | MBIO134.4 | JQ432207 |
| Muraenidae | *Uropterygius fuscoguttatus* | French Polynesia:Society Islands:Moorea | SI:Ich:MBIO1397 | MBIO1397.4 | JQ432205 |
| Muraenidae | *Uropterygius fuscoguttatus* | French Polynesia:Society Islands:Moorea | SI:Ich:MBIO159 | MBIO159.4 | JQ432206 |
| Muraenidae | *Uropterygius fuscoguttatus* | French Polynesia:Society Islands:Moorea | MNHN:Ich:BIOCODES288 | MBIO423.4 | JQ432208 |
| Muraenidae | *Uropterygius fuscoguttatus* | Madagascar:West:Nosy Be | ECOMAR:Ich:NBE0438 | NBE0438 | JQ350411 |
| Muraenidae | *Uropterygius fuscoguttatus* | Reunion:West:St Leu | ECOMAR:Ich:REU1676 | REU1676 | JQ350412 |
| Muraenidae | *Uropterygius fuscoguttatus* | Reunion:West:St Leu | ECOMAR:Ich:REU1927 | REU1927 | JQ350410 |
| Muraenidae | *Uropterygius xanthopterus* | French Polynesia:Society Islands:Moorea | MNHN:Ich:BIOCODES695 | MBIO1027.4 | JQ432212 |
| Muraenidae | *Uropterygius xanthopterus* | French Polynesia:Society Islands:Moorea | MNHN:Ich:BIOCODES0098 | MBIO148.4 | JQ432210 |
| Muraenidae | *Uropterygius xanthopterus* | French Polynesia:Society Islands:Moorea | MNHN:Ich:BIOCODES0099 | MBIO149.4 | JQ432211 |
| Muraenidae | *Uropterygius xanthopterus* | French Polynesia:Society Islands:Moorea | MNHN:Ich:BIOCODES0928 | MBIO1569.4 | JQ432213 |
| Muraenidae | *Uropterygius xanthopterus* | French Polynesia:Society Islands:Moorea | MNHN:Ich:BIOCODES0926 | MBIO1570.4 | JQ432209 |
| Nemipteridae | *Nemipterus bipunctatus* | Madagascar:West:Nosy Be | ECOMAR:Ich:NBE1086 | NBE1086 | JQ350137 |
| Nemipteridae | *Scolopsis bimaculatus* | Madagascar:West:Nosy Be | ECOMAR:Ich:NBE0542 | NBE0542 | JQ350339 |
| Nemipteridae | *Scolopsis bimaculatus* | Madagascar:West:Nosy Be | ECOMAR:Ich:NBE0543 | NBE0543 | JQ350336 |
| Nemipteridae | *Scolopsis bimaculatus* | Madagascar:West:Nosy Be | ECOMAR:Ich:NBE0544 | NBE0544 | JQ350337 |
| Nemipteridae | *Scolopsis bimaculatus* | Madagascar:West:Nosy Be | ECOMAR:Ich:NBE0545 | NBE0545 | JQ350340 |
| Nemipteridae | *Scolopsis bimaculatus* | Madagascar:West:Nosy Be | ECOMAR:Ich:NBE1107 | NBE1107 | JQ350338 |
| Nemipteridae | *Scolopsis ghanam* | Madagascar:West:Nosy Be | ECOMAR:Ich:NBE0019 | NBE0019 | JQ350343 |
| Nemipteridae | *Scolopsis ghanam* | Madagascar:West:Nosy Be | ECOMAR:Ich:NBE0223 | NBE0223 | JQ350344 |
| Nemipteridae | *Scolopsis ghanam* | Madagascar:West:Nosy Be | ECOMAR:Ich:NBE0224 | NBE0224 | JQ350341 |
| Nemipteridae | *Scolopsis ghanam* | Madagascar:West:Nosy Be | ECOMAR:Ich:NBE0225 | NBE0225 | JQ350342 |
| Nemipteridae | *Scolopsis vosmeri* | Madagascar:West:Nosy Be | ECOMAR:Ich:NBE0001 | NBE0001 | JQ350347 |
| Nemipteridae | *Scolopsis vosmeri* | Madagascar:West:Nosy Be | ECOMAR:Ich:NBE0450 | NBE0450 | JQ350349 |
| Nemipteridae | *Scolopsis vosmeri* | Madagascar:West:Nosy Be | ECOMAR:Ich:NBE0451 | NBE0451 | JQ350345 |
| Nemipteridae | *Scolopsis vosmeri* | Madagascar:West:Nosy Be | ECOMAR:Ich:NBE0452 | NBE0452 | JQ350346 |
| Nemipteridae | *Scolopsis vosmeri* | Madagascar:West:Nosy Be | ECOMAR:Ich:NBE0620 | NBE0620 | JQ350348 |
| Ophichthidae | *Apterichtus australis* | French Polynesia:Society Islands:Moorea | MNHN:Ich:BIOCODES0103 | MBIO155.4 | JQ431459 |
| Ophichthidae | *Brachysomophis henshawi* | French Polynesia:Society Islands:Moorea | MNHN:Ich:BIOCODES0083 | MBIO130.4 | JQ431496 |
| Ophichthidae | *Callechelys catostoma* | French Polynesia:Society Islands:Moorea | MNHN:Ich:BIOCODES0902 | MBIO1477.4 | JQ431505 |
| Ophichthidae | *Callechelys marmorata* | French Polynesia:Society Islands:Moorea | MNHN:Ich:BIOCODES797 | MBIO1243.4 | JQ431507 |
| Ophichthidae | *Callechelys marmorata* | French Polynesia:Society Islands:Moorea | MNHN:Ich:BIOCODES0892 | MBIO1482.4 | JQ431506 |
| Ophichthidae | *Callechelys marmorata* | French Polynesia:Society Islands:Moorea | MNHN:Ich:BIOCODES0893 | MBIO1483.4 | JQ431509 |
| Ophichthidae | *Callechelys marmorata* | French Polynesia:Society Islands:Moorea | SI:Ich:MBIO1504 | MBIO1504.4 | JQ431508 |
| Ophichthidae | *Leiuranus semicinctus* | French Polynesia:Society Islands:Moorea | MNHN:Ich:BIOCODES0356 | MBIO510.4 | JQ431881 |
| Ophichthidae | *Leiuranus semicinctus* | French Polynesia:Society Islands:Moorea | SI:Ich:MBIO511 | MBIO511.4 | JQ431880 |
| Ophichthidae | *Muraenichthys* sp. 2 | French Polynesia:Society Islands:Moorea | MNHN:Ich:BIOCODES0169 | MBIO242.4 | JQ431927 |
| Ophichthidae | *Myrichthys colubrinus* | French Polynesia:Society Islands:Moorea | MNHN:Ich:BIOCODES0895 | MBIO1489.4 | JQ431928 |
| Ophichthidae | *Ophichthus polyophthalmus* | French Polynesia:Society Islands:Moorea | MNHN:Ich:BIOCODES682 | MBIO1009.4 | JQ431944 |
| Ophichthidae | *Ophichthus sp.* | French Polynesia:Society Islands:Moorea | SI:Ich:BIOCODES761 | MBIO1177.4 | JQ431945 |
| Ophichthidae | *Scolecenchelys gymnota* | French Polynesia:Society Islands:Moorea | MNHN:Ich:BIOCODES794 | MBIO1238.4 | JQ432117 |
| Ophichthidae | *Scolecenchelys gymnota* | French Polynesia:Society Islands:Moorea | SI:Ich:MBIO1239 | MBIO1239.4 | JQ432116 |
| Ophichthidae | *Scolecenchelys gymnota* | French Polynesia:Society Islands:Moorea | MNHN:Ich:BIOCODES0903 | MBIO1499.4 | JQ432118 |
| Ophichthidae | *Scolecenchelys gymnota* | French Polynesia:Society Islands:Moorea | MNHN:Ich:BIOCODES0905 | MBIO1500.4 | JQ432115 |
| Ophidiidae | *Brotula multibarbata* | French Polynesia:Society Islands:Moorea | MNHN:Ich:BIOCODES729 | MBIO1132.4 | JQ431501 |
| Ophidiidae | *Brotula multibarbata* | French Polynesia:Society Islands:Moorea | MNHN:Ich:BIOCODES730 | MBIO1133.4 | JQ431502 |
| Ophidiidae | *Brotula multibarbata* | French Polynesia:Society Islands:Moorea | MNHN:Ich:BIOCODES0467 | MBIO680.4 | JQ431499 |
| Ophidiidae | *Brotula multibarbata* | French Polynesia:Society Islands:Moorea | MNHN:Ich:BIOCODES0461 | MBIO681.4 | JQ431500 |
| Ophidiidae | *Brotula multibarbata* | French Polynesia:Society Islands:Moorea | SI:Ich:MBIO973 | MBIO973.4 | JQ431498 |
| Ostraciidae | *Ostracion cubicus* | French Polynesia:Society Islands:Moorea | MNHN:Ich:BIOCODES1006 | MBIO1790.4 | JQ431949 |
| Ostraciidae | *Ostracion cubicus* | French Polynesia:Society Islands:Moorea | SI:Ich:MBIO1791 | MBIO1791.4 | JQ431948 |
| Ostraciidae | *Ostracion cubicus* | Madagascar:West:Nosy Be | ECOMAR:Ich:NBE0183 | NBE0183 | JQ350146 |
| Ostraciidae | *Ostracion cubicus* | Madagascar:West:Nosy Be | ECOMAR:Ich:NBE0675 | NBE0675 | JQ350148 |
| Ostraciidae | *Ostracion cubicus* | Reunion:West:St Gilles | ECOMAR:Ich:REU014-1 | REU014_1 | JQ350149 |
| Ostraciidae | *Ostracion cubicus* | Reunion:West:St Gilles | ECOMAR:Ich:REU1671 | REU1671 | JQ350150 |
| Ostraciidae | *Ostracion cubicus* | Reunion:West:St Gilles | ECOMAR:Ich:REU337-1 | REU337_1 | JQ350147 |
| Ostraciidae | *Ostracion meleagris* | French Polynesia:Society Islands:Moorea | MNHN:Ich:BIOCODES0160 | MBIO227.4 | JQ431950 |
| Ostraciidae | *Ostracion meleagris* | French Polynesia:Society Islands:Moorea | MNHN:Ich:BIOCODES0182 | MBIO259.4 | JQ431952 |
| Ostraciidae | *Ostracion meleagris* | French Polynesia:Society Islands:Moorea | MNHN:Ich:BIOCODES0351 | MBIO504.4 | JQ431951 |
| Ostraciidae | *Ostracion meleagris* | Reunion:West:St Gilles | ECOMAR:Ich:REU0115 | REU0115 | JQ350151 |
| Ostraciidae | *Ostracion meleagris* | Reunion:West:St Gilles | ECOMAR:Ich:REU0777 | REU0777 | JQ350153 |
| Ostraciidae | *Ostracion meleagris* | Reunion:West:St Leu | ECOMAR:Ich:REU2662 | REU2662 | JQ350152 |
| Pempheridae | *Pempheris adusta* | Reunion:West:St Gilles | ECOMAR:Ich:REU0731 | REU0731 | JQ350194 |
| Pempheridae | *Pempheris adusta* | Reunion:West:St Gilles | ECOMAR:Ich:REU0732 | REU0732 | JQ350196 |
| Pempheridae | *Pempheris adusta* | Reunion:West:St Leu | ECOMAR:Ich:REU0936 | REU0936 | JQ350195 |
| Pempheridae | *Pempheris adusta* | Reunion:West:St Leu | ECOMAR:Ich:REU0937 | REU0937 | JQ350197 |
| Pempheridae | *Pempheris adusta* | Reunion:West:St Leu | ECOMAR:Ich:REU0938 | REU0938 | JQ350198 |
| Pempheridae | *Pempheris mangula* | Madagascar:West:Nosy Be | ECOMAR:Ich:NBE0182 | NBE0182 | JQ350199 |
| Pempheridae | *Pempheris oualensis* | Reunion:West:St Leu | ECOMAR:Ich:REU0957 | REU0957 | JQ350201 |
| Pempheridae | *Pempheris oualensis* | Reunion:West:St Leu | ECOMAR:Ich:REU0958 | REU0958 | JQ350200 |
| Pempheridae | *Pempheris vanicolensis* | Madagascar:West:Nosy Be | ECOMAR:Ich:NBE0213 | NBE0213 | JQ350203 |
| Pempheridae | *Pempheris vanicolensis* | Madagascar:West:Nosy Be | ECOMAR:Ich:NBE0214 | NBE0214 | JQ350202 |
| Pempheridae | *Pempheris vanicolensis* | Madagascar:West:Nosy Be | ECOMAR:Ich:NBE0215 | NBE0215 | JQ350204 |
| Pinguipedidae | *Parapercis hexophtalma* | Madagascar:West:Nosy Be | ECOMAR:Ich:NBE0044 | NBE0044 | JQ350175 |
| Pinguipedidae | *Parapercis hexophtalma* | Madagascar:West:Nosy Be | ECOMAR:Ich:NBE0250 | NBE0250 | JQ350176 |
| Pinguipedidae | *Parapercis hexophtalma* | Madagascar:West:Nosy Be | ECOMAR:Ich:NBE1295 | NBE1295 | JQ350173 |
| Pinguipedidae | *Parapercis hexophtalma* | Madagascar:West:Nosy Be | ECOMAR:Ich:NBE1296 | NBE1296 | JQ350174 |
| Pinguipedidae | *Parapercis millepunctata* | French Polynesia:Society Islands:Moorea | MNHN:Ich:BIOCODES0383 | MBIO546.4 | JQ431972 |
| Platycephalidae | *Cociella crocodila* | Madagascar:West:Nosy Be | ECOMAR:Ich:NBE0292 | NBE0292 | JQ349911 |
| Platycephalidae | *Onigocia bimaculata* | French Polynesia:Society Islands:Moorea | MNHN:Ich:BIOCODES0857 | MBIO1383.4 | JQ431943 |
| Platycephalidae | *Onigocia bimaculata* | French Polynesia:Society Islands:Moorea | MNHN:Ich:BIOCODES0853 | MBIO1384.4 | JQ431942 |
| Platycephalidae | *Onigocia bimaculata* | French Polynesia:Society Islands:Moorea | MNHN:Ich:BIOCODES0126 | MBIO185.4 | JQ431939 |
| Platycephalidae | *Onigocia bimaculata* | French Polynesia:Society Islands:Moorea | MNHN:Ich:BIOCODES336 | MBIO479.4 | JQ431940 |
| Platycephalidae | *Onigocia bimaculata* | French Polynesia:Society Islands:Moorea | MNHN:Ich:BIOCODES334 | MBIO480.4 | JQ431941 |
| Platycephalidae | *Papilloculiceps longiceps* | Madagascar:West:Nosy Be | ECOMAR:Ich:NBE0600 | NBE0600 | JQ350159 |
| Plesiopidae | *Calloplesiops altivelis* | Madagascar:West:Nosy Be | ECOMAR:Ich:NBE0078 | NBE0078 | JQ349814 |
| Plesiopidae | *Plesiops coeruleolineatus* | Reunion:West:La Saline | ECOMAR:Ich:REU2579 | REU2579 | JQ350232 |
| Plotosidae | *Plotosus lineatus* | Reunion:West:La Saline | ECOMAR:Ich:REU214-1 | REU214_1 | JQ350233 |
| Poeciliidae | *Poecilia reticulata* | French Polynesia:Society Islands:Moorea | MNHN:Ich:BIOCODES1043 | MBIO1855.4 | JQ432022 |
| Polynemidae | *Polydactylus plebeius* | French Polynesia:Society Islands:Moorea | MNHN:Ich:BIOCODES562 | MBIO829.4 | JQ432025 |
| Polynemidae | *Polydactylus plebeius* | French Polynesia:Society Islands:Moorea | MNHN:Ich:BIOCODES563 | MBIO830.4 | JQ432024 |
| Pomacanthidae | *Apolemichthys trimaculatus* | Madagascar:West:Nosy Be | ECOMAR:Ich:NBE1101 | NBE1101 | JQ349773 |
| Pomacanthidae | *Apolemichthys trimaculatus* | Madagascar:West:Nosy Be | ECOMAR:Ich:NBE1102 | NBE1102 | JQ349772 |
| Pomacanthidae | *Apolemichthys trimaculatus* | Madagascar:West:Nosy Be | ECOMAR:Ich:NBE1103 | NBE1103 | JQ349771 |
| Pomacanthidae | *Apolemichthys trimaculatus* | Reunion:West:St Leu | ECOMAR:Ich:REU0902 | REU0902 | JQ349770 |
| Pomacanthidae | *Centropyge acanthops* | Reunion:West:La Saline | ECOMAR:Ich:REU0301 | REU0301 | JQ349844 |
| Pomacanthidae | *Centropyge acanthops* | Reunion:West:La Saline | ECOMAR:Ich:REU0302 | REU0302 | JQ349843 |
| Pomacanthidae | *Centropyge bispinosa* | French Polynesia:Society Islands:Moorea | MNHN:Ich:BIOCODES0129 | MBIO188.4 | JQ431554 |
| Pomacanthidae | *Centropyge bispinosa* | French Polynesia:Society Islands:Moorea | MNHN:Ich:BIOCODES0130 | MBIO189.4 | JQ431555 |
| Pomacanthidae | *Centropyge bispinosa* | Reunion:West:St Gilles | ECOMAR:Ich:REU0739 | REU0739 | JQ349845 |
| Pomacanthidae | *Centropyge bispinosa* | Reunion:West:St Leu | ECOMAR:Ich:REU0901 | REU0901 | JQ349847 |
| Pomacanthidae | *Centropyge bispinosa* | Reunion:West:St Leu | ECOMAR:Ich:REU0989 | REU0989 | JQ349846 |
| Pomacanthidae | *Centropyge flavissima* | French Polynesia:Society Islands:Moorea | MNHN:Ich:BIOCODES0068 | MBIO111.4 | JQ431558 |
| Pomacanthidae | *Centropyge flavissima* | French Polynesia:Society Islands:Moorea | MNHN:Ich:BIOCODES0069 | MBIO112.4 | JQ431557 |
| Pomacanthidae | *Centropyge flavissima* | French Polynesia:Society Islands:Moorea | MNHN:Ich:BIOCODES577 | MBIO852.4 | JQ431559 |
| Pomacanthidae | *Centropyge flavissima* | French Polynesia:Society Islands:Moorea | MNHN:Ich:BIOCODES578 | MBIO853.4 | JQ431556 |
| Pomacanthidae | *Centropyge heraldi* | French Polynesia:Society Islands:Moorea | SI:Ich:MBIO1259 | MBIO1259.4 | JQ431560 |
| Pomacanthidae | *Centropyge heraldi* | French Polynesia:Society Islands:Moorea | MNHN:Ich:BIOCODES659 | MBIO974.4 | JQ431561 |
| Pomacanthidae | *Centropyge loricula* | French Polynesia:Society Islands:Moorea | MNHN:Ich:BIOCODES0026 | MBIO53.4 | JQ431563 |
| Pomacanthidae | *Centropyge loricula* | French Polynesia:Society Islands:Moorea | MNHN:Ich:BIOCODES0027 | MBIO54.4 | JQ431562 |
| Pomacanthidae | *Centropyge multispinis* | Madagascar:West:Nosy Be | ECOMAR:Ich:NBE0369 | NBE0369 | JQ349852 |
| Pomacanthidae | *Centropyge multispinis* | Madagascar:West:Nosy Be | ECOMAR:Ich:NBE0370 | NBE0370 | JQ349848 |
| Pomacanthidae | *Centropyge multispinis* | Madagascar:West:Nosy Be | ECOMAR:Ich:NBE0390 | NBE0390 | JQ349850 |
| Pomacanthidae | *Centropyge multispinis* | Madagascar:West:Nosy Be | ECOMAR:Ich:NBE0391 | NBE0391 | JQ349849 |
| Pomacanthidae | *Centropyge multispinis* | Madagascar:West:Nosy Be | ECOMAR:Ich:NBE1149 | NBE1149 | JQ349851 |
| Pomacanthidae | *Centropyge multispinis* | Reunion:West:St Leu | ECOMAR:Ich:REU0900 | REU0900 | JQ349853 |
| Pomacanthidae | *Centropyge multispinis* | Reunion:West:St Leu | ECOMAR:Ich:REU0990 | REU0990 | JQ349855 |
| Pomacanthidae | *Centropyge multispinis* | Reunion:West:St Leu | ECOMAR:Ich:REU0991 | REU0991 | JQ349854 |
| Pomacanthidae | *Genicanthus bellus* | French Polynesia:Society Islands:Moorea | MNHN:Ich:BIOCODES0956 | MBIO1615.4 | JQ431750 |
| Pomacanthidae | *Genicanthus bellus* | French Polynesia:Society Islands:Moorea | SI:Ich:MBIO1616 | MBIO1616.4 | JQ431751 |
| Pomacanthidae | *Genicanthus watanabei* | French Polynesia:Society Islands:Moorea | MNHN:Ich:BIOCODES0889 | MBIO1473.4 | JQ431752 |
| Pomacanthidae | *Genicanthus watanabei* | French Polynesia:Society Islands:Moorea | SI:Ich:MBIO1474 | MBIO1474.4 | JQ431753 |
| Pomacanthidae | *Pomacanthus imperator* | French Polynesia:Society Islands:Moorea | MNHN:Ich:BIOCODES696 | MBIO1032.4 | JQ432027 |
| Pomacanthidae | *Pomacanthus imperator* | French Polynesia:Society Islands:Moorea | MNHN:Ich:BIOCODES815 | MBIO1273.4 | JQ432029 |
| Pomacanthidae | *Pomacanthus imperator* | French Polynesia:Society Islands:Moorea | SI:Ich:MBIO1274 | MBIO1274.4 | JQ432028 |
| Pomacanthidae | *Pomacanthus imperator* | Madagascar:West:Nosy Be | ECOMAR:Ich:NBE0338 | NBE0338 | JQ350235 |
| Pomacanthidae | *Pomacanthus imperator* | Madagascar:West:Nosy Be | ECOMAR:Ich:NBE1146 | NBE1146 | JQ350234 |
| Pomacanthidae | *Pomacanthus semicirculatus* | Madagascar:West:Nosy Be | ECOMAR:Ich:NBE0344 | NBE0344 | JQ350237 |
| Pomacanthidae | *Pomacanthus semicirculatus* | Madagascar:West:Nosy Be | ECOMAR:Ich:NBE0345 | NBE0345 | JQ350236 |
| Pomacanthidae | *Pomacanthus semicirculatus* | Madagascar:West:Nosy Be | ECOMAR:Ich:NBE0676 | NBE0676 | JQ350238 |
| Pomacanthidae | *Pygoplites diacanthus* | French Polynesia:Society Islands:Moorea | SI:Ich:MBIO1250 | MBIO1250.4 | JQ432082 |
| Pomacanthidae | *Pygoplites diacanthus* | French Polynesia:Society Islands:Moorea | MNHN:Ich:BIOCODES0451 | MBIO659.4 | JQ432083 |
| Pomacanthidae | *Pygoplites diacanthus* | French Polynesia:Society Islands:Moorea | SI:Ich:MBIO660 | MBIO660.4 | JQ432081 |
| Pomacanthidae | *Pygoplites diacanthus* | Madagascar:West:Nosy Be | ECOMAR:Ich:NBE0346 | NBE0346 | JQ350298 |
| Pomacanthidae | *Pygoplites diacanthus* | Madagascar:West:Nosy Be | ECOMAR:Ich:NBE0568 | NBE0568 | JQ350299 |
| Pomacentridae | *Abudefduf margariteus* | Reunion:West:St Leu | ECOMAR:Ich:REU0925 | REU0925 | JF434705 |
| Pomacentridae | *Abudefduf margariteus* | Reunion:West:St Leu | ECOMAR:Ich:REU0981 | REU0981 | JF434704 |
| Pomacentridae | *Abudefduf septemfasciatus* | French Polynesia:Society Islands:Moorea | MNHN:Ich:BIOCODES0309 | MBIO450.4 | JQ431394 |
| Pomacentridae | *Abudefduf septemfasciatus* | French Polynesia:Society Islands:Moorea | SI:Ich:MBIO451 | MBIO451.4 | JQ431395 |
| Pomacentridae | *Abudefduf septemfasciatus* | French Polynesia:Society Islands:Moorea | MNHN:Ich:BIOCODES555 | MBIO821.4 | JQ431396 |
| Pomacentridae | *Abudefduf septemfasciatus* | Madagascar:West:Nosy Be | ECOMAR:Ich:NBE0017 | NBE0017 | JF434706 |
| Pomacentridae | *Abudefduf sexfasciatus* | French Polynesia:Society Islands:Moorea | MNHN:Ich:BIOCODES478 | MBIO706.4 | JQ431398 |
| Pomacentridae | *Abudefduf sexfasciatus* | French Polynesia:Society Islands:Moorea | SI:Ich:MBIO707 | MBIO707.4 | JQ431397 |
| Pomacentridae | *Abudefduf sexfasciatus* | Madagascar:West:Nosy Be | ECOMAR:Ich:NBE0184 | NBE0184 | JF434708 |
| Pomacentridae | *Abudefduf sexfasciatus* | Madagascar:West:Nosy Be | ECOMAR:Ich:NBE0185 | NBE0185 | JF434707 |
| Pomacentridae | *Abudefduf sordidus* | French Polynesia:Society Islands:Moorea | MNHN:Ich:BIOCODES0978 | MBIO1644.4 | JQ431399 |
| Pomacentridae | *Abudefduf sordidus* | Madagascar:West:Nosy Be | ECOMAR:Ich:NBE0669 | NBE0669 | JF434713 |
| Pomacentridae | *Abudefduf sordidus* | Madagascar:West:Nosy Be | ECOMAR:Ich:NBE0670 | NBE0670 | JF434712 |
| Pomacentridae | *Abudefduf sordidus* | Madagascar:West:Nosy Be | ECOMAR:Ich:NBE0671 | NBE0671 | JF434711 |
| Pomacentridae | *Abudefduf sordidus* | Reunion:South:St Philippe | ECOMAR:Ich:REU1682 | REU1682 | JF434710 |
| Pomacentridae | *Abudefduf sordidus* | Reunion:South:St Philippe | ECOMAR:Ich:REU1686 | REU1686 | JF434709 |
| Pomacentridae | *Abudefduf sparoides* | Madagascar:West:Nosy Be | ECOMAR:Ich:NBE0198 | NBE0198 | JF434716 |
| Pomacentridae | *Abudefduf sparoides* | Madagascar:West:Nosy Be | ECOMAR:Ich:NBE0640 | NBE0640 | JF434715 |
| Pomacentridae | *Abudefduf sparoides* | Reunion:West:St Leu | ECOMAR:Ich:REU0969 | REU0969 | JF434719 |
| Pomacentridae | *Abudefduf sparoides* | Reunion:West:St Leu | ECOMAR:Ich:REU0970 | REU0970 | JF434718 |
| Pomacentridae | *Abudefduf sparoides* | Reunion:West:St Leu | ECOMAR:Ich:REU0971 | REU0971 | JF434717 |
| Pomacentridae | *Abudefduf sparoides* | Reunion:West:Hermitage | ECOMAR:Ich:REU195-1 | REU195-1 | JF434714 |
| Pomacentridae | *Abudefduf vaigiensis* | Madagascar:West:Nosy Be | ECOMAR:Ich:NBE0251 | NBE0251 | JF434721 |
| Pomacentridae | *Abudefduf vaigiensis* | Reunion:South:St Philippe | ECOMAR:Ich:REU1687 | REU1687 | JF434720 |
| Pomacentridae | *Amblyglyphidodon indicus* | Madagascar:West:Nosy Be | ECOMAR:Ich:NBE0025 | NBE0025 | JF434725 |
| Pomacentridae | *Amblyglyphidodon indicus* | Madagascar:West:Nosy Be | ECOMAR:Ich:NBE0099 | NBE0099 | JQ349682 |
| Pomacentridae | *Amblyglyphidodon indicus* | Madagascar:West:Nosy Be | ECOMAR:Ich:NBE0601 | NBE0601 | JF434724 |
| Pomacentridae | *Amblyglyphidodon indicus* | Madagascar:West:Nosy Be | ECOMAR:Ich:NBE0602 | NBE0602 | JF434723 |
| Pomacentridae | *Amblyglyphidodon indicus* | Madagascar:West:Nosy Be | ECOMAR:Ich:NBE1321 | NBE1321 | JF434722 |
| Pomacentridae | *Amphiprion akallopisos* | Madagascar:West:Nosy Be | ECOMAR:Ich:NBE1011 | NBE1011 | JF434730 |
| Pomacentridae | *Amphiprion akallopisos* | Madagascar:West:Nosy Be | ECOMAR:Ich:NBE1012 | NBE1012 | JF434729 |
| Pomacentridae | *Amphiprion akallopisos* | Madagascar:West:Nosy Be | ECOMAR:Ich:NBE1013 | NBE1013 | JF434728 |
| Pomacentridae | *Amphiprion akallopisos* | Madagascar:West:Nosy Be | ECOMAR:Ich:NBE1036 | NBE1036 | JF434727 |
| Pomacentridae | *Amphiprion akallopisos* | Madagascar:West:Nosy Be | ECOMAR:Ich:NBE1037 | NBE1037 | JF434726 |
| Pomacentridae | *Amphiprion chrysogaster* | Reunion:West:Hermitage | ECOMAR:Ich:REU0702 | REU0702 | JF434731 |
| Pomacentridae | *Amphiprion chrysopterus* | French Polynesia:Society Islands:Moorea | MNHN:Ich:2008-924 | MBIO1219 | JF434733 |
| Pomacentridae | *Amphiprion chrysopterus* | French Polynesia:Society Islands:Moorea | MNHN:Ich:2008-925 | MBIO1220 | JF434732 |
| Pomacentridae | *Amphiprion latifasciatus* | Madagascar:West:Nosy Be | ECOMAR:Ich:NBE0191 | NBE0191 | JF434737 |
| Pomacentridae | *Amphiprion latifasciatus* | Madagascar:West:Nosy Be | ECOMAR:Ich:NBE1138 | NBE1138 | JF434736 |
| Pomacentridae | *Amphiprion latifasciatus* | Madagascar:West:Nosy Be | ECOMAR:Ich:NBE1139 | NBE1139 | JF434735 |
| Pomacentridae | *Amphiprion latifasciatus* | Madagascar:West:Nosy Be | ECOMAR:Ich:NBE1272 | NBE1272 | JF434734 |
| Pomacentridae | *Chromis acares* | French Polynesia:Society Islands:Moorea | MNHN:Ich:2008-155 | MBIO0025 | JF434865 |
| Pomacentridae | *Chromis acares* | French Polynesia:Society Islands:Moorea | MNHN:Ich:2008-156 | MBIO0026 | JF434864 |
| Pomacentridae | *Chromis agilis* | French Polynesia:Society Islands:Moorea | MNHN:Ich:2008-926 | MBIO1224 | JF434866 |
| Pomacentridae | *Chromis alpha* | French Polynesia:Society Islands:Moorea | MNHN:Ich:2008-783 | MBIO0946 | JF434868 |
| Pomacentridae | *Chromis alpha* | French Polynesia:Society Islands:Moorea | MNHN:Ich:2008-784 | MBIO0947 | JF434867 |
| Pomacentridae | *Chromis atripectoralis* | French Polynesia:Society Islands:Moorea | MNHN:Ich:BIOCODES522 | MBIO763.4 | JQ431630 |
| Pomacentridae | *Chromis atripectoralis* | French Polynesia:Society Islands:Moorea | MNHN:Ich:BIOCODES518 | MBIO764.4 | JQ431629 |
| Pomacentridae | *Chromis atripectoralis* | Madagascar:West:Nosy Be | ECOMAR:Ich:NBE0023 | NBE0023 | JF434872 |
| Pomacentridae | *Chromis atripectoralis* | Madagascar:West:Nosy Be | ECOMAR:Ich:NBE0024 | NBE0024 | JF434871 |
| Pomacentridae | *Chromis atripectoralis* | Madagascar:West:Nosy Be | ECOMAR:Ich:NBE1019 | NBE1019 | JF434870 |
| Pomacentridae | *Chromis atripectoralis* | Madagascar:West:Nosy Be | ECOMAR:Ich:NBE1076 | NBE1076 | JF434869 |
| Pomacentridae | *Chromis chrysura* | Reunion:West:Hermitage | ECOMAR:Ich:REU0710 | REU0710 | JF434876 |
| Pomacentridae | *Chromis chrysura* | Reunion:West:Hermitage | ECOMAR:Ich:REU0711 | REU0711 | JF434875 |
| Pomacentridae | *Chromis chrysura* | Reunion:West:Hermitage | ECOMAR:Ich:REU0712 | REU0712 | JF434874 |
| Pomacentridae | *Chromis chrysura* | Reunion:West:St Leu | ECOMAR:Ich:REU0986 | REU0986 | JF434873 |
| Pomacentridae | *Chromis dimidiata* | French Polynesia:Society Islands:Moorea | MNHN:Ich:2008-873 | MBIO1128 | JF434886 |
| Pomacentridae | *Chromis dimidiata* | French Polynesia:Society Islands:Moorea | MNHN:Ich:2008-874 | MBIO1129 | JF434885 |
| Pomacentridae | *Chromis dimidiata* | Madagascar:West:Nosy Be | ECOMAR:Ich:NBE0056 | NBE0056 | JF434884 |
| Pomacentridae | *Chromis dimidiata* | Madagascar:West:Nosy Be | ECOMAR:Ich:NBE0444 | NBE0444 | JF434882 |
| Pomacentridae | *Chromis dimidiata* | Madagascar:West:Nosy Be | ECOMAR:Ich:NBE0447 | NBE0447 | JF434881 |
| Pomacentridae | *Chromis dimidiata* | Madagascar:West:Nosy Be | ECOMAR:Ich:NBE0448 | NBE0448 | JF434880 |
| Pomacentridae | *Chromis dimidiata* | Reunion:West:Hermitage | ECOMAR:Ich:REU0706 | REU0706 | JF434879 |
| Pomacentridae | *Chromis dimidiata* | Reunion:West:Hermitage | ECOMAR:Ich:REU0707 | REU0707 | JF434878 |
| Pomacentridae | *Chromis dimidiata* | Reunion:West:Hermitage | ECOMAR:Ich:REU0708 | REU0708 | JF434877 |
| Pomacentridae | *Chromis dimidiata* | Reunion:West:St Leu | ECOMAR:Ich:REU0927 | REU0927 | JF434883 |
| Pomacentridae | *Chromis iomelas* | French Polynesia:Society Islands:Moorea | MNHN:Ich:2008-206 | MBIO0095 | JF434887 |
| Pomacentridae | *Chromis iomelas* | French Polynesia:Society Islands:Moorea | MNHN:Ich:BIOCODES0056 | MBIO96.4 | JQ431631 |
| Pomacentridae | *Chromis nigrura* | Madagascar:West:Nosy Be | ECOMAR:Ich:NBE0432 | NBE0432 | JQ349893 |
| Pomacentridae | *Chromis nigrura* | Reunion:West:St Leu | ECOMAR:Ich:REU0996 | REU0996 | JF434889 |
| Pomacentridae | *Chromis nigrura* | Reunion:West:St Leu | ECOMAR:Ich:REU0997 | REU0997 | JF434888 |
| Pomacentridae | *Chromis opercularis* | Madagascar:West:Nosy Be | ECOMAR:Ich:NBE0430 | NBE0430 | JF434891 |
| Pomacentridae | *Chromis opercularis* | Madagascar:West:Nosy Be | ECOMAR:Ich:NBE0431 | NBE0431 | JF434890 |
| Pomacentridae | *Chromis ternatensis* | Madagascar:West:Nosy Be | ECOMAR:Ich:NBE0097 | NBE0097 | JF434897 |
| Pomacentridae | *Chromis ternatensis* | Madagascar:West:Nosy Be | ECOMAR:Ich:NBE0098 | NBE0098 | JF434896 |
| Pomacentridae | *Chromis ternatensis* | Madagascar:West:Nosy Be | ECOMAR:Ich:NBE0440 | NBE0440 | JF434895 |
| Pomacentridae | *Chromis ternatensis* | Madagascar:West:Nosy Be | ECOMAR:Ich:NBE0441 | NBE0441 | JF434894 |
| Pomacentridae | *Chromis ternatensis* | Madagascar:West:Nosy Be | ECOMAR:Ich:NBE0442 | NBE0442 | JF434893 |
| Pomacentridae | *Chromis ternatensis* | Madagascar:West:Nosy Be | ECOMAR:Ich:NBE0649 | NBE0649 | JF434892 |
| Pomacentridae | *Chromis vanderbilti* | French Polynesia:Society Islands:Moorea | MNHN:Ich:2008-345 | MBIO0280 | JF434899 |
| Pomacentridae | *Chromis vanderbilti* | French Polynesia:Society Islands:Moorea | MNHN:Ich:2008-346 | MBIO0281 | JF434898 |
| Pomacentridae | *Chromis viridis* | French Polynesia:Society Islands:Moorea | MNHN:Ich:BIOCODES0342 | MBIO489.4 | JQ431634 |
| Pomacentridae | *Chromis viridis* | French Polynesia:Society Islands:Moorea | MNHN:Ich:BIOCODES0330 | MBIO490.4 | JQ431633 |
| Pomacentridae | *Chromis viridis* | French Polynesia:Society Islands:Moorea | MNHN:Ich:BIOCODES520 | MBIO768.4 | JQ431632 |
| Pomacentridae | *Chromis viridis* | French Polynesia:Society Islands:Moorea | MNHN:Ich:BIOCODES523 | MBIO769.4 | JQ431635 |
| Pomacentridae | *Chromis viridis* | Madagascar:West:Nosy Be | ECOMAR:Ich:NBE1072 | NBE1072 | JF434906 |
| Pomacentridae | *Chromis viridis* | Madagascar:West:Nosy Be | ECOMAR:Ich:NBE1073 | NBE1073 | JF434905 |
| Pomacentridae | *Chromis viridis* | Madagascar:West:Nosy Be | ECOMAR:Ich:NBE1074 | NBE1074 | JF434904 |
| Pomacentridae | *Chromis viridis* | Madagascar:West:Nosy Be | ECOMAR:Ich:NBE1075 | NBE1075 | JF434903 |
| Pomacentridae | *Chromis viridis* | Reunion:West:Hermitage | ECOMAR:Ich:REU0118 | REU0118 | JF434902 |
| Pomacentridae | *Chromis viridis* | Reunion:West:Hermitage | ECOMAR:Ich:REU077-1 | REU077-1 | JF434901 |
| Pomacentridae | *Chromis viridis* | Reunion:West:Hermitage | ECOMAR:Ich:REU269-1 | REU269-1 | JF434900 |
| Pomacentridae | *Chromis weberi* | Madagascar:West:Nosy Be | ECOMAR:Ich:NBE1238 | NBE1238 | JF434908 |
| Pomacentridae | *Chromis weberi* | Madagascar:West:Nosy Be | ECOMAR:Ich:NBE1239 | NBE1239 | JF434907 |
| Pomacentridae | *Chromis xanthochira* | Madagascar:West:Nosy Be | ECOMAR:Ich:NBE0105 | NBE0105 | JF434914 |
| Pomacentridae | *Chromis xanthochira* | Madagascar:West:Nosy Be | ECOMAR:Ich:NBE0112 | NBE0112 | JF434913 |
| Pomacentridae | *Chromis xanthochira* | Madagascar:West:Nosy Be | ECOMAR:Ich:NBE0196 | NBE0196 | JF434912 |
| Pomacentridae | *Chromis xanthochira* | Madagascar:West:Nosy Be | ECOMAR:Ich:NBE0439 | NBE0439 | JF434911 |
| Pomacentridae | *Chromis xanthochira* | Madagascar:West:Nosy Be | ECOMAR:Ich:NBE0595 | NBE0595 | JF434910 |
| Pomacentridae | *Chromis xanthochira* | Reunion:West:Hermitage | ECOMAR:Ich:REU0709 | REU0709 | JF434909 |
| Pomacentridae | *Chromis xanthura* | French Polynesia:Society Islands:Moorea | MNHN:Ich:2008-782 | MBIO0945 | JF434916 |
| Pomacentridae | *Chromis xanthura* | French Polynesia:Society Islands:Moorea | MNHN:Ich:2008-946 | MBIO1264 | JF434915 |
| Pomacentridae | *Chrysiptera annulata* | Madagascar:West:Nosy Be | ECOMAR:Ich:NBE0633 | NBE0633 | JF434918 |
| Pomacentridae | *Chrysiptera annulata* | Madagascar:West:Nosy Be | ECOMAR:Ich:NBE0634 | NBE0634 | JF434917 |
| Pomacentridae | *Chrysiptera brownriggii* | French Polynesia:Society Islands:Moorea | MNHN:Ich:2008-489 | MBIO0493 | JF434930 |
| Pomacentridae | *Chrysiptera brownriggii* | French Polynesia:Society Islands:Moorea | MNHN:Ich:2008-490 | MBIO0494 | JF434929 |
| Pomacentridae | *Chrysiptera brownriggii* | French Polynesia:Society Islands:Moorea | SI:Ich:MBIO1717 | MBIO1717.4 | JQ431636 |
| Pomacentridae | *Chrysiptera brownriggii* | French Polynesia:Society Islands:Moorea | MNHN:Ich:BIOCODES530 | MBIO775.4 | JQ431637 |
| Pomacentridae | *Chrysiptera brownriggii* | Madagascar:West:Nosy Be | ECOMAR:Ich:NBE0032 | NBE0032 | JF434928 |
| Pomacentridae | *Chrysiptera brownriggii* | Madagascar:West:Nosy Be | ECOMAR:Ich:NBE0035 | NBE0035 | JF434927 |
| Pomacentridae | *Chrysiptera brownriggii* | Madagascar:West:Nosy Be | ECOMAR:Ich:NBE0296 | NBE0296 | JF434926 |
| Pomacentridae | *Chrysiptera brownriggii* | Madagascar:West:Nosy Be | ECOMAR:Ich:NBE0297 | NBE0297 | JF434925 |
| Pomacentridae | *Chrysiptera brownriggii* | Madagascar:West:Nosy Be | ECOMAR:Ich:NBE0298 | NBE0298 | JF434924 |
| Pomacentridae | *Chrysiptera brownriggii* | Madagascar:West:Nosy Be | ECOMAR:Ich:NBE0650 | NBE0650 | JF434923 |
| Pomacentridae | *Chrysiptera brownriggii* | Reunion:North:La Possession | ECOMAR:Ich:REU0136 | REU0136 | JF434922 |
| Pomacentridae | *Chrysiptera brownriggii* | Reunion:North:La Possession | ECOMAR:Ich:REU0137 | REU0137 | JF434921 |
| Pomacentridae | *Chrysiptera brownriggii* | Reunion:North:La Possession | ECOMAR:Ich:REU0138 | REU0138 | JF434920 |
| Pomacentridae | *Chrysiptera brownriggii* | Reunion:North:La Possession | ECOMAR:Ich:REU0139 | REU0139 | JF434919 |
| Pomacentridae | *Chrysiptera glauca* | Reunion:West:Hermitage | ECOMAR:Ich:REU003-1 | REU003-1 | JF434934 |
| Pomacentridae | *Chrysiptera glauca* | Reunion:West:Hermitage | ECOMAR:Ich:REU003-2 | REU003-2 | JF434933 |
| Pomacentridae | *Chrysiptera glauca* | Reunion:West:Hermitage | ECOMAR:Ich:REU197-1 | REU197-1 | JF434932 |
| Pomacentridae | *Chrysiptera glauca* | Reunion:West:Hermitage | ECOMAR:Ich:REU197-2 | REU197-2 | JF434931 |
| Pomacentridae | *Chrysiptera unimaculata* | Reunion:West:Hermitage | ECOMAR:Ich:REU1659 | REU1659 | JF435153 |
| Pomacentridae | *Dascyllus aruanus* | French Polynesia:Society Islands:Moorea | MNHN:Ich:BIOCODES0390 | MBIO556.4 | JQ431676 |
| Pomacentridae | *Dascyllus aruanus* | French Polynesia:Society Islands:Moorea | MNHN:Ich:BIOCODES0388 | MBIO557.4 | JQ431677 |
| Pomacentridae | *Dascyllus aruanus* | French Polynesia:Society Islands:Moorea | MNHN:Ich:BIOCODES533 | MBIO786.4 | JQ431675 |
| Pomacentridae | *Dascyllus aruanus* | French Polynesia:Society Islands:Moorea | MNHN:Ich:BIOCODES534 | MBIO787.4 | JQ431674 |
| Pomacentridae | *Dascyllus aruanus* | Madagascar:West:Nosy Be | ECOMAR:Ich:NBE0187 | NBE0187 | JF434955 |
| Pomacentridae | *Dascyllus aruanus* | Madagascar:West:Nosy Be | ECOMAR:Ich:NBE0188 | NBE0188 | JF434954 |
| Pomacentridae | *Dascyllus aruanus* | Madagascar:West:Nosy Be | ECOMAR:Ich:NBE0189 | NBE0189 | JF434953 |
| Pomacentridae | *Dascyllus aruanus* | Reunion:West:Hermitage | ECOMAR:Ich:REU004-1 | REU004-1 | JF434952 |
| Pomacentridae | *Dascyllus aruanus* | Reunion:West:Hermitage | ECOMAR:Ich:REU004-2 | REU004-2 | JF434951 |
| Pomacentridae | *Dascyllus aruanus* | Reunion:West:St Leu | ECOMAR:Ich:REU2764 | REU2764 | JF434950 |
| Pomacentridae | *Dascyllus aruanus* | Reunion:West:St Leu | ECOMAR:Ich:REU2765 | REU2765 | JF434949 |
| Pomacentridae | *Dascyllus carneus* | Madagascar:West:Nosy Be | ECOMAR:Ich:NBE0199 | NBE0199 | JF434959 |
| Pomacentridae | *Dascyllus carneus* | Madagascar:West:Nosy Be | ECOMAR:Ich:NBE0597 | NBE0597 | JF434958 |
| Pomacentridae | *Dascyllus carneus* | Madagascar:West:Nosy Be | ECOMAR:Ich:NBE0598 | NBE0598 | JF434957 |
| Pomacentridae | *Dascyllus carneus* | Madagascar:West:Nosy Be | ECOMAR:Ich:NBE0599 | NBE0599 | JF434956 |
| Pomacentridae | *Dascyllus flavicaudus* | French Polynesia:Society Islands:Moorea | MNHN:Ich:2008-171 | MBIO0045 | JF434961 |
| Pomacentridae | *Dascyllus flavicaudus* | French Polynesia:Society Islands:Moorea | MNHN:Ich:2008-172 | MBIO0046 | JF434960 |
| Pomacentridae | *Dascyllus flavicaudus* | French Polynesia:Society Islands:Moorea | SI:Ich:MBIO773 | MBIO773.4 | JQ431678 |
| Pomacentridae | *Dascyllus trimaculatus* | French Polynesia:Society Islands:Moorea | MNHN:Ich:BIOCODES702 | MBIO1043.4 | JQ431680 |
| Pomacentridae | *Dascyllus trimaculatus* | French Polynesia:Society Islands:Moorea | SI:Ich:MBIO1262 | MBIO1262.4 | JQ431679 |
| Pomacentridae | *Dascyllus trimaculatus* | Madagascar:West:Nosy Be | ECOMAR:Ich:NBE0022 | NBE0022 | JQ349931 |
| Pomacentridae | *Dascyllus trimaculatus* | Madagascar:West:Nosy Be | ECOMAR:Ich:NBE0197 | NBE0197 | JQ349929 |
| Pomacentridae | *Dascyllus trimaculatus* | Madagascar:West:Nosy Be | ECOMAR:Ich:NBE0621 | NBE0621 | JQ349930 |
| Pomacentridae | *Dascyllus trimaculatus* | Reunion:West:St Leu | ECOMAR:Ich:REU0931 | REU0931 | JF434964 |
| Pomacentridae | *Dascyllus trimaculatus* | Reunion:West:St Leu | ECOMAR:Ich:REU0932 | REU0932 | JF434963 |
| Pomacentridae | *Dascyllus trimaculatus* | Reunion:West:St Leu | ECOMAR:Ich:REU0988 | REU0988 | JF434962 |
| Pomacentridae | *Lepidozygus tapeinosoma* | French Polynesia:Society Islands:Moorea | MNHN:Ich:BIOCODES663 | MBIO985.4 | JQ431883 |
| Pomacentridae | *Lepidozygus tapeinosoma* | French Polynesia:Society Islands:Moorea | SI:Ich:MBIO986 | MBIO986.4 | JQ431882 |
| Pomacentridae | *Neoglyphidodon melas* | Madagascar:West:Nosy Be | ECOMAR:Ich:NBE0015 | NBE0015 | JF435053 |
| Pomacentridae | *Neoglyphidodon melas* | Madagascar:West:Nosy Be | ECOMAR:Ich:NBE0660 | NBE0660 | JF435052 |
| Pomacentridae | *Neopomacentrus azysron* | Madagascar:West:Nosy Be | ECOMAR:Ich:NBE0245 | NBE0245 | JF435056 |
| Pomacentridae | *Neopomacentrus azysron* | Madagascar:West:Nosy Be | ECOMAR:Ich:NBE0246 | NBE0246 | JF435055 |
| Pomacentridae | *Neopomacentrus azysron* | Madagascar:West:Nosy Be | ECOMAR:Ich:NBE0247 | NBE0247 | JF435054 |
| Pomacentridae | *Neopomacentrus azysron* | Madagascar:West:Nosy Be | ECOMAR:Ich:NBE0249 | NBE0249 | JQ350143 |
| Pomacentridae | *Neopomacentrus cyanomos* | Madagascar:West:Nosy Be | ECOMAR:Ich:NBE0240 | NBE0240 | JF435059 |
| Pomacentridae | *Neopomacentrus cyanomos* | Madagascar:West:Nosy Be | ECOMAR:Ich:NBE0241 | NBE0241 | JF435058 |
| Pomacentridae | *Neopomacentrus cyanomos* | Madagascar:West:Nosy Be | ECOMAR:Ich:NBE0242 | NBE0242 | JF435057 |
| Pomacentridae | *Neopomacentrus cyanomos* | Madagascar:West:Nosy Be | ECOMAR:Ich:NBE0243 | NBE0243 | JQ350144 |
| Pomacentridae | *Neopomacentrus cyanomos* | Madagascar:West:Nosy Be | ECOMAR:Ich:NBE0244 | NBE0244 | JQ350145 |
| Pomacentridae | *Plectroglyphidodon dickii* | Reunion:West:Hermitage | ECOMAR:Ich:REU0715 | REU0715 | JF435069 |
| Pomacentridae | *Plectroglyphidodon dickii* | Reunion:West:St Leu | ECOMAR:Ich:REU0926 | REU0926 | JF435070 |
| Pomacentridae | *Plectroglyphidodon dickii* | Reunion:West:St Gilles | ECOMAR:Ich:REU110-1 | REU110_1 | JQ350223 |
| Pomacentridae | *Plectroglyphidodon dickii* | Reunion:West:St Gilles | ECOMAR:Ich:REU110-2 | REU110_2 | JQ350224 |
| Pomacentridae | *Plectroglyphidodon imparipennis* | French Polynesia:Society Islands:Moorea | SI:Ich:MBIO1651 | MBIO1651.4 | JQ432006 |
| Pomacentridae | *Plectroglyphidodon imparipennis* | French Polynesia:Society Islands:Moorea | MNHN:Ich:BIOCODES0204 | MBIO295.4 | JQ432005 |
| Pomacentridae | *Plectroglyphidodon imparipennis* | French Polynesia:Society Islands:Moorea | MNHN:Ich:BIOCODES0205 | MBIO296.4 | JQ432007 |
| Pomacentridae | *Plectroglyphidodon imparipennis* | Reunion:South:St Philippe | ECOMAR:Ich:REU1685 | REU1685 | JF435074 |
| Pomacentridae | *Plectroglyphidodon imparipennis* | Reunion:South:St Philippe | ECOMAR:Ich:REU1746 | REU1746 | JF435073 |
| Pomacentridae | *Plectroglyphidodon imparipennis* | Reunion:South:St Philippe | ECOMAR:Ich:REU1747 | REU1747 | JF435072 |
| Pomacentridae | *Plectroglyphidodon imparipennis* | Reunion:South:St Philippe | ECOMAR:Ich:REU1803 | REU1803 | JF435071 |
| Pomacentridae | *Plectroglyphidodon imparipennis* | Reunion:South:St Philippe | ECOMAR:Ich:REU1804 | REU1804 | JQ350225 |
| Pomacentridae | *Plectroglyphidodon imparipennis* | Reunion:South:St Philippe | ECOMAR:Ich:REU1805 | REU1805 | JQ350226 |
| Pomacentridae | *Plectroglyphidodon johnstonianus* | French Polynesia:Society Islands:Moorea | MNHN:Ich:2008-200 | MBIO0087 | JF435079 |
| Pomacentridae | *Plectroglyphidodon johnstonianus* | French Polynesia:Society Islands:Moorea | MNHN:Ich:BIOCODES0050 | MBIO88.4 | JQ432008 |
| Pomacentridae | *Plectroglyphidodon johnstonianus* | Reunion:West:Hermitage | ECOMAR:Ich:REU0716 | REU0716 | JF435078 |
| Pomacentridae | *Plectroglyphidodon johnstonianus* | Reunion:West:Hermitage | ECOMAR:Ich:REU0717 | REU0717 | JF435077 |
| Pomacentridae | *Plectroglyphidodon johnstonianus* | Reunion:West:Hermitage | ECOMAR:Ich:REU109-1 | REU109-1 | JF435076 |
| Pomacentridae | *Plectroglyphidodon johnstonianus* | Reunion:West:St Gilles | ECOMAR:Ich:REU156-1 | REU156_1 | JQ350227 |
| Pomacentridae | *Plectroglyphidodon johnstonianus* | Reunion:West:Hermitage | ECOMAR:Ich:REU156-2 | REU156-2 | JF435075 |
| Pomacentridae | *Plectroglyphidodon johnstonianus* | Reunion:West:St Leu | ECOMAR:Ich:REU1916 | REU1916 | JQ350228 |
| Pomacentridae | *Plectroglyphidodon lacrymatus* | French Polynesia:Society Islands:Moorea | MNHN:Ich:BIOCODES0059 | MBIO100.4 | JQ432011 |
| Pomacentridae | *Plectroglyphidodon lacrymatus* | French Polynesia:Society Islands:Moorea | MNHN:Ich:BIOCODES0060 | MBIO101.4 | JQ432012 |
| Pomacentridae | *Plectroglyphidodon lacrymatus* | French Polynesia:Society Islands:Moorea | SI:Ich:MBIO1265 | MBIO1265.4 | JQ432010 |
| Pomacentridae | *Plectroglyphidodon lacrymatus* | French Polynesia:Society Islands:Moorea | MNHN:Ich:BIOCODES0128 | MBIO187.4 | JQ432009 |
| Pomacentridae | *Plectroglyphidodon lacrymatus* | Madagascar:West:Nosy Be | ECOMAR:Ich:NBE0036 | NBE0036 | JF435084 |
| Pomacentridae | *Plectroglyphidodon lacrymatus* | Madagascar:West:Nosy Be | ECOMAR:Ich:NBE0037 | NBE0037 | JF435083 |
| Pomacentridae | *Plectroglyphidodon lacrymatus* | Madagascar:West:Nosy Be | ECOMAR:Ich:NBE0038 | NBE0038 | JF435082 |
| Pomacentridae | *Plectroglyphidodon lacrymatus* | Madagascar:West:Nosy Be | ECOMAR:Ich:NBE0039 | NBE0039 | JF435081 |
| Pomacentridae | *Plectroglyphidodon lacrymatus* | Madagascar:West:Nosy Be | ECOMAR:Ich:NBE1270 | NBE1270 | JF435080 |
| Pomacentridae | *Plectroglyphidodon leucozonus* | French Polynesia:Society Islands:Moorea | MNHN:Ich:MBIO1673 | MBIO1673.4 | JQ432013 |
| Pomacentridae | *Plectroglyphidodon leucozonus* | French Polynesia:Society Islands:Moorea | MNHN:Ich:BIOCODES529 | MBIO774.4 | JQ432014 |
| Pomacentridae | *Plectroglyphidodon leucozonus* | Madagascar:West:Nosy Be | ECOMAR:Ich:NBE0635 | NBE0635 | JF435086 |
| Pomacentridae | *Plectroglyphidodon leucozonus* | Madagascar:West:Nosy Be | ECOMAR:Ich:NBE0636 | NBE0636 | JF435085 |
| Pomacentridae | *Plectroglyphidodon phoenixensis* | French Polynesia:Society Islands:Moorea | MNHN:Ich:2008-1098 | MBIO1652 | JF435088 |
| Pomacentridae | *Plectroglyphidodon phoenixensis* | French Polynesia:Society Islands:Moorea | MNHN:Ich:BIOCODES0975 | MBIO1653.4 | JQ432015 |
| Pomacentridae | *Plectroglyphidodon phoenixensis* | French Polynesia:Society Islands:Moorea | MNHN:Ich:BIOCODES0199 | MBIO288.4 | JQ432016 |
| Pomacentridae | *Plectroglyphidodon phoenixensis* | French Polynesia:Society Islands:Moorea | MNHN:Ich:BIOCODES0200 | MBIO289.4 | JQ432017 |
| Pomacentridae | *Plectroglyphidodon phoenixensis* | Reunion:South:St Philippe | ECOMAR:Ich:REU1745 | REU1745 | JF435087 |
| Pomacentridae | *Plectroglyphidodon randalli* | Reunion:North:La Possession | ECOMAR:Ich:REU0140 | REU0140 | JF435092 |
| Pomacentridae | *Plectroglyphidodon randalli* | Reunion:North:La Possession | ECOMAR:Ich:REU0141 | REU0141 | JF435091 |
| Pomacentridae | *Plectroglyphidodon randalli* | Reunion:North:La Possession | ECOMAR:Ich:REU0142 | REU0142 | JF435090 |
| Pomacentridae | *Plectroglyphidodon randalli* | Reunion:North:La Possession | ECOMAR:Ich:REU0143 | REU0143 | JF435089 |
| Pomacentridae | *Pomacentrus agassizii* | Reunion:West:Hermitage | ECOMAR:Ich:REU0713 | REU0713 | JF435096 |
| Pomacentridae | *Pomacentrus agassizii* | Reunion:West:Hermitage | ECOMAR:Ich:REU0714 | REU0714 | JF435095 |
| Pomacentridae | *Pomacentrus agassizii* | Reunion:West:St Leu | ECOMAR:Ich:REU0933 | REU0933 | JF435098 |
| Pomacentridae | *Pomacentrus agassizii* | Reunion:West:St Leu | ECOMAR:Ich:REU0934 | REU0934 | JF435097 |
| Pomacentridae | *Pomacentrus agassizii* | Reunion:West:St Leu | ECOMAR:Ich:REU0983 | REU0983 | JF435094 |
| Pomacentridae | *Pomacentrus agassizii* | Reunion:West:La Saline | ECOMAR:Ich:REU2832 | REU2832 | JQ350239 |
| Pomacentridae | *Pomacentrus agassizii* | Reunion:West:La Saline | ECOMAR:Ich:REU2834 | REU2834 | JQ350240 |
| Pomacentridae | *Pomacentrus arabicus* | Madagascar:West:Nosy Be | ECOMAR:Ich:NBE1556 | NBE1556 | JF435099 |
| Pomacentridae | *Pomacentrus baenschi* | Madagascar:West:Nosy Be | ECOMAR:Ich:NBE0057 | NBE0057 | JF435108 |
| Pomacentridae | *Pomacentrus baenschi* | Madagascar:West:Nosy Be | ECOMAR:Ich:NBE0060 | NBE0060 | JF435107 |
| Pomacentridae | *Pomacentrus baenschi* | Madagascar:West:Nosy Be | ECOMAR:Ich:NBE0061 | NBE0061 | JF435106 |
| Pomacentridae | *Pomacentrus baenschi* | Madagascar:West:Nosy Be | ECOMAR:Ich:NBE0062 | NBE0062 | JF435105 |
| Pomacentridae | *Pomacentrus baenschi* | Madagascar:West:Nosy Be | ECOMAR:Ich:NBE0063 | NBE0063 | JF435104 |
| Pomacentridae | *Pomacentrus baenschi* | Madagascar:West:Nosy Be | ECOMAR:Ich:NBE0373 | NBE0373 | JF435103 |
| Pomacentridae | *Pomacentrus baenschi* | Madagascar:West:Nosy Be | ECOMAR:Ich:NBE0374 | NBE0374 | JF435102 |
| Pomacentridae | *Pomacentrus baenschi* | Madagascar:West:Nosy Be | ECOMAR:Ich:NBE1719 | NBE1719 | JF435101 |
| Pomacentridae | *Pomacentrus baenschi* | Madagascar:West:Nosy Be | ECOMAR:Ich:NBE1720 | NBE1720 | JF435100 |
| Pomacentridae | *Pomacentrus caeruleus* | Madagascar:West:Nosy Be | ECOMAR:Ich:NBE0055 | NBE0055 | JF435115 |
| Pomacentridae | *Pomacentrus caeruleus* | Madagascar:West:Nosy Be | ECOMAR:Ich:NBE0372 | NBE0372 | JF435112 |
| Pomacentridae | *Pomacentrus caeruleus* | Madagascar:West:Nosy Be | ECOMAR:Ich:NBE0388 | NBE0388 | JF435111 |
| Pomacentridae | *Pomacentrus caeruleus* | Madagascar:West:Nosy Be | ECOMAR:Ich:NBE1197 | NBE1197 | JF435110 |
| Pomacentridae | *Pomacentrus caeruleus* | Reunion:West:Hermitage | ECOMAR:Ich:REU0704 | REU0704 | JF435109 |
| Pomacentridae | *Pomacentrus caeruleus* | Reunion:West:St Leu | ECOMAR:Ich:REU0928 | REU0928 | JF435114 |
| Pomacentridae | *Pomacentrus caeruleus* | Reunion:West:St Leu | ECOMAR:Ich:REU0929 | REU0929 | JF435113 |
| Pomacentridae | *Pomacentrus pavo* | French Polynesia:Society Islands:Moorea | MNHN:Ich:2008-540 | MBIO0565 | JF435124 |
| Pomacentridae | *Pomacentrus pavo* | French Polynesia:Society Islands:Moorea | MNHN:Ich:2008-541 | MBIO0566 | JF435123 |
| Pomacentridae | *Pomacentrus pavo* | French Polynesia:Society Islands:Moorea | MNHN:Ich:BIOCODES1002 | MBIO1748.4 | JQ432030 |
| Pomacentridae | *Pomacentrus pavo* | Madagascar:West:Nosy Be | ECOMAR:Ich:NBE0256 | NBE0256 | JF435122 |
| Pomacentridae | *Pomacentrus pavo* | Madagascar:West:Nosy Be | ECOMAR:Ich:NBE0257 | NBE0257 | JF435121 |
| Pomacentridae | *Pomacentrus pavo* | Madagascar:West:Nosy Be | ECOMAR:Ich:NBE0258 | NBE0258 | JF435120 |
| Pomacentridae | *Pomacentrus pavo* | Madagascar:West:Nosy Be | ECOMAR:Ich:NBE0259 | NBE0259 | JF435119 |
| Pomacentridae | *Pomacentrus pavo* | Madagascar:West:Nosy Be | ECOMAR:Ich:NBE0622 | NBE0622 | JF435118 |
| Pomacentridae | *Pomacentrus pavo* | Madagascar:West:Nosy Be | ECOMAR:Ich:NBE0623 | NBE0623 | JF435117 |
| Pomacentridae | *Pomacentrus pavo* | Madagascar:West:Nosy Be | ECOMAR:Ich:NBE0624 | NBE0624 | JF435116 |
| Pomacentridae | *Pomacentrus sulfureus* | Madagascar:West:Nosy Be | ECOMAR:Ich:NBE0092 | NBE0092 | JF435128 |
| Pomacentridae | *Pomacentrus sulfureus* | Madagascar:West:Nosy Be | ECOMAR:Ich:NBE0093 | NBE0093 | JF435127 |
| Pomacentridae | *Pomacentrus sulfureus* | Madagascar:West:Nosy Be | ECOMAR:Ich:NBE0094 | NBE0094 | JF435126 |
| Pomacentridae | *Pomacentrus sulfureus* | Madagascar:West:Nosy Be | ECOMAR:Ich:NBE0095 | NBE0095 | JQ350242 |
| Pomacentridae | *Pomacentrus sulfureus* | Madagascar:West:Nosy Be | ECOMAR:Ich:NBE0096 | NBE0096 | JQ350241 |
| Pomacentridae | *Pomacentrus sulfureus* | Madagascar:West:Nosy Be | ECOMAR:Ich:NBE0594 | NBE0594 | JF435125 |
| Pomacentridae | *Pomacentrus trichourus* | Madagascar:West:Nosy Be | ECOMAR:Ich:NBE0087 | NBE0087 | JQ350243 |
| Pomacentridae | *Pomacentrus trichourus* | Madagascar:West:Nosy Be | ECOMAR:Ich:NBE0088 | NBE0088 | JQ350245 |
| Pomacentridae | *Pomacentrus trichourus* | Madagascar:West:Nosy Be | ECOMAR:Ich:NBE0089 | NBE0089 | JF435130 |
| Pomacentridae | *Pomacentrus trichourus* | Madagascar:West:Nosy Be | ECOMAR:Ich:NBE0090 | NBE0090 | JF435129 |
| Pomacentridae | *Pomacentrus trichourus* | Madagascar:West:Nosy Be | ECOMAR:Ich:NBE0091 | NBE0091 | JQ350244 |
| Pomacentridae | *Pomacentrus trilineatus* | Madagascar:West:Nosy Be | ECOMAR:Ich:NBE0264 | NBE0264 | JF435136 |
| Pomacentridae | *Pomacentrus trilineatus* | Madagascar:West:Nosy Be | ECOMAR:Ich:NBE0265 | NBE0265 | JF435135 |
| Pomacentridae | *Pomacentrus trilineatus* | Madagascar:West:Nosy Be | ECOMAR:Ich:NBE0383 | NBE0383 | JF435134 |
| Pomacentridae | *Pomacentrus trilineatus* | Madagascar:West:Nosy Be | ECOMAR:Ich:NBE0497 | NBE0497 | JF435133 |
| Pomacentridae | *Pomacentrus trilineatus* | Madagascar:West:Nosy Be | ECOMAR:Ich:NBE1015 | NBE1015 | JF435132 |
| Pomacentridae | *Pomacentrus trilineatus* | Madagascar:West:Nosy Be | ECOMAR:Ich:NBE1271 | NBE1271 | JF435131 |
| Pomacentridae | *Pomachromis fuscidorsalis* | French Polynesia:Society Islands:Moorea | MNHN:Ich:2008-790 | MBIO0957 | JF435138 |
| Pomacentridae | *Pomachromis fuscidorsalis* | French Polynesia:Society Islands:Moorea | MNHN:Ich:2008-791 | MBIO0958 | JF435137 |
| Pomacentridae | *Stegastes albifasciatus* | French Polynesia:Society Islands:Moorea | MNHN:Ich:2008-638 | MBIO0732 | JF435152 |
| Pomacentridae | *Stegastes albifasciatus* | French Polynesia:Society Islands:Moorea | MNHN:Ich:BIOCODES0318 | MBIO462.4 | JQ432162 |
| Pomacentridae | *Stegastes albifasciatus* | French Polynesia:Society Islands:Moorea | MNHN:Ich:BIOCODES0319 | MBIO463.4 | JQ432163 |
| Pomacentridae | *Stegastes fasciolatus* | French Polynesia:Society Islands:Moorea | MNHN:Ich:BIOCODES0207 | MBIO299.4 | JQ432164 |
| Pomacentridae | *Stegastes fasciolatus* | French Polynesia:Society Islands:Moorea | MNHN:Ich:BIOCODES0206 | MBIO300.4 | JQ432165 |
| Pomacentridae | *Stegastes limbatus* | Reunion:West:Hermitage | ECOMAR:Ich:REU002-1 | REU002-1 | JF435160 |
| Pomacentridae | *Stegastes limbatus* | Reunion:West:Hermitage | ECOMAR:Ich:REU002-2 | REU002-2 | JF435159 |
| Pomacentridae | *Stegastes limbatus* | Reunion:West:Hermitage | ECOMAR:Ich:REU1654 | REU1654 | JF435158 |
| Pomacentridae | *Stegastes limbatus* | Reunion:West:St Leu | ECOMAR:Ich:REU2774 | REU2774 | JF435157 |
| Pomacentridae | *Stegastes limbatus* | Reunion:West:St Leu | ECOMAR:Ich:REU2775 | REU2775 | JF435156 |
| Pomacentridae | *Stegastes limbatus* | Reunion:West:St Leu | ECOMAR:Ich:REU2776 | REU2776 | JF435155 |
| Pomacentridae | *Stegastes limbatus* | Reunion:West:St Leu | ECOMAR:Ich:REU2777 | REU2777 | JF435154 |
| Pomacentridae | *Stegastes lividus* | Reunion:West:Hermitage | ECOMAR:Ich:REU001-1 | REU001-1 | JF435164 |
| Pomacentridae | *Stegastes lividus* | Reunion:West:Hermitage | ECOMAR:Ich:REU001-2 | REU001-2 | JF435163 |
| Pomacentridae | *Stegastes lividus* | Reunion:West:St Leu | ECOMAR:Ich:REU2754 | REU2754 | JF435162 |
| Pomacentridae | *Stegastes lividus* | Reunion:West:St Leu | ECOMAR:Ich:REU2755 | REU2755 | JF435161 |
| Pomacentridae | *Stegastes nigricans* | French Polynesia:Society Islands:Moorea | MNHN:Ich:BIOCODES0230 | MBIO336.4 | JQ432166 |
| Pomacentridae | *Stegastes nigricans* | French Polynesia:Society Islands:Moorea | MNHN:Ich:BIOCODES0231 | MBIO337.4 | JQ432167 |
| Pomacentridae | *Stegastes nigricans* | Madagascar:West:Nosy Be | ECOMAR:Ich:NBE1085 | NBE1085 | JF435170 |
| Pomacentridae | *Stegastes nigricans* | Madagascar:West:Nosy Be | ECOMAR:Ich:NBE1269 | NBE1269 | JF435169 |
| Pomacentridae | *Stegastes nigricans* | Reunion:West:Hermitage | ECOMAR:Ich:REU005-1 | REU005-1 | JF435168 |
| Pomacentridae | *Stegastes nigricans* | Reunion:West:Hermitage | ECOMAR:Ich:REU111-1 | REU111-1 | JF435167 |
| Pomacentridae | *Stegastes nigricans* | Reunion:West:St Leu | ECOMAR:Ich:REU2661 | REU2661 | JF435166 |
| Pomacentridae | *Stegastes nigricans* | Reunion:West:St Leu | ECOMAR:Ich:REU2784 | REU2784 | JF435165 |
| Pomacentridae | *Stegastes pelicieri* | Reunion:West:Hermitage | ECOMAR:Ich:REU0703 | REU0703 | JF435173 |
| Pomacentridae | *Stegastes pelicieri* | Reunion:West:St Leu | ECOMAR:Ich:REU1909 | REU1909 | JF435172 |
| Pomacentridae | *Stegastes pelicieri* | Reunion:West:St Leu | ECOMAR:Ich:REU1910 | REU1910 | JQ350371 |
| Pomacentridae | *Stegastes pelicieri* | Reunion:West:La Saline | ECOMAR:Ich:REU207-2 | REU207_2 | JQ350370 |
| Pomacentridae | *Stegastes pelicieri* | Reunion:West:Hermitage | ECOMAR:Ich:REU207-1 | REU207-1 | JF435171 |
| Priacanthidae | *Heteropriacanthus cruentatus* | French Polynesia:Society Islands:Moorea | MNHN:Ich:BIOCODES691 | MBIO1028.4 | JQ431859 |
| Priacanthidae | *Heteropriacanthus cruentatus* | French Polynesia:Society Islands:Moorea | MNHN:Ich:BIOCODES692 | MBIO1029.4 | JQ431858 |
| Priacanthidae | *Priacanthus hamrur* | French Polynesia:Society Islands:Moorea | MNHN:Ich:BIOCODES0954 | MBIO1613.4 | JQ432031 |
| Priacanthidae | *Priacanthus hamrur* | Madagascar:West:Nosy Be | ECOMAR:Ich:NBE0541 | NBE0541 | JQ350246 |
| Priacanthidae | *Priacanthus hamrur* | Madagascar:West:Nosy Be | ECOMAR:Ich:NBE1097 | NBE1097 | JQ350247 |
| Priacanthidae | *Priacanthus hamrur* | Reunion:West:St Gilles | ECOMAR:Ich:REU0776 | REU0776 | JQ350248 |
| Pseudochromidae | *Pseudochromis kristinae* | Madagascar:West:Nosy Be | ECOMAR:Ich:NBE0132 | NBE0132 | JQ350270 |
| Pseudochromidae | *Pseudochromis kristinae* | Madagascar:West:Nosy Be | ECOMAR:Ich:NBE0133 | NBE0133 | JQ350269 |
| Pseudochromidae | *Pseudochromis kristinae* | Madagascar:West:Nosy Be | ECOMAR:Ich:NBE0134 | NBE0134 | JQ350268 |
| Pseudochromidae | *Pseudochromis kristinae* | Madagascar:West:Nosy Be | ECOMAR:Ich:NBE0416 | NBE0416 | JQ350273 |
| Pseudochromidae | *Pseudochromis kristinae* | Madagascar:West:Nosy Be | ECOMAR:Ich:NBE0417 | NBE0417 | JQ350274 |
| Pseudochromidae | *Pseudochromis kristinae* | Madagascar:West:Nosy Be | ECOMAR:Ich:NBE0484 | NBE0484 | JQ350275 |
| Pseudochromidae | *Pseudochromis kristinae* | Madagascar:West:Nosy Be | ECOMAR:Ich:NBE0485 | NBE0485 | JQ350267 |
| Pseudochromidae | *Pseudochromis kristinae* | Madagascar:West:Nosy Be | ECOMAR:Ich:NBE0562 | NBE0562 | JQ350272 |
| Pseudochromidae | *Pseudochromis kristinae* | Madagascar:West:Nosy Be | ECOMAR:Ich:NBE1172 | NBE1172 | JQ350271 |
| Pseudochromidae | *Pseudochromis madagascariensis* | Madagascar:West:Nosy Be | ECOMAR:Ich:NBE0283 | NBE0283 | JQ350276 |
| Pseudochromidae | *Pseudochromis madagascariensis* | Madagascar:West:Nosy Be | ECOMAR:Ich:NBE1007 | NBE1007 | JQ350277 |
| Pseudochromidae | *Pseudochromis madagascariensis* | Madagascar:West:Nosy Be | ECOMAR:Ich:NBE1008 | NBE1008 | JQ350278 |
| Pseudochromidae | *Pseudochromis tauberae* | Madagascar:West:Nosy Be | ECOMAR:Ich:NBE0154 | NBE0154 | JQ350279 |
| Pseudochromidae | *Pseudochromis tauberae* | Madagascar:West:Nosy Be | ECOMAR:Ich:NBE0157 | NBE0157 | JQ350281 |
| Pseudochromidae | *Pseudochromis tauberae* | Madagascar:West:Nosy Be | ECOMAR:Ich:NBE0496 | NBE0496 | JQ350280 |
| Pseudochromidae | *Pseudoplesiops revellei* | French Polynesia:Society Islands:Moorea | MNHN:Ich:BIOCODES837 | MBIO1312.4 | JQ432067 |
| Pseudochromidae | *Pseudoplesiops revellei* | French Polynesia:Society Islands:Moorea | SI:Ich:MBIO1313 | MBIO1313.4 | JQ432068 |
| Pseudochromidae | *Pseudoplesiops revellei* | French Polynesia:Society Islands:Moorea | MNHN:Ich:BIOCODES0096 | MBIO146.4 | JQ432066 |
| Samaridae | *Samariscus triocellatus* | French Polynesia:Society Islands:Moorea | MNHN:Ich:MBIO1386 | MBIO1386.5 | JQ432091 |
| Samaridae | *Samariscus triocellatus* | French Polynesia:Society Islands:Moorea | SI:Ich:MBIO1413 | MBIO1413.4 | JQ432092 |
| Scaridae | *Calotomus carolinus* | French Polynesia:Society Islands:Moorea | MNHN:Ich:BIOCODES0876 | MBIO1452.4 | JQ431515 |
| Scaridae | *Calotomus carolinus* | French Polynesia:Society Islands:Moorea | SI:Ich:MBIO1453 | MBIO1453.4 | JQ431516 |
| Scaridae | *Calotomus carolinus* | French Polynesia:Society Islands:Moorea | MNHN:Ich:BIOCODES0877 | MBIO1454.4 | JQ431517 |
| Scaridae | *Calotomus carolinus* | Madagascar:West:Nosy Be | ECOMAR:Ich:NBE0664 | NBE0664 | JQ349815 |
| Scaridae | *Cetoscarus bicolor* | French Polynesia:Society Islands:Moorea | MNHN:Ich:BIOCODES1004 | MBIO1761.4 | JQ431580 |
| Scaridae | *Cetoscarus bicolor* | Madagascar:West:Nosy Be | ECOMAR:Ich:NBE0029 | NBE0029 | JQ349875 |
| Scaridae | *Cetoscarus bicolor* | Madagascar:West:Nosy Be | ECOMAR:Ich:NBE1306 | NBE1306 | JQ349874 |
| Scaridae | *Chlorurus frontalis* | French Polynesia:Society Islands:Moorea | MNHN:Ich:BIOCODES792 | MBIO1234.4 | JQ431619 |
| Scaridae | *Chlorurus frontalis* | French Polynesia:Society Islands:Moorea | MNHN:Ich:BIOCODES823 | MBIO1293.4 | JQ431618 |
| Scaridae | *Chlorurus frontalis* | French Polynesia:Society Islands:Moorea | MNHN:Ich:BIOCODES602 | MBIO894.4 | JQ431620 |
| Scaridae | *Chlorurus frontalis* | French Polynesia:Society Islands:Moorea | MNHN:Ich:BIOCODES604 | MBIO895.4 | JQ431617 |
| Scaridae | *Chlorurus sordidus* | French Polynesia:Society Islands:Moorea | MNHN:Ich:BIOCODES0868 | MBIO1440.4 | JQ431621 |
| Scaridae | *Chlorurus sordidus* | French Polynesia:Society Islands:Moorea | MNHN:Ich:BIOCODES0869 | MBIO1441.4 | JQ431626 |
| Scaridae | *Chlorurus sordidus* | French Polynesia:Society Islands:Moorea | MNHN:Ich:BIOCODES0314 | MBIO455.4 | JQ431625 |
| Scaridae | *Chlorurus sordidus* | French Polynesia:Society Islands:Moorea | MNHN:Ich:BIOCODES0338 | MBIO483.4 | JQ431624 |
| Scaridae | *Chlorurus sordidus* | French Polynesia:Society Islands:Moorea | MNHN:Ich:BIOCODES624 | MBIO922.4 | JQ431623 |
| Scaridae | *Chlorurus sordidus* | French Polynesia:Society Islands:Moorea | MNHN:Ich:BIOCODES623 | MBIO923.4 | JQ431622 |
| Scaridae | *Chlorurus sordidus* | Madagascar:West:Nosy Be | ECOMAR:Ich:NBE1303 | NBE1303 | JQ349891 |
| Scaridae | *Chlorurus sordidus* | Reunion:West:St Gilles | ECOMAR:Ich:REU302-1 | REU302_1 | JQ349892 |
| Scaridae | *Leptoscarus vaigiensis* | French Polynesia:Society Islands:Moorea | MNHN:Ich:BIOCODES1040 | MBIO1853.4 | JQ431884 |
| Scaridae | *Scarus altipinnis* | French Polynesia:Society Islands:Moorea | SI:Ich:MBIO1294 | MBIO1294.4 | JQ432095 |
| Scaridae | *Scarus forsteni* | French Polynesia:Society Islands:Moorea | MNHN:Ich:BIOCODES817 | MBIO1275.4 | JQ432096 |
| Scaridae | *Scarus forsteni* | French Polynesia:Society Islands:Moorea | MNHN:Ich:BIOCODES0923 | MBIO1563.4 | JQ432097 |
| Scaridae | *Scarus ghobban* | Madagascar:West:Nosy Be | ECOMAR:Ich:NBE1099 | NBE1099 | JQ350328 |
| Scaridae | *Scarus ghobban* | Reunion:West:St Gilles | ECOMAR:Ich:REU006-1 | REU006_1 | JQ350327 |
| Scaridae | *Scarus ghobban* | Reunion:West:La Saline | ECOMAR:Ich:REU0299 | REU0299 | JQ350329 |
| Scaridae | *Scarus globiceps* | French Polynesia:Society Islands:Moorea | MNHN:Ich:BIOCODES0872 | MBIO1444.4 | JQ432101 |
| Scaridae | *Scarus globiceps* | French Polynesia:Society Islands:Moorea | MNHN:Ich:BIOCODES0873 | MBIO1445.4 | JQ432098 |
| Scaridae | *Scarus globiceps* | French Polynesia:Society Islands:Moorea | MNHN:Ich:BIOCODES0874 | MBIO1450.4 | JQ432100 |
| Scaridae | *Scarus globiceps* | French Polynesia:Society Islands:Moorea | MNHN:Ich:BIOCODES0424 | MBIO605.4 | JQ432099 |
| Scaridae | *Scarus globiceps* | French Polynesia:Society Islands:Moorea | MNHN:Ich:BIOCODES600 | MBIO885.4 | JQ432103 |
| Scaridae | *Scarus globiceps* | French Polynesia:Society Islands:Moorea | SI:Ich:MBIO886 | MBIO886.4 | JQ432102 |
| Scaridae | *Scarus globiceps* | Madagascar:West:Nosy Be | ECOMAR:Ich:NBE1308 | NBE1308 | JQ350330 |
| Scaridae | *Scarus niger* | French Polynesia:Society Islands:Moorea | SI:Ich:MBIO1232 | MBIO1232.4 | JQ432105 |
| Scaridae | *Scarus niger* | French Polynesia:Society Islands:Moorea | MNHN:Ich:BIOCODES622 | MBIO921.4 | JQ432104 |
| Scaridae | *Scarus niger* | Madagascar:West:Nosy Be | ECOMAR:Ich:NBE0067 | NBE0067 | JQ350332 |
| Scaridae | *Scarus niger* | Madagascar:West:Nosy Be | ECOMAR:Ich:NBE1298 | NBE1298 | JQ350331 |
| Scaridae | *Scarus oviceps* | French Polynesia:Society Islands:Moorea | MNHN:Ich:BIOCODES0870 | MBIO1443.4 | JQ432108 |
| Scaridae | *Scarus oviceps* | French Polynesia:Society Islands:Moorea | MNHN:Ich:BIOCODES603 | MBIO891.4 | JQ432107 |
| Scaridae | *Scarus oviceps* | French Polynesia:Society Islands:Moorea | MNHN:Ich:BIOCODES601 | MBIO892.4 | JQ432106 |
| Scaridae | *Scarus psittacus* | French Polynesia:Society Islands:Moorea | MNHN:Ich:BIOCODES0871 | MBIO1448.4 | JQ432109 |
| Scaridae | *Scarus psittacus* | French Polynesia:Society Islands:Moorea | SI:Ich:MBIO1449 | MBIO1449.4 | JQ432111 |
| Scaridae | *Scarus psittacus* | French Polynesia:Society Islands:Moorea | MNHN:Ich:BIOCODES0425 | MBIO606.4 | JQ432110 |
| Scaridae | *Scarus psittacus* | French Polynesia:Society Islands:Moorea | MNHN:Ich:BIOCODES599 | MBIO889.4 | JQ432113 |
| Scaridae | *Scarus psittacus* | French Polynesia:Society Islands:Moorea | SI:Ich:MBIO890 | MBIO890.4 | JQ432112 |
| Scaridae | *Scarus scaber* | Madagascar:West:Nosy Be | ECOMAR:Ich:NBE1305 | NBE1305 | JQ350334 |
| Scaridae | *Scarus scaber* | Reunion:West:St Gilles | ECOMAR:Ich:REU148-1 | REU148_1 | JQ350333 |
| Scaridae | *Scarus schlegeli* | French Polynesia:Society Islands:Moorea | MNHN:Ich:BIOCODES791 | MBIO1235.4 | JQ432114 |
| Scaridae | *Scarus tricolor* | Madagascar:West:Nosy Be | ECOMAR:Ich:NBE1098 | NBE1098 | JQ350335 |
| Scombridae | *Gymnosarda unicolor* | French Polynesia:Society Islands:Moorea | MNHN:Ich:BIOCODES819 | MBIO1285.4 | JQ431785 |
| Scombridae | *Gymnosarda unicolor* | French Polynesia:Society Islands:Moorea | SI:Ich:MBIO1830 | MBIO1830.4 | JQ431784 |
| Scorpaenidae | *Dendrochirus biocellatus* | French Polynesia:Society Islands:Moorea | MNHN:Ich:BIOCODES0934 | MBIO1580.4 | JQ431683 |
| Scorpaenidae | *Dendrochirus biocellatus* | French Polynesia:Society Islands:Moorea | SI:Ich:MBIO1581 | MBIO1581.4 | JQ431684 |
| Scorpaenidae | *Dendrochirus biocellatus* | Reunion:West:St Leu | ECOMAR:Ich:REU1027 | REU1027 | JQ349932 |
| Scorpaenidae | *Parascorpaena mossambica* | French Polynesia:Society Islands:Moorea | MNHN:Ich:BIOCODES0936 | MBIO1582.4 | JQ431973 |
| Scorpaenidae | *Parascorpaena mossambica* | French Polynesia:Society Islands:Moorea | MNHN:Ich:BIOCODES0932 | MBIO1583.4 | JQ431974 |
| Scorpaenidae | *Pterois antennata* | French Polynesia:Society Islands:Moorea | MNHN:Ich:BIOCODES0452 | MBIO661.4 | JQ432075 |
| Scorpaenidae | *Pterois antennata* | French Polynesia:Society Islands:Moorea | SI:Ich:MBIO662 | MBIO662.4 | JQ432074 |
| Scorpaenidae | *Pterois antennata* | French Polynesia:Society Islands:Moorea | MNHN:Ich:BIOCODES576 | MBIO850.4 | JQ432076 |
| Scorpaenidae | *Pterois antennata* | French Polynesia:Society Islands:Moorea | SI:Ich:MBIO851 | MBIO851.4 | JQ432077 |
| Scorpaenidae | *Pterois antennata* | Madagascar:West:Nosy Be | ECOMAR:Ich:NBE0619 | NBE0619 | JQ350293 |
| Scorpaenidae | *Pterois antennata* | Reunion:West:La Saline | ECOMAR:Ich:REU2720 | REU2720 | JQ350294 |
| Scorpaenidae | *Pterois miles* | Madagascar:West:Nosy Be | ECOMAR:Ich:NBE0387 | NBE0387 | JQ350297 |
| Scorpaenidae | *Pterois miles* | Madagascar:West:Nosy Be | ECOMAR:Ich:NBE0462 | NBE0462 | JQ350296 |
| Scorpaenidae | *Pterois miles* | Madagascar:West:Nosy Be | ECOMAR:Ich:NBE0618 | NBE0618 | JQ350295 |
| Scorpaenidae | *Pterois radiata* | French Polynesia:Society Islands:Moorea | MNHN:Ich:BIOCODES0311 | MBIO452.4 | JQ432078 |
| Scorpaenidae | *Pterois radiata* | French Polynesia:Society Islands:Moorea | MNHN:Ich:BIOCODES573 | MBIO848.4 | JQ432079 |
| Scorpaenidae | *Pterois radiata* | French Polynesia:Society Islands:Moorea | SI:Ich:MBIO849 | MBIO849.4 | JQ432080 |
| Scorpaenidae | *Scorpaenodes corallinus* | French Polynesia:Society Islands:Moorea | MNHN:Ich:BIOCODES0918 | MBIO1539.4 | JQ432121 |
| Scorpaenidae | *Scorpaenodes corallinus* | French Polynesia:Society Islands:Moorea | SI:Ich:MBIO349 | MBIO349.4 | JQ432120 |
| Scorpaenidae | *Scorpaenodes guamensis* | French Polynesia:Society Islands:Moorea | MNHN:Ich:BIOCODES0235 | MBIO344.4 | JQ432122 |
| Scorpaenidae | *Scorpaenodes guamensis* | French Polynesia:Society Islands:Moorea | MNHN:Ich:BIOCODES0236 | MBIO345.4 | JQ432126 |
| Scorpaenidae | *Scorpaenodes guamensis* | French Polynesia:Society Islands:Moorea | MNHN:Ich:BIOCODES0238 | MBIO348.4 | JQ432125 |
| Scorpaenidae | *Scorpaenodes guamensis* | French Polynesia:Society Islands:Moorea | MNHN:Ich:BIOCODES491 | MBIO726.4 | JQ432124 |
| Scorpaenidae | *Scorpaenodes guamensis* | French Polynesia:Society Islands:Moorea | MNHN:Ich:BIOCODES492 | MBIO727.4 | JQ432123 |
| Scorpaenidae | *Scorpaenodes kelloggi* | Reunion:West:St Leu | ECOMAR:Ich:REU2613 | REU2613 | JQ350350 |
| Scorpaenidae | *Scorpaenodes minor* | French Polynesia:Society Islands:Moorea | MNHN:Ich:BIOCODES839 | MBIO1318.4 | JQ432128 |
| Scorpaenidae | *Scorpaenodes minor* | French Polynesia:Society Islands:Moorea | SI:Ich:MBIO1319 | MBIO1319.4 | JQ432129 |
| Scorpaenidae | *Scorpaenodes minor* | French Polynesia:Society Islands:Moorea | MNHN:Ich:MBIO1641 | MBIO1641.4 | JQ432127 |
| Scorpaenidae | *Scorpaenodes parvipinnis* | Madagascar:West:Nosy Be | ECOMAR:Ich:NBE0080 | NBE0080 | JQ350351 |
| Scorpaenidae | *Scorpaenodes parvipinnis* | Madagascar:West:Nosy Be | ECOMAR:Ich:NBE0081 | NBE0081 | JQ350352 |
| Scorpaenidae | *Scorpaenodes* sp*.* | French Polynesia:Society Islands:Moorea | MNHN:Ich:BIOCODES491 | MBIO730.4 | JQ432130 |
| Scorpaenidae | *Scorpaenodes* sp. | French Polynesia:Society Islands:Moorea | MNHN:Ich:BIOCODES490 | MBIO731.4 | JQ432131 |
| Scorpaenidae | *Scorpaenopsis diabolus* | French Polynesia:Society Islands:Moorea | MNHN:Ich:BIOCODES680 | MBIO1003.4 | JQ432132 |
| Scorpaenidae | *Scorpaenopsis diabolus* | French Polynesia:Society Islands:Moorea | SI:Ich:MBIO1004 | MBIO1004.4 | JQ432133 |
| Scorpaenidae | *Scorpaenopsis diabolus* | French Polynesia:Society Islands:Moorea | SI:Ich:MBIO1546 | MBIO1546.4 | JQ432134 |
| Scorpaenidae | *Scorpaenopsis diabolus* | Reunion:West:St Leu | ECOMAR:Ich:REU1031 | REU1031 | JQ350355 |
| Scorpaenidae | *Scorpaenopsis diabolus* | Reunion:West:St Leu | ECOMAR:Ich:REU1032 | REU1032 | JQ350354 |
| Scorpaenidae | *Scorpaenopsis diabolus* | Reunion:West:La Saline | ECOMAR:Ich:REU2753 | REU2753 | JQ350353 |
| Scorpaenidae | *Scorpaenopsis gibbosa* | Madagascar:West:Nosy Be | ECOMAR:Ich:NBE0082 | NBE0082 | JQ350358 |
| Scorpaenidae | *Scorpaenopsis gibbosa* | Madagascar:West:Nosy Be | ECOMAR:Ich:NBE0389 | NBE0389 | JQ350357 |
| Scorpaenidae | *Scorpaenopsis gibbosa* | Madagascar:West:Nosy Be | ECOMAR:Ich:NBE1246 | NBE1246 | JQ350356 |
| Scorpaenidae | *Scorpaenopsis gibbosa* | Madagascar:West:Nosy Be | ECOMAR:Ich:NBE1248 | NBE1248 | JQ350359 |
| Scorpaenidae | *Scorpaenopsis longispina* | Madagascar:West:Nosy Be | ECOMAR:Ich:NBE0218 | NBE0218 | JQ350361 |
| Scorpaenidae | *Scorpaenopsis longispina* | Madagascar:West:Nosy Be | ECOMAR:Ich:NBE0219 | NBE0219 | JQ350360 |
| Scorpaenidae | *Scorpaenopsis macrochir* | French Polynesia:Society Islands:Moorea | MNHN:Ich:BIOCODES777 | MBIO1183.4 | JQ432135 |
| Scorpaenidae | *Scorpaenopsis macrochir* | French Polynesia:Society Islands:Moorea | SI:Ich:MBIO1184 | MBIO1184.4 | JQ432136 |
| Scorpaenidae | *Scorpaenopsis possi* | French Polynesia:Society Islands:Moorea | MNHN:Ich:BIOCODES810 | MBIO1270.4 | JQ432139 |
| Scorpaenidae | *Scorpaenopsis possi* | French Polynesia:Society Islands:Moorea | MNHN:Ich:BIOCODES0920 | MBIO1547.4 | JQ432138 |
| Scorpaenidae | *Scorpaenopsis possi* | French Polynesia:Society Islands:Moorea | MNHN:Ich:BIOCODES0423 | MBIO604.4 | JQ432137 |
| Scorpaenidae | *Sebastapistes fowleri* | French Polynesia:Society Islands:Moorea | MNHN:Ich:BIOCODES699 | MBIO1041.4 | JQ432142 |
| Scorpaenidae | *Sebastapistes fowleri* | French Polynesia:Society Islands:Moorea | MNHN:Ich:BIOCODES705 | MBIO1045.4 | JQ432143 |
| Scorpaenidae | *Sebastapistes fowleri* | French Polynesia:Society Islands:Moorea | MNHN:Ich:BIOCODES0077 | MBIO124.4 | JQ432145 |
| Scorpaenidae | *Sebastapistes fowleri* | French Polynesia:Society Islands:Moorea | MNHN:Ich:BIOCODES844 | MBIO1331.4 | JQ432146 |
| Scorpaenidae | *Sebastapistes fowleri* | French Polynesia:Society Islands:Moorea | SI:Ich:MBIO1332 | MBIO1332.4 | JQ432144 |
| Scorpaenidae | *Sebastapistes fowleri* | French Polynesia:Society Islands:Moorea | MNHN:Ich:BIOCODES0052 | MBIO91.4 | JQ432141 |
| Scorpaenidae | *Sebastapistes fowleri* | French Polynesia:Society Islands:Moorea | MNHN:Ich:BIOCODES0053 | MBIO92.4 | JQ432140 |
| Scorpaenidae | *Sebastapistes tinkhami* | French Polynesia:Society Islands:Moorea | MNHN:Ich:BIOCODES0209 | MBIO303.4 | JQ432147 |
| Scorpaenidae | *Sebastapistes tinkhami* | French Polynesia:Society Islands:Moorea | MNHN:Ich:BIOCODES0210 | MBIO304.4 | JQ432148 |
| Scorpaenidae | *Sebastapistes tinkhami* | Reunion:West:St Leu | ECOMAR:Ich:REU0954 | REU0954 | JQ350363 |
| Scorpaenidae | *Sebastapistes tinkhami* | Reunion:West:St Leu | ECOMAR:Ich:REU0955 | REU0955 | JQ350362 |
| Serranidae | *Aethaloperca rogaa* | Madagascar:West:Nosy Be | ECOMAR:Ich:NBE0341 | NBE0341 | JQ349677 |
| Serranidae | *Aporops bilinearis* | French Polynesia:Society Islands:Moorea | MNHN:Ich:BIOCODES0157 | MBIO226.4 | JQ431457 |
| Serranidae | *Belonoperca chabanaudi* | French Polynesia:Society Islands:Moorea | MNHN:Ich:BIOCODES1038 | MBIO1850.4 | JQ431484 |
| Serranidae | *Cephalopholis argus* | French Polynesia:Society Islands:Moorea | MNHN:Ich:BIOCODES0078 | MBIO125.4 | JQ431565 |
| Serranidae | *Cephalopholis argus* | French Polynesia:Society Islands:Moorea | MNHN:Ich:BIOCODES0222 | MBIO327.4 | JQ431569 |
| Serranidae | *Cephalopholis argus* | French Polynesia:Society Islands:Moorea | MNHN:Ich:BIOCODES0223 | MBIO328.4 | JQ431566 |
| Serranidae | *Cephalopholis argus* | French Polynesia:Society Islands:Moorea | MNHN:Ich:BIOCODES0325 | MBIO475.4 | JQ431567 |
| Serranidae | *Cephalopholis argus* | French Polynesia:Society Islands:Moorea | MNHN:Ich:BIOCODES588 | MBIO868.4 | JQ431564 |
| Serranidae | *Cephalopholis argus* | French Polynesia:Society Islands:Moorea | SI:Ich:MBIO869 | MBIO869.4 | JQ431568 |
| Serranidae | *Cephalopholis argus* | Madagascar:West:Nosy Be | ECOMAR:Ich:NBE0010 | NBE0010 | JQ349856 |
| Serranidae | *Cephalopholis argus* | Madagascar:West:Nosy Be | ECOMAR:Ich:NBE1253 | NBE1253 | JQ349857 |
| Serranidae | *Cephalopholis boenak* | Madagascar:West:Nosy Be | ECOMAR:Ich:NBE0021 | NBE0021 | JQ349861 |
| Serranidae | *Cephalopholis boenak* | Madagascar:West:Nosy Be | ECOMAR:Ich:NBE0084 | NBE0084 | JQ349860 |
| Serranidae | *Cephalopholis boenak* | Madagascar:West:Nosy Be | ECOMAR:Ich:NBE0085 | NBE0085 | JQ349859 |
| Serranidae | *Cephalopholis boenak* | Madagascar:West:Nosy Be | ECOMAR:Ich:NBE0086 | NBE0086 | JQ349858 |
| Serranidae | *Cephalopholis leopardus* | French Polynesia:Society Islands:Moorea | MNHN:Ich:BIOCODES0038 | MBIO68.4 | JQ431571 |
| Serranidae | *Cephalopholis leopardus* | French Polynesia:Society Islands:Moorea | MNHN:Ich:BIOCODES0039 | MBIO69.4 | JQ431570 |
| Serranidae | *Cephalopholis miniata* | Madagascar:West:Nosy Be | ECOMAR:Ich:NBE0342 | NBE0342 | JQ349866 |
| Serranidae | *Cephalopholis miniata* | Madagascar:West:Nosy Be | ECOMAR:Ich:NBE1120 | NBE1120 | JQ349864 |
| Serranidae | *Cephalopholis miniata* | Madagascar:West:Nosy Be | ECOMAR:Ich:NBE1121 | NBE1121 | JQ349862 |
| Serranidae | *Cephalopholis miniata* | Madagascar:West:Nosy Be | ECOMAR:Ich:NBE1250 | NBE1250 | JQ349865 |
| Serranidae | *Cephalopholis miniata* | Madagascar:West:Nosy Be | ECOMAR:Ich:NBE1251 | NBE1251 | JQ349863 |
| Serranidae | *Cephalopholis sexmaculata* | French Polynesia:Society Islands:Moorea | MNHN:Ich:BIOCODES0460 | MBIO667.4 | JQ431573 |
| Serranidae | *Cephalopholis sexmaculata* | French Polynesia:Society Islands:Moorea | MNHN:Ich:BIOCODES614 | MBIO905.4 | JQ431572 |
| Serranidae | *Cephalopholis sonnerati* | French Polynesia:Society Islands:Moorea | MNHN:Ich:BIOCODES1051 | MBIO1868.4 | JQ431574 |
| Serranidae | *Cephalopholis sonnerati* | French Polynesia:Society Islands:Moorea | MNHN:Ich:BIOCODES1052 | MBIO1869.4 | JQ431575 |
| Serranidae | *Cephalopholis spiloparaea* | French Polynesia:Society Islands:Moorea | MNHN:Ich:BIOCODES606 | MBIO900.4 | JQ431576 |
| Serranidae | *Cephalopholis spiloparaea* | Reunion:West:St Leu | ECOMAR:Ich:REU0959 | REU0959 | JQ349868 |
| Serranidae | *Cephalopholis spiloparaea* | Reunion:West:St Leu | ECOMAR:Ich:REU0960 | REU0960 | JQ349867 |
| Serranidae | *Cephalopholis urodeta* | French Polynesia:Society Islands:Moorea | MNHN:Ich:BIOCODES0035 | MBIO64.4 | JQ431578 |
| Serranidae | *Cephalopholis urodeta* | French Polynesia:Society Islands:Moorea | MNHN:Ich:BIOCODES0036 | MBIO65.4 | JQ431577 |
| Serranidae | *Cephalopholis urodeta* | Reunion:West:St Gilles | ECOMAR:Ich:REU0783 | REU0783 | JQ349872 |
| Serranidae | *Cephalopholis urodeta* | Reunion:West:St Leu | ECOMAR:Ich:REU0949 | REU0949 | JQ349871 |
| Serranidae | *Cephalopholis urodeta* | Reunion:West:St Leu | ECOMAR:Ich:REU0950 | REU0950 | JQ349869 |
| Serranidae | *Cephalopholis urodeta* | Reunion:West:St Leu | ECOMAR:Ich:REU0951 | REU0951 | JQ349870 |
| Serranidae | *Cephalopholis urodeta* | Reunion:West:St Leu | ECOMAR:Ich:REU1620 | REU1620 | JQ349873 |
| Serranidae | *Epinephelus coeruleopunctatus* | Madagascar:West:Nosy Be | ECOMAR:Ich:NBE0252 | NBE0252 | JQ349961 |
| Serranidae | *Epinephelus coeruleopunctatus* | Madagascar:West:Nosy Be | ECOMAR:Ich:NBE0339 | NBE0339 | JQ349962 |
| Serranidae | *Epinephelus fasciatus* | French Polynesia:Society Islands:Moorea | MNHN:Ich:BIOCODES0116 | MBIO172.4 | JQ431717 |
| Serranidae | *Epinephelus flavocaeruleus* | Madagascar:West:Nosy Be | ECOMAR:Ich:NBE1264 | NBE1264 | JQ349963 |
| Serranidae | *Epinephelus hexagonatus* | French Polynesia:Society Islands:Moorea | MNHN:Ich:BIOCODES0186 | MBIO267.4 | JQ431718 |
| Serranidae | *Epinephelus hexagonatus* | French Polynesia:Society Islands:Moorea | MNHN:Ich:BIOCODES0187 | MBIO268.4 | JQ431719 |
| Serranidae | *Epinephelus hexagonatus* | French Polynesia:Society Islands:Moorea | MNHN:Ich:BIOCODES589 | MBIO874.4 | JQ431720 |
| Serranidae | *Epinephelus hexagonatus* | Reunion:South:St Philippe | ECOMAR:Ich:REU1754 | REU1754 | JQ349965 |
| Serranidae | *Epinephelus hexagonatus* | Reunion:South:St Philippe | ECOMAR:Ich:REU1867 | REU1867 | JQ349964 |
| Serranidae | *Epinephelus melanostigma* | Madagascar:West:Nosy Be | ECOMAR:Ich:NBE0343 | NBE0343 | JQ349966 |
| Serranidae | *Epinephelus merra* | French Polynesia:Society Islands:Moorea | SI:Ich:MBIO1199 | MBIO1199.4 | JQ431722 |
| Serranidae | *Epinephelus merra* | French Polynesia:Society Islands:Moorea | MNHN:Ich:BIOCODES0225 | MBIO323.4 | JQ431723 |
| Serranidae | *Epinephelus merra* | French Polynesia:Society Islands:Moorea | MNHN:Ich:BIOCODES0226 | MBIO324.4 | JQ431721 |
| Serranidae | *Epinephelus merra* | Reunion:West:St Gilles | ECOMAR:Ich:REU106-1 | REU106_1 | JQ349969 |
| Serranidae | *Epinephelus merra* | Reunion:West:St Gilles | ECOMAR:Ich:REU150-1 | REU150_1 | JQ349968 |
| Serranidae | *Epinephelus merra* | Reunion:West:St Gilles | ECOMAR:Ich:REU1669 | REU1669 | JQ349967 |
| Serranidae | *Epinephelus retouti* | French Polynesia:Society Islands:Moorea | MNHN:Ich:BIOCODES1050 | MBIO1872.4 | JQ431724 |
| Serranidae | *Epinephelus tauvina* | French Polynesia:Society Islands:Moorea | SI:Ich:MBIO1373 | MBIO1373.4 | JQ431726 |
| Serranidae | *Epinephelus tauvina* | French Polynesia:Society Islands:Moorea | MNHN:Ich:BIOCODES0145 | MBIO212.4 | JQ431725 |
| Serranidae | *Epinephelus tauvina* | Madagascar:West:Nosy Be | ECOMAR:Ich:NBE0674 | NBE0674 | JQ349970 |
| Serranidae | *Grammistes sexlineatus* | French Polynesia:Society Islands:Moorea | MNHN:Ich:BIOCODES0983 | MBIO1671.4 | JQ431776 |
| Serranidae | *Grammistes sexlineatus* | Madagascar:West:Nosy Be | ECOMAR:Ich:NBE0384 | NBE0384 | JQ350014 |
| Serranidae | *Grammistes sexlineatus* | Reunion:South:St Philippe | ECOMAR:Ich:REU1808 | REU1808 | JQ350013 |
| Serranidae | *Grammistops ocellatus* | French Polynesia:Society Islands:Moorea | SI:Ich:MBIO1329 | MBIO1329.4 | JQ431778 |
| Serranidae | *Grammistops ocellatus* | French Polynesia:Society Islands:Moorea | MNHN:Ich:BIOCODES662 | MBIO975.4 | JQ431777 |
| Serranidae | *Liopropoma lunulatum* | French Polynesia:Society Islands:Moorea | MNHN:Ich:BIOCODES0887 | MBIO1472.4 | JQ431888 |
| Serranidae | *Liopropoma lunulatum* | French Polynesia:Society Islands:Moorea | SI:Ich:MBIO1710 | MBIO1710.4 | JQ431889 |
| Serranidae | *Liopropoma pallidum* | French Polynesia:Society Islands:Moorea | MNHN:Ich:BIOCODES655 | MBIO961.4 | JQ431890 |
| Serranidae | *Liopropoma pallidum* | French Polynesia:Society Islands:Moorea | MNHN:Ich:BIOCODES653 | MBIO962.4 | JQ431891 |
| Serranidae | *Nemanthias carberryi* | Madagascar:West:Nosy Be | ECOMAR:Ich:NBE1130 | NBE1130 | JQ350133 |
| Serranidae | *Odontanthias tapui* | French Polynesia:Society Islands:Moorea | MNHN:Ich:BIOCODES0886 | MBIO1469.4 | JQ431935 |
| Serranidae | *Odontanthias tapui* | French Polynesia:Society Islands:Moorea | MNHN:Ich:BIOCODES0884 | MBIO1470.4 | JQ431934 |
| Serranidae | *Plectranthias longimanus* | French Polynesia:Society Islands:Moorea | MNHN:Ich:BIOCODES703 | MBIO1048.4 | JQ432002 |
| Serranidae | *Plectranthias longimanus* | French Polynesia:Society Islands:Moorea | MNHN:Ich:BIOCODES704 | MBIO1049.4 | JQ432003 |
| Serranidae | *Plectranthias longimanus* | French Polynesia:Society Islands:Moorea | MNHN:Ich:BIOCODES0028 | MBIO56.4 | JQ432004 |
| Serranidae | *Plectranthias longimanus* | French Polynesia:Society Islands:Moorea | MNHN:Ich:BIOCODES0029 | MBIO57.4 | JQ432001 |
| Serranidae | *Plectropomus laevis* | Madagascar:West:Nosy Be | ECOMAR:Ich:NBE1263 | NBE1263 | JQ350229 |
| Serranidae | *Pogonoperca punctata* | French Polynesia:Society Islands:Moorea | MNHN:Ich:BIOCODES782 | MBIO1217.4 | JQ432023 |
| Serranidae | *Pseudanthias evansi* | Reunion:West:St Gilles | ECOMAR:Ich:REU0741 | REU0741 | JQ350255 |
| Serranidae | *Pseudanthias evansi* | Reunion:West:St Gilles | ECOMAR:Ich:REU0742 | REU0742 | JQ350254 |
| Serranidae | *Pseudanthias evansi* | Reunion:West:St Leu | ECOMAR:Ich:REU1621 | REU1621 | JQ350256 |
| Serranidae | *Pseudanthias mooreanus* | French Polynesia:Society Islands:Moorea | MNHN:Ich:BIOCODES0998 | MBIO1742.4 | JQ432050 |
| Serranidae | *Pseudanthias mooreanus* | French Polynesia:Society Islands:Moorea | MNHN:Ich:BIOCODES0468 | MBIO687.4 | JQ432051 |
| Serranidae | *Pseudanthias mooreanus* | French Polynesia:Society Islands:Moorea | MNHN:Ich:BIOCODES0470 | MBIO688.4 | JQ432052 |
| Serranidae | *Pseudanthias olivaceus* | French Polynesia:Society Islands:Moorea | MNHN:Ich:BIOCODES1036 | MBIO1846.4 | JQ432054 |
| Serranidae | *Pseudanthias olivaceus* | French Polynesia:Society Islands:Moorea | MNHN:Ich:BIOCODES656 | MBIO976.4 | JQ432053 |
| Serranidae | *Pseudanthias pascalus* | French Polynesia:Society Islands:Moorea | MNHN:Ich:BIOCODES701 | MBIO1035.4 | JQ432056 |
| Serranidae | *Pseudanthias pascalus* | French Polynesia:Society Islands:Moorea | MNHN:Ich:BIOCODES628 | MBIO929.4 | JQ432055 |
| Serranidae | *Pseudanthias squamipinnis* | Madagascar:West:Nosy Be | ECOMAR:Ich:NBE0407 | NBE0407 | JQ350262 |
| Serranidae | *Pseudanthias squamipinnis* | Madagascar:West:Nosy Be | ECOMAR:Ich:NBE0409 | NBE0409 | JQ350264 |
| Serranidae | *Pseudanthias squamipinnis* | Madagascar:West:Nosy Be | ECOMAR:Ich:NBE0410 | NBE0410 | JQ350263 |
| Serranidae | *Pseudanthias squamipinnis* | Madagascar:West:Nosy Be | ECOMAR:Ich:NBE1144 | NBE1144 | JQ350259 |
| Serranidae | *Pseudanthias squamipinnis* | Madagascar:West:Nosy Be | ECOMAR:Ich:NBE1145 | NBE1145 | JQ350258 |
| Serranidae | *Pseudanthias squamipinnis* | Madagascar:West:Nosy Be | ECOMAR:Ich:NBE1147 | NBE1147 | JQ350260 |
| Serranidae | *Pseudanthias squamipinnis* | Madagascar:West:Nosy Be | ECOMAR:Ich:NBE1240 | NBE1240 | JQ350257 |
| Serranidae | *Pseudanthias squamipinnis* | Madagascar:West:Nosy Be | ECOMAR:Ich:NBE1241 | NBE1241 | JQ350261 |
| Serranidae | *Pseudogramma polyacanthum* | French Polynesia:Society Islands:Moorea | MNHN:Ich:BIOCODES0012 | MBIO35.4 | JQ432064 |
| Serranidae | *Pseudogramma polyacanthum* | French Polynesia:Society Islands:Moorea | MNHN:Ich:BIOCODES0013 | MBIO36.4 | JQ432065 |
| Serranidae | *Pseudogramma polyacanthum* | French Polynesia:Society Islands:Moorea | MNHN:Ich:MBIO509 | MBIO509.4 | JQ432063 |
| Serranidae | *Pseudogramma polyacanthum* | Madagascar:West:Nosy Be | ECOMAR:Ich:NBE0418 | NBE0418 | JQ350283 |
| Serranidae | *Pseudogramma polyacanthum* | Madagascar:West:Nosy Be | ECOMAR:Ich:NBE0419 | NBE0419 | JQ350284 |
| Serranidae | *Pseudogramma polyacanthum* | Madagascar:West:Nosy Be | ECOMAR:Ich:NBE0421 | NBE0421 | JQ350282 |
| Serranidae | *Pseudogramma polyacanthum* | Reunion:West:St Leu | ECOMAR:Ich:REU1009 | REU1009 | JQ350285 |
| Serranidae | *Saloptia powelli* | French Polynesia:Society Islands:Moorea | MNHN:Ich:BIOCODES0879 | MBIO1460.4 | JQ432090 |
| Serranidae | *Suttonia lineata* | French Polynesia:Society Islands:Moorea | MNHN:Ich:BIOCODES0917 | MBIO1536.4 | JQ432178 |
| Serranidae | *Variola louti* | French Polynesia:Society Islands:Moorea | MNHN:Ich:MBIO1231 | MBIO1231.4 | JQ432216 |
| Serranidae | *Variola louti* | French Polynesia:Society Islands:Moorea | SI:Ich:MBIO1562 | MBIO1562.4 | JQ432217 |
| Serranidae | *Variola louti* | French Polynesia:Society Islands:Moorea | MNHN:Ich:BIOCODES0146 | MBIO213.4 | JQ432218 |
| Siganidae | *Siganus argenteus* | French Polynesia:Society Islands:Moorea | MNHN:Ich:BIOCODES822 | MBIO1291.4 | JQ432156 |
| Siganidae | *Siganus argenteus* | French Polynesia:Society Islands:Moorea | SI:Ich:MBIO1851 | MBIO1851.4 | JQ432157 |
| Siganidae | *Siganus argenteus* | Reunion:West:St Gilles | ECOMAR:Ich:REU0505 | REU0505 | JQ350365 |
| Siganidae | *Siganus argenteus* | Reunion:West:St Gilles | ECOMAR:Ich:REU0725 | REU0725 | JQ350364 |
| Siganidae | *Siganus luridus* | Madagascar:West:Nosy Be | ECOMAR:Ich:NBE0018 | NBE0018 | JQ350366 |
| Siganidae | *Siganus spinus* | French Polynesia:Society Islands:Moorea | MNHN:Ich:BIOCODES618 | MBIO913.4 | JQ432158 |
| Siganidae | *Siganus spinus* | French Polynesia:Society Islands:Moorea | MNHN:Ich:BIOCODES617 | MBIO914.4 | JQ432159 |
| Siganidae | *Siganus stellatus* | Madagascar:West:Nosy Be | ECOMAR:Ich:NBE0667 | NBE0667 | JQ350367 |
| Siganidae | *Siganus sutor* | Madagascar:West:Nosy Be | ECOMAR:Ich:NBE0665 | NBE0665 | JQ350368 |
| Siganidae | *Siganus sutor* | Madagascar:West:Nosy Be | ECOMAR:Ich:NBE0666 | NBE0666 | JQ350369 |
| Soleidae | *Aseraggodes melanostictus* | French Polynesia:Society Islands:Moorea | MNHN:Ich:BIOCODES679 | MBIO998.4 | JQ431466 |
| Soleidae | *Aseraggodes melanostictus* | French Polynesia:Society Islands:Moorea | SI:Ich:MBIO999 | MBIO999.4 | JQ431467 |
| Soleidae | *Aseraggodes* sp. 1 | French Polynesia:Society Islands:Moorea | MNHN:Ich:BIOCODES0154 | MBIO222.4 | JQ431468 |
| Soleidae | *Aseraggodes* sp. 2 | French Polynesia:Society Islands:Moorea | MNHN:Ich:BIOCODES0863 | MBIO1419.4 | JQ431469 |
| Soleidae | *Pardachirus marmoratus* | Madagascar:West:Nosy Be | ECOMAR:Ich:NBE1291 | NBE1291 | JQ350177 |
| Soleidae | *Pardachirus morrowi* | Madagascar:West:Nosy Be | ECOMAR:Ich:NBE1039 | NBE1039 | JQ350178 |
| Synanceiidae | *Synanceia verrucosa* | French Polynesia:Society Islands:Moorea | MNHN:Ich:BIOCODES566 | MBIO833.4 | JQ432179 |
| Syngnathidae | *Choeroichthys brachysoma* | French Polynesia:Society Islands:Moorea | MNHN:Ich:BIOCODES0234 | MBIO343.4 | JQ431627 |
| Syngnathidae | *Choeroichthys brachysoma* | French Polynesia:Society Islands:Moorea | MNHN:Ich:BIOCODES0375 | MBIO539.4 | JQ431628 |
| Syngnathidae | *Corythoichthys amplexus* | Madagascar:West:Nosy Be | ECOMAR:Ich:NBE1157 | NBE1157 | JQ349916 |
| Syngnathidae | *Corythoichthys flavofasciatus* | French Polynesia:Society Islands:Moorea | SI:Ich:MBIO1762 | MBIO1762.4 | JQ431666 |
| Syngnathidae | *Corythoichthys flavofasciatus* | French Polynesia:Society Islands:Moorea | MNHN:Ich:BIOCODES485 | MBIO716.4 | JQ431667 |
| Syngnathidae | *Corythoichthys flavofasciatus* | French Polynesia:Society Islands:Moorea | MNHN:Ich:BIOCODES486 | MBIO717.4 | JQ431668 |
| Syngnathidae | *Corythoichthys flavofasciatus* | Madagascar:West:Nosy Be | ECOMAR:Ich:NBE1044 | NBE1044 | JQ349917 |
| Syngnathidae | *Corythoichthys flavofasciatus* | Madagascar:West:Nosy Be | ECOMAR:Ich:NBE1045 | NBE1045 | JQ349918 |
| Syngnathidae | *Doryrhamphus excisus* | French Polynesia:Society Islands:Moorea | MNHN:Ich:BIOCODES0232 | MBIO340.4 | JQ431692 |
| Syngnathidae | *Doryrhamphus excisus* | French Polynesia:Society Islands:Moorea | MNHN:Ich:BIOCODES0233 | MBIO341.4 | JQ431689 |
| Syngnathidae | *Doryrhamphus excisus* | French Polynesia:Society Islands:Moorea | MNHN:Ich:BIOCODES500 | MBIO734.4 | JQ431691 |
| Syngnathidae | *Doryrhamphus excisus* | French Polynesia:Society Islands:Moorea | MNHN:Ich:BIOCODES497 | MBIO735.4 | JQ431690 |
| Syngnathidae | *Hippichthys cyanospilus* | Madagascar:West:Nosy Be | ECOMAR:Ich:NBE1156 | NBE1156 | JQ350058 |
| Synodontidae | *Saurida gracilis* | French Polynesia:Society Islands:Moorea | MNHN:Ich:BIOCODES481 | MBIO708.4 | JQ432093 |
| Synodontidae | *Saurida gracilis* | French Polynesia:Society Islands:Moorea | MNHN:Ich:BIOCODES479 | MBIO709.4 | JQ432094 |
| Synodontidae | *Saurida nebulosa* | Reunion:West:St Gilles | ECOMAR:Ich:REU1622 | REU1622 | JQ350326 |
| Synodontidae | *Synodus binotatus* | Madagascar:West:Nosy Be | ECOMAR:Ich:NBE0121 | NBE0121 | JQ350383 |
| Synodontidae | *Synodus binotatus* | Madagascar:West:Nosy Be | ECOMAR:Ich:NBE0433 | NBE0433 | JQ350385 |
| Synodontidae | *Synodus binotatus* | Madagascar:West:Nosy Be | ECOMAR:Ich:NBE0609 | NBE0609 | JQ350387 |
| Synodontidae | *Synodus binotatus* | Madagascar:West:Nosy Be | ECOMAR:Ich:NBE0610 | NBE0610 | JQ350386 |
| Synodontidae | *Synodus binotatus* | Madagascar:West:Nosy Be | ECOMAR:Ich:NBE0611 | NBE0611 | JQ350388 |
| Synodontidae | *Synodus binotatus* | Madagascar:West:Nosy Be | ECOMAR:Ich:NBE1185 | NBE1185 | JQ350389 |
| Synodontidae | *Synodus binotatus* | Madagascar:West:Nosy Be | ECOMAR:Ich:NBE1186 | NBE1186 | JQ350384 |
| Synodontidae | *Synodus dermatogenys* | French Polynesia:Society Islands:Moorea | MNHN:Ich:BIOCODES0380 | MBIO542.4 | JQ432183 |
| Synodontidae | *Synodus dermatogenys* | French Polynesia:Society Islands:Moorea | MNHN:Ich:BIOCODES0376 | MBIO543.4 | JQ432181 |
| Synodontidae | *Synodus dermatogenys* | French Polynesia:Society Islands:Moorea | MNHN:Ich:BIOCODES586 | MBIO865.4 | JQ432182 |
| Synodontidae | *Synodus dermatogenys* | Reunion:West:St Gilles | ECOMAR:Ich:REU078-1 | REU078_1 | JQ350390 |
| Synodontidae | *Synodus jaculum* | French Polynesia:Society Islands:Moorea | MNHN:Ich:BIOCODES734 | MBIO1117.4 | JQ432184 |
| Synodontidae | *Synodus jaculum* | French Polynesia:Society Islands:Moorea | SI:Ich:MBIO1118 | MBIO1118.4 | JQ432185 |
| Synodontidae | *Synodus jaculum* | Madagascar:West:Nosy Be | ECOMAR:Ich:NBE1187 | NBE1187 | JQ350391 |
| Synodontidae | *Synodus jaculum* | Madagascar:West:Nosy Be | ECOMAR:Ich:NBE1188 | NBE1188 | JQ350392 |
| Synodontidae | *Synodus variegatus* | French Polynesia:Society Islands:Moorea | MNHN:Ich:BIOCODES0040 | MBIO71.4 | JQ432189 |
| Synodontidae | *Synodus variegatus* | French Polynesia:Society Islands:Moorea | MNHN:Ich:BIOCODES0034 | MBIO72.4 | JQ432188 |
| Synodontidae | *Synodus variegatus* | French Polynesia:Society Islands:Moorea | MNHN:Ich:BIOCODES487 | MBIO721.4 | JQ432187 |
| Synodontidae | *Synodus variegatus* | French Polynesia:Society Islands:Moorea | SI:Ich:MBIO722 | MBIO722.4 | JQ432186 |
| Tetraodontidae | *Arothron hispidus* | French Polynesia:Society Islands:Moorea | MNHN:Ich:BIOCODES795 | MBIO1236.4 | JQ431462 |
| Tetraodontidae | *Arothron hispidus* | Madagascar:West:Nosy Be | ECOMAR:Ich:NBE0677 | NBE0677 | JQ349778 |
| Tetraodontidae | *Arothron mappa* | Madagascar:West:Nosy Be | ECOMAR:Ich:NBE1313 | NBE1313 | JQ349779 |
| Tetraodontidae | *Arothron meleagris* | French Polynesia:Society Islands:Moorea | MNHN:Ich:BIOCODES814 | MBIO1280.4 | JQ431465 |
| Tetraodontidae | *Arothron meleagris* | French Polynesia:Society Islands:Moorea | SI:Ich:MBIO1281 | MBIO1281.4 | JQ431463 |
| Tetraodontidae | *Arothron meleagris* | French Polynesia:Society Islands:Moorea | MNHN:Ich:BIOCODES637 | MBIO931.4 | JQ431464 |
| Tetraodontidae | *Arothron meleagris* | Madagascar:West:Nosy Be | ECOMAR:Ich:NBE0678 | NBE0678 | JQ349780 |
| Tetraodontidae | *Arothron meleagris* | Reunion:West:St Leu | ECOMAR:Ich:REU0907 | REU0907 | JQ349781 |
| Tetraodontidae | *Canthigaster amboinensis* | French Polynesia:Society Islands:Moorea | MNHN:Ich:BIOCODES0191 | MBIO277.4 | JQ431526 |
| Tetraodontidae | *Canthigaster amboinensis* | French Polynesia:Society Islands:Moorea | MNHN:Ich:BIOCODES0192 | MBIO278.4 | JQ431525 |
| Tetraodontidae | *Canthigaster amboinensis* | Reunion:South:St Philippe | ECOMAR:Ich:REU1752 | REU1752 | JQ349823 |
| Tetraodontidae | *Canthigaster bennetti* | French Polynesia:Society Islands:Moorea | MNHN:Ich:BIOCODES0324 | MBIO471.4 | JQ431527 |
| Tetraodontidae | *Canthigaster bennetti* | Madagascar:West:Nosy Be | ECOMAR:Ich:NBE0371 | NBE0371 | JQ349824 |
| Tetraodontidae | *Canthigaster janthinoptera* | French Polynesia:Society Islands:Moorea | MNHN:Ich:BIOCODES0321 | MBIO467.4 | JQ431529 |
| Tetraodontidae | *Canthigaster janthinoptera* | French Polynesia:Society Islands:Moorea | SI:Ich:MBIO715 | MBIO715.4 | JQ431528 |
| Tetraodontidae | *Canthigaster janthinoptera* | Madagascar:West:Nosy Be | ECOMAR:Ich:NBE1142 | NBE1142 | JQ349826 |
| Tetraodontidae | *Canthigaster janthinoptera* | Madagascar:West:Nosy Be | ECOMAR:Ich:NBE1143 | NBE1143 | JQ349825 |
| Tetraodontidae | *Canthigaster janthinoptera* | Madagascar:West:Nosy Be | ECOMAR:Ich:NBE1232 | NBE1232 | JQ349827 |
| Tetraodontidae | *Canthigaster natalensis* | Reunion:North:La possession | ECOMAR:Ich:REU0147 | REU0147 | JQ349828 |
| Tetraodontidae | *Canthigaster smithae* | Reunion:West:St Gilles | ECOMAR:Ich:REU0780 | REU0780 | JQ349830 |
| Tetraodontidae | *Canthigaster smithae* | Reunion:West:St Leu | ECOMAR:Ich:REU0908 | REU0908 | JQ349829 |
| Tetraodontidae | *Canthigaster solandri* | French Polynesia:Society Islands:Moorea | MNHN:Ich:BIOCODES543 | MBIO804.4 | JQ431530 |
| Tetraodontidae | *Canthigaster solandri* | French Polynesia:Society Islands:Moorea | MNHN:Ich:BIOCODES638 | MBIO949.4 | JQ431531 |
| Tetraodontidae | *Canthigaster solandri* | Madagascar:West:Nosy Be | ECOMAR:Ich:NBE0030 | NBE0030 | JQ349833 |
| Tetraodontidae | *Canthigaster solandri* | Madagascar:West:Nosy Be | ECOMAR:Ich:NBE0031 | NBE0031 | JQ349834 |
| Tetraodontidae | *Canthigaster solandri* | Madagascar:West:Nosy Be | ECOMAR:Ich:NBE0299 | NBE0299 | JQ349831 |
| Tetraodontidae | *Canthigaster solandri* | Reunion:West:St Gilles | ECOMAR:Ich:REU304-1 | REU304_1 | JQ349832 |
| Tetraodontidae | *Canthigaster valentini* | French Polynesia:Society Islands:Moorea | SI:Ich:MBIO1218 | MBIO1218.4 | JQ431532 |
| Tetraodontidae | *Canthigaster valentini* | French Polynesia:Society Islands:Moorea | MNHN:Ich:BIOCODES0464 | MBIO674.4 | JQ431533 |
| Tetraodontidae | *Canthigaster valentini* | Madagascar:West:Nosy Be | ECOMAR:Ich:NBE0108 | NBE0108 | JQ349836 |
| Tetraodontidae | *Canthigaster valentini* | Madagascar:West:Nosy Be | ECOMAR:Ich:NBE0109 | NBE0109 | JQ349838 |
| Tetraodontidae | *Canthigaster valentini* | Reunion:West:St Gilles | ECOMAR:Ich:REU0779 | REU0779 | JQ349837 |
| Tetraodontidae | *Canthigaster valentini* | Reunion:West:St Gilles | ECOMAR:Ich:REU114-1 | REU114_1 | JQ349835 |
| Torpedinidae | *Torpedo fuscomaculata* | Madagascar:West:Nosy Be | ECOMAR:Ich:NBE0540 | NBE0540 | JQ350399 |
| Tripterygiidae | *Enneapterygius hemimelas* | French Polynesia:Society Islands:Moorea | MNHN:Ich:BIOCODES0163 | MBIO234.4 | JQ431709 |
| Tripterygiidae | *Enneapterygius hemimelas* | French Polynesia:Society Islands:Moorea | MNHN:Ich:BIOCODES0164 | MBIO235.4 | JQ431708 |
| Tripterygiidae | *Enneapterygius pyramis* | French Polynesia:Society Islands:Moorea | MNHN:Ich:BIOCODES0158 | MBIO231.4 | JQ431712 |
| Tripterygiidae | *Enneapterygius pyramis* | French Polynesia:Society Islands:Moorea | MNHN:Ich:BIOCODES0159 | MBIO232.4 | JQ431711 |
| Tripterygiidae | *Enneapterygius pyramis* | French Polynesia:Society Islands:Moorea | MNHN:Ich:BIOCODES0268 | MBIO394.4 | JQ431710 |
| Tripterygiidae | *Enneapterygius* sp. | Madagascar:West:Nosy Be | ECOMAR:Ich:NBE0294 | NBE0294 | JQ349952 |
| Tripterygiidae | *Enneapterygius* sp. | Madagascar:West:Nosy Be | ECOMAR:Ich:NBE0295 | NBE0295 | JQ349953 |
| Tripterygiidae | *Enneapterygius* sp. | Madagascar:West:Nosy Be | ECOMAR:Ich:NBE1038 | NBE1038 | JQ349954 |
| Tripterygiidae | *Enneapterygius tutuilae* | Madagascar:West:Nosy Be | ECOMAR:Ich:NBE0289 | NBE0289 | JQ349955 |
| Zanclidae | *Zanclus cornutus* | French Polynesia:Society Islands:Moorea | MNHN:Ich:BIOCODES482 | MBIO712.4 | JQ432221 |
| Zanclidae | *Zanclus cornutus* | French Polynesia:Society Islands:Moorea | SI:Ich:MBIO803 | MBIO803.4 | JQ432222 |
| Zanclidae | *Zanclus cornutus* | Madagascar:West:Nosy Be | ECOMAR:Ich:NBE1077 | NBE1077 | JQ350419 |
| Zanclidae | *Zanclus cornutus* | Reunion:West:St Gilles | ECOMAR:Ich:REU010-1 | REU010_1 | JQ350421 |
| Zanclidae | *Zanclus cornutus* | Reunion:West:St Gilles | ECOMAR:Ich:REU0764 | REU0764 | JQ350417 |
| Zanclidae | *Zanclus cornutus* | Reunion:West:St Gilles | ECOMAR:Ich:REU115-1 | REU115_1 | JQ350420 |
| Zanclidae | *Zanclus cornutus* | Reunion:West:St Gilles | ECOMAR:Ich:REU115-2 | REU115_2 | JQ350418 |
