# Supplementary material for: Cryptic Diversity in Indo-Pacific Coral-Reef Fishes Revealed by DNA-Barcoding Provides New Support to the Centre-of-Overlap Hypothesis
Source: PLoS One. 2012 Mar 15;7(3):e28987. doi: 10.1371/journal.pone.0028987 (PMC3305298; doi:10.1371/journal.pone.0028987)
Supplement: Table S2 — Summary of K2P distances to the nearest-neighbour for the 668 species. (DOCX) [file pone.0028987.s002.docx]

**Table S2.** Summary of K2P distances to the nearest-neighbour for the 668 species.

|  |  | Number of species | | | | |
| --- | --- | --- | --- | --- | --- | --- |
| Order | Family | barcoded | < 0.1 | 0.1 - 1.0 | 1.0 - 2.7 | > 2.7 |
| Perciformes | Labridae | 67 | 0 | 8 | 0 | 59 |
|  | Acanthuridae | 29 | 0 | 0 | 2 | 27 |
|  | Apogonidae | 37 | 0 | 0 | 0 | 37 |
|  | Chaetodontidae | 34 | 0 | 6 | 6 | 22 |
|  | Carangidae | 13 | 0 | 0 | 2 | 11 |
|  | Pseudochromidae | 4 | 0 | 0 | 0 | 4 |
|  | Scaridae | 15 | 0 | 2 | 0 | 13 |
|  | Serranidae | 37 | 0 | 0 | 0 | 37 |
|  | Gobiidae | 56 | 0 | 0 | 2 | 54 |
|  | Pinguipedidae | 2 | 0 | 0 | 0 | 2 |
|  | Microdesmidae | 6 | 0 | 0 | 0 | 6 |
|  | Pomacentridae | 60 | 0 | 2 | 4 | 54 |
|  | Pomacanthidae | 12 | 0 | 0 | 0 | 12 |
|  | Siganidae | 5 | 0 | 0 | 0 | 5 |
|  | Haemulidae | 4 | 0 | 0 | 0 | 4 |
|  | Blenniidae | 30 | 0 | 0 | 0 | 30 |
|  | Mullidae | 13 | 0 | 0 | 2 | 11 |
|  | Plesiopidae | 2 | 0 | 0 | 0 | 2 |
|  | Tripterygiidae | 4 | 0 | 0 | 0 | 4 |
|  | Caesionidae | 5 | 0 | 0 | 0 | 5 |
|  | Callionymidae | 3 | 0 | 0 | 0 | 3 |
|  | Cirrhitidae | 8 | 0 | 0 | 0 | 8 |
|  | Eleotridae | 2 | 0 | 0 | 0 | 2 |
|  | Lethrinidae | 7 | 0 | 0 | 0 | 7 |
|  | Kuhliidae | 4 | 0 | 0 | 0 | 4 |
|  | Lutjanidae | 12 | 0 | 0 | 0 | 12 |
|  | Creediidae | 2 | 0 | 0 | 0 | 2 |
|  | Pempheridae | 4 | 0 | 0 | 0 | 4 |
|  | Scombridae | 1 | 0 | 0 | 0 | 1 |
|  | Gerreidae | 1 | 0 | 0 | 0 | 1 |
|  | Polynemidae | 1 | 0 | 0 | 0 | 1 |
|  | Nemipteridae | 4 | 0 | 0 | 0 | 4 |
|  | Cichlidae | 1 | 0 | 0 | 0 | 1 |
|  | Kyphosidae | 2 | 0 | 0 | 2 | 0 |
|  | Zanclidae | 1 | 0 | 0 | 0 | 1 |
|  | Caproidae | 1 | 0 | 0 | 0 | 1 |
|  | Priacanthidae | 2 | 0 | 0 | 0 | 2 |
|  | Monodactylidae | 1 | 0 | 0 | 0 | 1 |
|  | Centropomidae | 1 | 0 | 0 | 0 | 1 |
|  | Malacanthidae | 1 | 0 | 0 | 0 | 1 |
| Scorpaeniformes | Scorpaenidae | 18 | 0 | 0 | 0 | 18 |
|  | Dactylopteridae | 1 | 0 | 0 | 0 | 1 |
|  | Caracanthidae | 3 | 0 | 0 | 0 | 3 |
|  | Platycephalidae | 3 | 0 | 0 | 0 | 3 |
|  | Synanceiidae | 1 | 0 | 0 | 0 | 1 |
| Anguilliformes | Congridae | 5 | 0 | 0 | 0 | 5 |
|  | Muraenidae | 33 | 0 | 0 | 0 | 33 |
|  | Ophichthidae | 10 | 0 | 0 | 0 | 10 |
|  | Anguillidae | 2 | 0 | 0 | 0 | 2 |
|  | Chlopsidae | 1 | 0 | 0 | 0 | 1 |
|  | Moringuidae | 3 | 0 | 0 | 0 | 3 |
| Tetraodontiformes | Ostraciidae | 2 | 0 | 0 | 0 | 2 |
|  | Monacanthidae | 8 | 0 | 0 | 0 | 8 |
|  | Balistidae | 13 | 0 | 0 | 0 | 13 |
|  | Tetraodontidae | 10 | 0 | 0 | 2 | 8 |
|  | Diodontidae | 1 | 0 | 0 | 0 | 1 |
| Beryciformes | Holocentridae | 19 | 0 | 3 | 5 | 11 |
| Syngnathiformes | Syngnathidae | 5 | 0 | 0 | 0 | 5 |
|  | Aulostomidae | 1 | 0 | 0 | 0 | 1 |
|  | Fistulariidae | 1 | 0 | 0 | 0 | 1 |
| Pleuronectiformes | Samaridae | 1 | 0 | 0 | 0 | 1 |
|  | Bothidae | 3 | 0 | 0 | 0 | 3 |
|  | Soleidae | 5 | 0 | 0 | 0 | 5 |
| Albuliformes | Albulidae | 1 | 0 | 0 | 0 | 1 |
| Ophidiiformes | Carapidae | 2 | 0 | 0 | 0 | 2 |
|  | Bythitidae | 2 | 0 | 0 | 0 | 2 |
|  | Ophidiidae | 1 | 0 | 0 | 0 | 1 |
| Mugiliformes | Mugilidae | 3 | 0 | 0 | 0 | 3 |
| Lophiiformes | Antennariidae | 1 | 0 | 0 | 0 | 1 |
| Beloniformes | Belonidae | 1 | 0 | 0 | 0 | 1 |
|  | Exocoetidae | 1 | 0 | 0 | 0 | 1 |
| Cyprinodontiformes | Poeciliidae | 1 | 0 | 0 | 0 | 1 |
| Clupeiformes | Clupeidae | 1 | 0 | 0 | 0 | 1 |
| Aulopiformes | Synodontidae | 6 | 0 | 0 | 0 | 6 |
| Carcharhiniformes | Carcharhinidae | 1 | 0 | 0 | 0 | 1 |
| Rajiformes | Dasyatidae | 1 | 0 | 0 | 0 | 1 |
| Torpediniformes | Torpedinidae | 1 | 0 | 0 | 0 | 1 |
| Gonorynchiformes | Chanidae | 1 | 0 | 0 | 0 | 1 |
| Siluriformes | Plotosidae | 1 | 0 | 0 | 0 | 1 |
|  | Total | 668 | 0 | 21 | 27 | 620 |
