# Supplementary material for: Cryptic Diversity in Indo-Pacific Coral-Reef Fishes Revealed by DNA-Barcoding Provides New Support to the Centre-of-Overlap Hypothesis
Source: PLoS One. 2012 Mar 15;7(3):e28987. doi: 10.1371/journal.pone.0028987 (PMC3305298; doi:10.1371/journal.pone.0028987)
Supplement: Table S3 — List of species with Indo-Pacific range distribution belonging to each of the four pattern of COI barcodes distribution. (DOCX) [file pone.0028987.s003.docx]

**Table S3.** List of the species from the present survey belonging to each of the four phylogenetic pattern.

| Pattern 1 | Pattern 2 | Pattern 3 | Pattern 4 |
| --- | --- | --- | --- |
| Shallow spatial structure | Geographic monophyly, deep divergence | Geographic paraphyly | Taxonomic paraphyly |
| *Abudefduf sordidus* (0.31^a^)^b^ | *Amblycirrhitus bimacula* (8.29) | *Dinematichthys iluocoeteoides* (15.61) | *Labroides dimidiatus* (14.98) |
| *Acanthurus guttatus* (0.35) | *Apogon angustatus* (3.47) | *Halichoeres hortulanus* (5.91) | *Myripristis berndti* (7.48) |
| *Acanthurus lineatus* (0.46) | *Apogon apogonides*  (6.85) | *Kaupichthys diodontus* (22.16) | *Plectroglyphidodon lacrymatus* (5.86) |
| *Acanthurus nigricauda* (0.62) | *Apogon exostigma* (3.32) | *Pseudogramma polyacantha* (6.62) | *Pterois antennata* (7.03) |
| *Acanthurus nigrofuscus* (1.09) | *Apogon fraenatus* (4.31) | *Synodus dermatogenys* (3.03) | *Sargocentron diadema* (4.64) |
| *Acanthurus triostegus* (0.93^a^) | *Aulostomus chinensis* (1.01) | *Sebastapistes tinkhami* (13.21) | *Zebrasoma scopas* (4.83) |
| *Anampses caeruleopunctatus* (0.62^a^) | *Apogon kallopterus* (3.76) |  |  |
| *Arothron meleagris* (0.15) | *Apogonichthys ocellatus* (6.31) |  |  |
| *Balistapus undulatus* (0.69) | *Asterropteryx ensifera* (2.85) |  |  |
| *Bodianus axillaris* (1.39) | *Asterropteryx semipunctata* (6.15) |  |  |
| *Bothus pantherinus* (0.47) | *Blenniella gibbifrons* (3.17) |  |  |
| *Calotomus carolinus* (0.36) | *Cantherines dumerilii* (1.59) |  |  |
| *Canthigaster amboinensis* (0.15^a^) | *Canthigaster solandri* (2.67) |  |  |
| *Canthigaster janthinoptera* (0.62) | *Caracanthus unipinna* (12.14) |  |  |
| *Canthigaster valentini* (0.91) | *Centropyge bispinosa* (1.15) |  |  |
| *Cephalopholis argus* (0.65) | *Cetoscarus bicolor* (2.52) |  |  |
| *Cephalopholis urodeta* (0.67) | *Chaetodon bennetti* (1.08) |  |  |
| *Chaetodon auriga* (0.31^a^) | *Chaetodon trifascialis* (0.92) |  |  |
| *Chaetodon lunula* (0.92) | *Cheilinus chlorourus* (2.82) |  |  |
| *Chaetodon unimaculatus* (0.61) | *Cheilinus trilobatus* (4.28) |  |  |
| *Chaetodon vagabundus* (0.77^a^) | *Cheilio inermis* (1.13) |  |  |
| *Cheilinus oxycephalus* (0.93) | *Cheilodipterus artus* (6.87) |  |  |
| *Chromis dimidiata* (0.77) | *Cheilodipterus quinquelineatus* (3.18) |  |  |
| *Chromis viridis* (2.87) | *Chlorurus sordidus* (1.72) |  |  |
| *Coris aygula* (0.92) | *Chromis atripectoralis* (2.13) |  |  |
| *Epibulus insidiator* (1.87^a^) | *Chrysiptera brownriggii* (3.82) |  |  |
| *Exallias brevis* (0.77) | *Conger cinereus* (9.45) |  |  |
| *Forcipiger flavissimus* (0.67^a^) | *Coryphopterus neophytus* (2.8) |  |  |
| *Gnathodentex aureolineatus* (0.67) | *Corythoichthys flavofasciatus* (4.29) |  |  |
| *Grammistes sexlineatus* (0.78) | *Ctenochaetus flavicauda* (1.2) |  |  |
| *Gymnothorax buroensis* (1.72) | *Ctenochaetus striatus* (2.28) |  |  |
| *Gymnothorax chilospilus* (1.4) | *Dascyllus aruanus* (0.93) |  |  |
| *Gymnothorax javanicus* (2.52^a^) | *Dascyllus trimaculatus* (2.36) |  |  |
| *Gymnothorax margaritophorus* (0.62^a^) | *Dendrochirus biocellatus* (1.11) |  |  |
| *Gymnothorax zonipectis* (2.17) | *Epinephelus hexagonatus* (1.24) |  |  |
| *Heniochus acuminatus* (0.46) | *Epinephelus merra* (3.86) |  |  |
| *Heniochus monoceros* (1.24^a^) | *Epinephelus tauvina* (1.55) |  |  |
| *Lutjanus gibbus* (0.16^a^) | *Fistularia commersonii* (1.93) |  |  |
| *Lutjanus kasmira* (0.46) | *Fowleria marmorata* (3.16) |  |  |
| *Mulloidichthys flavolineatus* (1.25) | *Gobiodon unicolor* (6.4) |  |  |
| *Myripristis pralinia* (0.98^a^) | *Gymnothorax undulatus* (3.99) |  |  |
| *Naso unicornis* (0.78^a^) | *Halichoeres marginatus* (5.06) |  |  |
| *Nemateleotris magnifica* (0.77) | *Hemigymnus fasciatus* (3.82) |  |  |
| *Ostracion cubicus* (1.55) | *Labroides bicolor* (1.13) |  |  |
| *Ostracion meleagris* (0.46^a^) | *Monotaxis grandoculis* (2.35) |  |  |
| *Paracirrhites arcatus* (0.72) | *Naso annulatus* (1.72) |  |  |
| *Paracirrhites forsteri* (1.16) | *Naso lituratus* (3.14) |  |  |
| *Plagiotremus tapeinosoma* (2.56) | *Neoniphon samara* (5.31) |  |  |
| *Plectroglyphidodon leucozonus* (0.52) | *Parupeneus barberinus* (7.52) |  |  |
| *Plectroglyphidodon imparipennis* (0.89) | *Parupeneus ciliatus* (5.48) |  |  |
| *Plectrypops lima* (1.09) | *Parupeneus cyclostomus* (1.47) |  |  |
| *Pterocaesio tile* (0.48) | *Parupeneus pleurostigma* (0.98) |  |  |
| *Rhinecanthus aculeatus* (0.31) | *Pervagor aspricaudus* (3.05) |  |  |
| *Rhinecanthus rectangulus* (0.31) | *Plectroglyphidodon johnstonianus* (1.91) |  |  |
| *Sargocentron punctatissimum* (1.87) | *Plectroglyphidodon phoenixensis* (2.38) |  |  |
| *Sargocentron spiniferum* (2.23) | *Pomacanthus imperator* (1.56) |  |  |
| *Sargocentron tiere* (1.4^a^) | *Pomacentrus pavo* (3.98) |  |  |
| *Sufflamen bursa* (1.2) | *Priolepis inhaca* (8.73) |  |  |
| *Thalassoma amblycephalum* (0.05^a^) | *Pseudocheilinus hexataenia* (11.75) |  |  |
| *Thalassoma hardwicke* (0.34^a^) | *Pseudocheilinus octotaenia* (3.48) |  |  |
| *Uropterygius fuscoguttatus* (1.08) | *Pygoplites diacanthus* (0.89) |  |  |
| *Zanclus cornutus* (1.01) | *Scarus niger* (2.68) |  |  |
|  | *Scorpaenopsis diabolus* (2.68) |  |  |
|  | *Siganus argenteus* (0.93) |  |  |
|  | *Stegastes nigricans* (4.45) |  |  |
|  | *Synodus jaculum* (1.09) |  |  |
|  | *Valenciennea strigata* (8.58) |  |  |

^a^ Haplotype sharing detected between Indian and Pacific oceans localities

^b^ maximum K2P distance
