# Supplementary material for: Cryptic Diversity in Indo-Pacific Coral-Reef Fishes Revealed by DNA-Barcoding Provides New Support to the Centre-of-Overlap Hypothesis
Source: PLoS One. 2012 Mar 15;7(3):e28987. doi: 10.1371/journal.pone.0028987 (PMC3305298; doi:10.1371/journal.pone.0028987)
Supplement: Figure S1 — Neighbour-joining tree of 2276 COI barcodes belonging to the 668 coral reef fish species examined here. (PDF) [file pone.0028987.s004.pdf]

BOLD TaxonID Tree

Project	:	MERGED: {MBFC,FPFL,IPCOM,MBFA,MBFB,SBF}
Subprojects	:	Acanthurids and Holocentrids[MBFC]
		Barcoding French Polynesia fish larvae[FPFL]
		Indo-Pacific reef fish community assembly[IPCOM]
		Moorea Biocode Fishes I[MBFA]
		Moorea Biocode Fishes II[MBFB]
		SWIO Biocode Fishes[SBF]
Date	:	30-January-2012
Data Type	:	Nucleotide
Distance Model	:	Kimura 2 Parameter
Marker	:	COI-5P
Codon Positions	:	1st, 2nd, 3rd
Labels	:	SampleID, Family,
Filters	:	Length > 200
Colorization	:	tax_family

Sequence Count	:	1387
Species count	:	470
Genus count	:	201
Family count	:	66
Unidentified	:	0

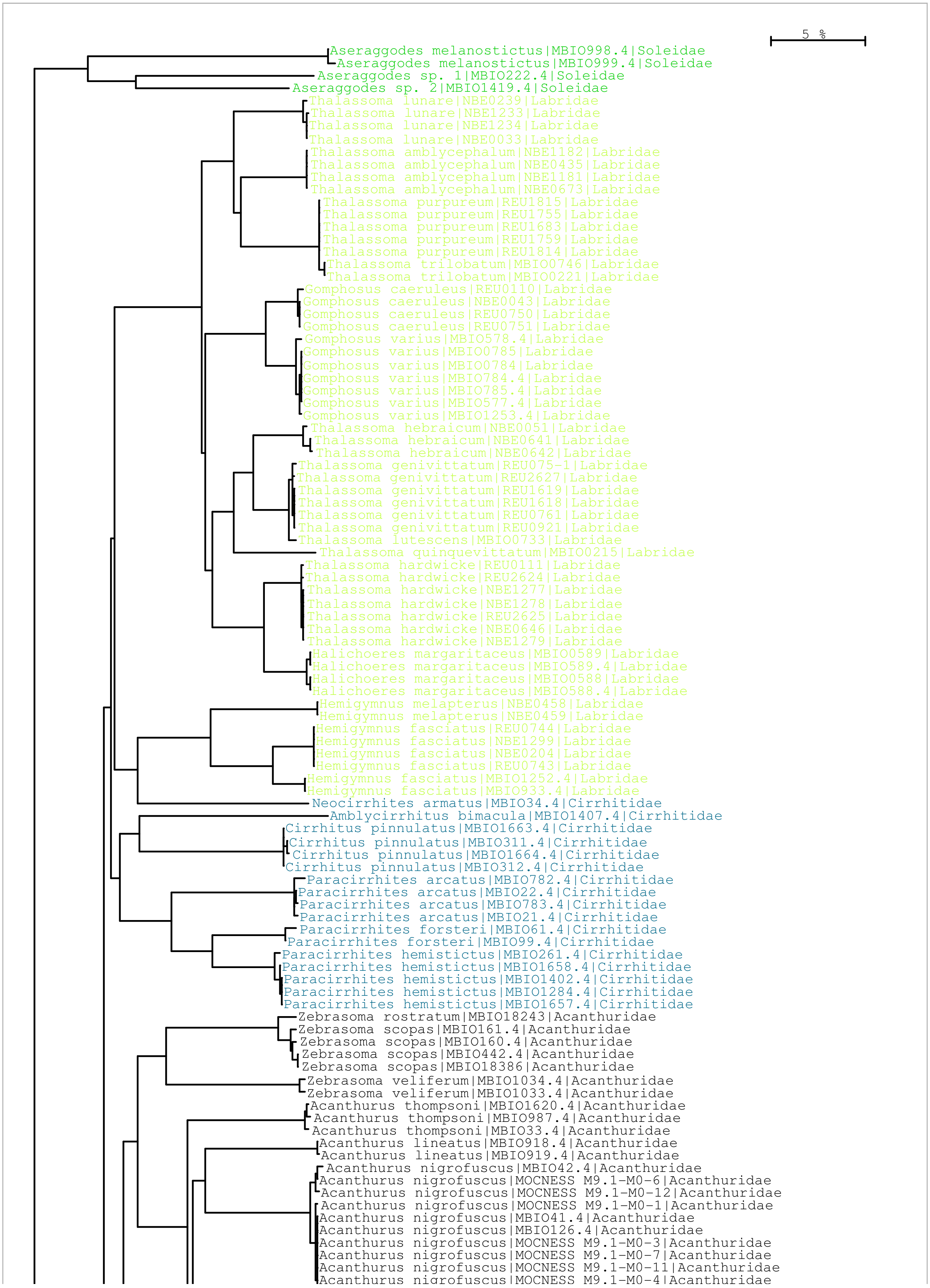
5 %
Aseraggodes melanostictus|MBIO998.4|Soleidae
Aseraggodes melanostictus|MBIO999.4|Soleidae
Aseraggodes sp. 1|MBIO222.4|Soleidae
Aseraggodes sp. 2|MBIO1419.4|Soleidae
Thalassoma lunare|NBE0239|Labridae
Thalassoma lunare|NBE1233|Labridae
Thalassoma lunare|NBE1234|Labridae
Thalassoma lunare|NBE0033|Labridae
Thalassoma amblycephalum|NBE1182|Labridae
Thalassoma amblycephalum|NBE0435|Labridae
Thalassoma amblycephalum|NBE1181|Labridae
Thalassoma amblycephalum|NBE0673|Labridae
Thalassoma purpureum|REU1815|Labridae
Thalassoma purpureum|REU1755|Labridae
Thalassoma purpureum|REU1683|Labridae
Thalassoma purpureum|REU1759|Labridae
Thalassoma purpureum|REU1814|Labridae
Thalassoma trilobatum|MBIO0746|Labridae
Thalassoma trilobatum|MBIO0221|Labridae
Gomphosus caeruleus|REU0110|Labridae
Gomphosus caeruleus|NBE0043|Labridae
Gomphosus caeruleus|REU0750|Labridae
Gomphosus caeruleus|REU0751|Labridae
Gomphosus varius|MBIO578.4|Labridae
Gomphosus varius|MBIO0785|Labridae
Gomphosus varius|MBIO0784|Labridae
Gomphosus varius|MBIO784.4|Labridae
Gomphosus varius|MBIO785.4|Labridae
Gomphosus varius|MBIO577.4|Labridae
Gomphosus varius|MBIO1253.4|Labridae
Thalassoma hebraicum|NBE0051|Labridae
Thalassoma hebraicum|NBE0641|Labridae
Thalassoma hebraicum|NBE0642|Labridae
Thalassoma genivittatum|REU075-1|Labridae
Thalassoma genivittatum|REU2627|Labridae
Thalassoma genivittatum|REU1619|Labridae
Thalassoma genivittatum|REU1618|Labridae
Thalassoma genivittatum|REU0761|Labridae
Thalassoma genivittatum|REU0921|Labridae
Thalassoma lutescens|MBIO0733|Labridae
Thalassoma quinquevittatum|MBIO0215|Labridae
Thalassoma hardwicke|REU0111|Labridae
Thalassoma hardwicke|REU2624|Labridae
Thalassoma hardwicke|NBE1277|Labridae
Thalassoma hardwicke|NBE1278|Labridae
Thalassoma hardwicke|REU2625|Labridae
Thalassoma hardwicke|NBE0646|Labridae
Thalassoma hardwicke|NBE1279|Labridae
Halichoeres margaritaceus|MBIO0589|Labridae
Halichoeres margaritaceus|MBIO589.4|Labridae
Halichoeres margaritaceus|MBIO0588|Labridae
Halichoeres margaritaceus|MBIO588.4|Labridae
Hemigymnus melapterus|NBE0458|Labridae
Hemigymnus melapterus|NBE0459|Labridae
Hemigymnus fasciatus|REU0744|Labridae
Hemigymnus fasciatus|NBE1299|Labridae
Hemigymnus fasciatus|NBE0204|Labridae
Hemigymnus fasciatus|REU0743|Labridae
Hemigymnus fasciatus|MBIO1252.4|Labridae
Hemigymnus fasciatus|MBIO933.4|Labridae
Neocirrhites armatus|MBIO34.4|Cirrhitidae
Amblycirrhitus bimacula|MBIO1407.4|Cirrhitidae
Cirrhitus pinnulatus|MBIO1663.4|Cirrhitidae
Cirrhitus pinnulatus|MBIO311.4|Cirrhitidae
Cirrhitus pinnulatus|MBIO1664.4|Cirrhitidae
Cirrhitus pinnulatus|MBIO312.4|Cirrhitidae
Paracirrhites arcatus|MBIO782.4|Cirrhitidae
Paracirrhites arcatus|MBIO22.4|Cirrhitidae
Paracirrhites arcatus|MBIO783.4|Cirrhitidae
Paracirrhites arcatus|MBIO21.4|Cirrhitidae
Paracirrhites forsteri|MBIO61.4|Cirrhitidae
Paracirrhites forsteri|MBIO99.4|Cirrhitidae
Paracirrhites hemistictus|MBIO261.4|Cirrhitidae
Paracirrhites hemistictus|MBIO1658.4|Cirrhitidae
Paracirrhites hemistictus|MBIO1402.4|Cirrhitidae
Paracirrhites hemistictus|MBIO1284.4|Cirrhitidae
Paracirrhites hemistictus|MBIO1657.4|Cirrhitidae
Zebrasoma rostratum|MBIO18243|Acanthuridae
Zebrasoma scopas|MBIO161.4|Acanthuridae
Zebrasoma scopas|MBIO160.4|Acanthuridae
Zebrasoma scopas|MBIO442.4|Acanthuridae
Zebrasoma scopas|MBIO18386|Acanthuridae
Zebrasoma veliferum|MBIO1034.4|Acanthuridae
Zebrasoma veliferum|MBIO1033.4|Acanthuridae
Acanthurus thompsoni|MBIO1620.4|Acanthuridae
Acanthurus thompsoni|MBIO987.4|Acanthuridae
Acanthurus thompsoni|MBIO33.4|Acanthuridae
Acanthurus lineatus|MBIO918.4|Acanthuridae
Acanthurus lineatus|MBIO919.4|Acanthuridae
Acanthurus nigrofuscus|MBIO42.4|Acanthuridae
Acanthurus nigrofuscus|MOCNESS M9.1-M0-6|Acanthuridae
Acanthurus nigrofuscus|MOCNESS M9.1-M0-12|Acanthuridae
Acanthurus nigrofuscus|MOCNESS M9.1-M0-1|Acanthuridae
Acanthurus nigrofuscus|MBIO41.4|Acanthuridae
Acanthurus nigrofuscus|MBIO126.4|Acanthuridae
Acanthurus nigrofuscus|MOCNESS M9.1-M0-3|Acanthuridae
Acanthurus nigrofuscus|MOCNESS M9.1-M0-7|Acanthuridae
Acanthurus nigrofuscus|MOCNESS M9.1-M0-11|Acanthuridae
Acanthurus nigrofuscus|MOCNESS M9.1-M0-4|Acanthuridae

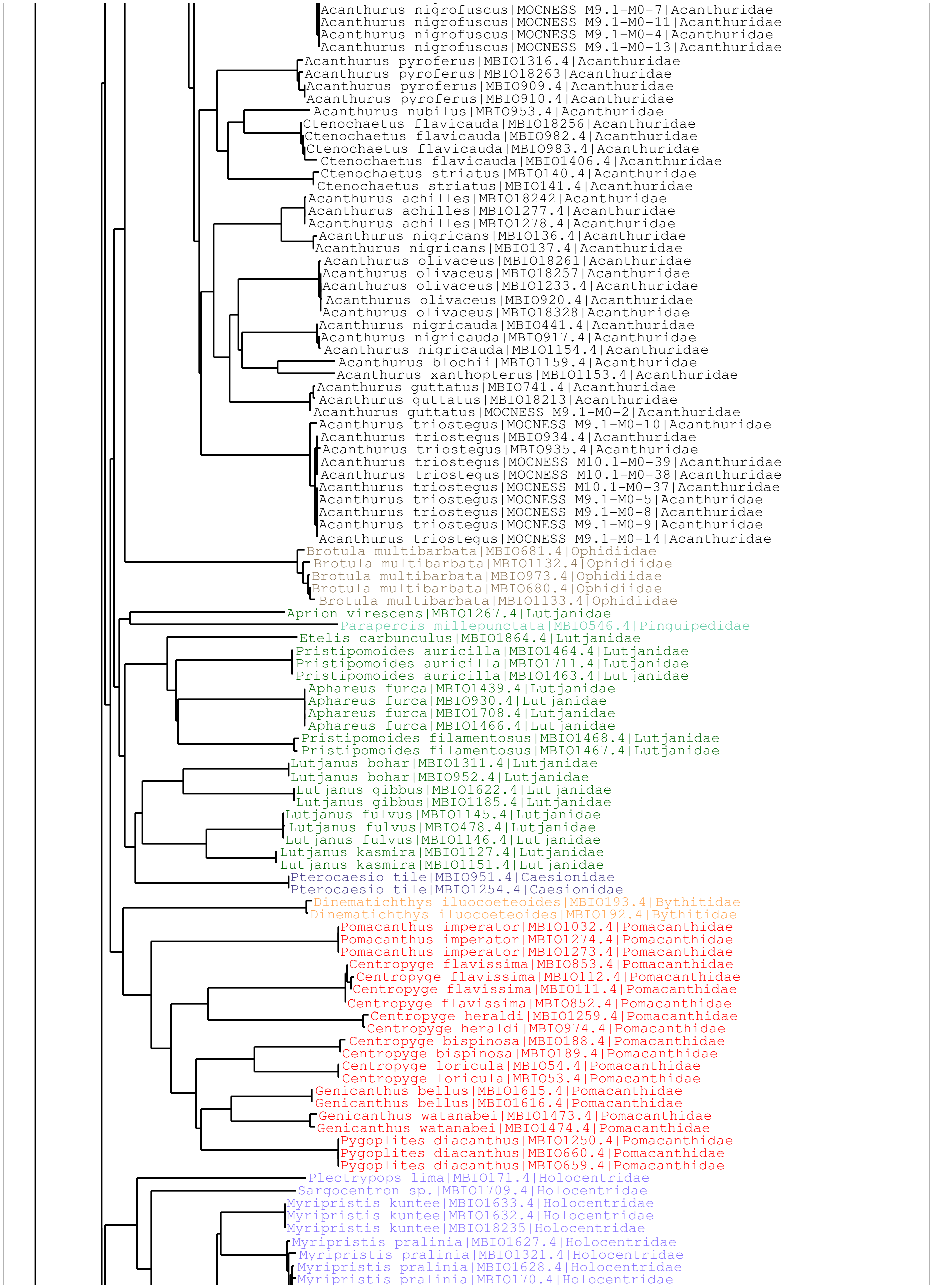
Acanthurus nigrofuscus|MOCNESS M9.1-M0-7|Acanthuridae
Acanthurus nigrofuscus|MOCNESS M9.1-M0-11|Acanthuridae
Acanthurus nigrofuscus|MOCNESS M9.1-M0-4|Acanthuridae
Acanthurus nigrofuscus|MOCNESS M9.1-M0-13|Acanthuridae
Acanthurus pyroferus|MBIO1316.4|Acanthuridae
Acanthurus pyroferus|MBIO18263|Acanthuridae
Acanthurus pyroferus|MBIO909.4|Acanthuridae
Acanthurus pyroferus|MBIO910.4|Acanthuridae
Acanthurus nubilus|MBIO953.4|Acanthuridae
Ctenochaetus flavicauda|MBIO18256|Acanthuridae
Ctenochaetus flavicauda|MBIO982.4|Acanthuridae
Ctenochaetus flavicauda|MBIO983.4|Acanthuridae
Ctenochaetus flavicauda|MBIO1406.4|Acanthuridae
Ctenochaetus striatus|MBIO140.4|Acanthuridae
Ctenochaetus striatus|MBIO141.4|Acanthuridae
Acanthurus achilles|MBIO18242|Acanthuridae
Acanthurus achilles|MBIO1277.4|Acanthuridae
Acanthurus achilles|MBIO1278.4|Acanthuridae
Acanthurus nigricans|MBIO136.4|Acanthuridae
Acanthurus nigricans|MBIO137.4|Acanthuridae
Acanthurus olivaceus|MBIO18261|Acanthuridae
Acanthurus olivaceus|MBIO18257|Acanthuridae
Acanthurus olivaceus|MBIO1233.4|Acanthuridae
Acanthurus olivaceus|MBIO920.4|Acanthuridae
Acanthurus olivaceus|MBIO18328|Acanthuridae
Acanthurus nigricauda|MBIO441.4|Acanthuridae
Acanthurus nigricauda|MBIO917.4|Acanthuridae
Acanthurus nigricauda|MBIO1154.4|Acanthuridae
Acanthurus blochii|MBIO1159.4|Acanthuridae
Acanthurus xanthopterus|MBIO1153.4|Acanthuridae
Acanthurus guttatus|MBIO741.4|Acanthuridae
Acanthurus guttatus|MBIO18213|Acanthuridae
Acanthurus guttatus|MOCNESS M9.1-M0-2|Acanthuridae
Acanthurus triostegus|MOCNESS M9.1-M0-10|Acanthuridae
Acanthurus triostegus|MBIO934.4|Acanthuridae
Acanthurus triostegus|MBIO935.4|Acanthuridae
Acanthurus triostegus|MOCNESS M10.1-M0-39|Acanthuridae
Acanthurus triostegus|MOCNESS M10.1-M0-38|Acanthuridae
Acanthurus triostegus|MOCNESS M10.1-M0-37|Acanthuridae
Acanthurus triostegus|MOCNESS M9.1-M0-5|Acanthuridae
Acanthurus triostegus|MOCNESS M9.1-M0-8|Acanthuridae
Acanthurus triostegus|MOCNESS M9.1-M0-9|Acanthuridae
Acanthurus triostegus|MOCNESS M9.1-M0-14|Acanthuridae
Brotula multibarbata|MBIO681.4|Ophidiidae
Brotula multibarbata|MBIO1132.4|Ophidiidae
Brotula multibarbata|MBIO973.4|Ophidiidae
Brotula multibarbata|MBIO680.4|Ophidiidae
Brotula multibarbata|MBIO1133.4|Ophidiidae
Aprion virescens|MBIO1267.4|Lutjanidae
Parapercis millepunctata|MBIO546.4|Pinguipedidae
Etelis carbunculus|MBIO1864.4|Lutjanidae
Pristipomoides auricilla|MBIO1464.4|Lutjanidae
Pristipomoides auricilla|MBIO1711.4|Lutjanidae
Pristipomoides auricilla|MBIO1463.4|Lutjanidae
Aphareus furca|MBIO1439.4|Lutjanidae
Aphareus furca|MBIO930.4|Lutjanidae
Aphareus furca|MBIO1708.4|Lutjanidae
Aphareus furca|MBIO1466.4|Lutjanidae
Pristipomoides filamentosus|MBIO1468.4|Lutjanidae
Pristipomoides filamentosus|MBIO1467.4|Lutjanidae
Lutjanus bohar|MBIO1311.4|Lutjanidae
Lutjanus bohar|MBIO952.4|Lutjanidae
Lutjanus gibbus|MBIO1622.4|Lutjanidae
Lutjanus gibbus|MBIO1185.4|Lutjanidae
Lutjanus fulvus|MBIO1145.4|Lutjanidae
Lutjanus fulvus|MBIO478.4|Lutjanidae
Lutjanus fulvus|MBIO1146.4|Lutjanidae
Lutjanus kasmira|MBIO1127.4|Lutjanidae
Lutjanus kasmira|MBIO1151.4|Lutjanidae
Pterocaesio tile|MBIO951.4|Caesionidae
Pterocaesio tile|MBIO1254.4|Caesionidae
Dinematichthys iluocoeteoides|MBIO193.4|Bythitidae
Dinematichthys iluocoeteoides|MBIO192.4|Bythitidae
Pomacanthus imperator|MBIO1032.4|Pomacanthidae
Pomacanthus imperator|MBIO1274.4|Pomacanthidae
Pomacanthus imperator|MBIO1273.4|Pomacanthidae
Centropyge flavissima|MBIO853.4|Pomacanthidae
Centropyge flavissima|MBIO112.4|Pomacanthidae
Centropyge flavissima|MBIO111.4|Pomacanthidae
Centropyge flavissima|MBIO852.4|Pomacanthidae
Centropyge heraldi|MBIO1259.4|Pomacanthidae
Centropyge heraldi|MBIO974.4|Pomacanthidae
Centropyge bispinosa|MBIO188.4|Pomacanthidae
Centropyge bispinosa|MBIO189.4|Pomacanthidae
Centropyge loricula|MBIO54.4|Pomacanthidae
Centropyge loricula|MBIO53.4|Pomacanthidae
Genicanthus bellus|MBIO1615.4|Pomacanthidae
Genicanthus bellus|MBIO1616.4|Pomacanthidae
Genicanthus watanabei|MBIO1473.4|Pomacanthidae
Genicanthus watanabei|MBIO1474.4|Pomacanthidae
Pygoplites diacanthus|MBIO1250.4|Pomacanthidae
Pygoplites diacanthus|MBIO660.4|Pomacanthidae
Pygoplites diacanthus|MBIO659.4|Pomacanthidae
Plectrypops lima|MBIO171.4|Holocentridae
Sargocentron sp.|MBIO1709.4|Holocentridae
Myripristis kuntee|MBIO1633.4|Holocentridae
Myripristis kuntee|MBIO1632.4|Holocentridae
Myripristis kuntee|MBIO18235|Holocentridae
Myripristis pralinia|MBIO1627.4|Holocentridae
Myripristis pralinia|MBIO1321.4|Holocentridae
Myripristis pralinia|MBIO1628.4|Holocentridae
Myripristis pralinia|MBIO170.4|Holocentridae

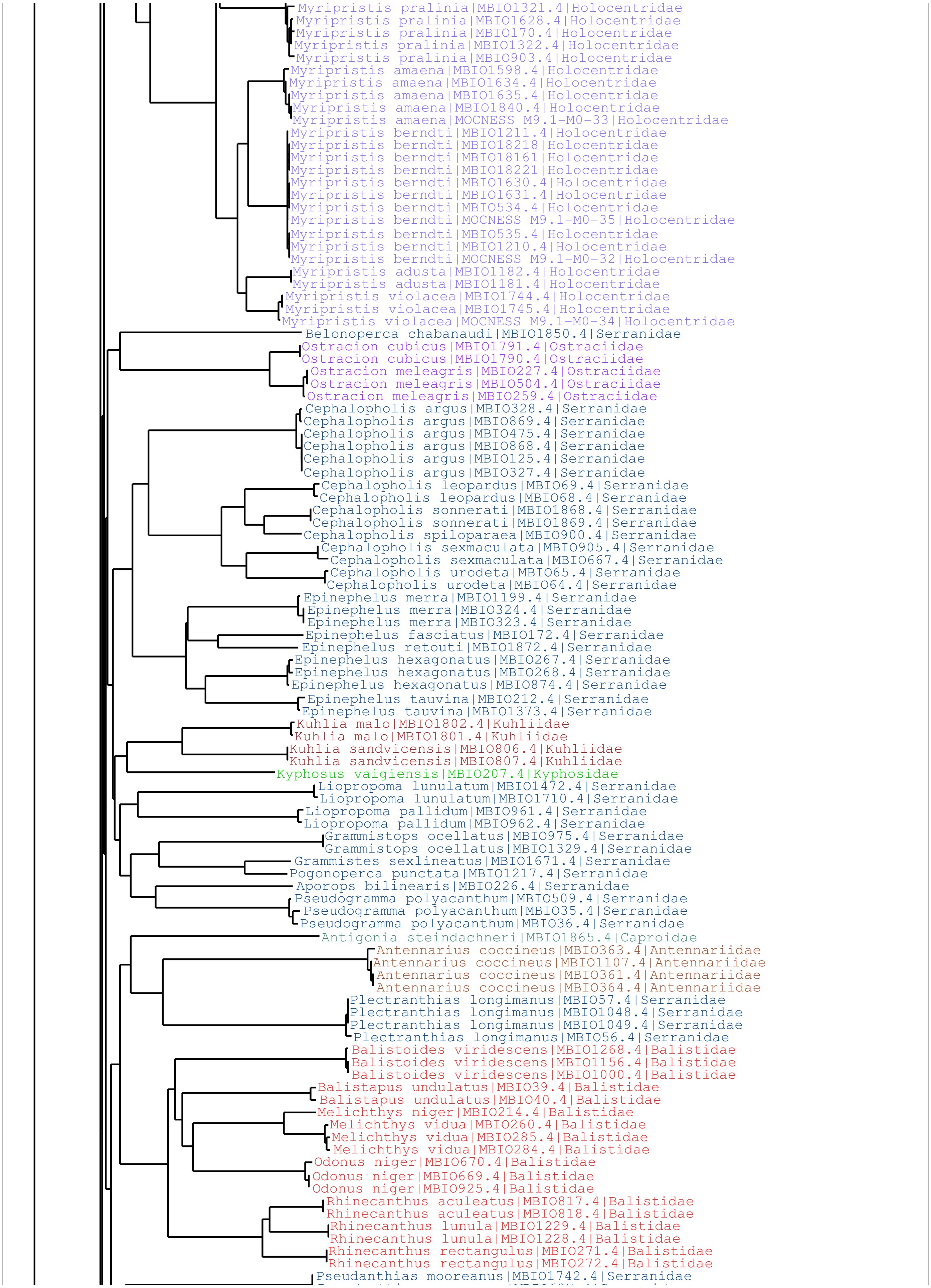
Myripristis pralinia|MBIO1321.4|Holocentridae
Myripristis pralinia|MBIO1628.4|Holocentridae
Myripristis pralinia|MBIO170.4|Holocentridae
Myripristis pralinia|MBIO1322.4|Holocentridae
Myripristis pralinia|MBIO903.4|Holocentridae
Myripristis amaena|MBIO1598.4|Holocentridae
Myripristis amaena|MBIO1634.4|Holocentridae
Myripristis amaena|MBIO1635.4|Holocentridae
Myripristis amaena|MBIO1840.4|Holocentridae
Myripristis amaena|MOCNESS M9.1-M0-33|Holocentridae
Myripristis berndti|MBIO1211.4|Holocentridae
Myripristis berndti|MBIO18218|Holocentridae
Myripristis berndti|MBIO18161|Holocentridae
Myripristis berndti|MBIO18221|Holocentridae
Myripristis berndti|MBIO1630.4|Holocentridae
Myripristis berndti|MBIO1631.4|Holocentridae
Myripristis berndti|MBIO534.4|Holocentridae
Myripristis berndti|MOCNESS M9.1-M0-35|Holocentridae
Myripristis berndti|MBIO535.4|Holocentridae
Myripristis berndti|MBIO1210.4|Holocentridae
Myripristis berndti|MOCNESS M9.1-M0-32|Holocentridae
Myripristis adusta|MBIO1182.4|Holocentridae
Myripristis adusta|MBIO1181.4|Holocentridae
Myripristis violacea|MBIO1744.4|Holocentridae
Myripristis violacea|MBIO1745.4|Holocentridae
Myripristis violacea|MOCNESS M9.1-M0-34|Holocentridae
Belonoperca chabanaudi|MBIO1850.4|Serranidae
Ostracion cubicus|MBIO1791.4|Ostraciidae
Ostracion cubicus|MBIO1790.4|Ostraciidae
Ostracion meleagris|MBIO227.4|Ostraciidae
Ostracion meleagris|MBIO504.4|Ostraciidae
Ostracion meleagris|MBIO259.4|Ostraciidae
Cephalopholis argus|MBIO328.4|Serranidae
Cephalopholis argus|MBIO869.4|Serranidae
Cephalopholis argus|MBIO475.4|Serranidae
Cephalopholis argus|MBIO868.4|Serranidae
Cephalopholis argus|MBIO125.4|Serranidae
Cephalopholis argus|MBIO327.4|Serranidae
Cephalopholis leopardus|MBIO69.4|Serranidae
Cephalopholis leopardus|MBIO68.4|Serranidae
Cephalopholis sonnerati|MBIO1868.4|Serranidae
Cephalopholis sonnerati|MBIO1869.4|Serranidae
Cephalopholis spiloparaea|MBIO900.4|Serranidae
Cephalopholis sexmaculata|MBIO905.4|Serranidae
Cephalopholis sexmaculata|MBIO667.4|Serranidae
Cephalopholis urodeta|MBIO65.4|Serranidae
Cephalopholis urodeta|MBIO64.4|Serranidae
Epinephelus merra|MBIO1199.4|Serranidae
Epinephelus merra|MBIO324.4|Serranidae
Epinephelus merra|MBIO323.4|Serranidae
Epinephelus fasciatus|MBIO172.4|Serranidae
Epinephelus retouti|MBIO1872.4|Serranidae
Epinephelus hexagonatus|MBIO267.4|Serranidae
Epinephelus hexagonatus|MBIO268.4|Serranidae
Epinephelus hexagonatus|MBIO874.4|Serranidae
Epinephelus tauvina|MBIO212.4|Serranidae
Epinephelus tauvina|MBIO1373.4|Serranidae
Kuhlia malo|MBIO1802.4|Kuhliidae
Kuhlia malo|MBIO1801.4|Kuhliidae
Kuhlia sandvicensis|MBIO806.4|Kuhliidae
Kuhlia sandvicensis|MBIO807.4|Kuhliidae
Kyphosus vaigiensis|MBIO207.4|Kyphosidae
Liopropoma lunulatum|MBIO1472.4|Serranidae
Liopropoma lunulatum|MBIO1710.4|Serranidae
Liopropoma pallidum|MBIO961.4|Serranidae
Liopropoma pallidum|MBIO962.4|Serranidae
Grammistops ocellatus|MBIO975.4|Serranidae
Grammistops ocellatus|MBIO1329.4|Serranidae
Grammistes sexlineatus|MBIO1671.4|Serranidae
Pogonoperca punctata|MBIO1217.4|Serranidae
Aporops bilinearis|MBIO226.4|Serranidae
Pseudogramma polyacanthum|MBIO509.4|Serranidae
Pseudogramma polyacanthum|MBIO35.4|Serranidae
Pseudogramma polyacanthum|MBIO36.4|Serranidae
Antigonia steindachneri|MBIO1865.4|Caproidae
Antennarius coccineus|MBIO363.4|Antennariidae
Antennarius coccineus|MBIO1107.4|Antennariidae
Antennarius coccineus|MBIO361.4|Antennariidae
Antennarius coccineus|MBIO364.4|Antennariidae
Plectranthias longimanus|MBIO57.4|Serranidae
Plectranthias longimanus|MBIO1048.4|Serranidae
Plectranthias longimanus|MBIO1049.4|Serranidae
Plectranthias longimanus|MBIO56.4|Serranidae
Balistoides viridescens|MBIO1268.4|Balistidae
Balistoides viridescens|MBIO1156.4|Balistidae
Balistoides viridescens|MBIO1000.4|Balistidae
Balistapus undulatus|MBIO39.4|Balistidae
Balistapus undulatus|MBIO40.4|Balistidae
Melichthys niger|MBIO214.4|Balistidae
Melichthys vidua|MBIO260.4|Balistidae
Melichthys vidua|MBIO285.4|Balistidae
Melichthys vidua|MBIO284.4|Balistidae
Odonus niger|MBIO670.4|Balistidae
Odonus niger|MBIO669.4|Balistidae
Odonus niger|MBIO925.4|Balistidae
Rhinecanthus aculeatus|MBIO817.4|Balistidae
Rhinecanthus aculeatus|MBIO818.4|Balistidae
Rhinecanthus lunula|MBIO1229.4|Balistidae
Rhinecanthus lunula|MBIO1228.4|Balistidae
Rhinecanthus rectangulus|MBIO271.4|Balistidae
Rhinecanthus rectangulus|MBIO272.4|Balistidae
Pseudanthias mooreanus|MBIO1742.4|Serranidae

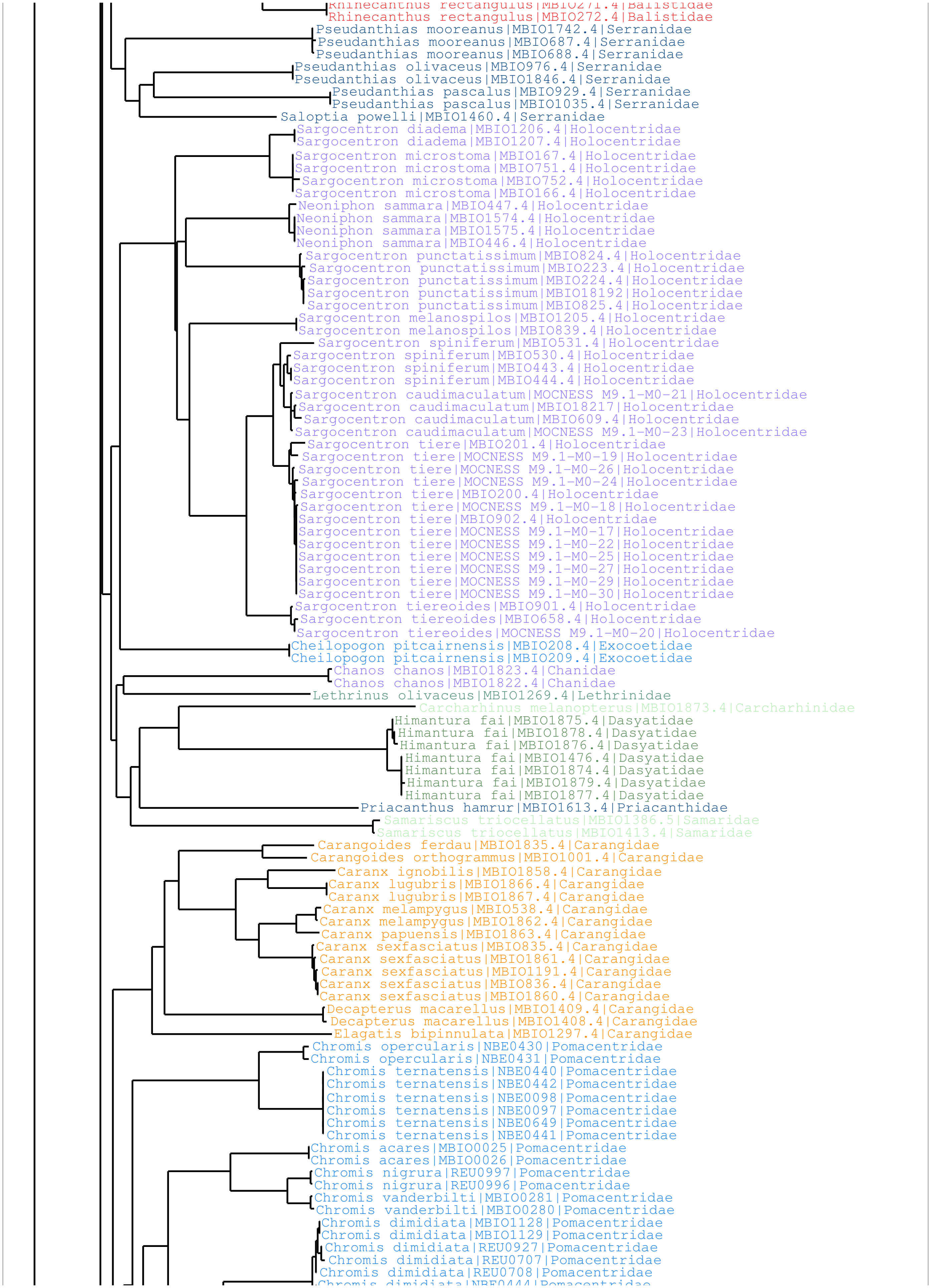
Rhinecanthus rectangulus|MBIO272.4|Balistidae
Pseudanthias mooreanus|MBIO1742.4|Serranidae
Pseudanthias mooreanus|MBIO687.4|Serranidae
Pseudanthias mooreanus|MBIO688.4|Serranidae
Pseudanthias olivaceus|MBIO976.4|Serranidae
Pseudanthias olivaceus|MBIO1846.4|Serranidae
Pseudanthias pascalus|MBIO929.4|Serranidae
Pseudanthias pascalus|MBIO1035.4|Serranidae
Saloptia powelli|MBIO1460.4|Serranidae
Sargocentron diadema|MBIO1206.4|Holocentridae
Sargocentron diadema|MBIO1207.4|Holocentridae
Sargocentron microstoma|MBIO167.4|Holocentridae
Sargocentron microstoma|MBIO751.4|Holocentridae
Sargocentron microstoma|MBIO752.4|Holocentridae
Sargocentron microstoma|MBIO166.4|Holocentridae
Neoniphon sammara|MBIO447.4|Holocentridae
Neoniphon sammara|MBIO1574.4|Holocentridae
Neoniphon sammara|MBIO1575.4|Holocentridae
Neoniphon sammara|MBIO446.4|Holocentridae
Sargocentron punctatissimum|MBIO824.4|Holocentridae
Sargocentron punctatissimum|MBIO223.4|Holocentridae
Sargocentron punctatissimum|MBIO224.4|Holocentridae
Sargocentron punctatissimum|MBIO18192|Holocentridae
Sargocentron punctatissimum|MBIO825.4|Holocentridae
Sargocentron melanospilos|MBIO1205.4|Holocentridae
Sargocentron melanospilos|MBIO839.4|Holocentridae
Sargocentron spiniferum|MBIO531.4|Holocentridae
Sargocentron spiniferum|MBIO530.4|Holocentridae
Sargocentron spiniferum|MBIO443.4|Holocentridae
Sargocentron spiniferum|MBIO444.4|Holocentridae
Sargocentron caudimaculatum|MOCNESS M9.1-M0-21|Holocentridae
Sargocentron caudimaculatum|MBIO18217|Holocentridae
Sargocentron caudimaculatum|MBIO609.4|Holocentridae
Sargocentron caudimaculatum|MOCNESS M9.1-M0-23|Holocentridae
Sargocentron tiere|MBIO201.4|Holocentridae
Sargocentron tiere|MOCNESS M9.1-M0-19|Holocentridae
Sargocentron tiere|MOCNESS M9.1-M0-26|Holocentridae
Sargocentron tiere|MOCNESS M9.1-M0-24|Holocentridae
Sargocentron tiere|MBIO200.4|Holocentridae
Sargocentron tiere|MOCNESS M9.1-M0-18|Holocentridae
Sargocentron tiere|MBIO902.4|Holocentridae
Sargocentron tiere|MOCNESS M9.1-M0-17|Holocentridae
Sargocentron tiere|MOCNESS M9.1-M0-22|Holocentridae
Sargocentron tiere|MOCNESS M9.1-M0-25|Holocentridae
Sargocentron tiere|MOCNESS M9.1-M0-27|Holocentridae
Sargocentron tiere|MOCNESS M9.1-M0-29|Holocentridae
Sargocentron tiere|MOCNESS M9.1-M0-30|Holocentridae
Sargocentron tiereoides|MBIO901.4|Holocentridae
Sargocentron tiereoides|MBIO658.4|Holocentridae
Sargocentron tiereoides|MOCNESS M9.1-M0-20|Holocentridae
Cheilopogon pitcairnensis|MBIO208.4|Exocoetidae
Cheilopogon pitcairnensis|MBIO209.4|Exocoetidae
Chanos chanos|MBIO1823.4|Chanidae
Chanos chanos|MBIO1822.4|Chanidae
Lethrinus olivaceus|MBIO1269.4|Lethrinidae
Carcharhinus melanopterus|MBIO1873.4|Carcharhinidae
Himantura fai|MBIO1875.4|Dasyatidae
Himantura fai|MBIO1878.4|Dasyatidae
Himantura fai|MBIO1876.4|Dasyatidae
Himantura fai|MBIO1476.4|Dasyatidae
Himantura fai|MBIO1874.4|Dasyatidae
Himantura fai|MBIO1879.4|Dasyatidae
Himantura fai|MBIO1877.4|Dasyatidae
Priacanthus hamrur|MBIO1613.4|Priacanthidae
Samariscus triocellatus|MBIO1386.5|Samaridae
Samariscus triocellatus|MBIO1413.4|Samaridae
Carangoides ferdau|MBIO1835.4|Carangidae
Carangoides orthogrammus|MBIO1001.4|Carangidae
Caranx ignobilis|MBIO1858.4|Carangidae
Caranx lugubris|MBIO1866.4|Carangidae
Caranx lugubris|MBIO1867.4|Carangidae
Caranx melampygus|MBIO538.4|Carangidae
Caranx melampygus|MBIO1862.4|Carangidae
Caranx papuensis|MBIO1863.4|Carangidae
Caranx sexfasciatus|MBIO835.4|Carangidae
Caranx sexfasciatus|MBIO1861.4|Carangidae
Caranx sexfasciatus|MBIO1191.4|Carangidae
Caranx sexfasciatus|MBIO836.4|Carangidae
Caranx sexfasciatus|MBIO1860.4|Carangidae
Decapterus macarellus|MBIO1409.4|Carangidae
Decapterus macarellus|MBIO1408.4|Carangidae
Elagatis bipinnulata|MBIO1297.4|Carangidae
Chromis opercularis|NBE0430|Pomacentridae
Chromis opercularis|NBE0431|Pomacentridae
Chromis ternatensis|NBE0440|Pomacentridae
Chromis ternatensis|NBE0442|Pomacentridae
Chromis ternatensis|NBE0098|Pomacentridae
Chromis ternatensis|NBE0097|Pomacentridae
Chromis ternatensis|NBE0649|Pomacentridae
Chromis ternatensis|NBE0441|Pomacentridae
Chromis acares|MBIO0025|Pomacentridae
Chromis acares|MBIO0026|Pomacentridae
Chromis nigrura|REU0997|Pomacentridae
Chromis nigrura|REU0996|Pomacentridae
Chromis vanderbilti|MBIO0281|Pomacentridae
Chromis vanderbilti|MBIO0280|Pomacentridae
Chromis dimidiata|MBIO1128|Pomacentridae
Chromis dimidiata|MBIO1129|Pomacentridae
Chromis dimidiata|REU0927|Pomacentridae
Chromis dimidiata|REU0707|Pomacentridae
Chromis dimidiata|REU0708|Pomacentridae

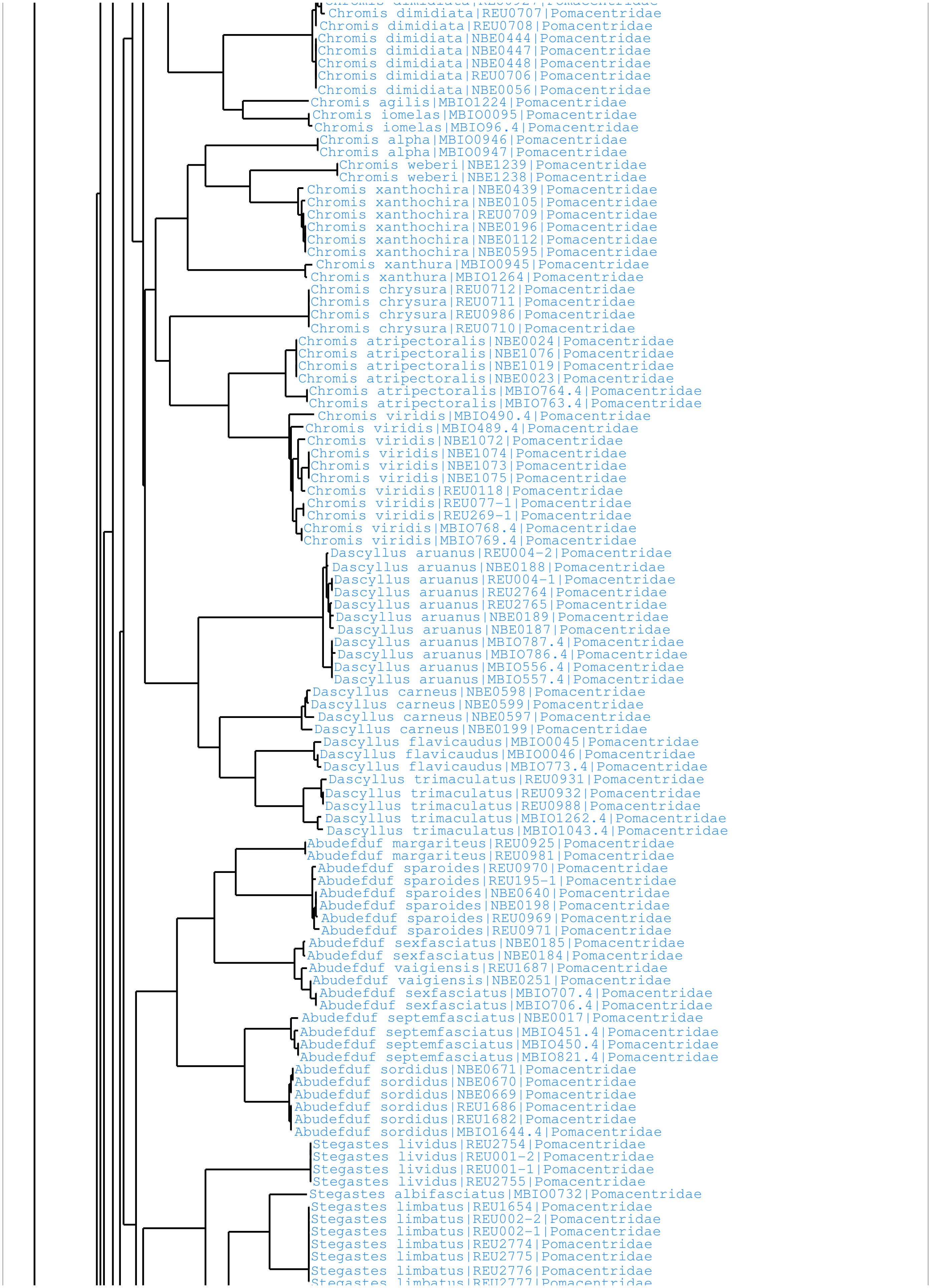
Chromis dimidiata|REU0707|Pomacentridae
Chromis dimidiata|REU0708|Pomacentridae
Chromis dimidiata|NBE0444|Pomacentridae
Chromis dimidiata|NBE0447|Pomacentridae
Chromis dimidiata|NBE0448|Pomacentridae
Chromis dimidiata|REU0706|Pomacentridae
Chromis dimidiata|NBE0056|Pomacentridae
Chromis agilis|MBIO1224|Pomacentridae
Chromis iomelas|MBIO0095|Pomacentridae
Chromis iomelas|MBIO96.4|Pomacentridae
Chromis alpha|MBIO0946|Pomacentridae
Chromis alpha|MBIO0947|Pomacentridae
Chromis weberi|NBE1239|Pomacentridae
Chromis weberi|NBE1238|Pomacentridae
Chromis xanthochira|NBE0439|Pomacentridae
Chromis xanthochira|NBE0105|Pomacentridae
Chromis xanthochira|REU0709|Pomacentridae
Chromis xanthochira|NBE0196|Pomacentridae
Chromis xanthochira|NBE0112|Pomacentridae
Chromis xanthochira|NBE0595|Pomacentridae
Chromis xanthura|MBIO0945|Pomacentridae
Chromis xanthura|MBIO1264|Pomacentridae
Chromis chrysura|REU0712|Pomacentridae
Chromis chrysura|REU0711|Pomacentridae
Chromis chrysura|REU0986|Pomacentridae
Chromis chrysura|REU0710|Pomacentridae
Chromis atripectoralis|NBE0024|Pomacentridae
Chromis atripectoralis|NBE1076|Pomacentridae
Chromis atripectoralis|NBE1019|Pomacentridae
Chromis atripectoralis|NBE0023|Pomacentridae
Chromis atripectoralis|MBIO764.4|Pomacentridae
Chromis atripectoralis|MBIO763.4|Pomacentridae
Chromis viridis|MBIO490.4|Pomacentridae
Chromis viridis|MBIO489.4|Pomacentridae
Chromis viridis|NBE1072|Pomacentridae
Chromis viridis|NBE1074|Pomacentridae
Chromis viridis|NBE1073|Pomacentridae
Chromis viridis|NBE1075|Pomacentridae
Chromis viridis|REU0118|Pomacentridae
Chromis viridis|REU077-1|Pomacentridae
Chromis viridis|REU269-1|Pomacentridae
Chromis viridis|MBIO768.4|Pomacentridae
Chromis viridis|MBIO769.4|Pomacentridae
Dascyllus aruanus|REU004-2|Pomacentridae
Dascyllus aruanus|NBE0188|Pomacentridae
Dascyllus aruanus|REU004-1|Pomacentridae
Dascyllus aruanus|REU2764|Pomacentridae
Dascyllus aruanus|REU2765|Pomacentridae
Dascyllus aruanus|NBE0189|Pomacentridae
Dascyllus aruanus|NBE0187|Pomacentridae
Dascyllus aruanus|MBIO787.4|Pomacentridae
Dascyllus aruanus|MBIO786.4|Pomacentridae
Dascyllus aruanus|MBIO556.4|Pomacentridae
Dascyllus aruanus|MBIO557.4|Pomacentridae
Dascyllus carneus|NBE0598|Pomacentridae
Dascyllus carneus|NBE0599|Pomacentridae
Dascyllus carneus|NBE0597|Pomacentridae
Dascyllus carneus|NBE0199|Pomacentridae
Dascyllus flavicaudus|MBIO0045|Pomacentridae
Dascyllus flavicaudus|MBIO0046|Pomacentridae
Dascyllus flavicaudus|MBIO773.4|Pomacentridae
Dascyllus trimaculatus|REU0931|Pomacentridae
Dascyllus trimaculatus|REU0932|Pomacentridae
Dascyllus trimaculatus|REU0988|Pomacentridae
Dascyllus trimaculatus|MBIO1262.4|Pomacentridae
Dascyllus trimaculatus|MBIO1043.4|Pomacentridae
Abudefduf margariteus|REU0925|Pomacentridae
Abudefduf margariteus|REU0981|Pomacentridae
Abudefduf sparoides|REU0970|Pomacentridae
Abudefduf sparoides|REU195-1|Pomacentridae
Abudefduf sparoides|NBE0640|Pomacentridae
Abudefduf sparoides|NBE0198|Pomacentridae
Abudefduf sparoides|REU0969|Pomacentridae
Abudefduf sparoides|REU0971|Pomacentridae
Abudefduf sexfasciatus|NBE0185|Pomacentridae
Abudefduf sexfasciatus|NBE0184|Pomacentridae
Abudefduf vaigiensis|REU1687|Pomacentridae
Abudefduf vaigiensis|NBE0251|Pomacentridae
Abudefduf sexfasciatus|MBIO707.4|Pomacentridae
Abudefduf sexfasciatus|MBIO706.4|Pomacentridae
Abudefduf septemfasciatus|NBE0017|Pomacentridae
Abudefduf septemfasciatus|MBIO451.4|Pomacentridae
Abudefduf septemfasciatus|MBIO450.4|Pomacentridae
Abudefduf septemfasciatus|MBIO821.4|Pomacentridae
Abudefduf sordidus|NBE0671|Pomacentridae
Abudefduf sordidus|NBE0670|Pomacentridae
Abudefduf sordidus|NBE0669|Pomacentridae
Abudefduf sordidus|REU1686|Pomacentridae
Abudefduf sordidus|REU1682|Pomacentridae
Abudefduf sordidus|MBIO1644.4|Pomacentridae
Stegastes lividus|REU2754|Pomacentridae
Stegastes lividus|REU001-2|Pomacentridae
Stegastes lividus|REU001-1|Pomacentridae
Stegastes lividus|REU2755|Pomacentridae
Stegastes albifasciatus|MBIO0732|Pomacentridae
Stegastes limbatus|REU1654|Pomacentridae
Stegastes limbatus|REU002-2|Pomacentridae
Stegastes limbatus|REU002-1|Pomacentridae
Stegastes limbatus|REU2774|Pomacentridae
Stegastes limbatus|REU2775|Pomacentridae
Stegastes limbatus|REU2776|Pomacentridae
Stegastes limbatus|REU2777|Pomacentridae

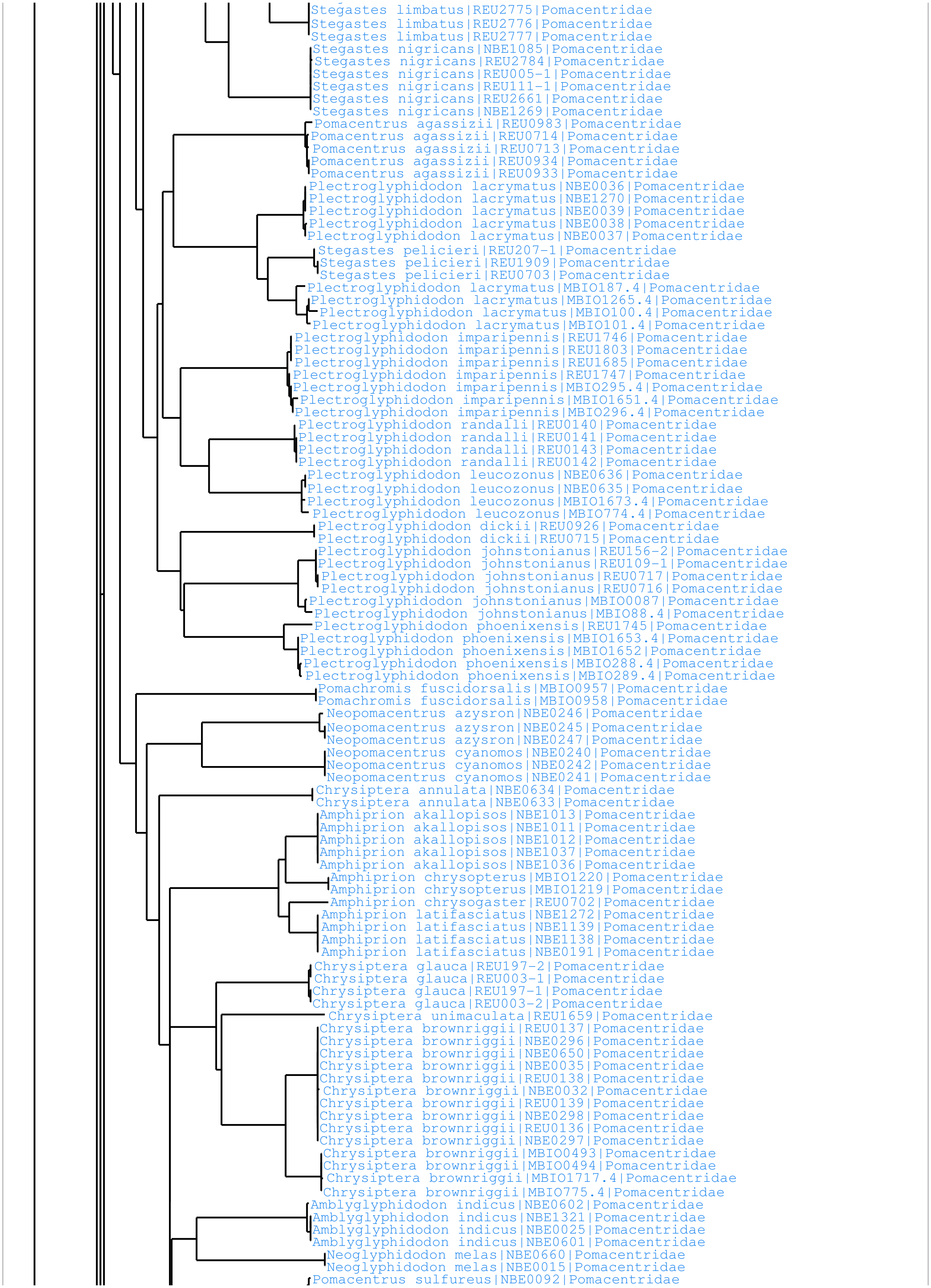
Stegastes limbatus|REU2775|Pomacentridae
Stegastes limbatus|REU2776|Pomacentridae
Stegastes limbatus|REU2777|Pomacentridae
Stegastes nigricans|NBE1085|Pomacentridae
Stegastes nigricans|REU2784|Pomacentridae
Stegastes nigricans|REU005-1|Pomacentridae
Stegastes nigricans|REU111-1|Pomacentridae
Stegastes nigricans|REU2661|Pomacentridae
Stegastes nigricans|NBE1269|Pomacentridae
Pomacentrus agassizii|REU0983|Pomacentridae
Pomacentrus agassizii|REU0714|Pomacentridae
Pomacentrus agassizii|REU0713|Pomacentridae
Pomacentrus agassizii|REU0934|Pomacentridae
Pomacentrus agassizii|REU0933|Pomacentridae
Plectroglyphidodon lacrymatus|NBE0036|Pomacentridae
Plectroglyphidodon lacrymatus|NBE1270|Pomacentridae
Plectroglyphidodon lacrymatus|NBE0039|Pomacentridae
Plectroglyphidodon lacrymatus|NBE0038|Pomacentridae
Plectroglyphidodon lacrymatus|NBE0037|Pomacentridae
Stegastes pelicieri|REU207-1|Pomacentridae
Stegastes pelicieri|REU1909|Pomacentridae
Stegastes pelicieri|REU0703|Pomacentridae
Plectroglyphidodon lacrymatus|MBIO187.4|Pomacentridae
Plectroglyphidodon lacrymatus|MBIO1265.4|Pomacentridae
Plectroglyphidodon lacrymatus|MBIO100.4|Pomacentridae
Plectroglyphidodon lacrymatus|MBIO101.4|Pomacentridae
Plectroglyphidodon imparipennis|REU1746|Pomacentridae
Plectroglyphidodon imparipennis|REU1803|Pomacentridae
Plectroglyphidodon imparipennis|REU1685|Pomacentridae
Plectroglyphidodon imparipennis|REU1747|Pomacentridae
Plectroglyphidodon imparipennis|MBIO295.4|Pomacentridae
Plectroglyphidodon imparipennis|MBIO1651.4|Pomacentridae
Plectroglyphidodon imparipennis|MBIO296.4|Pomacentridae
Plectroglyphidodon randalli|REU0140|Pomacentridae
Plectroglyphidodon randalli|REU0141|Pomacentridae
Plectroglyphidodon randalli|REU0143|Pomacentridae
Plectroglyphidodon randalli|REU0142|Pomacentridae
Plectroglyphidodon leucozonus|NBE0636|Pomacentridae
Plectroglyphidodon leucozonus|NBE0635|Pomacentridae
Plectroglyphidodon leucozonus|MBIO1673.4|Pomacentridae
Plectroglyphidodon leucozonus|MBIO774.4|Pomacentridae
Plectroglyphidodon dickii|REU0926|Pomacentridae
Plectroglyphidodon dickii|REU0715|Pomacentridae
Plectroglyphidodon johnstonianus|REU156-2|Pomacentridae
Plectroglyphidodon johnstonianus|REU109-1|Pomacentridae
Plectroglyphidodon johnstonianus|REU0717|Pomacentridae
Plectroglyphidodon johnstonianus|REU0716|Pomacentridae
Plectroglyphidodon johnstonianus|MBIO0087|Pomacentridae
Plectroglyphidodon johnstonianus|MBIO88.4|Pomacentridae
Plectroglyphidodon phoenixensis|REU1745|Pomacentridae
Plectroglyphidodon phoenixensis|MBIO1653.4|Pomacentridae
Plectroglyphidodon phoenixensis|MBIO1652|Pomacentridae
Plectroglyphidodon phoenixensis|MBIO288.4|Pomacentridae
Plectroglyphidodon phoenixensis|MBIO289.4|Pomacentridae
Pomachromis fuscidorsalis|MBIO0957|Pomacentridae
Pomachromis fuscidorsalis|MBIO0958|Pomacentridae
Neopomacentrus azysron|NBE0246|Pomacentridae
Neopomacentrus azysron|NBE0245|Pomacentridae
Neopomacentrus azysron|NBE0247|Pomacentridae
Neopomacentrus cyanomos|NBE0240|Pomacentridae
Neopomacentrus cyanomos|NBE0242|Pomacentridae
Neopomacentrus cyanomos|NBE0241|Pomacentridae
Chrysiptera annulata|NBE0634|Pomacentridae
Chrysiptera annulata|NBE0633|Pomacentridae
Amphiprion akallopisos|NBE1013|Pomacentridae
Amphiprion akallopisos|NBE1011|Pomacentridae
Amphiprion akallopisos|NBE1012|Pomacentridae
Amphiprion akallopisos|NBE1037|Pomacentridae
Amphiprion akallopisos|NBE1036|Pomacentridae
Amphiprion chrysopterus|MBIO1220|Pomacentridae
Amphiprion chrysopterus|MBIO1219|Pomacentridae
Amphiprion chrysogaster|REU0702|Pomacentridae
Amphiprion latifasciatus|NBE1272|Pomacentridae
Amphiprion latifasciatus|NBE1139|Pomacentridae
Amphiprion latifasciatus|NBE1138|Pomacentridae
Amphiprion latifasciatus|NBE0191|Pomacentridae
Chrysiptera glauca|REU197-2|Pomacentridae
Chrysiptera glauca|REU003-1|Pomacentridae
Chrysiptera glauca|REU197-1|Pomacentridae
Chrysiptera glauca|REU003-2|Pomacentridae
Chrysiptera unimaculata|REU1659|Pomacentridae
Chrysiptera brownriggii|REU0137|Pomacentridae
Chrysiptera brownriggii|NBE0296|Pomacentridae
Chrysiptera brownriggii|NBE0650|Pomacentridae
Chrysiptera brownriggii|NBE0035|Pomacentridae
Chrysiptera brownriggii|REU0138|Pomacentridae
Chrysiptera brownriggii|NBE0032|Pomacentridae
Chrysiptera brownriggii|REU0139|Pomacentridae
Chrysiptera brownriggii|NBE0298|Pomacentridae
Chrysiptera brownriggii|REU0136|Pomacentridae
Chrysiptera brownriggii|NBE0297|Pomacentridae
Chrysiptera brownriggii|MBIO0493|Pomacentridae
Chrysiptera brownriggii|MBIO0494|Pomacentridae
Chrysiptera brownriggii|MBIO1717.4|Pomacentridae
Chrysiptera brownriggii|MBIO775.4|Pomacentridae
Amblyglyphidodon indicus|NBE0602|Pomacentridae
Amblyglyphidodon indicus|NBE1321|Pomacentridae
Amblyglyphidodon indicus|NBE0025|Pomacentridae
Amblyglyphidodon indicus|NBE0601|Pomacentridae
Neoglyphidodon melas|NBE0660|Pomacentridae
Neoglyphidodon melas|NBE0015|Pomacentridae
Pomacentrus sulfureus|NBE0092|Pomacentridae

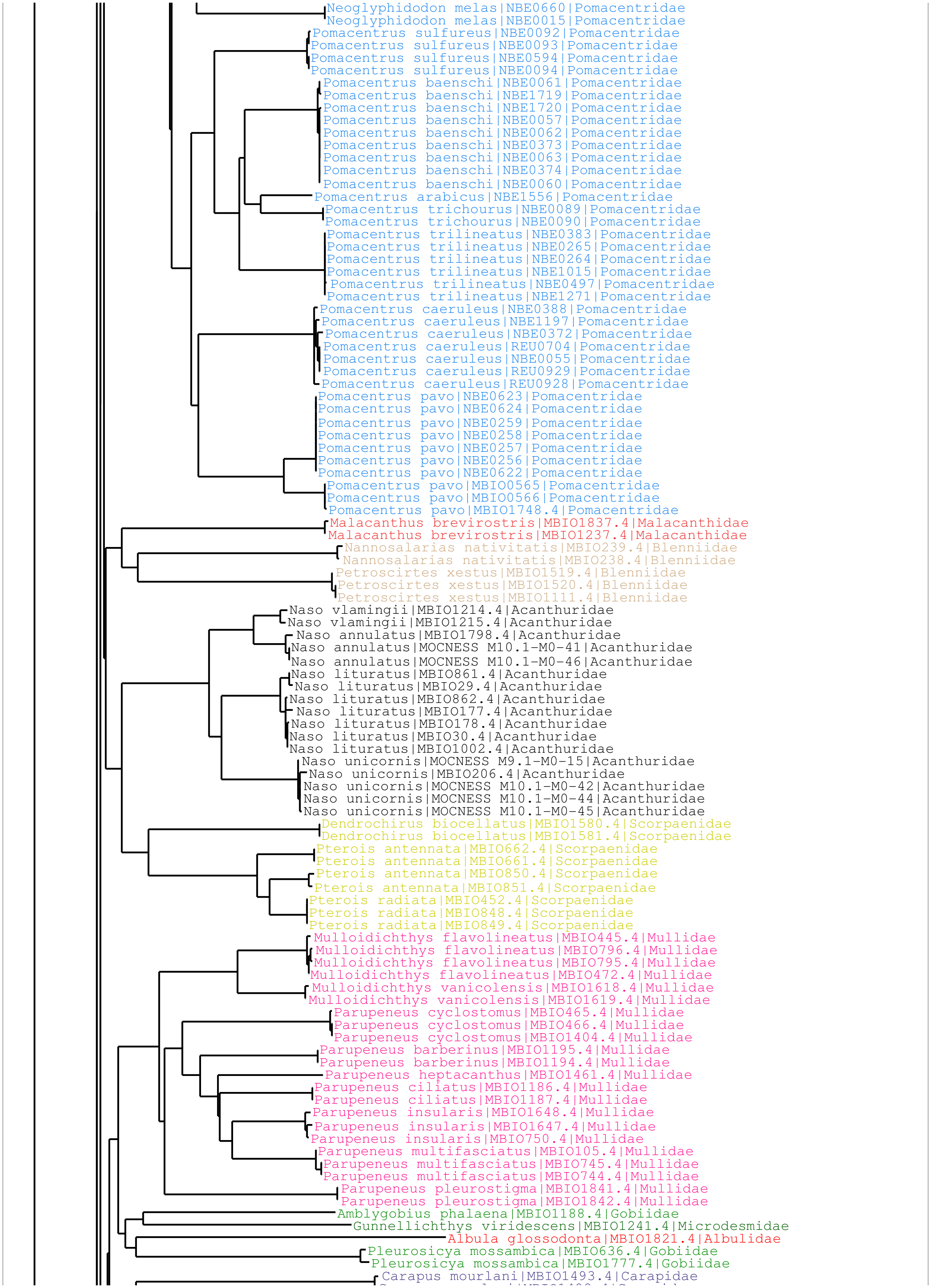
Neoglyphidodon melas|NBE0660|Pomacentridae
Neoglyphidodon melas|NBE0015|Pomacentridae
Pomacentrus sulfureus|NBE0092|Pomacentridae
Pomacentrus sulfureus|NBE0093|Pomacentridae
Pomacentrus sulfureus|NBE0594|Pomacentridae
Pomacentrus sulfureus|NBE0094|Pomacentridae
Pomacentrus baenschi|NBE0061|Pomacentridae
Pomacentrus baenschi|NBE1719|Pomacentridae
Pomacentrus baenschi|NBE1720|Pomacentridae
Pomacentrus baenschi|NBE0057|Pomacentridae
Pomacentrus baenschi|NBE0062|Pomacentridae
Pomacentrus baenschi|NBE0373|Pomacentridae
Pomacentrus baenschi|NBE0063|Pomacentridae
Pomacentrus baenschi|NBE0374|Pomacentridae
Pomacentrus baenschi|NBE0060|Pomacentridae
Pomacentrus arabicus|NBE1556|Pomacentridae
Pomacentrus trichourus|NBE0089|Pomacentridae
Pomacentrus trichourus|NBE0090|Pomacentridae
Pomacentrus trilineatus|NBE0383|Pomacentridae
Pomacentrus trilineatus|NBE0265|Pomacentridae
Pomacentrus trilineatus|NBE0264|Pomacentridae
Pomacentrus trilineatus|NBE1015|Pomacentridae
Pomacentrus trilineatus|NBE0497|Pomacentridae
Pomacentrus trilineatus|NBE1271|Pomacentridae
Pomacentrus caeruleus|NBE0388|Pomacentridae
Pomacentrus caeruleus|NBE1197|Pomacentridae
Pomacentrus caeruleus|NBE0372|Pomacentridae
Pomacentrus caeruleus|REU0704|Pomacentridae
Pomacentrus caeruleus|NBE0055|Pomacentridae
Pomacentrus caeruleus|REU0929|Pomacentridae
Pomacentrus caeruleus|REU0928|Pomacentridae
Pomacentrus pavo|NBE0623|Pomacentridae
Pomacentrus pavo|NBE0624|Pomacentridae
Pomacentrus pavo|NBE0259|Pomacentridae
Pomacentrus pavo|NBE0258|Pomacentridae
Pomacentrus pavo|NBE0257|Pomacentridae
Pomacentrus pavo|NBE0256|Pomacentridae
Pomacentrus pavo|NBE0622|Pomacentridae
Pomacentrus pavo|MBIO0565|Pomacentridae
Pomacentrus pavo|MBIO0566|Pomacentridae
Pomacentrus pavo|MBIO1748.4|Pomacentridae
Malacanthus brevirostris|MBIO1837.4|Malacanthidae
Malacanthus brevirostris|MBIO1237.4|Malacanthidae
Nannosalarias nativitatis|MBIO239.4|Blenniidae
Nannosalarias nativitatis|MBIO238.4|Blenniidae
Petroscirtes xestus|MBIO1519.4|Blenniidae
Petroscirtes xestus|MBIO1520.4|Blenniidae
Petroscirtes xestus|MBIO1111.4|Blenniidae
Naso vlamingii|MBIO1214.4|Acanthuridae
Naso vlamingii|MBIO1215.4|Acanthuridae
Naso annulatus|MBIO1798.4|Acanthuridae
Naso annulatus|MOCNESS M10.1-M0-41|Acanthuridae
Naso annulatus|MOCNESS M10.1-M0-46|Acanthuridae
Naso lituratus|MBIO861.4|Acanthuridae
Naso lituratus|MBIO29.4|Acanthuridae
Naso lituratus|MBIO862.4|Acanthuridae
Naso lituratus|MBIO177.4|Acanthuridae
Naso lituratus|MBIO178.4|Acanthuridae
Naso lituratus|MBIO30.4|Acanthuridae
Naso lituratus|MBIO1002.4|Acanthuridae
Naso unicornis|MOCNESS M9.1-M0-15|Acanthuridae
Naso unicornis|MBIO206.4|Acanthuridae
Naso unicornis|MOCNESS M10.1-M0-42|Acanthuridae
Naso unicornis|MOCNESS M10.1-M0-44|Acanthuridae
Naso unicornis|MOCNESS M10.1-M0-45|Acanthuridae
Dendrochirus biocellatus|MBIO1580.4|Scorpaenidae
Dendrochirus biocellatus|MBIO1581.4|Scorpaenidae
Pterois antennata|MBIO662.4|Scorpaenidae
Pterois antennata|MBIO661.4|Scorpaenidae
Pterois antennata|MBIO850.4|Scorpaenidae
Pterois antennata|MBIO851.4|Scorpaenidae
Pterois radiata|MBIO452.4|Scorpaenidae
Pterois radiata|MBIO848.4|Scorpaenidae
Pterois radiata|MBIO849.4|Scorpaenidae
Mulloidichthys flavolineatus|MBIO445.4|Mullidae
Mulloidichthys flavolineatus|MBIO796.4|Mullidae
Mulloidichthys flavolineatus|MBIO795.4|Mullidae
Mulloidichthys flavolineatus|MBIO472.4|Mullidae
Mulloidichthys vanicolensis|MBIO1618.4|Mullidae
Mulloidichthys vanicolensis|MBIO1619.4|Mullidae
Parupeneus cyclostomus|MBIO465.4|Mullidae
Parupeneus cyclostomus|MBIO466.4|Mullidae
Parupeneus cyclostomus|MBIO1404.4|Mullidae
Parupeneus barberinus|MBIO1195.4|Mullidae
Parupeneus barberinus|MBIO1194.4|Mullidae
Parupeneus heptacanthus|MBIO1461.4|Mullidae
Parupeneus ciliatus|MBIO1186.4|Mullidae
Parupeneus ciliatus|MBIO1187.4|Mullidae
Parupeneus insularis|MBIO1648.4|Mullidae
Parupeneus insularis|MBIO1647.4|Mullidae
Parupeneus insularis|MBIO750.4|Mullidae
Parupeneus multifasciatus|MBIO105.4|Mullidae
Parupeneus multifasciatus|MBIO745.4|Mullidae
Parupeneus multifasciatus|MBIO744.4|Mullidae
Parupeneus pleurostigma|MBIO1841.4|Mullidae
Parupeneus pleurostigma|MBIO1842.4|Mullidae
Amblygobius phalaena|MBIO1188.4|Gobiidae
Gunnellichthys viridescens|MBIO1241.4|Microdesmidae
Albula glossodonta|MBIO1821.4|Albulidae
Pleurosicya mossambica|MBIO636.4|Gobiidae
Pleurosicya mossambica|MBIO1777.4|Gobiidae
Carapus mourlani|MBIO1493.4|Carapidae

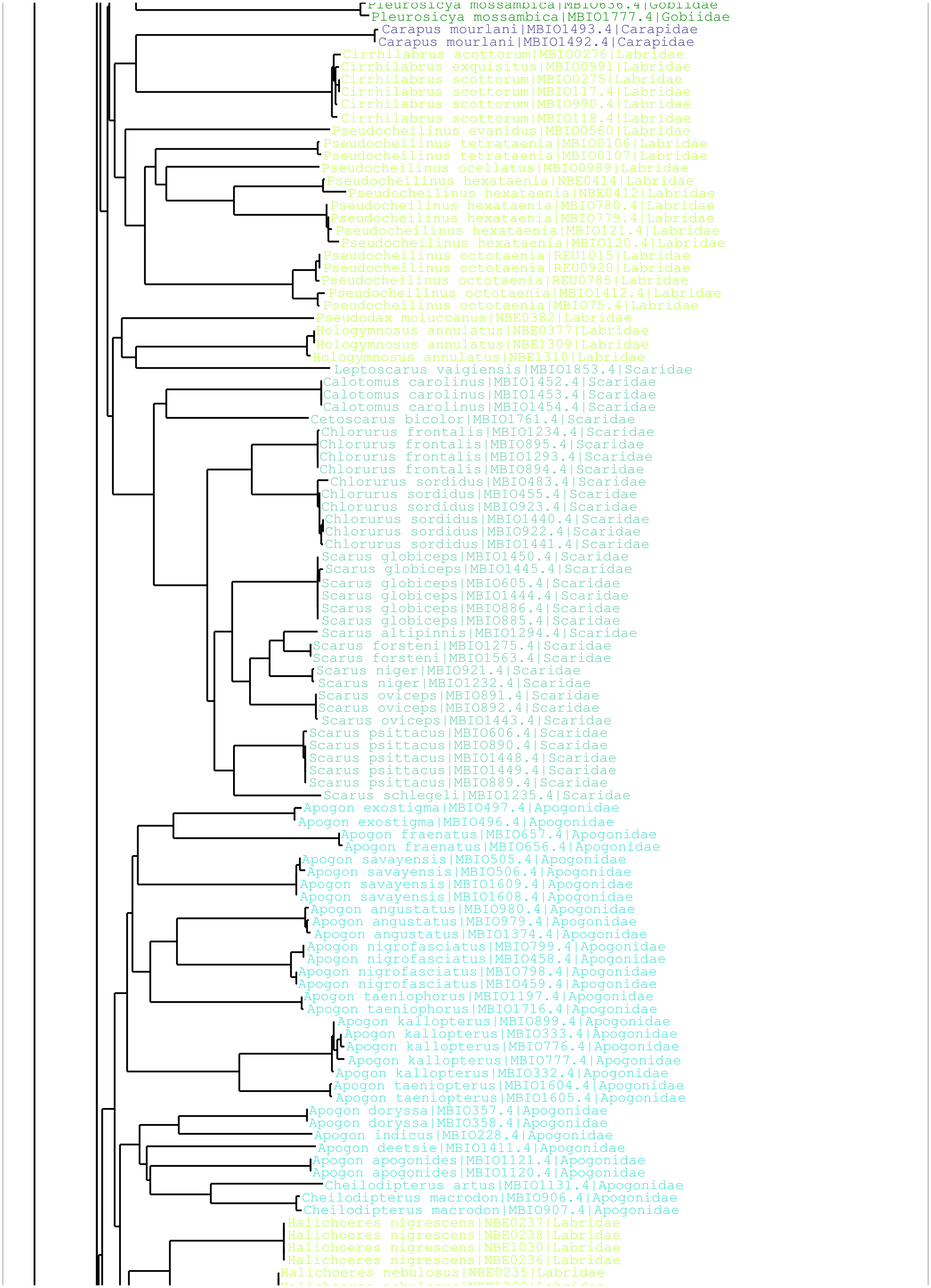
Pleurosicya mossambica|MBIO636.4|Gobiidae
Pleurosicya mossambica|MBIO1777.4|Gobiidae
Carapus mourlani|MBIO1493.4|Carapidae
Carapus mourlani|MBIO1492.4|Carapidae
Cirrhilabrus scottorum|MBIO0276|Labridae
Cirrhilabrus exquisitus|MBIO0991|Labridae
Cirrhilabrus scottorum|MBIO0275|Labridae
Cirrhilabrus scottorum|MBIO117.4|Labridae
Cirrhilabrus scottorum|MBIO990.4|Labridae
Cirrhilabrus scottorum|MBIO118.4|Labridae
Pseudocheilinus evanidus|MBIO0560|Labridae
Pseudocheilinus tetrataenia|MBIO0106|Labridae
Pseudocheilinus tetrataenia|MBIO0107|Labridae
Pseudocheilinus ocellatus|MBIO0989|Labridae
Pseudocheilinus hexataenia|NBE0414|Labridae
Pseudocheilinus hexataenia|NBE0412|Labridae
Pseudocheilinus hexataenia|MBIO780.4|Labridae
Pseudocheilinus hexataenia|MBIO779.4|Labridae
Pseudocheilinus hexataenia|MBIO121.4|Labridae
Pseudocheilinus hexataenia|MBIO120.4|Labridae
Pseudocheilinus octotaenia|REU1015|Labridae
Pseudocheilinus octotaenia|REU0920|Labridae
Pseudocheilinus octotaenia|REU0785|Labridae
Pseudocheilinus octotaenia|MBIO1412.4|Labridae
Pseudocheilinus octotaenia|MBIO75.4|Labridae
Pseudodax moluccanus|NBE0382|Labridae
Hologymnosus annulatus|NBE0377|Labridae
Hologymnosus annulatus|NBE1309|Labridae
Hologymnosus annulatus|NBE1310|Labridae
Leptoscarus vaigiensis|MBIO1853.4|Scaridae
Calotomus carolinus|MBIO1452.4|Scaridae
Calotomus carolinus|MBIO1453.4|Scaridae
Calotomus carolinus|MBIO1454.4|Scaridae
Cetoscarus bicolor|MBIO1761.4|Scaridae
Chlorurus frontalis|MBIO1234.4|Scaridae
Chlorurus frontalis|MBIO895.4|Scaridae
Chlorurus frontalis|MBIO1293.4|Scaridae
Chlorurus frontalis|MBIO894.4|Scaridae
Chlorurus sordidus|MBIO483.4|Scaridae
Chlorurus sordidus|MBIO455.4|Scaridae
Chlorurus sordidus|MBIO923.4|Scaridae
Chlorurus sordidus|MBIO1440.4|Scaridae
Chlorurus sordidus|MBIO922.4|Scaridae
Chlorurus sordidus|MBIO1441.4|Scaridae
Scarus globiceps|MBIO1450.4|Scaridae
Scarus globiceps|MBIO1445.4|Scaridae
Scarus globiceps|MBIO605.4|Scaridae
Scarus globiceps|MBIO1444.4|Scaridae
Scarus globiceps|MBIO886.4|Scaridae
Scarus globiceps|MBIO885.4|Scaridae
Scarus altipinnis|MBIO1294.4|Scaridae
Scarus forsteni|MBIO1275.4|Scaridae
Scarus forsteni|MBIO1563.4|Scaridae
Scarus niger|MBIO921.4|Scaridae
Scarus niger|MBIO1232.4|Scaridae
Scarus oviceps|MBIO891.4|Scaridae
Scarus oviceps|MBIO892.4|Scaridae
Scarus oviceps|MBIO1443.4|Scaridae
Scarus psittacus|MBIO606.4|Scaridae
Scarus psittacus|MBIO890.4|Scaridae
Scarus psittacus|MBIO1448.4|Scaridae
Scarus psittacus|MBIO1449.4|Scaridae
Scarus psittacus|MBIO889.4|Scaridae
Scarus schlegeli|MBIO1235.4|Scaridae
Apogon exostigma|MBIO497.4|Apogonidae
Apogon exostigma|MBIO496.4|Apogonidae
Apogon fraenatus|MBIO657.4|Apogonidae
Apogon fraenatus|MBIO656.4|Apogonidae
Apogon savayensis|MBIO505.4|Apogonidae
Apogon savayensis|MBIO506.4|Apogonidae
Apogon savayensis|MBIO1609.4|Apogonidae
Apogon savayensis|MBIO1608.4|Apogonidae
Apogon angustatus|MBIO980.4|Apogonidae
Apogon angustatus|MBIO979.4|Apogonidae
Apogon angustatus|MBIO1374.4|Apogonidae
Apogon nigrofasciatus|MBIO799.4|Apogonidae
Apogon nigrofasciatus|MBIO458.4|Apogonidae
Apogon nigrofasciatus|MBIO798.4|Apogonidae
Apogon nigrofasciatus|MBIO459.4|Apogonidae
Apogon taeniophorus|MBIO1197.4|Apogonidae
Apogon taeniophorus|MBIO1716.4|Apogonidae
Apogon kallopterus|MBIO899.4|Apogonidae
Apogon kallopterus|MBIO333.4|Apogonidae
Apogon kallopterus|MBIO776.4|Apogonidae
Apogon kallopterus|MBIO777.4|Apogonidae
Apogon kallopterus|MBIO332.4|Apogonidae
Apogon taeniopterus|MBIO1604.4|Apogonidae
Apogon taeniopterus|MBIO1605.4|Apogonidae
Apogon doryssa|MBIO357.4|Apogonidae
Apogon doryssa|MBIO358.4|Apogonidae
Apogon indicus|MBIO228.4|Apogonidae
Apogon deetsie|MBIO1411.4|Apogonidae
Apogon apogonides|MBIO1121.4|Apogonidae
Apogon apogonides|MBIO1120.4|Apogonidae
Cheilodipterus artus|MBIO1131.4|Apogonidae
Cheilodipterus macrodon|MBIO906.4|Apogonidae
Cheilodipterus macrodon|MBIO907.4|Apogonidae
Halichoeres nigrescens|NBE0237|Labridae
Halichoeres nigrescens|NBE0238|Labridae
Halichoeres nigrescens|NBE1030|Labridae
Halichoeres nigrescens|NBE0236|Labridae
Halichoeres nebulosus|NBE0235|Labridae

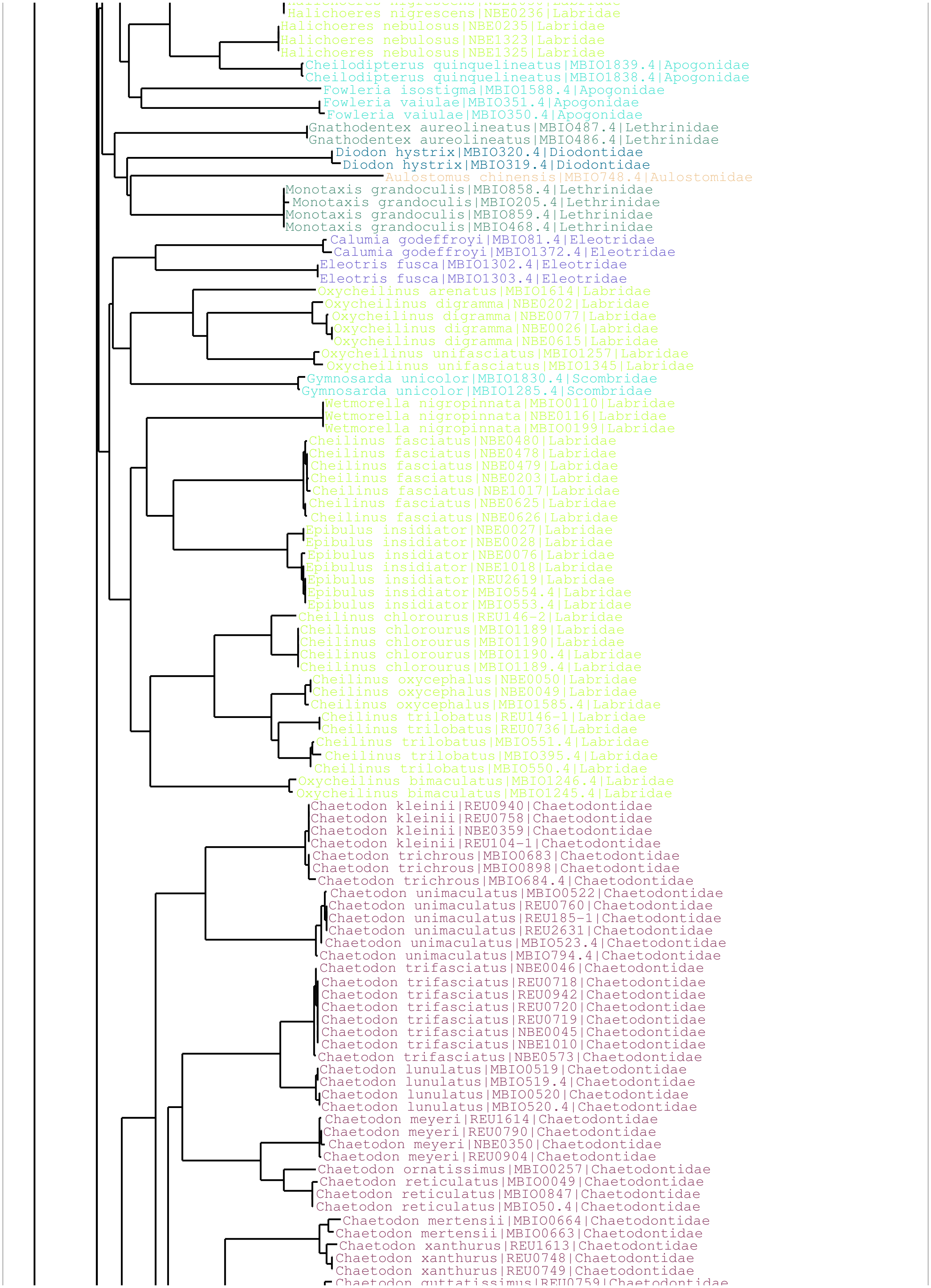
Halichoeres nigrescens|NBE0236|Labridae
Halichoeres nebulosus|NBE0235|Labridae
Halichoeres nebulosus|NBE1323|Labridae
Halichoeres nebulosus|NBE1325|Labridae
Cheilodipterus quinquelineatus|MBIO1839.4|Apogonidae
Cheilodipterus quinquelineatus|MBIO1838.4|Apogonidae
Fowleria isostigma|MBIO1588.4|Apogonidae
Fowleria vaiulae|MBIO351.4|Apogonidae
Fowleria vaiulae|MBIO350.4|Apogonidae
Gnathodentex aureolineatus|MBIO487.4|Lethrinidae
Gnathodentex aureolineatus|MBIO486.4|Lethrinidae
Diodon hystrix|MBIO320.4|Diodontidae
Diodon hystrix|MBIO319.4|Diodontidae
Aulostomus chinensis|MBIO748.4|Aulostomidae
Monotaxis grandoculis|MBIO858.4|Lethrinidae
Monotaxis grandoculis|MBIO205.4|Lethrinidae
Monotaxis grandoculis|MBIO859.4|Lethrinidae
Monotaxis grandoculis|MBIO468.4|Lethrinidae
Calumia godeffroyi|MBIO81.4|Eleotridae
Calumia godeffroyi|MBIO1372.4|Eleotridae
Eleotris fusca|MBIO1302.4|Eleotridae
Eleotris fusca|MBIO1303.4|Eleotridae
Oxycheilinus arenatus|MBIO1614|Labridae
Oxycheilinus digramma|NBE0202|Labridae
Oxycheilinus digramma|NBE0077|Labridae
Oxycheilinus digramma|NBE0026|Labridae
Oxycheilinus digramma|NBE0615|Labridae
Oxycheilinus unifasciatus|MBIO1257|Labridae
Oxycheilinus unifasciatus|MBIO1345|Labridae
Gymnosarda unicolor|MBIO1830.4|Scombridae
Gymnosarda unicolor|MBIO1285.4|Scombridae
Wetmorella nigropinnata|MBIO0110|Labridae
Wetmorella nigropinnata|NBE0116|Labridae
Wetmorella nigropinnata|MBIO0199|Labridae
Cheilinus fasciatus|NBE0480|Labridae
Cheilinus fasciatus|NBE0478|Labridae
Cheilinus fasciatus|NBE0479|Labridae
Cheilinus fasciatus|NBE0203|Labridae
Cheilinus fasciatus|NBE1017|Labridae
Cheilinus fasciatus|NBE0625|Labridae
Cheilinus fasciatus|NBE0626|Labridae
Epibulus insidiator|NBE0027|Labridae
Epibulus insidiator|NBE0028|Labridae
Epibulus insidiator|NBE0076|Labridae
Epibulus insidiator|NBE1018|Labridae
Epibulus insidiator|REU2619|Labridae
Epibulus insidiator|MBIO554.4|Labridae
Epibulus insidiator|MBIO553.4|Labridae
Cheilinus chlorourus|REU146-2|Labridae
Cheilinus chlorourus|MBIO1189|Labridae
Cheilinus chlorourus|MBIO1190|Labridae
Cheilinus chlorourus|MBIO1190.4|Labridae
Cheilinus chlorourus|MBIO1189.4|Labridae
Cheilinus oxycephalus|NBE0050|Labridae
Cheilinus oxycephalus|NBE0049|Labridae
Cheilinus oxycephalus|MBIO1585.4|Labridae
Cheilinus trilobatus|REU146-1|Labridae
Cheilinus trilobatus|REU0736|Labridae
Cheilinus trilobatus|MBIO551.4|Labridae
Cheilinus trilobatus|MBIO395.4|Labridae
Cheilinus trilobatus|MBIO550.4|Labridae
Oxycheilinus bimaculatus|MBIO1246.4|Labridae
Oxycheilinus bimaculatus|MBIO1245.4|Labridae
Chaetodon kleinii|REU0940|Chaetodontidae
Chaetodon kleinii|REU0758|Chaetodontidae
Chaetodon kleinii|NBE0359|Chaetodontidae
Chaetodon kleinii|REU104-1|Chaetodontidae
Chaetodon trichrous|MBIO0683|Chaetodontidae
Chaetodon trichrous|MBIO0898|Chaetodontidae
Chaetodon trichrous|MBIO684.4|Chaetodontidae
Chaetodon unimaculatus|MBIO0522|Chaetodontidae
Chaetodon unimaculatus|REU0760|Chaetodontidae
Chaetodon unimaculatus|REU185-1|Chaetodontidae
Chaetodon unimaculatus|REU2631|Chaetodontidae
Chaetodon unimaculatus|MBIO523.4|Chaetodontidae
Chaetodon unimaculatus|MBIO794.4|Chaetodontidae
Chaetodon trifasciatus|NBE0046|Chaetodontidae
Chaetodon trifasciatus|REU0718|Chaetodontidae
Chaetodon trifasciatus|REU0942|Chaetodontidae
Chaetodon trifasciatus|REU0720|Chaetodontidae
Chaetodon trifasciatus|REU0719|Chaetodontidae
Chaetodon trifasciatus|NBE0045|Chaetodontidae
Chaetodon trifasciatus|NBE1010|Chaetodontidae
Chaetodon trifasciatus|NBE0573|Chaetodontidae
Chaetodon lunulatus|MBIO0519|Chaetodontidae
Chaetodon lunulatus|MBIO519.4|Chaetodontidae
Chaetodon lunulatus|MBIO0520|Chaetodontidae
Chaetodon lunulatus|MBIO520.4|Chaetodontidae
Chaetodon meyeri|REU1614|Chaetodontidae
Chaetodon meyeri|REU0790|Chaetodontidae
Chaetodon meyeri|NBE0350|Chaetodontidae
Chaetodon meyeri|REU0904|Chaetodontidae
Chaetodon ornatissimus|MBIO0257|Chaetodontidae
Chaetodon reticulatus|MBIO0049|Chaetodontidae
Chaetodon reticulatus|MBIO0847|Chaetodontidae
Chaetodon reticulatus|MBIO50.4|Chaetodontidae
Chaetodon mertensii|MBIO0664|Chaetodontidae
Chaetodon mertensii|MBIO0663|Chaetodontidae
Chaetodon xanthurus|REU1613|Chaetodontidae
Chaetodon xanthurus|REU0748|Chaetodontidae
Chaetodon xanthurus|REU0749|Chaetodontidae
Chaetodon guttatissimus|REU0759|Chaetodontidae

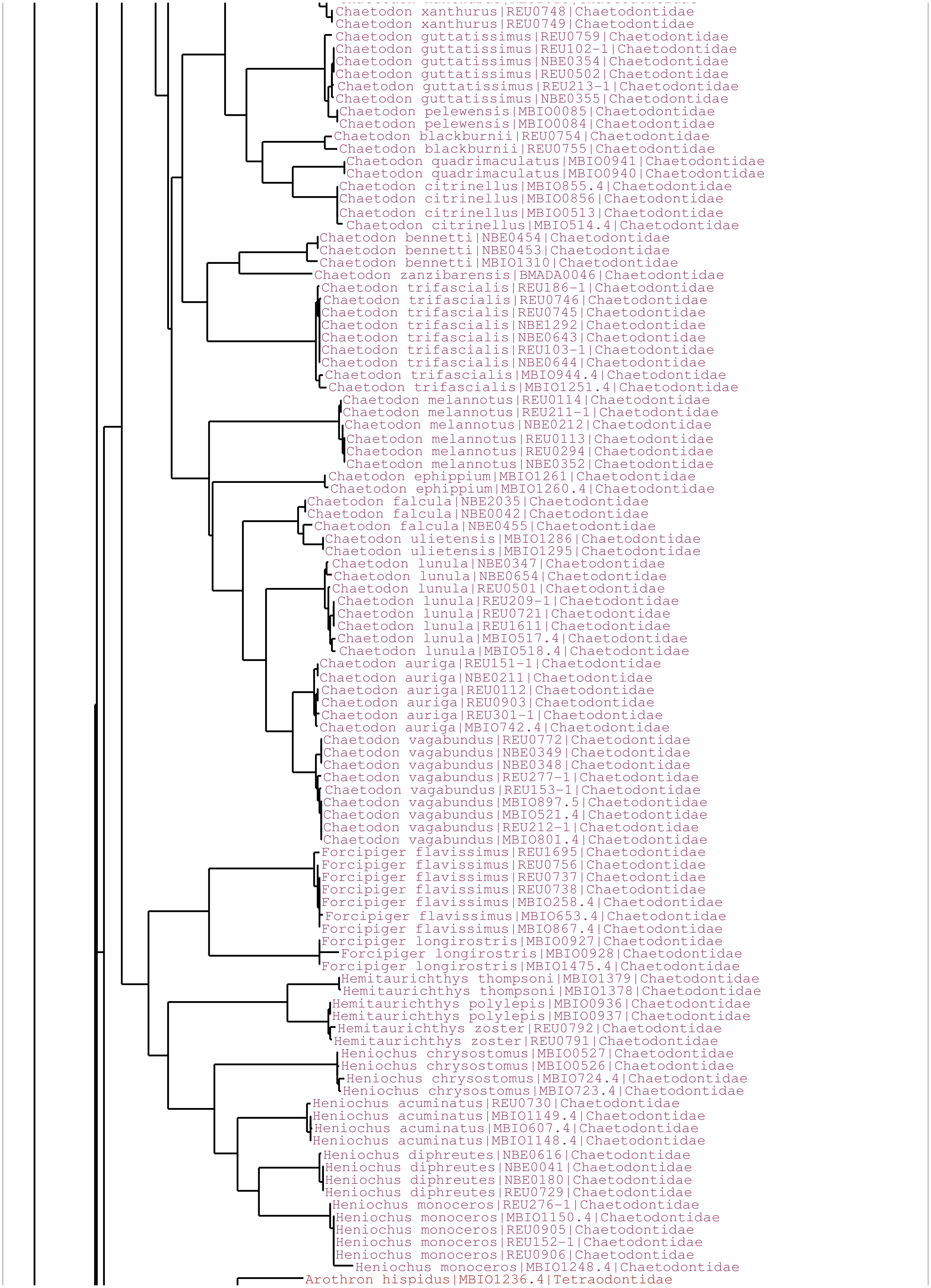

Chaetodon xanthurus|REU0748|Chaetodontidae
Chaetodon xanthurus|REU0749|Chaetodontidae
Chaetodon guttatissimus|REU0759|Chaetodontidae
Chaetodon guttatissimus|REU102-1|Chaetodontidae
Chaetodon guttatissimus|NBE0354|Chaetodontidae
Chaetodon guttatissimus|REU0502|Chaetodontidae
Chaetodon guttatissimus|REU213-1|Chaetodontidae
Chaetodon guttatissimus|NBE0355|Chaetodontidae
Chaetodon pelewensis|MBIO0085|Chaetodontidae
Chaetodon pelewensis|MBIO0084|Chaetodontidae
Chaetodon blackburnii|REU0754|Chaetodontidae
Chaetodon blackburnii|REU0755|Chaetodontidae
Chaetodon quadrimaculatus|MBIO0941|Chaetodontidae
Chaetodon quadrimaculatus|MBIO0940|Chaetodontidae
Chaetodon citrinellus|MBIO855.4|Chaetodontidae
Chaetodon citrinellus|MBIO0856|Chaetodontidae
Chaetodon citrinellus|MBIO0513|Chaetodontidae
Chaetodon citrinellus|MBIO514.4|Chaetodontidae
Chaetodon bennetti|NBE0454|Chaetodontidae
Chaetodon bennetti|NBE0453|Chaetodontidae
Chaetodon bennetti|MBIO1310|Chaetodontidae
Chaetodon zanzibarensis|BMADA0046|Chaetodontidae
Chaetodon trifascialis|REU186-1|Chaetodontidae
Chaetodon trifascialis|REU0746|Chaetodontidae
Chaetodon trifascialis|REU0745|Chaetodontidae
Chaetodon trifascialis|NBE1292|Chaetodontidae
Chaetodon trifascialis|NBE0643|Chaetodontidae
Chaetodon trifascialis|REU103-1|Chaetodontidae
Chaetodon trifascialis|NBE0644|Chaetodontidae
Chaetodon trifascialis|MBIO944.4|Chaetodontidae
Chaetodon trifascialis|MBIO1251.4|Chaetodontidae
Chaetodon melannotus|REU0114|Chaetodontidae
Chaetodon melannotus|REU211-1|Chaetodontidae
Chaetodon melannotus|NBE0212|Chaetodontidae
Chaetodon melannotus|REU0113|Chaetodontidae
Chaetodon melannotus|REU0294|Chaetodontidae
Chaetodon melannotus|NBE0352|Chaetodontidae
Chaetodon ephippium|MBIO1261|Chaetodontidae
Chaetodon ephippium|MBIO1260.4|Chaetodontidae
Chaetodon falcula|NBE2035|Chaetodontidae
Chaetodon falcula|NBE0042|Chaetodontidae
Chaetodon falcula|NBE0455|Chaetodontidae
Chaetodon ulietensis|MBIO1286|Chaetodontidae
Chaetodon ulietensis|MBIO1295|Chaetodontidae
Chaetodon lunula|NBE0347|Chaetodontidae
Chaetodon lunula|NBE0654|Chaetodontidae
Chaetodon lunula|REU0501|Chaetodontidae
Chaetodon lunula|REU209-1|Chaetodontidae
Chaetodon lunula|REU0721|Chaetodontidae
Chaetodon lunula|REU1611|Chaetodontidae
Chaetodon lunula|MBIO517.4|Chaetodontidae
Chaetodon lunula|MBIO518.4|Chaetodontidae
Chaetodon auriga|REU151-1|Chaetodontidae
Chaetodon auriga|NBE0211|Chaetodontidae
Chaetodon auriga|REU0112|Chaetodontidae
Chaetodon auriga|REU0903|Chaetodontidae
Chaetodon auriga|REU301-1|Chaetodontidae
Chaetodon auriga|MBIO742.4|Chaetodontidae
Chaetodon vagabundus|REU0772|Chaetodontidae
Chaetodon vagabundus|NBE0349|Chaetodontidae
Chaetodon vagabundus|NBE0348|Chaetodontidae
Chaetodon vagabundus|REU277-1|Chaetodontidae
Chaetodon vagabundus|REU153-1|Chaetodontidae
Chaetodon vagabundus|MBIO897.5|Chaetodontidae
Chaetodon vagabundus|MBIO521.4|Chaetodontidae
Chaetodon vagabundus|REU212-1|Chaetodontidae
Chaetodon vagabundus|MBIO801.4|Chaetodontidae
Forcipiger flavissimus|REU1695|Chaetodontidae
Forcipiger flavissimus|REU0756|Chaetodontidae
Forcipiger flavissimus|REU0737|Chaetodontidae
Forcipiger flavissimus|REU0738|Chaetodontidae
Forcipiger flavissimus|MBIO258.4|Chaetodontidae
Forcipiger flavissimus|MBIO653.4|Chaetodontidae
Forcipiger flavissimus|MBIO867.4|Chaetodontidae
Forcipiger longirostris|MBIO0927|Chaetodontidae
Forcipiger longirostris|MBIO0928|Chaetodontidae
Forcipiger longirostris|MBIO1475.4|Chaetodontidae
Hemitaurichthys thompsoni|MBIO1379|Chaetodontidae
Hemitaurichthys thompsoni|MBIO1378|Chaetodontidae
Hemitaurichthys polylepis|MBIO0936|Chaetodontidae
Hemitaurichthys polylepis|MBIO0937|Chaetodontidae
Hemitaurichthys zoster|REU0792|Chaetodontidae
Hemitaurichthys zoster|REU0791|Chaetodontidae
Heniochus chrysostomus|MBIO0527|Chaetodontidae
Heniochus chrysostomus|MBIO0526|Chaetodontidae
Heniochus chrysostomus|MBIO724.4|Chaetodontidae
Heniochus chrysostomus|MBIO723.4|Chaetodontidae
Heniochus acuminatus|REU0730|Chaetodontidae
Heniochus acuminatus|MBIO1149.4|Chaetodontidae
Heniochus acuminatus|MBIO607.4|Chaetodontidae
Heniochus acuminatus|MBIO1148.4|Chaetodontidae
Heniochus diphreutes|NBE0616|Chaetodontidae
Heniochus diphreutes|NBE0041|Chaetodontidae
Heniochus diphreutes|NBE0180|Chaetodontidae
Heniochus diphreutes|REU0729|Chaetodontidae
Heniochus monoceros|REU276-1|Chaetodontidae
Heniochus monoceros|MBIO1150.4|Chaetodontidae
Heniochus monoceros|REU0905|Chaetodontidae
Heniochus monoceros|REU152-1|Chaetodontidae
Heniochus monoceros|REU0906|Chaetodontidae
Heniochus monoceros|MBIO1248.4|Chaetodontidae
Arothron hispidus|MBIO1236.4|Tetraodontidae

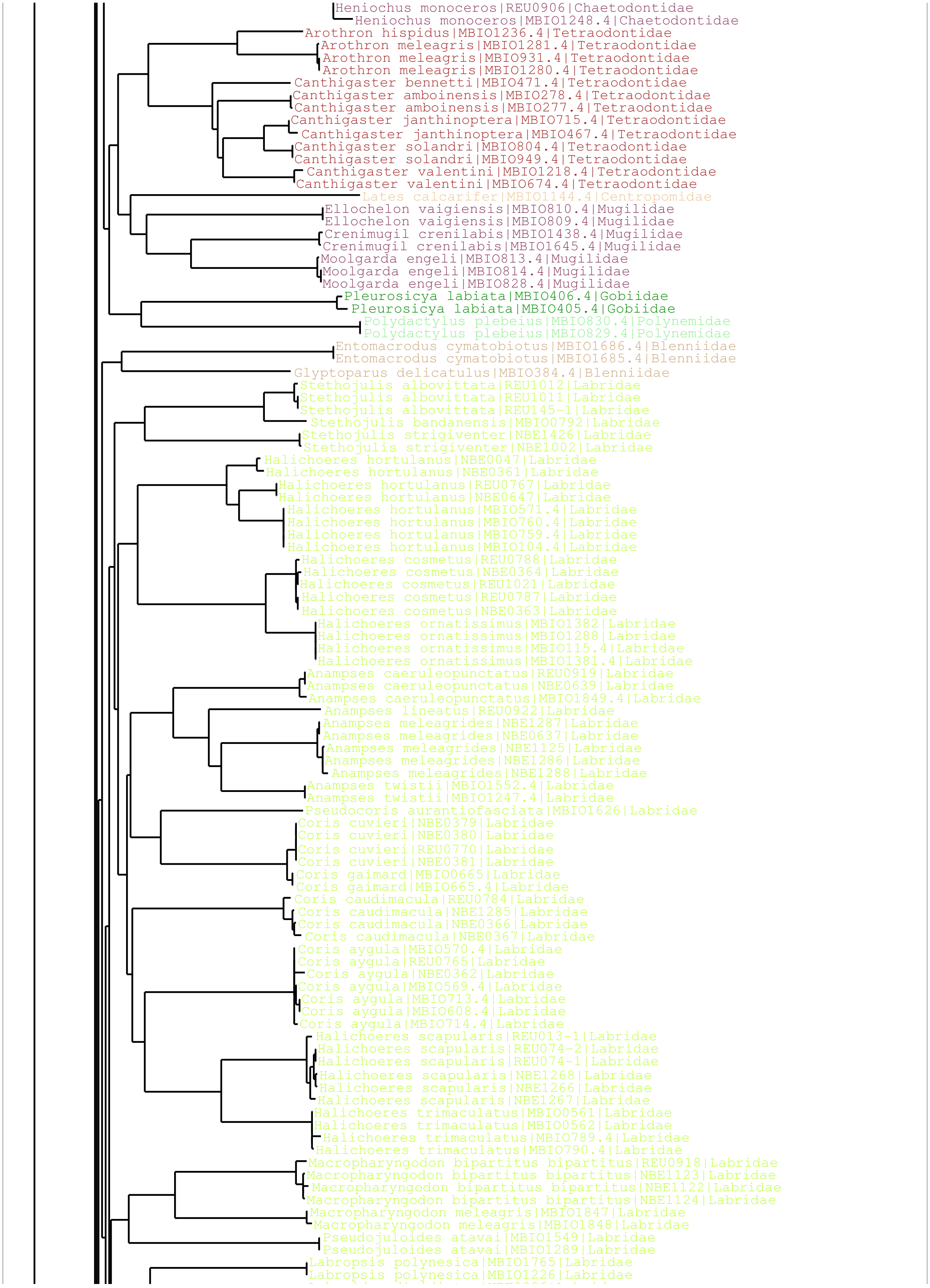
Heniochus monoceros|REU0906|Chaetodontidae
Heniochus monoceros|MBIO1248.4|Chaetodontidae
Arothron hispidus|MBIO1236.4|Tetraodontidae
Arothron meleagris|MBIO1281.4|Tetraodontidae
Arothron meleagris|MBIO931.4|Tetraodontidae
Arothron meleagris|MBIO1280.4|Tetraodontidae
Canthigaster bennetti|MBIO471.4|Tetraodontidae
Canthigaster amboinensis|MBIO278.4|Tetraodontidae
Canthigaster amboinensis|MBIO277.4|Tetraodontidae
Canthigaster janthinoptera|MBIO715.4|Tetraodontidae
Canthigaster janthinoptera|MBIO467.4|Tetraodontidae
Canthigaster solandri|MBIO804.4|Tetraodontidae
Canthigaster solandri|MBIO949.4|Tetraodontidae
Canthigaster valentini|MBIO1218.4|Tetraodontidae
Canthigaster valentini|MBIO674.4|Tetraodontidae
Lates calcarifer|MBIO1144.4|Centropomidae
Ellochelon vaigiensis|MBIO810.4|Mugilidae
Ellochelon vaigiensis|MBIO809.4|Mugilidae
Crenimugil crenilabis|MBIO1438.4|Mugilidae
Crenimugil crenilabis|MBIO1645.4|Mugilidae
Moolgarda engeli|MBIO813.4|Mugilidae
Moolgarda engeli|MBIO814.4|Mugilidae
Moolgarda engeli|MBIO828.4|Mugilidae
Pleurosicya labiata|MBIO406.4|Gobiidae
Pleurosicya labiata|MBIO405.4|Gobiidae
Polydactylus plebeius|MBIO830.4|Polynemidae
Polydactylus plebeius|MBIO829.4|Polynemidae
Entomacrodus cymatobiotus|MBIO1686.4|Blenniidae
Entomacrodus cymatobiotus|MBIO1685.4|Blenniidae
Glyptoparus delicatulus|MBIO384.4|Blenniidae
Stethojulis albovittata|REU1012|Labridae
Stethojulis albovittata|REU1011|Labridae
Stethojulis albovittata|REU145-1|Labridae
Stethojulis bandanensis|MBIO0792|Labridae
Stethojulis strigiventer|NBE1426|Labridae
Stethojulis strigiventer|NBE1002|Labridae
Halichoeres hortulanus|NBE0047|Labridae
Halichoeres hortulanus|NBE0361|Labridae
Halichoeres hortulanus|REU0767|Labridae
Halichoeres hortulanus|NBE0647|Labridae
Halichoeres hortulanus|MBIO571.4|Labridae
Halichoeres hortulanus|MBIO760.4|Labridae
Halichoeres hortulanus|MBIO759.4|Labridae
Halichoeres hortulanus|MBIO104.4|Labridae
Halichoeres cosmetus|REU0788|Labridae
Halichoeres cosmetus|NBE0364|Labridae
Halichoeres cosmetus|REU1021|Labridae
Halichoeres cosmetus|REU0787|Labridae
Halichoeres cosmetus|NBE0363|Labridae
Halichoeres ornatissimus|MBIO1382|Labridae
Halichoeres ornatissimus|MBIO1288|Labridae
Halichoeres ornatissimus|MBIO115.4|Labridae
Halichoeres ornatissimus|MBIO1381.4|Labridae
Anampses caeruleopunctatus|REU0919|Labridae
Anampses caeruleopunctatus|NBE0639|Labridae
Anampses caeruleopunctatus|MBIO1849.4|Labridae
Anampses lineatus|REU0922|Labridae
Anampses meleagrides|NBE1287|Labridae
Anampses meleagrides|NBE0637|Labridae
Anampses meleagrides|NBE1125|Labridae
Anampses meleagrides|NBE1286|Labridae
Anampses meleagrides|NBE1288|Labridae
Anampses twistii|MBIO1552.4|Labridae
Anampses twistii|MBIO1247.4|Labridae
Pseudocoris aurantiofasciata|MBIO1626|Labridae
Coris cuvieri|NBE0379|Labridae
Coris cuvieri|NBE0380|Labridae
Coris cuvieri|REU0770|Labridae
Coris cuvieri|NBE0381|Labridae
Coris gaimard|MBIO0665|Labridae
Coris gaimard|MBIO665.4|Labridae
Coris caudimacula|REU0784|Labridae
Coris caudimacula|NBE1285|Labridae
Coris caudimacula|NBE0366|Labridae
Coris caudimacula|NBE0367|Labridae
Coris aygula|MBIO570.4|Labridae
Coris aygula|REU0765|Labridae
Coris aygula|NBE0362|Labridae
Coris aygula|MBIO569.4|Labridae
Coris aygula|MBIO713.4|Labridae
Coris aygula|MBIO608.4|Labridae
Coris aygula|MBIO714.4|Labridae
Halichoeres scapularis|REU013-1|Labridae
Halichoeres scapularis|REU074-2|Labridae
Halichoeres scapularis|REU074-1|Labridae
Halichoeres scapularis|NBE1268|Labridae
Halichoeres scapularis|NBE1266|Labridae
Halichoeres scapularis|NBE1267|Labridae
Halichoeres trimaculatus|MBIO0561|Labridae
Halichoeres trimaculatus|MBIO0562|Labridae
Halichoeres trimaculatus|MBIO789.4|Labridae
Halichoeres trimaculatus|MBIO790.4|Labridae
Macropharyngodon bipartitus bipartitus|REU0918|Labridae
Macropharyngodon bipartitus bipartitus|NBE1123|Labridae
Macropharyngodon bipartitus bipartitus|NBE1122|Labridae
Macropharyngodon bipartitus bipartitus|NBE1124|Labridae
Macropharyngodon meleagris|MBIO1847|Labridae
Macropharyngodon meleagris|MBIO1848|Labridae
Pseudojuloides atavai|MBIO1549|Labridae
Pseudojuloides atavai|MBIO1289|Labridae
Labropsis polynesica|MBIO1765|Labridae
Labropsis polynesica|MBIO1226|Labridae

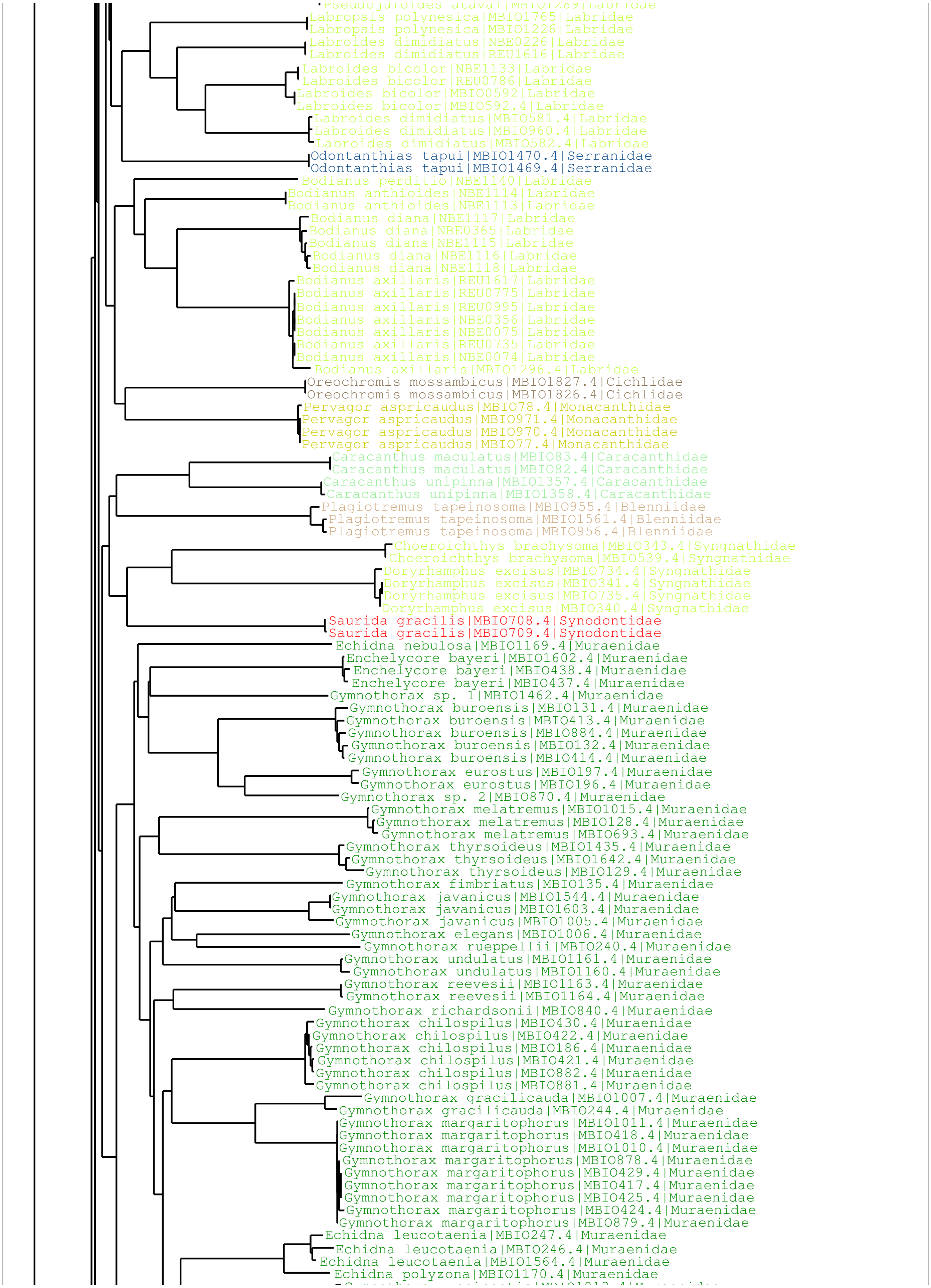
Pseudojuloides atavai|MBIO1289|Labridae
Labropsis polynesica|MBIO1765|Labridae
Labropsis polynesica|MBIO1226|Labridae
Labroides dimidiatus|NBE0226|Labridae
Labroides dimidiatus|REU1616|Labridae
Labroides bicolor|NBE1133|Labridae
Labroides bicolor|REU0786|Labridae
Labroides bicolor|MBIO0592|Labridae
Labroides bicolor|MBIO592.4|Labridae
Labroides dimidiatus|MBIO581.4|Labridae
Labroides dimidiatus|MBIO960.4|Labridae
Labroides dimidiatus|MBIO582.4|Labridae
Odontanthias tapui|MBIO1470.4|Serranidae
Odontanthias tapui|MBIO1469.4|Serranidae
Bodianus perditio|NBE1140|Labridae
Bodianus anthioides|NBE1114|Labridae
Bodianus anthioides|NBE1113|Labridae
Bodianus diana|NBE1117|Labridae
Bodianus diana|NBE0365|Labridae
Bodianus diana|NBE1115|Labridae
Bodianus diana|NBE1116|Labridae
Bodianus diana|NBE1118|Labridae
Bodianus axillaris|REU1617|Labridae
Bodianus axillaris|REU0775|Labridae
Bodianus axillaris|REU0995|Labridae
Bodianus axillaris|NBE0356|Labridae
Bodianus axillaris|NBE0075|Labridae
Bodianus axillaris|REU0735|Labridae
Bodianus axillaris|NBE0074|Labridae
Bodianus axillaris|MBIO1296.4|Labridae
Oreochromis mossambicus|MBIO1827.4|Cichlidae
Oreochromis mossambicus|MBIO1826.4|Cichlidae
Pervagor aspricaudus|MBIO78.4|Monacanthidae
Pervagor aspricaudus|MBIO971.4|Monacanthidae
Pervagor aspricaudus|MBIO970.4|Monacanthidae
Pervagor aspricaudus|MBIO77.4|Monacanthidae
Caracanthus maculatus|MBIO83.4|Caracanthidae
Caracanthus maculatus|MBIO82.4|Caracanthidae
Caracanthus unipinna|MBIO1357.4|Caracanthidae
Caracanthus unipinna|MBIO1358.4|Caracanthidae
Plagiotremus tapeinosoma|MBIO955.4|Blenniidae
Plagiotremus tapeinosoma|MBIO1561.4|Blenniidae
Plagiotremus tapeinosoma|MBIO956.4|Blenniidae
Choeroichthys brachysoma|MBIO343.4|Syngnathidae
Choeroichthys brachysoma|MBIO539.4|Syngnathidae
Doryrhamphus excisus|MBIO734.4|Syngnathidae
Doryrhamphus excisus|MBIO341.4|Syngnathidae
Doryrhamphus excisus|MBIO735.4|Syngnathidae
Doryrhamphus excisus|MBIO340.4|Syngnathidae
Saurida gracilis|MBIO708.4|Synodontidae
Saurida gracilis|MBIO709.4|Synodontidae
Echidna nebulosa|MBIO1169.4|Muraenidae
Enchelycore bayeri|MBIO1602.4|Muraenidae
Enchelycore bayeri|MBIO438.4|Muraenidae
Enchelycore bayeri|MBIO437.4|Muraenidae
Gymnothorax sp. 1|MBIO1462.4|Muraenidae
Gymnothorax buroensis|MBIO131.4|Muraenidae
Gymnothorax buroensis|MBIO413.4|Muraenidae
Gymnothorax buroensis|MBIO884.4|Muraenidae
Gymnothorax buroensis|MBIO132.4|Muraenidae
Gymnothorax buroensis|MBIO414.4|Muraenidae
Gymnothorax eurostus|MBIO197.4|Muraenidae
Gymnothorax eurostus|MBIO196.4|Muraenidae
Gymnothorax sp. 2|MBIO870.4|Muraenidae
Gymnothorax melatremus|MBIO1015.4|Muraenidae
Gymnothorax melatremus|MBIO128.4|Muraenidae
Gymnothorax melatremus|MBIO693.4|Muraenidae
Gymnothorax thyrsoideus|MBIO1435.4|Muraenidae
Gymnothorax thyrsoideus|MBIO1642.4|Muraenidae
Gymnothorax thyrsoideus|MBIO129.4|Muraenidae
Gymnothorax fimbriatus|MBIO135.4|Muraenidae
Gymnothorax javanicus|MBIO1544.4|Muraenidae
Gymnothorax javanicus|MBIO1603.4|Muraenidae
Gymnothorax javanicus|MBIO1005.4|Muraenidae
Gymnothorax elegans|MBIO1006.4|Muraenidae
Gymnothorax rueppellii|MBIO240.4|Muraenidae
Gymnothorax undulatus|MBIO1161.4|Muraenidae
Gymnothorax undulatus|MBIO1160.4|Muraenidae
Gymnothorax reevesii|MBIO1163.4|Muraenidae
Gymnothorax reevesii|MBIO1164.4|Muraenidae
Gymnothorax richardsonii|MBIO840.4|Muraenidae
Gymnothorax chilospilus|MBIO430.4|Muraenidae
Gymnothorax chilospilus|MBIO422.4|Muraenidae
Gymnothorax chilospilus|MBIO186.4|Muraenidae
Gymnothorax chilospilus|MBIO421.4|Muraenidae
Gymnothorax chilospilus|MBIO882.4|Muraenidae
Gymnothorax chilospilus|MBIO881.4|Muraenidae
Gymnothorax gracilicauda|MBIO1007.4|Muraenidae
Gymnothorax gracilicauda|MBIO244.4|Muraenidae
Gymnothorax margaritophorus|MBIO1011.4|Muraenidae
Gymnothorax margaritophorus|MBIO418.4|Muraenidae
Gymnothorax margaritophorus|MBIO1010.4|Muraenidae
Gymnothorax margaritophorus|MBIO878.4|Muraenidae
Gymnothorax margaritophorus|MBIO429.4|Muraenidae
Gymnothorax margaritophorus|MBIO417.4|Muraenidae
Gymnothorax margaritophorus|MBIO425.4|Muraenidae
Gymnothorax margaritophorus|MBIO424.4|Muraenidae
Gymnothorax margaritophorus|MBIO879.4|Muraenidae
Echidna leucotaenia|MBIO247.4|Muraenidae
Echidna leucotaenia|MBIO246.4|Muraenidae
Echidna leucotaenia|MBIO1564.4|Muraenidae
Echidna polyzona|MBIO1170.4|Muraenidae

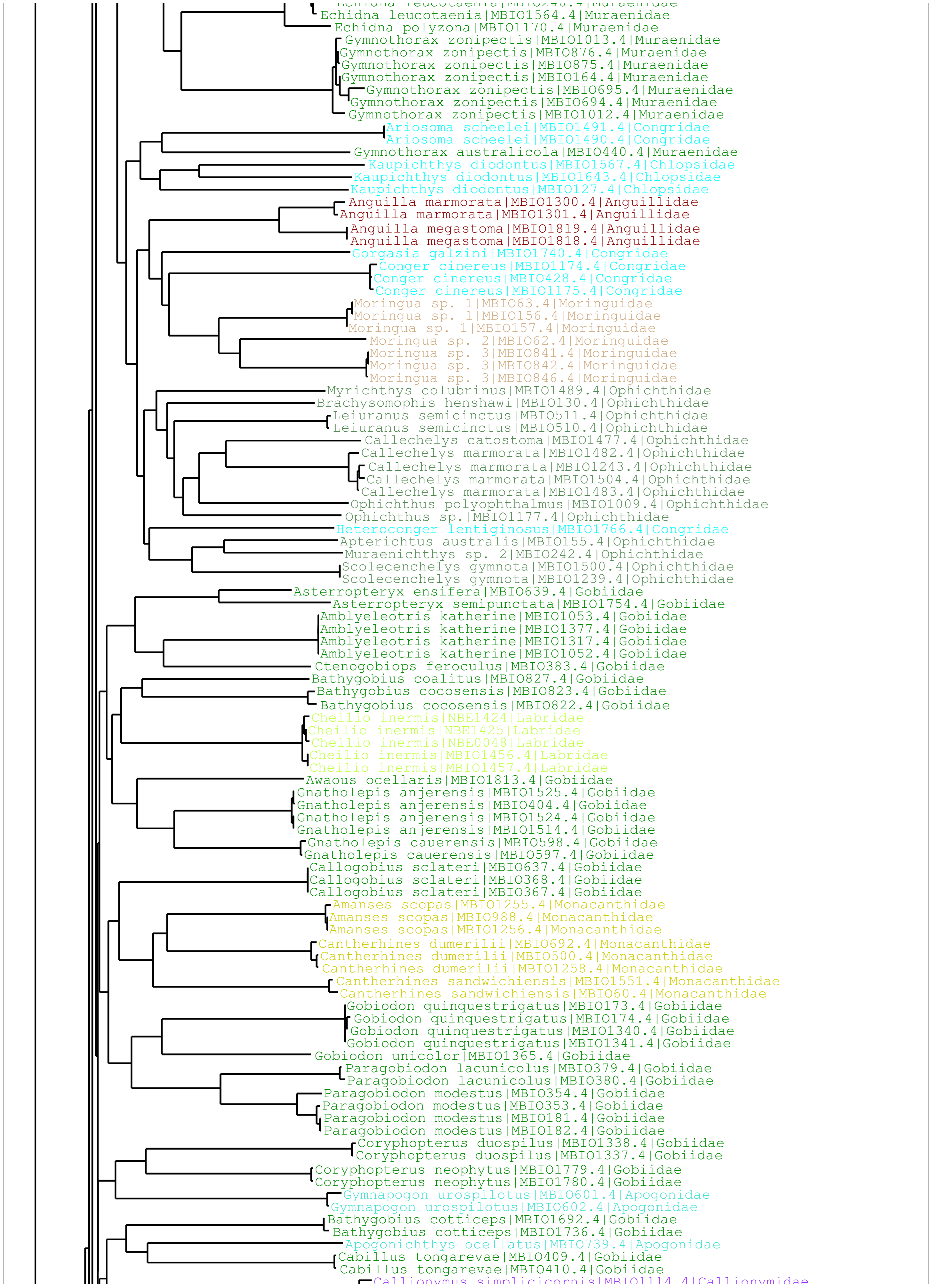
Echidna leucotaenia|MBIO1564.4|Muraenidae
Echidna polyzona|MBIO1170.4|Muraenidae
Gymnothorax zonipectis|MBIO1013.4|Muraenidae
Gymnothorax zonipectis|MBIO876.4|Muraenidae
Gymnothorax zonipectis|MBIO875.4|Muraenidae
Gymnothorax zonipectis|MBIO164.4|Muraenidae
Gymnothorax zonipectis|MBIO695.4|Muraenidae
Gymnothorax zonipectis|MBIO694.4|Muraenidae
Gymnothorax zonipectis|MBIO1012.4|Muraenidae
Ariosoma scheelei|MBIO1491.4|Congridae
Ariosoma scheelei|MBIO1490.4|Congridae
Gymnothorax australicola|MBIO440.4|Muraenidae
Kaupichthys diodontus|MBIO1567.4|Chlopsidae
Kaupichthys diodontus|MBIO1643.4|Chlopsidae
Kaupichthys diodontus|MBIO127.4|Chlopsidae
Anguilla marmorata|MBIO1300.4|Anguillidae
Anguilla marmorata|MBIO1301.4|Anguillidae
Anguilla megastoma|MBIO1819.4|Anguillidae
Anguilla megastoma|MBIO1818.4|Anguillidae
Gorgasia galzini|MBIO1740.4|Congridae
Conger cinereus|MBIO1174.4|Congridae
Conger cinereus|MBIO428.4|Congridae
Conger cinereus|MBIO1175.4|Congridae
Moringua sp. 1|MBIO63.4|Moringuidae
Moringua sp. 1|MBIO156.4|Moringuidae
Moringua sp. 1|MBIO157.4|Moringuidae
Moringua sp. 2|MBIO62.4|Moringuidae
Moringua sp. 3|MBIO841.4|Moringuidae
Moringua sp. 3|MBIO842.4|Moringuidae
Moringua sp. 3|MBIO846.4|Moringuidae
Myrichthys colubrinus|MBIO1489.4|Ophichthidae
Brachysomophis henshawi|MBIO130.4|Ophichthidae
Leiuranus semicinctus|MBIO511.4|Ophichthidae
Leiuranus semicinctus|MBIO510.4|Ophichthidae
Callechelys catostoma|MBIO1477.4|Ophichthidae
Callechelys marmorata|MBIO1482.4|Ophichthidae
Callechelys marmorata|MBIO1243.4|Ophichthidae
Callechelys marmorata|MBIO1504.4|Ophichthidae
Callechelys marmorata|MBIO1483.4|Ophichthidae
Ophichthus polyophthalmus|MBIO1009.4|Ophichthidae
Ophichthus sp.|MBIO1177.4|Ophichthidae
Heteroconger lentiginosus|MBIO1766.4|Congridae
Apterichtus australis|MBIO155.4|Ophichthidae
Muraenichthys sp. 2|MBIO242.4|Ophichthidae
Scolecenchelys gymnota|MBIO1500.4|Ophichthidae
Scolecenchelys gymnota|MBIO1239.4|Ophichthidae
Asterropteryx ensifera|MBIO639.4|Gobiidae
Asterropteryx semipunctata|MBIO1754.4|Gobiidae
Amblyeleotris katherine|MBIO1053.4|Gobiidae
Amblyeleotris katherine|MBIO1377.4|Gobiidae
Amblyeleotris katherine|MBIO1317.4|Gobiidae
Amblyeleotris katherine|MBIO1052.4|Gobiidae
Ctenogobiops feroculus|MBIO383.4|Gobiidae
Bathygobius coalitus|MBIO827.4|Gobiidae
Bathygobius cocosensis|MBIO823.4|Gobiidae
Bathygobius cocosensis|MBIO822.4|Gobiidae
Cheilio inermis|NBE1424|Labridae
Cheilio inermis|NBE1425|Labridae
Cheilio inermis|NBE0048|Labridae
Cheilio inermis|MBIO1456.4|Labridae
Cheilio inermis|MBIO1457.4|Labridae
Awaous ocellaris|MBIO1813.4|Gobiidae
Gnatholepis anjerensis|MBIO1525.4|Gobiidae
Gnatholepis anjerensis|MBIO404.4|Gobiidae
Gnatholepis anjerensis|MBIO1524.4|Gobiidae
Gnatholepis anjerensis|MBIO1514.4|Gobiidae
Gnatholepis cauerensis|MBIO598.4|Gobiidae
Gnatholepis cauerensis|MBIO597.4|Gobiidae
Callogobius sclateri|MBIO637.4|Gobiidae
Callogobius sclateri|MBIO368.4|Gobiidae
Callogobius sclateri|MBIO367.4|Gobiidae
Amanses scopas|MBIO1255.4|Monacanthidae
Amanses scopas|MBIO988.4|Monacanthidae
Amanses scopas|MBIO1256.4|Monacanthidae
Cantherhines dumerilii|MBIO692.4|Monacanthidae
Cantherhines dumerilii|MBIO500.4|Monacanthidae
Cantherhines dumerilii|MBIO1258.4|Monacanthidae
Cantherhines sandwichiensis|MBIO1551.4|Monacanthidae
Cantherhines sandwichiensis|MBIO60.4|Monacanthidae
Gobiodon quinquestrigatus|MBIO173.4|Gobiidae
Gobiodon quinquestrigatus|MBIO174.4|Gobiidae
Gobiodon quinquestrigatus|MBIO1340.4|Gobiidae
Gobiodon quinquestrigatus|MBIO1341.4|Gobiidae
Gobiodon unicolor|MBIO1365.4|Gobiidae
Paragobiodon lacunicolus|MBIO379.4|Gobiidae
Paragobiodon lacunicolus|MBIO380.4|Gobiidae
Paragobiodon modestus|MBIO354.4|Gobiidae
Paragobiodon modestus|MBIO353.4|Gobiidae
Paragobiodon modestus|MBIO181.4|Gobiidae
Paragobiodon modestus|MBIO182.4|Gobiidae
Coryphopterus duospilus|MBIO1338.4|Gobiidae
Coryphopterus duospilus|MBIO1337.4|Gobiidae
Coryphopterus neophytus|MBIO1779.4|Gobiidae
Coryphopterus neophytus|MBIO1780.4|Gobiidae
Gymnapogon urospilotus|MBIO601.4|Apogonidae
Gymnapogon urospilotus|MBIO602.4|Apogonidae
Bathygobius cotticeps|MBIO1692.4|Gobiidae
Bathygobius cotticeps|MBIO1736.4|Gobiidae
Apogonichthys ocellatus|MBIO739.4|Apogonidae
Cabillus tongarevae|MBIO409.4|Gobiidae
Cabillus tongarevae|MBIO410.4|Gobiidae

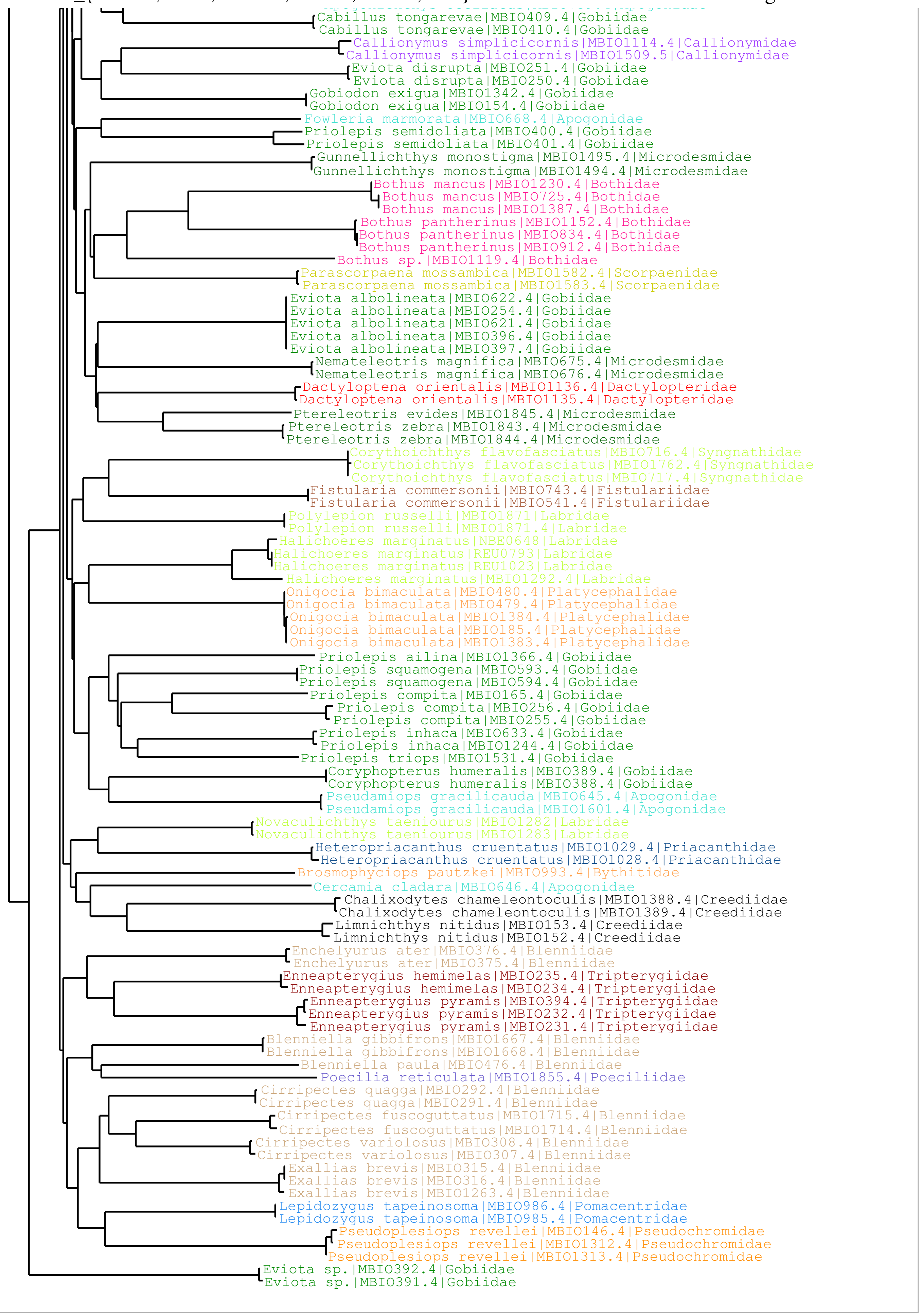
Cabillus tongarevae|MBIO409.4|Gobiidae
Cabillus tongarevae|MBIO410.4|Gobiidae
Callionymus simplicicornis|MBIO1114.4|Callionymidae
Callionymus simplicicornis|MBIO1509.5|Callionymidae
Eviota disrupta|MBIO251.4|Gobiidae
Eviota disrupta|MBIO250.4|Gobiidae
Gobiodon exigua|MBIO1342.4|Gobiidae
Gobiodon exigua|MBIO154.4|Gobiidae
Fowleria marmorata|MBIO668.4|Apogonidae
Priolepis semidoliata|MBIO400.4|Gobiidae
Priolepis semidoliata|MBIO401.4|Gobiidae
Gunnellichthys monostigma|MBIO1495.4|Microdesmidae
Gunnellichthys monostigma|MBIO1494.4|Microdesmidae
Bothus mancus|MBIO1230.4|Bothidae
Bothus mancus|MBIO725.4|Bothidae
Bothus mancus|MBIO1387.4|Bothidae
Bothus pantherinus|MBIO1152.4|Bothidae
Bothus pantherinus|MBIO834.4|Bothidae
Bothus pantherinus|MBIO912.4|Bothidae
Bothus sp.|MBIO1119.4|Bothidae
Parascorpaena mossambica|MBIO1582.4|Scorpaenidae
Parascorpaena mossambica|MBIO1583.4|Scorpaenidae
Eviota albolineata|MBIO622.4|Gobiidae
Eviota albolineata|MBIO254.4|Gobiidae
Eviota albolineata|MBIO621.4|Gobiidae
Eviota albolineata|MBIO396.4|Gobiidae
Eviota albolineata|MBIO397.4|Gobiidae
Nemateleotris magnifica|MBIO675.4|Microdesmidae
Nemateleotris magnifica|MBIO676.4|Microdesmidae
Dactyloptena orientalis|MBIO1136.4|Dactylopteridae
Dactyloptena orientalis|MBIO1135.4|Dactylopteridae
Ptereleotris evides|MBIO1845.4|Microdesmidae
Ptereleotris zebra|MBIO1843.4|Microdesmidae
Ptereleotris zebra|MBIO1844.4|Microdesmidae
Corythoichthys flavofasciatus|MBIO716.4|Syngnathidae
Corythoichthys flavofasciatus|MBIO1762.4|Syngnathidae
Corythoichthys flavofasciatus|MBIO717.4|Syngnathidae
Fistularia commersonii|MBIO743.4|Fistulariidae
Fistularia commersonii|MBIO541.4|Fistulariidae
Polylepion russelli|MBIO1871|Labridae
Polylepion russelli|MBIO1871.4|Labridae
Halichoeres marginatus|NBE0648|Labridae
Halichoeres marginatus|REU0793|Labridae
Halichoeres marginatus|REU1023|Labridae
Halichoeres marginatus|MBIO1292.4|Labridae
Onigocia bimaculata|MBIO480.4|Platycephalidae
Onigocia bimaculata|MBIO479.4|Platycephalidae
Onigocia bimaculata|MBIO1384.4|Platycephalidae
Onigocia bimaculata|MBIO185.4|Platycephalidae
Onigocia bimaculata|MBIO1383.4|Platycephalidae
Priolepis ailina|MBIO1366.4|Gobiidae
Priolepis squamogena|MBIO593.4|Gobiidae
Priolepis squamogena|MBIO594.4|Gobiidae
Priolepis compita|MBIO165.4|Gobiidae
Priolepis compita|MBIO256.4|Gobiidae
Priolepis compita|MBIO255.4|Gobiidae
Priolepis inhaca|MBIO633.4|Gobiidae
Priolepis inhaca|MBIO1244.4|Gobiidae
Priolepis triops|MBIO1531.4|Gobiidae
Coryphopterus humeralis|MBIO389.4|Gobiidae
Coryphopterus humeralis|MBIO388.4|Gobiidae
Pseudamiops gracilicauda|MBIO645.4|Apogonidae
Pseudamiops gracilicauda|MBIO1601.4|Apogonidae
Novaculichthys taeniourus|MBIO1282|Labridae
Novaculichthys taeniourus|MBIO1283|Labridae
Heteropriacanthus cruentatus|MBIO1029.4|Priacanthidae
Heteropriacanthus cruentatus|MBIO1028.4|Priacanthidae
Brosmophyciops pautzkei|MBIO993.4|Bythitidae
Cercamia cladara|MBIO646.4|Apogonidae
Chalixodytes chameleontoculis|MBIO1388.4|Creediidae
Chalixodytes chameleontoculis|MBIO1389.4|Creediidae
Limnichthys nitidus|MBIO153.4|Creediidae
Limnichthys nitidus|MBIO152.4|Creediidae
Enchelyurus ater|MBIO376.4|Blenniidae
Enchelyurus ater|MBIO375.4|Blenniidae
Enneapterygius hemimelas|MBIO235.4|Tripterygiidae
Enneapterygius hemimelas|MBIO234.4|Tripterygiidae
Enneapterygius pyramis|MBIO394.4|Tripterygiidae
Enneapterygius pyramis|MBIO232.4|Tripterygiidae
Enneapterygius pyramis|MBIO231.4|Tripterygiidae
Blenniella gibbifrons|MBIO1667.4|Blenniidae
Blenniella gibbifrons|MBIO1668.4|Blenniidae
Blenniella paula|MBIO476.4|Blenniidae
Poecilia reticulata|MBIO1855.4|Poeciliidae
Cirripectes quagga|MBIO292.4|Blenniidae
Cirripectes quagga|MBIO291.4|Blenniidae
Cirripectes fuscoguttatus|MBIO1715.4|Blenniidae
Cirripectes fuscoguttatus|MBIO1714.4|Blenniidae
Cirripectes variolosus|MBIO308.4|Blenniidae
Cirripectes variolosus|MBIO307.4|Blenniidae
Exallias brevis|MBIO315.4|Blenniidae
Exallias brevis|MBIO316.4|Blenniidae
Exallias brevis|MBIO1263.4|Blenniidae
Lepidozygus tapeinosoma|MBIO986.4|Pomacentridae
Lepidozygus tapeinosoma|MBIO985.4|Pomacentridae
Pseudoplesiops revellei|MBIO146.4|Pseudochromidae
Pseudoplesiops revellei|MBIO1312.4|Pseudochromidae
Pseudoplesiops revellei|MBIO1313.4|Pseudochromidae
Eviota sp.|MBIO392.4|Gobiidae
Eviota sp.|MBIO391.4|Gobiidae
