# Supplementary figures and images for: Cryptic Diversity in Indo-Pacific Coral-Reef Fishes Revealed by DNA-Barcoding Provides New Support to the Centre-of-Overlap Hypothesis
Source: PLoS One. 2012 Mar 15;7(3):e28987. doi: 10.1371/journal.pone.0028987 (PMC3305298; doi:10.1371/journal.pone.0028987)

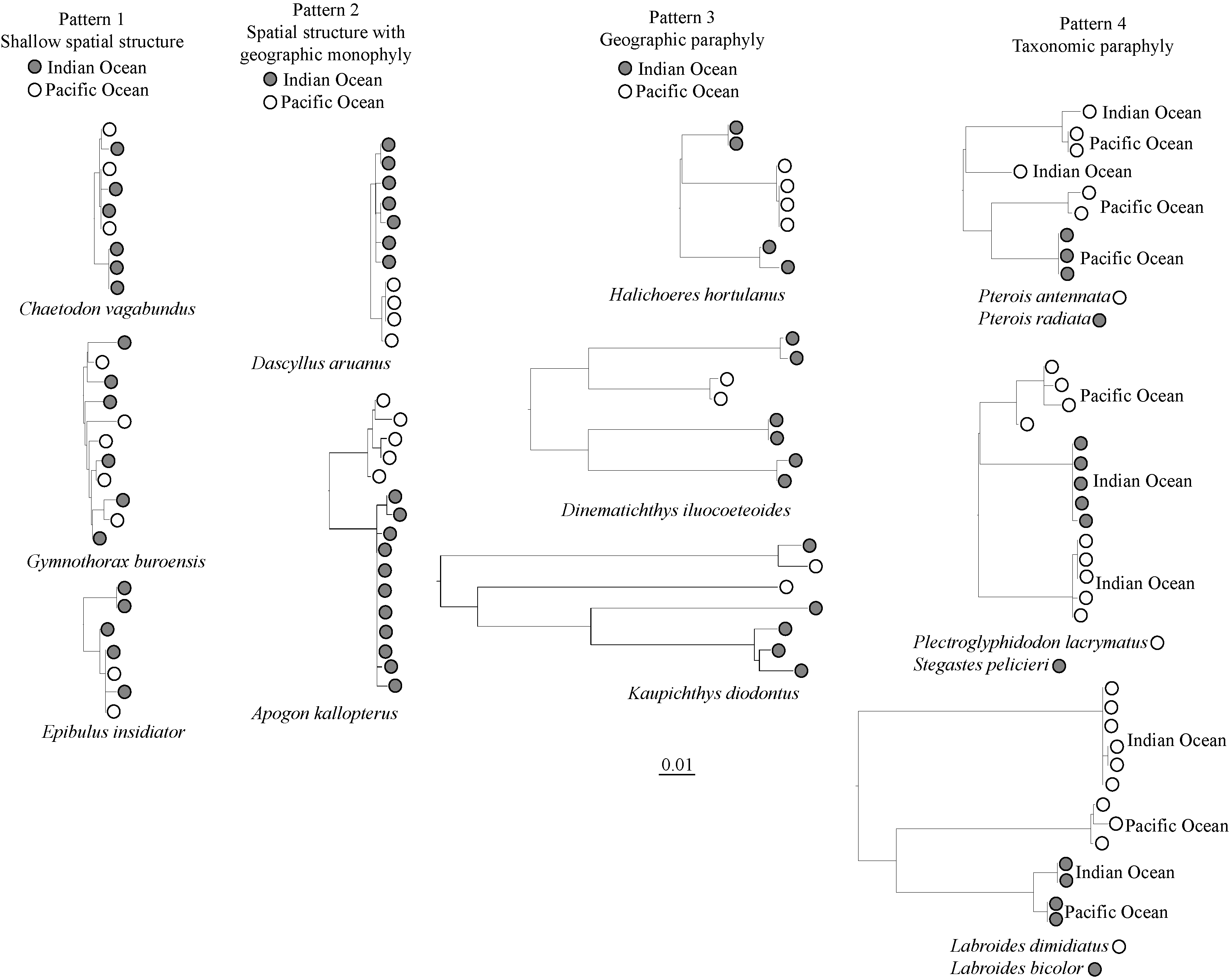

Supplement: Figure S2 — Description of the four pattern of COI barcodes distribution in species with Indo-Pacific range distribution. (TIF) [file pone.0028987.s005.tif]
